# Metatranscriptome Sequencing Reveals Insights into the Gene Expression and Functional Potential of Rumen Wall Bacteria

**DOI:** 10.3389/fmicb.2018.00043

**Published:** 2018-01-23

**Authors:** Evelyne Mann, Stefanie U. Wetzels, Martin Wagner, Qendrim Zebeli, Stephan Schmitz-Esser

**Affiliations:** ^1^Department for Farm Animals and Veterinary Public Health, Institute of Milk Hygiene, Milk Technology and Food Science, University of Veterinary Medicine Vienna, Vienna, Austria; ^2^Department for Farm Animals and Veterinary Public Health, Institute of Animal Nutrition and Functional Plant Compounds, University of Veterinary Medicine Vienna, Vienna, Austria; ^3^Department of Animal Science, Iowa State University, Ames, IA, United States

**Keywords:** epimural microbiota, subacute rumen acidosis, metatranscriptome, dairy cattle, rumen epithelium

## Abstract

Microbiota of the rumen wall constitute an important niche of rumen microbial ecology and their composition has been elucidated in different ruminants during the last years. However, the knowledge about the function of rumen wall microbes is still limited. Rumen wall biopsies were taken from three fistulated dairy cows under a standard forage-based diet and after 4 weeks of high concentrate feeding inducing a subacute rumen acidosis (SARA). Extracted RNA was used for metatranscriptome sequencing using Illumina HiSeq sequencing technology. The gene expression of the rumen wall microbial community was analyzed by mapping 35 million sequences against the Kyoto Encyclopedia for Genes and Genomes (KEGG) database and determining differentially expressed genes. A total of 1,607 functional features were assigned with high expression of genes involved in central metabolism, galactose, starch and sucrose metabolism. The glycogen phosphorylase (EC:2.4.1.1) which degrades (1->4)-alpha-D-glucans was among the highest expressed genes being transcribed by 115 bacterial genera. Energy metabolism genes were also highly expressed, including the pyruvate orthophosphate dikinase (EC:2.7.9.1) involved in pyruvate metabolism, which was covered by 177 genera. Nitrogen metabolism genes, in particular glutamate dehydrogenase (EC:1.4.1.4), glutamine synthetase (EC:6.3.1.2) and glutamate synthase (EC:1.4.1.13, EC:1.4.1.14) were also found to be highly expressed and prove rumen wall microbiota to be actively involved in providing host-relevant metabolites for exchange across the rumen wall. In addition, we found all four urease subunits (EC:3.5.1.5) transcribed by members of the genera *Flavobacterium*, *Corynebacterium*, *Helicobacter*, *Clostridium*, and *Bacillus*, and the dissimilatory sulfate reductase (EC 1.8.99.5) *dsrABC*, which is responsible for the reduction of sulfite to sulfide. We also provide *in situ* evidence for cellulose and cellobiose degradation, a key step in fiber-rich feed digestion, as well as oxidative stress response and oxygen scavenging at the rumen wall. Archaea, mainly *Methanocaldococcus* and *Methanobrevibacter*, were found to be metabolically active with a high number of transcripts matching to methane and carbohydrate metabolism. These findings enhance our understanding of the metabolic function of the bovine rumen wall microbiota.

## Introduction

Ruminants are crucial for humans by producing milk and meat as major protein sources for human nutrition. They are characterized by their unique mode of digesting plants in their forestomach, the rumen. The rumen is densely populated by different microorganisms which are central for the breakdown of plant material which cannot be degraded by the ruminant itself. The symbiosis of ruminants and their microbiota is essential for ruminal function and enteric fermentation processes. The intimate association of ruminants and their symbiotic microbes is a result of evolutionary coexistence, and is central for energy harvest from otherwise indigestive plant material. The rumen wall, the interface between the rumen content and the host animal, has important physiological roles for the host in energy absorption, metabolism and transport of nutrients and might be associated with changes in feed efficiency (Remond et al., 1995; Kong et al., 2016). The rumen wall is covered by microorganisms that are directly attached to the rumen epithelium (Cheng et al., 1979). These bacteria are also known as “epimural” bacteria or microbiota (Mead and Jones, 1981). Many essential metabolites, such as VFA, ammonia, urea and minerals are exchanged across the rumen wall (Rémond et al., 1995), making the epimural microbiota being exposed to – and possibly participating in these nutrient exchanges.

In the last decade, a number of studies have yielded insights into the composition of the epimural microbiota in cattle and revealed that epimural bacteria are largely distinct from the microbial community in the rumen content (Malmuthuge et al., 2014; Mao et al., 2015; Liu et al., 2016). While these studies provided valuable insights into the composition of these microbial communities, our knowledge about the function of epimural bacteria and archaea is still very limited. It is known that epimural bacteria are involved in the hydrolysis of urea (Abdel Rahman, 1966; Wallace et al., 1979; Jin et al., 2017), the scavenging of oxygen (Cheng et al., 1979; Wallace et al., 1979) and in epithelial tissue recycling (Mccowan et al., 1978). It has been suggested that epimural bacteria might be involved in amino acid metabolism, using a function-predicting tool (Mao et al., 2015). Epimural microbiota may also compete with adhesive pathogenic microorganisms and they can form a protective layer on the ruminal epithelium (Hungate, 1966; Kamra, 2005).

High production dairy cows are challenged by feeding more concentrates and less forage to support high milk yields, which can result in a ruminal accumulation of VFA and a reduced buffering capacity (Kleen et al., 2003; Rustomo et al., 2006; Plaizier et al., 2008). In consequence, a depression of the rumen pH can lead to subacute ruminal acidosis (SARA), a critical metabolic disorder of cattle (Nocek, 1997; Kleen et al., 2003; AlZahal et al., 2007; Zebeli and Metzler-Zebeli, 2012).

Previously, we have performed a long-term SARA experiment with rumen-cannulated non-lactating Holstein cows fed forage (grass silage-hay mix) and increasing concentrate amounts. At the baseline, before concentrate was fed, and during the SARA challenges, rumen papillae biopsies were taken and the epimural bacterial microbiota were determined using Illumina MiSeq sequencing of 16S rRNA genes. SARA led to a decrease in diversity and species richness and induced shifts in the epimural bacterial community structure (Wetzels et al., 2016, 2017). Although this dataset revealed first insights into the composition and the complexity of the epimural bacterial microbiome, no conclusions about the metabolically active part or the functional potential of the epimural microbiome could be drawn. Transcriptome profiling of the host animal rumen epithelial tissue has gained attention by the scientific community in the last few years (Baldwin et al., 2012; Kong et al., 2016; Zhang et al., 2016; Wang et al., 2017), but functional data for the rumen wall microbiota is still missing.

Our metatranscriptome sequencing approach had two aims: the main aim was to obtain insights into the gene expression of epimural microbiota in general and secondly to determine changes in the gene expression of the epimural microbiota in response to a dietary shift from forage-based feeding to high concentrate diet induced SARA conditions.

## Materials and Methods

### Experimental Design

A feeding experiment with three rumen-cannulated (100 mm inner diameter Bar Diamond, ID) multiparous non-lactating Holstein cows (3–4 parities; initial body weight: 710 ± 118 kg, mean ± SD) was conducted. A feeding model to induce a continuous and long-term SARA challenge was used as follows: 2-week baseline feeding, followed by 1-week gradual adaptation to a 60% concentrate diet, followed by 4-week continuous SARA challenge with 60% concentrate feeding. Experimental animals were housed together in a free stall barn at the research farm of the University of Veterinary Medicine Vienna in Pottenstein, Austria. During the baseline period, cows were fed forage only, consisting of 50% grass silage and 50% second-cut meadow hay (dry matter basis) (Wetzels et al., 2016). During the adaptation period and SARA challenge a concentrate mixture was fed in separate and controlled feeding troughs (RIC system; Insentec B.V., Marknesse, Netherlands) additionally to the forage. The concentrate mixture consisted of barley (33.0%), wheat (30.0%), corn (15.0%), rapeseed meal (17.0%), dried beet pulp (3.2%), calcium carbonate (0.5%), NaCl (0.3%), and a mineral-vitamin premix for cattle (1.0%). During the adaptation period, the concentrate amount was increased by 10% daily and remained at 60% during the 4-week SARA challenge (Wetzels et al., 2017). Daily concentrate and forage intake were electronically recorded. Cows that did not consume their planned concentrate allowance, were force-fed the residual concentrate through the rumen cannula to ensure the intake of a 60:40 concentrate:forage ratio during the entire SARA challenge period. Ruminal pH was monitored via ruminal pH-sensors (smaXtec animal care sales GmbH, Graz, Austria), which were manually introduced into the bottom of ventral rumen via cannula in each cow, as described in Pourazad et al. (2015). Criterion for definition of SARA was a rumen pH below 5.8 for at least 330 min/d (Zebeli et al., 2008).

The experimental setup was approved by the institutional ethics committee of the University of Veterinary Medicine Vienna in accordance with Good Scientific Practice guidelines and the national authority according to §26 of Law for Animal Experiments, Tierversuchsgesetz – TVG 2012 (GZ 68.205/0093-II/3b/2013).

### Rumen Papillae Sampling

Rumen papillae biopsy samples were taken at the end of the baseline period and at the last day of the 4-week SARA challenge. Biopsies were taken 1 h after the morning forage feeding and before concentrates were fed during the SARA challenge. Rumen papillae biopsies were taken from the rumen wall of the ventral sac about 40–50 cm below the bottom edge of the rumen cannula located in the left *Fossa paralumbalis* (Wetzels et al., 2016). The rumen wall was briefly rinsed with sterile 1 × PBS to remove adhering feed particles before cutting the papillae aseptically. Rumen papillae were immediately shock-frozen in liquid nitrogen and stored at -80°C until further processing.

### RNA Extraction, HiSeq 2500 Sequencing and Data Analysis

For RNA extraction, per sample, a total of 100 mg ruminal papillae tissue was homogenized for 2.5 min in 500 μl TRIzol^TM^ reagent (Invitrogen) using a mortar. After homogenization, another 500 μl TRIzol^TM^ reagent was added and RNA extraction was done according to the manufacturer’s recommendations. The RNA pellet was dissolved in 15 μl ddH_2_O_DEPC_. Contaminating genomic DNA was digested with the TURBO DNA-free Kit (Ambion) and the completeness of DNA digestion was confirmed by PCR with a 16S rRNA gene targeting bacterial primer pair. The RNA integrity was measured with an Agilent 2100 Bioanalyzer (Agilent Technologies) and RNA was stored at -80°C until use. RNA samples were subjected to standard Illumina library preparation with the NEBNext^®^ Ultra^TM^ RNA Library Prep Kit (Illumina). rRNA was removed with the Ribo-Zero^TM^ Magnetic Gold (Epidemiology) Kit (Epicentre Biotechnologies). Six double-stranded cDNA libraries, two for each cow (one baseline and one SARA sample, respectively) were created. Two libraries were pooled on one lane and sequenced using an Illumina HiSeq2500 sequencer (Vienna Biocenter Core Facilities VBCF NGS Unit^1^) with a 125 bp read length paired-end protocol. Sequences were quality filtered with mothur (Schloss et al., 2009). The following trimming parameters were used: The number of ambiguous bases allowed was zero, the minimum length of reads accepted was 30 bp, the minimum average quality score (allowed over a window of 10 bp) was 25 and the maximum length of homopolymers was 8 bp; duplicate reads were removed.

Quality-filtered RNA sequences were processed and annotated using the MG-RAST (Meta Genome Rapid Annotation using Subsystem Technology; v4.0) server at the Argonne National Laboratories^2^ (Keegan et al., 2016). Artificial duplicate reads were removed (Cox et al., 2010) and host reads mapped to the genome of *Bos taurus* were filtered. Potential rRNA genes with a cut-off of 70% identity to ribosomal sequences were identified (Rognes et al., 2016) and sequences were clustered at 97% identity (Fu et al., 2012). After removal of rRNA sequences, putative protein coding features were predicted using FragGeneScan (Rho et al., 2010) and clustered at 90% identity. Protein similarity search against the M5NR protein database was done with BLAT (Kent, 2002). For post-processing taxonomic and functional analysis, the KEGG (Kanehisa and Goto, 2000; Kanehisa et al., 2016) and KO systems were used with an *e*-value cutoff of 1 × 10^-5^, identity cut-off value of 60% and a minimum alignment length of 15. The top 10% expressed genes were considered as highly expressed, the last 50% of all genes were considered as lowly expressed. All other genes were defined to be moderately expressed.

### Data Availability

Raw sequence data are available for download from the NCBI Sequence Read Archive (Accession no. PRJEB22186) and processed data is stored at the MG-RAST server (MG-RAST IDs 4712547.3, 4712551.3, 4712550.3, 4712552.3, 4712549.3, 4712548.3; MG-RAST project ID mgp19117).

### Statistical Analysis

Taxonomic and functional data was imported in DeSeq Bioconductor using the R software environment (R Development Core Team, 2008). Data were normalized to size factors of libraries and dispersion estimation and were listed as normalized read counts per feature. Differentially expressed transcripts and significantly shifted abundances of phylotypes were determined by DeSeq using a binomial distribution model (Anders and Huber, 2010), including all features in one input file submitted to R. A feature was considered significant if *p* < 0.05 and if multiple testing correction of FDR < 5% (Benjamini and Hochberg, 1995).

## Results

### Sequencing and Annotation of Metatranscriptome Datasets

After merging the corresponding paired-end reads, 560 million sequences with an average read length of 185 bp were obtained, with a mean of 93.4 million reads per sample. After quality control and removal of sequencing artifacts and duplicates, 28.95% of reads remained on average for further processing. Host reads were removed, resulting in a mean of 5.9 million unique sequences per sample for all further downstream analysis. Predicted features could be assigned to 98.09–100% of these reads. Out of all predicted features, samples contained 2.33–7.74% rRNA transcripts. For further analysis on a functional or taxonomic level, normalized mRNA read counts assigned to known functions or taxa, respectively, were used (Supplementary Table S1).

### Taxonomic Distribution

On average, 604,061 reads mapped to bacteria, 30,486 reads mapped to archaea, and 77,087 reads mapped to fungi. No reads mapping to protozoa were detected.

**FIGURE 1:**
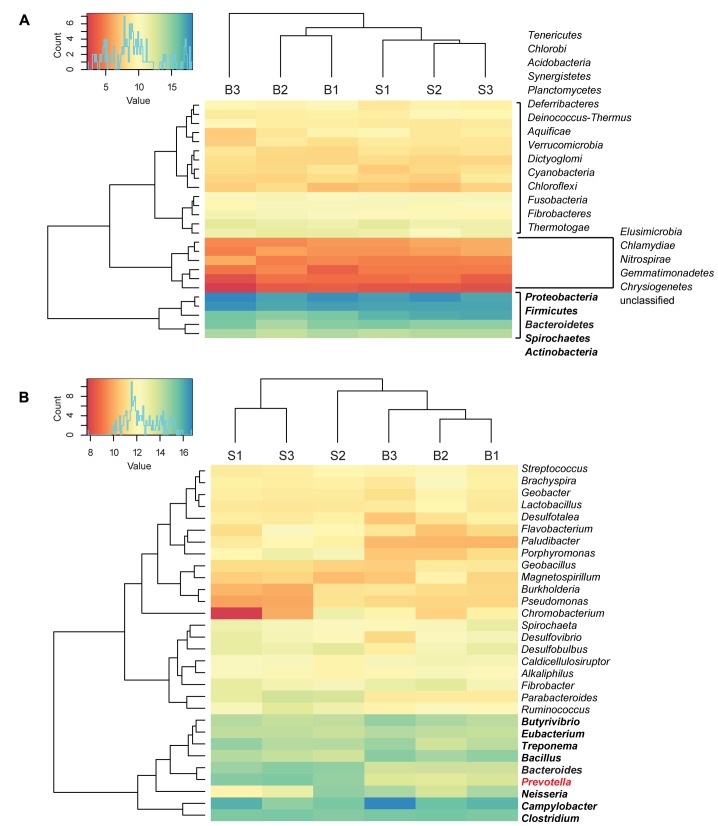
Taxonomic distribution of bacterial reads. Read count values are given as transformed log_2_ values of normalized counts per phylotype. Based on the dendrogram’s branching hierarchy, a ‘high abundance cluster’ was defined and marked in bold. Statistically significant differences of phylotypes between baseline (B) and SARA (S) samples were colored red. All phyla detected are shown in **(A)**, the 30 most abundant genera are shown in **(B)**.

Bacterial reads were assigned to 26 phyla and 420 genera. The most abundant phyla were *Proteobacteria*, *Firmicutes*, *Bacteroidetes*, *Spirochaetes*, and *Actinobacteria*. *Bacteroidetes* were statistically significantly enriched in the SARA samples compared with the baseline samples (2.6-fold-change, *q* < 0.001). At genus level, a cluster with highly abundant genera was detected, including *Butyrivibrio, Eubacterium, Bacillus, Treponema, Prevotella, Bacteroides, Neisseria, Campylobacter*, and *Clostridium*. From this high abundance cluster, *Prevotella* was statistically significantly enriched in the SARA samples when compared to the baseline samples (*q*-value = 0.02) (**Figure 1**). Statistically significant differences between SARA and baseline are indicated in **Table 1** and all genera are listed in Supplementary Table S2.

**Table 1:** Statistically significantly enriched genera in SARA samples compared with baseline samples.

Phylum	Genus	Baseline counts	SARA counts	Fold change	*p-*value	*q*-value
*Bacteroidetes*	*Prevotella*	9,620	34,738	3.61	<0.001	0.017
*Bacteroidetes*	*Parabacteroides*	3,641	10,257	2.82	<0.001	0.068
*Bacteroidetes*	*Pedobacter*	934	2,616	2.80	0.001	0.068
*Bacteroidetes*	*Marivirga*	395	1,349	3.42	0.001	0.068
*Bacteroidetes*	*Zunongwangia*	342	912	2.67	0.001	0.068
*Bacteroidetes*	*Leadbetterella*	330	872	2.65	0.001	0.097

*Counts are listed as normalized mRNA read counts. Duplicate reads were removed during quality control. A q-value of <0.05 indicates statistical significance and 0.05 ≤ q ≤ 0.1 indicates a trend.*

Archaeal reads were assigned to 5 phyla and 50 genera. The most abundant phylum was *Euryarchaeota* including two highly abundant methanogens: *Methanocaldococcus* and *Methanobrevibacter*. *Methanocaldococcus* was enriched by one log unit compared to other archaeal genera in all baseline and SARA samples (Supplementary Table S3). Between baseline and SARA samples, archaeal phyla and genera were not statistically significantly different.

Within fungi, all reads were assigned to two phyla: *Ascomycota* and *Basidiomycota*. In total, 28 genera belonged to *Ascomycota* with *Aspergillus*, *Neurospora* and *Debaryomyces* being most abundant. The *Basidiomycota* consisted of eight genera with *Filobasidiella*, *Coprinopsis*, *Laccaria*, *Ustilago*, and *Schizophyllum* being most abundant. No statistically significant differences were found between Baseline and SARA for fungal phylotypes.

### Overall Central and Bacterial Metabolism at the Rumen Wall

**FIGURE 2:**
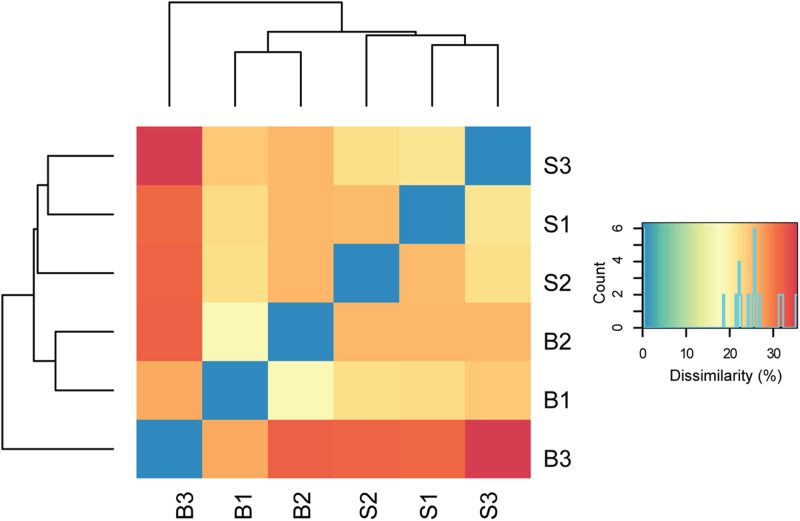
Dissimilarity of biological replicates and sampling time points. Euclidean distances between normalized read counts are shown, with blue indicating lowest distance. The dendrograms depict hierarchical clustering of replicates. Euclidean clustering was calculated based on all functional features detected (Supplementary Table S4). B1–B3 = baseline replicates, S1–S3 = SARA replicates.

**FIGURE 3:**
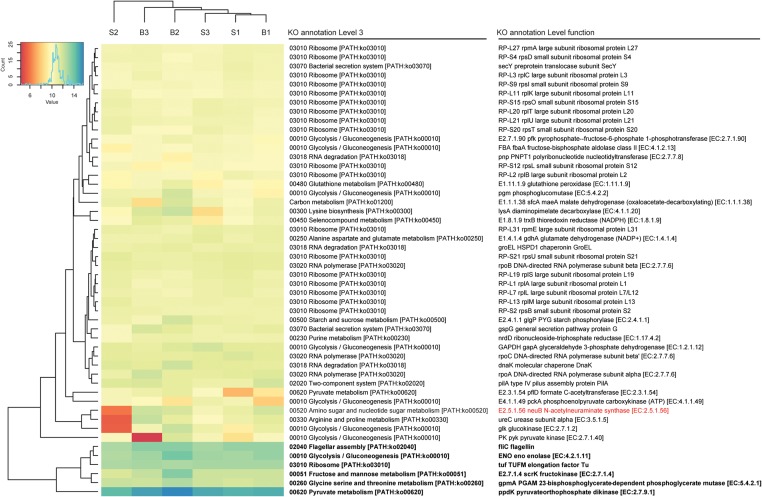
Functional bacterial hits. The 50 most abundant features of the KO annotation are listed. Read count values are given as transformed log_2_ values of normalized counts per feature. Based on the dendrogram’s branching hierarchy, a ‘high abundance cluster’ was defined and marked in bold. Statistically significant differences of features between baseline (B1–B3) and SARA (S1–S3) replicates were colored red.

In total, about 35 million sequences were annotated at the functional level using the KO database, resulting in 1,607 features at KO level function. Considering the expression levels of all functional features, replicate samples were similar and reproducible. This is shown by low Euclidean distances between replicates of baseline or SARA samples and clustering per sampling time point (**Figure 2**). SARA samples were more similar to each other than the baseline samples. The 50 most abundant functional genes are listed in **Figure 3**. In general, transcripts encoding for housekeeping genes involved in transcription, translation and chaperones showed highest levels of transcription.

A complete list of all functional features is shown in Supplementary Table S4, taxonomic hits are listed in Supplementary Table S5. High expression levels were found for the galactose metabolism and the starch and sucrose metabolism, where the glycogen phosphorylase (EC:2.4.1.1), responsible for degradation of different forms of (1->4)-alpha-D-glucans, such as starch, was among the highest expressed genes, and also the alpha-amylase (EC:3.2.1.1) was expressed. The glycogen phosphorylase was transcribed by 115 bacterial genera with most taxonomic hits belonging to the phyla *Proteobacteria*, *Firmicutes*, and *Actinobacteria*. The alpha-amylase was exclusively transcribed by *Bacteroidetes*, namely *Bacteroides* and *Prevotella*. Many genes involved in energy metabolism were also among the most highly expressed genes of the rumen wall microbiota including NADH dehydrogenases, F- and V-Type ATPases. The highest expressed gene was *ppdK* (EC:2.7.9.1), the pyruvate orthophosphate dikinase involved in pyruvate metabolism. It was expressed by 177 genera belonging to 17 phyla with most hits matching to *Firmicutes* (*Clostridium* and *Caldicellulosiruptor)* and *Proteobacteria* (*Magnetospirillum* and *Rhodospirillum*). *GpmA* (EC:5.4.2.11), a phosphoglycerate mutase involved in glycolysis and gluconeogenesis was also among the highest transcribed genes with taxonomic hits assigned to 23 phyla and 267 bacterial genera, mainly belonging to *Spirochaetes* (*Brachyspira* and *Treponema*), *Actinobacteria* (*Atopobium*) and *Proteobacteria* (*Cronobacter* and *Bdellovibrio*). The fructokinase (EC.2.7.1.4) used in the metabolism of fructose, mannitol and sorbitol was also among the most highly transcribed genes with taxonomy hits mainly belonging to *Firmicutes* (*Butyrivibrio, Eubacterium* and *Geobacillus*). Also enolase (EC.4.2.1.11), having a key position in glycolysis and gluconeogenesis, was among the highest transcribed genes. It was mostly transcribed by *Spirochaetes* (*Borrelia*), *Bacteroidetes* (*Prevotella* and *Bacteroides*) and *Deinococcus-Thermus* (*Thermotoga*).

Particularly nitrogen metabolism is of high relevance in the rumen with ammonia and urea being highly important metabolites which are also exchanged across the rumen wall. In line with this, glutamate dehydrogenase (EC:1.4.1.4), glutamine synthetase (EC:6.3.1.2), and glutamate synthase (EC:1.4.1.13, EC:1.4.1.14) were found to be highly or moderately expressed by the rumen wall microbiota. The glutamate dehydrogenase and the glutamine synthetase were widely expressed by bacteria (118 and 116 taxonomic hits, respectively), being most highly expressed by *Campylobacter*, *Neisseria*, and *Bacteroides.* The glutamate synthase, expressed by 66 genera, was most highly expressed by *Clostridium* and *Treponema*. Furthermore, we found expression of a number of genes involved in protein and amino acid degradation such as various peptidases and also tryptophanase (EC:4.1.99.1). The tryptophanase was mainly expressed by *Fusobacteria* and *Bacteroides*. Dissimilatory nitrate reduction is an energy-generating process converting nitrate to ammonia. Gene expression of three nitrogenase gene subunits (*nifD*, *nifH*, and *nifK*, EC:1.18.6.1) was detected, although at low expression level. All three subunits were transcribed by the genera *Fibrobacter* and *Clostridium*.

The periplasmic nitrate reductase *napA* (EC.1.7.99.4) was highly expressed by the genus *Campylobacter*.

Urease converts urea to ammonia and CO_2_; all four urease subunits (EC:3.5.1.5) were found to be transcribed in the rumen wall microbial community, some of which were among the highest transcribed features (Supplementary Table S4). Expressed urease sequences were found to affiliate mainly to the genera *Flavobacterium*, *Corynebacterium*, *Helicobacter*, *Clostridium*, and *Bacillus*.

Genes involved in degradation of cellulose such as endoglucanase (EC:3.2.1.4) and cellobiose such as cellobiose phosphorylase (EC:2.4.1.20) and beta-glucosidase (EC:3.2.1.21) were moderately to highly expressed. However, only few taxonomic hits were received, with the genera *Fibrobacter* and *Clostridium* being mostly represented.

We could also show expression of dissimilatory sulfate reductase (EC 1.8.99.5) *dsrABC*, which is responsible for the reduction of sulfite to sulfide. *DsrABC* was mainly expressed by the *Proteobacteria*, *Desulfotalea*, and *Desulfurivibrio*.

Furthermore, the rumen wall microbial community expressed various transporters for the uptake of: sugars including maltose, ribose, methyl-galactoside and xylose, polyamines such as spermidine and putrescine, amino acids such as glutamate, glutamine, methionine, and branched-chain amino acids as well as for trace elements such as iron, molybdate, zinc, cobalt, and nickel.

In total, five genes involved in oxidative stress response were detected: The thioredoxin reductase NADPH (EC:1.8.1.9), the glutathione peroxidase (EC:1.11.1.9) and the superoxide dismutase (EC:1.15.1.1) were among the highest transcribed features. The catalase peroxidase (EC:1.11.1.21) and the catalase (EC:1.11.1.6) were detected with low expression levels. The thioredoxin reductase was highest expressed by the genera *Campylobacter, Atopobium*, and *Bifidobacterium.* The glutathione peroxidase was highest expressed by *Fibrobacter, Prevotella, Clostridium, Fusobacterium*, and *Butyrivibrio.* The superoxide dismutase had a high number of taxonomy hits for *Bacillus, Clostridium, Neisseria*, and *Butyrivibrio*. The lowly expressed catalase was mainly expressed by *Desulfovibrio* and *Bordetella* and the catalase peroxidase was expressed mainly by *Vibrio*.

Interestingly, we found high levels of transcription of flagellar and chemotaxis-associated genes. *FliC* was highest expressed by the genera *Butyrivibrio, Treponema, Eubacterium, Clostridium*, and *Spirochaeta.* Also, the type II, Sec-dependent and the twin arginine targeting (TAT) secretion systems were found to be highly expressed. Some genes of the types II, IV, and VI secretion systems were also transcribed, although at a lower levels.

### Central Archaeal Metabolism at the Rumen Wall

Two highly abundant archaeal genera were detected: *Methano caldococcus* and *Methanobrevibacter*. *Methanocaldococcus* had a high number of transcripts belonging to glycan biosynthesis (e.g., the *N*-acetylglucosaminephosphotransferase EC:2.7.8.15), methane metabolism [e.g., *ftr*, (EC:2.3.1.101), *ehbQ* and *mcrA* (EC:2.8.4.1)] and carbohydrate metabolism (pentose phosphate pathway and fructose and mannose metabolism) being highly transcribed. *Methanobrevibacter* had exclusively high functional hits associated with methane metabolism, e.g., *mcrA*, *mcrB*, and *mcrG* (EC:2.8.4.1), *hdrA* (EC:1.8.98.1), and *mer* (EC:1.5.99.11), and glyoxylate and dicarboxylate metabolism (EC:1.2.1.2). Besides *Methanobrevibacter* and *Methanocaldococcus*, the genera *Methanococcoides*, *Methanohalobium, Methanosarcina*, and *Methanothermobacter* were found to be involved in methane metabolism at the rumen wall.

### Differences between Baseline and SARA Periods

Out of 1,607 KO features (level function), only one has a statistically significantly higher transcription in the SARA samples when compared to the baseline samples. One gene was significantly more highly transcribed in the baseline samples compared with SARA samples (**Table 2**). It should be noted that out of statistically significantly different enriched features, only the *N*-acetylneuraminate synthase was highly transcribed, all other genes showed low expression levels.

**Table 2:** KEGG orthology annotation of features with statistically significant shifts between baseline and SARA samples.

KO level 2	KO level 3	KO level function	Baseline counts	SARA counts	Fold change	*p*-value	*q*-value
Membrane_transport	02010 ABC transporters [PATH:ko02010]	malE maltose/maltodextrin transport system substrate-binding protein	21	104	5.0	<0.001	0.013
Carbohydrate_metabolism	00650 Butanoate metabolism [PATH:ko00650]	bdh 3-hydroxybutyrate dehydrogenase [EC:1.1.1.30]	374	149	2.5	0.000	0.081
Carbohydrate_metabolism	00520 Amino sugar and nucleotide sugar metabolism [PATH:ko00520]	neuB *N*-acetylneuraminate synthase [EC:2.5.1.56]	3,424	1,229	2.8	<0.001	0.013

*Counts are listed as normalized read counts. Duplicate reads were removed during quality control. Only features showing more than 100 read counts in one of the conditions are shown. A q-value of <0.05 indicates statistical significance and 0.05 ≤ q ≤ 0.1 indicates a trend.*

### Transcript Enrichment Analysis

**FIGURE 4:**
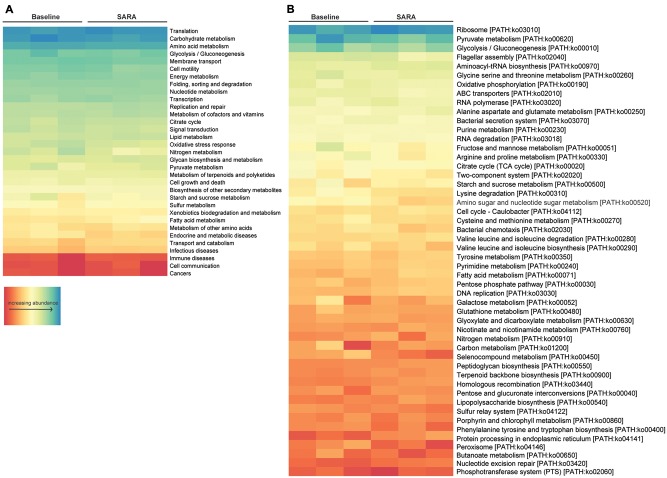
Enrichment analysis of all bacterial features. Features are ordered by decreasing abundance. **(A)** Enrichment on level 2 and **(B)** enrichment on level 3 of KO annotation.

Gene set enrichment analysis of central metabolic pathways based on KO level 2 showed that translation, carbohydrate metabolism and amino acid metabolism, as well as glycolysis/gluconeogenesis and membrane transport associated genes were highly represented. Analyzed per pathway (KO level 3), ribosomes, pyruvate metabolism, glycolysis/gluconeogenesis and the flagellar assembly pathway were highest expressed. Starch and sucrose-, fatty acid-, nitrogen-, and carbon metabolism was present in all samples, although being moderately to low expressed (**Figure 4**).

## Discussion

In a recent companion study, belonging to the same feeding experiment as the current one, we analyzed the composition of the epimural bacteria using DNA-based 16S amplicon sequencing within the same SARA feeding model, using eight Holstein cows (Wetzels et al., 2017). A cross-over design was used; thus, each animal served as its own control. Overall, SARA induced major shifts in the bacterial communities (Wetzels et al., 2017). Here, our untargeted RNA-based metatranscriptome sequencing approach revealed that on phylum level, *Proteobacteria*, *Firmicutes*, and *Bacteroidetes* were most abundant and *Bacteroidetes* showed statistically significantly higher abundance in SARA conditions, which is similar to what we found in our previous DNA-based amplicon sequencing study using samples from the same time points and animals (Wetzels et al., 2017). A reduction of bacterial diversity when transitioning from a fiber-based to a starch-based diet – as found in the current study – has been reported previously (Belanche et al., 2012; Plaizier et al., 2017).

Bacteria belonging to the genera *Clostridium*, *Campylobacter*, *Neisseria*, *Prevotella*, *Bacteroides*, *Treponema*, *Eubacterium*, and *Butyrivibrio* were found to be the most abundant phylotypes on the rumen wall based on metatranscriptome sequencing. Among these, *Campylobacter* and *Clostridium* were also highly abundant in the epimural bacterial community in the 16S rRNA gene DNA-based datasets. *Bacteroides, Desulfobulbus*, and *Desulfovibrio* and *Eubacterium* were less abundant but among the 50 most abundant DNA-based phylotypes. Some genera with high abundance in the metatranscriptome dataset – such as *Prevotella*, *Bacillus*, *Neisseria*, *Treponema*, or *Butyrivibrio* – were not detected with a high abundance in our previous 16S amplicon sequencing study (Wetzels et al., 2017), which is most likely explained by the two fundamentally different methodologies used in the studies. Here, as we have applied an untargeted activity (RNA)-based approach, we identify only metabolically active phylotypes, whereas in the DNA-based amplicon sequencing, also inactive bacteria or DNA from dead cells can be amplified and detected. In addition, the phylogenetic assignment of reads is different between these two approaches. Nevertheless, overall these two different methods provide a similar overview on the epimural bacterial communities. In accordance with other studies (Shin et al., 2004b; Pei et al., 2010; De Mulder et al., 2016; Scharen et al., 2017), archaea were found to be present at the rumen wall, although at relatively low abundance (varying from 0.3 to 1% of all KEGG hits), as well as fungi (relative abundance 0.4%). Analysis of eukaryotic sequences is currently not supported by MG-RAST. Therefore, our results represent only an overview of the taxonomic distribution of fungal reads. Nevertheless, we found several fungal genera to be abundant and metabolically active at the rumen wall, of which only *Aspergillus* has been described in the rumen content before (Lund, 1974; Abrao et al., 2014, 2017). We found no anaerobic fungi affiliating to the phylum *Neocallimastigomycota* and no protozoa in our metatranscriptomics approach. The absence of protozoa on the rumen wall has already been described before (Shin et al., 2004a). In conclusion, we reveal only moderate changes in the composition of epimural communities during the switch from forage-only feeding to SARA conditions. Based on recent observations that the composition of rumen wall and rumen content microbial communities is different (Mao et al., 2015) and we did not analyze rumen content microbial gene expression, we want to emphasize that no conclusions regarding the microbiota in the rumen content can be drawn from our results.

In a next step, we were interested in analyzing the function of epimural microbes and possible changes of their gene expression from forage-only feeding to SARA conditions. A number of recent 16S rRNA gene-based sequencing studies have provided an extensive knowledge on the composition of rumen wall microbes in different ruminants under different management and feeding strategies (Petri et al., 2013; Malmuthuge et al., 2014; Mao et al., 2015; Scharen et al., 2017). Nevertheless, the knowledge about the function of rumen wall microbiota is still highly limited, as 16S rRNA gene based community surveys reveal only very limited functional insights into bacterial and archaeal communities. Recently, some studies have described the metatranscriptomes of rumen content (Kamke et al., 2016; Comtet-Marre et al., 2017; Li and Guan, 2017) and the transcriptomes of rumen epithelial tissues (Kong et al., 2016; Wang et al., 2016; Zhang et al., 2016); however, the latter was performed without analyzing microbial gene expression.

We found high levels of expression of genes related to coping with oxidative stress conditions: particularly thioredoxin reductase, glutathione peroxidase and superoxide dismutase were among the most highly transcribed genes. A role of rumen wall microbes in oxygen scavenging has been proposed by Cheng et al. (1979). An exposure of epithelial microbial communities to oxygen diffusing from tissues has been described for non-ruminant mammals (Espey, 2013) and might also occur in the rumen when ruminants swallow air. Sampling location and rumen motility might also possibly affect rumen wall exposure to oxygen. Being able to cope with reactive oxygen might be thus advantageous for epimural bacteria, as also high-grain diets can influence the redox potential in the rumen, which affects microbial communities (Friedman et al., 2017). Furthermore, also a decrease in pH as a result of high grain diets, as seen in SARA, can also lead to an increase in redox potential (Friedman et al., 2017). Our metatranscriptome sequencing data thus suggest that the rumen wall is not a completely anoxic ecosystem and that some epimural microorganisms might be facultative anaerobes as suggested in earlier studies (Pennington, 1954; Cheng et al., 1979; Wallace et al., 1979). As there were no significant differences in the expression of genes involved in oxidative stress response between baseline and SARA, we assume that the high expression of these genes might be a physiological response and not a response to SARA. To the best of our knowledge, the transcription of oxidative stress response genes has not been detected in the rumen content using a metatranscriptome approach. Here, we also provide *in situ* evidence for cellulose and cellobiose degradation by rumen wall bacteria. Enzymatic hydrolysis of cellulose by microbes is a key step in the degradation of fiber-rich feed in the rumen of cattle. *Fibrobacter succinogenes*, *Ruminococcus albus*, and *R. flavefaciens* are major cellulolytic rumen bacteria, primarily responsible for biodegradation of plant material. Even if they are primarily associated with the solid phase of rumen contents (Henderson et al., 2015), the genera *Fibrobacter* and *Ruminococcus* were also shown to be abundant at the rumen wall (Petri et al., 2013). Evidence for active cellulose degradation has already been found in metatranscriptome approaches with rumen content samples (Kamke et al., 2016; Li and Guan, 2017) which reflects the higher importance of rumen content microorganisms (particularly of the particle-associated) for cellulose degradation compared to the rumen wall microbes.

In addition to cellulose degradation, rumen wall bacteria might also be involved in breakdown of starch indicated by the expression of glycogen phosphorylase and of alpha amylase. The high expression levels of glycogen phosphorylase and (to a lesser degree) of alpha amylase suggests that starch degradation might be an important metabolic process in the epimural bacteria. It should be noted that glycogen phosphorylase might also be involved in glycogen degradation; however, based on the high energy content in both diets, we believe that degradation of glycogen is unlikely to occur. On a functional level, high levels of transcription of carbohydrate metabolism genes have also been detected in the rumen content (Kamke et al., 2016; Li and Guan, 2017).

In general, amino acid metabolism was highly expressed in rumen wall bacteria, suggesting an active role of many rumen wall bacteria in degradation of proteins, which is in line with a previous hypothesis by Mao et al. (2015). Ammonia and urea are key metabolites in the rumen which are exchanged across the rumen wall (Abdoun et al., 2006; Reynolds and Kristensen, 2008). The ruminants recycle large amounts of urea into the rumen. Ammonia is produced by degradation of urea or dietary amino acids or other non-protein nitrogen sources of the diet, being then absorbed across the rumen wall or used by microbes for microbial protein synthesis (Abdoun et al., 2006). Already a few decades ago, several studies have shown that bacteria on the rumen wall have urease activity, although the identity of these microbes remained largely unknown (Abdel Rahman, 1966; Cheng et al., 1979; Cheng and Wallace, 1979; Wallace et al., 1979). Recently, a DNA-based amplicon sequencing study provided further evidence for ureolytic potential of rumen wall microbes (Jin et al., 2017) revealing members of the *Proteobacteria* and *Firmicutes* to have ureolytic potential. Their study also showed that bacteria and archaea of the liquid and particle-associated fractions of the rumen content possess ureolytic activity. However, the rumen wall and rumen content harbor distinct ureolytic bacteria and archaea (Jin et al., 2017). Here, we provide first transcriptome-based evidence for high urease activity of rumen wall microbes *in situ*, suggesting that urease activity is a central function of rumen wall microbial communities. In line with the study by Jin et al. (2017), we also found members of the *Firmicutes* and *Proteobacteria* and in addition also *Actinobacteria* and *Bacteroidetes* to express urease genes. As a result of their urease activity the epimural bacteria thus may influence the rumen ecosystem by affecting urea exchange (Abdoun et al., 2006) across the rumen wall more efficiently than thought, thereby playing an important role in the nitrogen cycle. Similarly, the high expression level of other key enzymes in nitrogen metabolism such as glutamate dehydrogenase, glutamine synthase and glutamate synthase underpins the importance of rumen wall bacteria in nitrogen metabolism. Furthermore, the detection of nitrogenase gene expression at the rumen wall – although overall at relatively low level – adds to the importance of rumen wall bacteria in many different areas of nitrogen metabolism. However, the importance of the rumen content microorganisms in nitrogen metabolism should also be acknowledged and, similar to our results, a high transcription of genes involved in glutamate metabolism in the rumen content has been described recently by Kamke et al. (2016), Li and Guan (2017). Li and Guan (2017) suggested that differences in nitrogen metabolism in the rumen content may be correlated with feed efficiency. Nitrogen fixation has been shown to be present by bacteria in the rumen content (Granhall and Ciszuk, 1971; Hobson et al., 1973; Li Pun and Satter, 1975) and in fecal samples of humans (Igai et al., 2016) although at low levels. To the best of our knowledge, the results from our study describe nitrogen fixation in epimural bacteria at the rumen wall of cattle for the first time.

Putative sulfate-reducing bacteria have been found on the rumen wall in a number of recent 16S rRNA gene-based studies (Petri et al., 2013; Mao et al., 2015; De Mulder et al., 2016; Wetzels et al., 2017). Here, we provide evidence for dissimilatory sulfate reduction activity by rumen wall bacteria. Whether the production of sulfide by sulfate reducers occurs at a sufficiently high level to have a possible negative impact on the rumen epithelium is currently unclear. We assume that sulfate reduction activity in the rumen wall has a negative effect on the rumen epithelium due to the toxicity of H_2_S (Drewnoski et al., 2014).

Enrichment analysis revealed only two statistically significantly differently expressed features on KO level 2 which were expressed at lower level during SARA conditions: nitrogen metabolism and oxidative stress response. On KO level function, we found only nine features to be statistically significantly differentially expressed between baseline and SARA conditions: only three features were higher expressed under SARA conditions. Furthermore, with the exception of the *N*-acetylneuraminate synthase, the expression level of the statistically significantly differently expressed genes was generally low. It was somewhat surprising that in spite of highly different diets and environmental conditions in the rumen such as a decreased pH during SARA conditions (Wetzels et al., 2017), our metatranscriptome sequencing results revealed only limited statistically significant changes in the gene expression pattern, as well as in community composition, of the epimural communities. This might be explained by the presence of functional guilds in the epimural bacteria: taxonomically different bacteria could fulfill the same metabolic function on the rumen wall. It has been shown that the epimural bacterial community is less sensitive to changes in environmental conditions than the microbial communities in the rumen content (Mao et al., 2015; Liu et al., 2016). This might also – at least to some degree – explain the lack of significant differences in gene expression patterns of the rumen wall microbial communities between baseline and SARA conditions. The response to SARA has been shown to be highly variable between animals (Humer et al., 2015). We can thus not exclude that our sample size of three animals might have been too low to determine an effect. Although we used only a small number of animals and due to our stringent quality control, we obtained only a comparatively low number of unique high-quality reads for our analyses. Based on our study design using a cross-over design, it should be highlighted that a control group (kept on the baseline diet) was not included, this might be helpful to reduce biases of possible time effects. Our results nevertheless reveal first insights into the gene expression of rumen wall microbiota. Future studies including more animals and higher sequencing depth will be needed to obtain a deeper understanding of rumen wall microbiota gene expression.

## Conclusion

Our study provides the first metatranscriptome sequencing of the rumen wall microbial communities. Although we could detect only limited statistically significant differences in gene expression of the epimural microbial communities between forage-only feeding and SARA conditions, we provide the first insights into the functional potential of rumen wall microbial communities *in situ*. Our results indicate that urease activity, oxygen scavenging, degradation of starch and amino acids are important functions of the rumen wall microbial communities. Furthermore, we provide evidence for nitrogen fixation and sulfate reduction by bacterial communities of the rumen wall and show the presence of archaea and fungi on the rumen wall.

## Author Contributions

EM and SW performed the experiments. SW, EM, and SS-E analyzed and interpreted the data. EM, SW, MW, QZ, and SS-E drafted and wrote the paper. SS-E and EM designed the study. All authors revised and approved the final manuscript.

## Conflict of Interest Statement

The authors declare that the research was conducted in the absence of any commercial or financial relationships that could be construed as a potential conflict of interest.

## Footnotes

1 www.vbcf.ac.at

2 http://metagenomics.anl.gov

## Abbreviations

KEGG: Kyoto Encyclopedia for Genes and Genomes
KO: KEGG orthology
SARA: subacute rumen acidosis
VFA: volatile fatty acids
