# Supplementary material for: Metatranscriptome Sequencing Reveals Insights into the Gene Expression and Functional Potential of Rumen Wall Bacteria
Source: Front Microbiol. 2018 Jan 23;9:43. doi: 10.3389/fmicb.2018.00043 (PMC5787071; doi:10.3389/fmicb.2018.00043)
Supplement: Supplementary file 1 [file Table_1.PDF]

Table S1. Read statistics of metatranscriptome samples (Baseline 1-3: Baseline replicates, SARA1-3: SARA replicates).

	Baseline 1 (no. of sequences)	Baseline 2 (no. of sequences)	Baseline 3 (no. of sequences)	SARA 1 (no. of sequences)	SARA 2 (no. of sequences)	SARA 3 (no. of sequences)
Raw read count	134,193,087	118,975,730	64,515,823	79,017,259	81,754,982	81,945,533
Quality controlled and duplicates removed (% of raw reads)	69,834,556 (52.04%)	62,764,018 (52.75%)	33,683,994 (52.21%)	42,224,985 (53.44%)	36,978,274 (45.23%)	46,178,415 (56.35%)
Sequencing artifacts removed (% of raw reads)	37,688,204 (28.09%)	33,565,392 (28.21%)	19,356,613 (30.00%)	23,243,655 (29.42%)	20,227,860 (24.74%)	25,475,780 (31.09%)
Average read length [bp]	177	179	205	180	191	180
Host reads removed (% of raw reads)	7,983,633 (5.94%)	7,886,856 (6.62%)	2,808,666 (4.35%)	4,579,783 (5.80%)	5,631,112 (6.89%)	6,600,499 (8.05%)
Predicted features (% of host reads removed)	7,869,067 (98.56%)	7,865,123 (99.72%)	2,808,666 (100.00%)	4,571,794 (99.83%)	5,631,112 (100.00%)	6,474,569 (98.09%)
Ribosomal RNA genes (% of predicted features)	263,676 (3.35%)	341,179 (4.34%)	217,523 (7.74%)	220,407 (4.82%)	287,933 (5.11%)	150,754 (2.33%)
Unknown features (% of host reads removed)	114,566 (1.44%)	21,733 (0.28%)	0 (0.00%)	7,989 (0.17%)	0 (0.00%)	125,930 (1.91%)
KEGG hits all domains	578,8409	429,6761	258,2623	344,2665	313,5455	458,6044
KEGG hits Eukaryota (% KEGG hits all domains)	4,856,772 (83.91%)	3,669,166 (85.39%)	2,440,012 (94.48%)	2,964,996 (86.13%)	2,323,951 (74.12%)	3,769,777 (82.20%)
KEGG hits Bacteria (% KEGG hits all domains)	880,047 (15.20%)	581,429 (13.53%)	133,610 (5.17%)	462,953 (13.45%)	777,767 (24.81%)	788,561 (17.19%)
KEGG hits Archaea (% KEGG hits all domains)	51,590 (0.89%)	46,166 (1.07%)	9,001 (0.35%)	14,716 (0.43%)	33,737 (1.08%)	27,706 (0.60%)
