# Supplementary material for: Metatranscriptome Sequencing Reveals Insights into the Gene Expression and Functional Potential of Rumen Wall Bacteria
Source: Front Microbiol. 2018 Jan 23;9:43. doi: 10.3389/fmicb.2018.00043 (PMC5787071; doi:10.3389/fmicb.2018.00043)
Supplement: Supplementary file 2 [file Table_2.PDF]

Table S2. Taxonomic distribution of bacterial reads on genus level. Read count values are given as transformed log_2 values of normalized counts per phylotype. Fold changes between baseline and SARA samples are listed. Statistically significant differences between baseline and SARA samples were indicated with $p<0.05$ and $q<0.05$. Duplicate reads were removed during quality control.

Phylum	Genus	Baseline 1	Baseline 2	Baseline 3	SARA 1	SARA 2	SARA 3	Fold change	p value	q value
Cyanobacteria	*Acaryochloris*	263	313	214	280	235	261	0.98	0.76	1.00
Proteobacteria	*Acetobacter*	73	57	43	92	45	50	1.09	0.72	1.00
Firmicutes	*Acetohalobium*	207	264	261	239	219	269	0.99	0.92	1.00
Tenericutes	*Acholeplasma*	719	668	865	790	766	552	0.94	0.90	1.00
Proteobacteria	*Achromobacter*	185	167	242	96	176	114	0.65	0.34	1.00
Firmicutes	*Acidaminococcus*	1606	1422	1577	2421	1504	2464	1.39	0.33	1.00
Actinobacteria	*Acidimicrobium*	17	28	14	22	14	19	0.92	0.67	1.00
Proteobacteria	*Acidiphilium*	373	425	252	289	330	480	1.05	0.97	1.00
Proteobacteria	*Acidithiobacillus*	265	134	295	96	280	184	0.81	0.87	1.00
Acidobacteria	*Acidobacterium*	92	215	38	91	94	127	0.90	0.64	1.00
Actinobacteria	*Acidothermus*	88	87	105	86	102	120	1.10	0.61	1.00
Proteobacteria	*Acidovorax*	3478	1900	2542	965	2314	818	0.52	0.07	0.85
Proteobacteria	*Acinetobacter*	1118	840	1587	541	1339	657	0.72	0.68	1.00
Proteobacteria	*Actinobacillus*	1175	816	1796	755	1968	575	0.87	0.89	1.00
Actinobacteria	*Actinosynnema*	87	58	57	87	87	56	1.14	0.97	1.00
Proteobacteria	*Aeromonas*	534	430	746	671	1285	709	1.56	0.07	0.87
Proteobacteria	*Aggregatibacter*	343	399	684	341	737	215	0.91	0.78	1.00
Proteobacteria	*Agrobacterium*	181	333	295	281	216	194	0.85	0.68	1.00
Verrucomicrobia	*Akkermansia*	569	589	409	785	481	723	1.27	0.70	1.00
Proteobacteria	*Alcanivorax*	166	182	466	282	538	395	1.49	0.05	0.77
Proteobacteria	*Alicycliphilus*	882	367	594	249	538	151	0.51	0.11	1.00
Firmicutes	*Alicyclobacillus*	375	271	314	372	272	328	1.01	0.87	1.00
Proteobacteria	*Aliivibrio*	244	238	190	170	361	233	1.14	0.69	1.00
Proteobacteria	*Alkalilimnicola*	282	251	323	204	439	205	0.99	0.79	1.00
Firmicutes	*Alkaliphilus*	4561	4500	5226	5420	3984	4748	0.99	1.00	1.00
Proteobacteria	*Allochromatium*	218	219	133	193	236	228	1.15	0.87	1.00
Proteobacteria	*Alteromonas*	119	115	71	118	266	162	1.79	0.14	1.00

Synergistetes	*Aminobacterium*	1234	1111	390	1658	873	1198	1.36	0.93	1.00
Firmicutes	*Ammonifex*	277	278	214	296	224	303	1.07	0.97	1.00
Actinobacteria	*Amycolatopsis*	84	90	57	59	62	57	0.77	0.30	1.00
Cyanobacteria	*Anabaena*	379	509	328	532	468	472	1.21	0.69	1.00
Firmicutes	*Anaerococcus*	705	838	936	932	652	726	0.93	0.87	1.00
Chloroflexi	*Anaerolinea*	629	443	361	895	495	485	1.31	0.83	1.00
Proteobacteria	*Anaeromyxobacter*	1301	1490	1682	1600	1407	2081	1.14	0.52	1.00
Proteobacteria	*Anaplasma*	58	114	62	103	74	76	1.08	1.00	1.00
Firmicutes	*Anoxybacillus*	360	369	437	326	380	331	0.89	0.85	1.00
Aquificae	*Aquifex*	343	440	266	251	239	291	0.75	0.24	1.00
Actinobacteria	*Arcanobacterium*	93	99	119	80	87	121	0.92	0.98	1.00
Proteobacteria	*Arcobacter*	775	968	1478	1120	792	665	0.80	0.71	1.00
Proteobacteria	*Aromatoleum*	1307	913	2912	779	1162	697	0.51	0.50	1.00
Actinobacteria	*Arthrobacter*	432	243	751	331	285	486	0.77	0.95	1.00
Proteobacteria	*Asticcacaulis*	275	221	409	867	127	128	1.24	0.97	1.00
Actinobacteria	*Atopobium*	1539	1250	2033	1851	1321	1703	1.01	0.81	1.00
Proteobacteria	*Azoarcus*	2216	1394	4504	1327	1811	1390	0.56	0.54	1.00
Proteobacteria	*Azorhizobium*	81	172	67	103	58	80	0.76	0.31	1.00
Proteobacteria	*Azospirillum*	658	1042	641	895	492	552	0.83	0.32	1.00
Proteobacteria	*Azotobacter*	246	194	261	164	208	113	0.69	0.28	1.00
Firmicutes	*Bacillus*	29570	20510	38108	21086	10855	15960	0.54	0.05	0.77
Bacteroidetes	*Bacteroides*	12746	10968	13545	29578	29484	35412	2.54	0.00	0.12
Proteobacteria	*Bartonella*	366	1119	945	597	504	627	0.71	0.55	1.00
Proteobacteria	*Basfia*	273	342	366	314	748	259	1.35	0.38	1.00
Proteobacteria	*Bdellovibrio*	1227	981	385	530	428	1267	0.86	0.53	1.00
Proteobacteria	*Beijerinckia*	77	57	38	129	35	50	1.24	0.85	1.00
Actinobacteria	*Beutenbergia*	73	241	52	40	42	82	0.45	0.25	1.00
Actinobacteria	*Bifidobacterium*	1479	1354	1340	1317	1298	1358	0.95	0.81	1.00
Bacteroidetes	*Blattabacterium*	105	97	86	115	157	133	1.41	0.33	1.00
Proteobacteria	*Bordetella*	976	710	993	495	1037	544	0.78	0.55	1.00
Spirochaetes	*Borrelia*	3038	2185	2081	2228	1488	2039	0.79	0.25	1.00

Actinobacteria	*Brachybacterium*	53	62	86	50	56	67	0.86	1.00	1.00
Spirochaetes	*Brachyspira*	3923	4404	4038	4297	3778	3921	0.97	0.87	1.00
Proteobacteria	*Bradyrhizobium*	688	559	622	648	415	515	0.84	0.45	1.00
Firmicutes	*Brevibacillus*	585	350	347	527	335	441	1.02	0.67	1.00
Proteobacteria	*Brevundimonas*	51	46	19	53	74	36	1.41	0.68	1.00
Proteobacteria	*Brucella*	159	290	157	273	187	172	1.04	0.85	1.00
Proteobacteria	*Buchnera*	88	150	86	93	160	97	1.08	0.82	1.00
Proteobacteria	*Burkholderia*	2580	2374	3663	1857	2858	1650	0.74	0.56	1.00
Firmicutes	*Butyrivibrio*	17122	19071	32730	20239	15118	15407	0.74	0.59	1.00
Firmicutes	*Caldicellulosiruptor*	5536	5119	6091	5517	3881	5413	0.88	0.69	1.00
Deferribacteres	*Calditerrivibrio*	459	349	394	719	410	538	1.39	0.44	1.00
Proteobacteria	*Campylobacter*	55998	42916	112509	68317	38106	31867	0.65	0.54	1.00
Bacteroidetes	*Candidatus* Amoebophilus	384	321	181	601	579	708	2.13	0.05	0.77
Proteobacteria	*Candidatus* Blochmannia	4	7	5	2	26	4	2.07	0.47	1.00
Firmicutes	*Candidatus* Desulforudis	288	336	285	435	278	353	1.17	0.75	1.00
Proteobacteria	*Candidatus* Hodgkinia	1	0	0	0	0	3	4.74	0.78	1.00
Acidobacteria	*Candidatus* Koribacter	725	1032	846	681	547	718	0.75	0.34	1.00
Proteobacteria	*Candidatus* Liberibacter	15	17	10	23	48	25	2.37	0.06	0.79
Proteobacteria	*Candidatus* Pelagibacter	94	235	233	213	108	145	0.83	0.76	1.00
Tenericutes	*Candidatus* Phytoplasma	691	978	371	359	315	643	0.65	0.17	1.00
Proteobacteria	*Candidatus* Puniceispirillum	252	130	48	92	64	359	1.20	0.97	1.00
Proteobacteria	*Candidatus* Riesia	7	8	5	15	7	5	1.36	0.96	1.00
Acidobacteria	*Candidatus* Solibacter	1550	1735	2556	1891	1386	1549	0.83	0.76	1.00
Bacteroidetes	*Candidatus* Sulcia	66	85	71	107	113	125	1.55	0.18	1.00
Proteobacteria	*Candidatus* Zinderia	1	1	0	1	1	0	1.93	1.00	1.00
Bacteroidetes	*Capnocytophaga*	892	664	1036	1458	1718	2173	2.06	0.01	0.23
Firmicutes	*Carboxydothermus*	1197	1271	1074	1420	1110	1450	1.12	0.82	1.00
Actinobacteria	*Catenulispora*	294	331	124	240	124	387	1.00	0.70	1.00
Proteobacteria	*Caulobacter*	357	790	428	862	394	450	1.08	0.91	1.00
Actinobacteria	*Cellulomonas*	126	117	86	36	116	84	0.72	0.33	1.00

Bacteroidetes	*Cellulophaga*	164	166	209	297	721	742	3.27	0.00	0.12
Proteobacteria	*Cellvibrio*	237	255	204	213	362	172	1.07	0.86	1.00
Proteobacteria	*Chelativorans*	262	553	371	239	142	144	0.44	0.01	0.28
Bacteroidetes	*Chitinophaga*	1024	656	1007	1279	2057	2448	2.15	0.01	0.20
Chlamydiae	*Chlamydia*	80	135	67	105	92	89	1.02	0.82	1.00
Chlamydiae	*Chlamydophila*	219	429	181	311	322	327	1.16	0.82	1.00
Chlorobi	*Chlorobaculum*	450	421	266	416	410	513	1.18	0.85	1.00
Chlorobi	*Chlorobium*	1411	1599	1045	1610	1602	1601	1.19	0.76	1.00
Chloroflexi	*Chloroflexus*	1214	1435	1078	1658	733	878	0.88	0.36	1.00
Chlorobi	*Chloroherpeton*	329	581	613	326	682	449	0.96	0.66	1.00
Proteobacteria	*Chromobacterium*	3998	2011	4704	996	6251	1696	0.83	0.98	1.00
Proteobacteria	*Chromohalobacter*	237	263	309	137	227	177	0.67	0.34	1.00
Proteobacteria	*Citrobacter*	150	264	171	174	274	292	1.26	0.38	1.00
Actinobacteria	*Clavibacter*	64	96	90	86	115	90	1.16	0.50	1.00
Firmicutes	*Clostridium*	36545	36122	46565	42017	33258	41563	0.98	0.92	1.00
Proteobacteria	*Colwellia*	210	213	185	215	248	158	1.02	0.94	1.00
Proteobacteria	*Comamonas*	680	346	461	233	483	239	0.64	0.17	1.00
Actinobacteria	*Conexibacter*	89	84	133	139	120	183	1.44	0.13	1.00
Firmicutes	*Coprothermobacter*	421	615	518	386	359	288	0.67	0.18	1.00
Verrucomicrobia	*Coraliomargarita*	369	438	556	351	308	705	1.00	0.67	1.00
Actinobacteria	*Corynebacterium*	897	744	1373	809	834	785	0.81	0.79	1.00
Proteobacteria	*Coxiella*	360	177	565	464	276	319	0.96	0.83	1.00
Bacteroidetes	*Croceibacter*	444	344	399	592	1041	1397	2.55	0.01	0.23
Proteobacteria	*Cronobacter*	324	576	656	298	299	198	0.51	0.07	0.86
Actinobacteria	*Cryptobacterium*	979	1009	1174	1064	736	597	0.76	0.33	1.00
Proteobacteria	*Cupriavidus*	1583	1267	1881	635	1478	799	0.62	0.23	1.00
Cyanobacteria	*Cyanothece*	840	1211	803	738	739	997	0.87	0.60	1.00
Bacteroidetes	*Cytophaga*	1109	773	1501	1800	2431	2924	2.11	0.00	0.12
Proteobacteria	*Dechloromonas*	1079	605	1454	457	986	534	0.63	0.46	1.00
Deferribacteres	*Deferribacter*	658	664	1288	1165	1010	948	1.20	0.24	1.00
Chloroflexi	*Dehalococcoides*	294	346	252	337	313	376	1.15	0.77	1.00

Chloroflexi	*Dehalogenimonas*	76	56	76	77	84	70	1.11	0.74	1.00
Deinococcus-Thermus	*Deinococcus*	414	491	679	922	514	535	1.24	0.39	1.00
Proteobacteria	*Delftia*	1337	572	884	470	887	370	0.62	0.19	1.00
Deferribacteres	*Denitrovibrio*	372	915	337	527	326	733	0.98	0.81	1.00
Proteobacteria	*Desulfarculus*	560	510	475	472	248	509	0.80	0.33	1.00
Proteobacteria	*Desulfatibacillum*	653	606	342	625	586	765	1.23	0.79	1.00
Firmicutes	*Desulfitobacterium*	2219	2111	2338	2451	1826	2261	0.98	0.92	1.00
Proteobacteria	*Desulfobacterium*	512	476	242	470	495	543	1.22	0.85	1.00
Proteobacteria	*Desulfobulbus*	7195	4913	4395	8169	7570	6574	1.35	0.56	1.00
Proteobacteria	*Desulfococcus*	625	400	499	590	491	570	1.08	0.93	1.00
Proteobacteria	*Desulfohalobium*	251	267	138	264	247	258	1.17	0.95	1.00
Proteobacteria	*Desulfomicrobium*	300	236	247	427	303	375	1.41	0.39	1.00
Proteobacteria	*Desulfotalea*	3853	2591	2285	4225	3824	3673	1.34	0.58	1.00
Firmicutes	*Desulfotomaculum*	1755	1696	1905	2239	1604	2034	1.10	0.78	1.00
Proteobacteria	*Desulfovibrio*	5421	4217	3045	7932	4389	5858	1.43	0.56	1.00
Chrysiogenetes	*Desulfurispirillum*	213	271	128	239	239	173	1.06	0.81	1.00
Proteobacteria	*Desulfurivibrio*	2176	1557	1378	2570	2294	2129	1.37	0.52	1.00
Proteobacteria	*Dichelobacter*	632	819	675	674	4712	446	2.74	0.41	1.00
Proteobacteria	*Dickeya*	606	428	879	250	539	155	0.49	0.18	1.00
Dictyoglomi	*Dictyoglomus*	979	1971	1326	1304	959	1466	0.87	0.67	1.00
Proteobacteria	*Dinoroseobacter*	81	42	81	70	61	60	0.93	0.88	1.00
Bacteroidetes	*Dyadobacter*	600	549	551	1004	1436	1717	2.45	0.00	0.12
Proteobacteria	*Edwardsiella*	153	177	157	193	274	275	1.52	0.15	1.00
Actinobacteria	*Eggerthella*	1282	1122	1402	1199	994	1421	0.95	0.94	1.00
Proteobacteria	*Ehrlichia*	74	103	33	76	72	68	1.03	0.68	1.00
Elusimicrobia	*Elusimicrobium*	483	416	385	608	623	763	1.55	0.19	1.00
Proteobacteria	*Enterobacter*	170	216	223	152	266	204	1.02	0.70	1.00
Firmicutes	*Enterococcus*	563	959	689	823	504	604	0.87	0.55	1.00
Proteobacteria	*Erwinia*	86	185	257	132	209	107	0.85	0.80	1.00
Proteobacteria	*Erythrobacter*	86	213	114	151	136	78	0.88	0.64	1.00

Proteobacteria	*Escherichia*	1168	1439	1254	1534	2173	1344	1.31	0.35	1.00
Firmicutes	*Ethanoligenens*	1604	1548	1820	2042	1813	2307	1.24	0.42	1.00
Firmicutes	*Eubacterium*	14725	13588	21589	17516	13340	17183	0.96	0.85	1.00
Firmicutes	*Exiguobacterium*	554	457	599	639	504	533	1.04	0.89	1.00
Proteobacteria	*Ferrimonas*	102	133	119	109	260	98	1.32	0.42	1.00
Thermotogae	*Fervidobacterium*	1475	1767	1487	1040	621	1010	0.56	0.04	0.67
Fibrobacteres	*Fibrobacter*	5440	7429	8010	8618	5103	5944	0.94	0.88	1.00
Firmicutes	*Finegoldia*	692	587	808	1011	545	755	1.11	0.77	1.00
Bacteroidetes	*Flavobacterium*	2485	1653	3644	3063	4057	5133	1.57	0.04	0.62
Proteobacteria	*Francisella*	414	940	713	348	775	470	0.77	0.71	1.00
Actinobacteria	*Frankia*	217	241	166	202	224	226	1.04	0.96	1.00
Fusobacteria	*Fusobacterium*	1362	1853	1748	1398	1216	1218	0.77	0.43	1.00
Proteobacteria	*Gallionella*	136	114	214	139	210	206	1.19	0.25	1.00
Actinobacteria	*Gardnerella*	185	169	100	147	223	235	1.33	0.52	1.00
Gemmatimonadetes	*Gemmatimonas*	164	446	242	318	264	299	1.03	0.94	1.00
Firmicutes	*Geobacillus*	3435	3168	2475	2937	2150	2817	0.87	0.43	1.00
Proteobacteria	*Geobacter*	3605	3673	3273	4163	3372	3853	1.08	0.92	1.00
Actinobacteria	*Geodermatophilus*	84	54	71	63	40	39	0.67	0.17	1.00
Cyanobacteria	*Gloeobacter*	177	431	238	410	167	415	1.17	0.79	1.00
Proteobacteria	*Gluconacetobacter*	229	608	380	446	148	131	0.60	0.20	1.00
Proteobacteria	*Gluconobacter*	97	79	86	132	81	89	1.16	0.87	1.00
Actinobacteria	*Gordonia*	52	62	81	58	80	62	1.03	0.65	1.00
Bacteroidetes	*Gramella*	940	857	993	1516	2255	2789	2.35	0.00	0.12
Proteobacteria	*Granulibacter*	116	175	480	662	173	210	1.36	0.46	1.00
Proteobacteria	*Haemophilus*	890	858	1273	744	1797	935	1.15	0.39	1.00
Proteobacteria	*Hahella*	399	284	542	337	476	359	0.96	0.77	1.00
Firmicutes	*Halanaerobium*	603	465	290	294	318	489	0.81	0.29	1.00
Proteobacteria	*Haliangium*	275	549	185	303	229	669	1.19	0.86	1.00
Proteobacteria	*Halomonas*	124	162	90	78	133	85	0.79	0.38	1.00
Proteobacteria	*Halorhodospira*	158	184	152	125	184	136	0.90	0.77	1.00
Firmicutes	*Halothermothrix*	672	688	622	562	531	751	0.93	0.79	1.00

Proteobacteria	*Halothiobacillus*	320	184	105	80	236	84	0.66	0.29	1.00
Proteobacteria	*Helicobacter*	1590	1832	2366	1939	1331	1130	0.76	0.43	1.00
Firmicutes	*Heliobacterium*	1027	577	1012	1079	790	801	1.02	0.99	1.00
Proteobacteria	*Herbaspirillum*	312	189	337	128	462	128	0.86	0.96	1.00
Proteobacteria	*Herminiimonas*	309	244	489	174	382	159	0.69	0.65	1.00
Chloroflexi	*Herpetosiphon*	187	178	119	158	175	187	1.08	0.97	1.00
Proteobacteria	*Hirschia*	54	58	38	88	42	47	1.18	0.97	1.00
Proteobacteria	*Histophilus*	120	144	195	126	309	92	1.15	0.52	1.00
Aquificae	*Hydrogenobacter*	96	71	43	52	77	188	1.51	0.56	1.00
Aquificae	*Hydrogenobaculum*	86	79	67	119	97	96	1.34	0.54	1.00
Proteobacteria	*Hyphomicrobium*	127	146	57	86	53	102	0.73	0.16	1.00
Proteobacteria	*Hyphomonas*	95	269	442	634	386	166	1.47	0.29	1.00
Proteobacteria	*Idiomarina*	137	130	143	190	287	153	1.54	0.16	1.00
Fusobacteria	*Ilyobacter*	682	569	774	782	607	702	1.03	0.87	1.00
Actinobacteria	*Intrasporangium*	32	35	29	25	27	29	0.84	0.63	1.00
Planctomycetes	*Isosphaera*	610	1072	371	248	421	432	0.54	0.12	1.00
Proteobacteria	*Jannaschia*	60	94	114	76	75	58	0.78	0.70	1.00
Actinobacteria	*Jonesia*	93	64	95	56	74	72	0.80	0.63	1.00
Proteobacteria	*Kangiella*	226	183	223	188	284	161	1.00	0.93	1.00
Proteobacteria	*Ketogulonicigenium*	23	27	14	52	35	46	2.06	0.14	1.00
Actinobacteria	*Kineococcus*	84	80	86	93	75	132	1.20	0.55	1.00
Proteobacteria	*Klebsiella*	262	185	261	180	296	192	0.94	0.99	1.00
Actinobacteria	*Kocuria*	96	82	461	113	85	121	0.50	0.88	1.00
Thermotogae	*Kosmotoga*	277	222	318	310	289	336	1.14	0.55	1.00
Actinobacteria	*Kribbella*	49	156	52	47	54	82	0.71	0.50	1.00
Actinobacteria	*Kytococcus*	32	27	33	26	43	101	1.83	0.23	1.00
Firmicutes	*Lactobacillus*	3398	3796	4228	3854	3171	3433	0.92	0.84	1.00
Firmicutes	*Lactococcus*	1253	1741	594	697	753	751	0.61	0.09	1.00
Proteobacteria	*Laribacter*	2118	975	2594	526	3158	789	0.79	0.96	1.00
Proteobacteria	*Lawsonia*	439	299	266	542	286	300	1.12	0.81	1.00
Bacteroidetes	*Leadbetterella*	359	289	342	646	922	1049	2.65	0.00	0.10

Proteobacteria	*Legionella*	702	469	1387	522	731	508	0.69	0.81	1.00
Actinobacteria	*Leifsonia*	52	39	48	48	59	51	1.14	0.71	1.00
Spirochaetes	*Leptospira*	622	643	504	1141	684	828	1.50	0.34	1.00
Proteobacteria	*Leptothrix*	689	445	599	200	558	213	0.56	0.11	1.00
Fusobacteria	*Leptotrichia*	451	1005	556	763	533	446	0.87	0.52	1.00
Firmicutes	*Leuconostoc*	339	743	209	313	285	346	0.73	0.35	1.00
Firmicutes	*Listeria*	908	1504	884	1068	973	965	0.91	0.63	1.00
Firmicutes	*Lysinibacillus*	320	279	380	313	303	406	1.04	0.73	1.00
Firmicutes	*Macrococcus*	259	233	138	224	172	207	0.96	0.53	1.00
Proteobacteria	*Magnetococcus*	774	591	1288	735	374	451	0.59	0.23	1.00
Proteobacteria	*Magnetospirillum*	2381	3670	2295	2460	1718	2585	0.81	0.38	1.00
Bacteroidetes	*Maribacter*	512	450	508	704	1047	1143	1.97	0.02	0.39
Proteobacteria	*Maricaulis*	96	71	71	119	145	118	1.60	0.21	1.00
Proteobacteria	*Marinobacter*	609	298	689	248	407	282	0.59	0.21	1.00
Proteobacteria	*Marinomonas*	291	265	432	269	607	257	1.15	0.44	1.00
Bacteroidetes	*Marivirga*	377	322	485	830	1370	1846	3.42	0.00	0.07
Deinococcus-Thermus	*Meiothermus*	620	726	755	524	471	580	0.75	0.41	1.00
Tenericutes	*Mesoplasma*	258	164	247	184	195	159	0.80	0.49	1.00
Proteobacteria	*Mesorhizobium*	472	1709	437	441	518	832	0.68	0.51	1.00
Verrucomicrobia	*Methylacidiphilum*	112	131	62	104	97	139	1.12	0.98	1.00
Proteobacteria	*Methylibium*	641	415	447	302	538	260	0.73	0.25	1.00
Proteobacteria	*Methylobacillus*	628	369	855	316	752	381	0.78	0.88	1.00
Proteobacteria	*Methylobacterium*	554	1678	2490	1099	1420	1451	0.84	0.69	1.00
Proteobacteria	*Methylocella*	90	71	124	93	58	66	0.76	0.51	1.00
Proteobacteria	*Methylococcus*	382	276	242	323	372	432	1.25	0.62	1.00
Proteobacteria	*Methylotenera*	366	250	527	155	449	200	0.70	0.73	1.00
Proteobacteria	*Methylovorus*	306	179	499	110	469	131	0.72	0.93	1.00
Actinobacteria	*Micrococcus*	138	49	166	99	82	99	0.79	0.72	1.00
Cyanobacteria	*Microcystis*	147	162	157	189	229	182	1.29	0.39	1.00
Actinobacteria	*Micromonospora*	44	90	67	31	49	35	0.57	0.17	1.00

Actinobacteria	*Mobiluncus*	83	60	57	78	104	107	1.44	0.35	1.00
Firmicutes	*Moorella*	1346	1394	1677	1475	1031	1254	0.85	0.64	1.00
Proteobacteria	*Moraxella*	145	133	223	108	260	99	0.93	0.78	1.00
Actinobacteria	*Mycobacterium*	521	671	637	553	559	594	0.93	0.91	1.00
Tenericutes	*Mycoplasma*	1659	1583	1511	1158	1393	1350	0.82	0.51	1.00
Proteobacteria	*Myxococcus*	494	600	276	411	405	567	1.01	0.74	1.00
Actinobacteria	*Nakamurella*	46	96	86	34	48	48	0.57	0.23	1.00
Firmicutes	*Natranaerobius*	504	491	542	470	417	467	0.88	0.70	1.00
Proteobacteria	*Nautilia*	398	272	760	441	261	251	0.67	0.57	1.00
Proteobacteria	*Neisseria*	21207	9295	24439	4596	29042	7308	0.75	0.87	1.00
Proteobacteria	*Neorickettsia*	29	25	10	23	20	30	1.17	0.93	1.00
Proteobacteria	*Nitratifractor*	120	206	261	220	115	92	0.73	0.48	1.00
Proteobacteria	*Nitratiruptor*	637	519	1131	806	532	513	0.81	0.81	1.00
Proteobacteria	*Nitrobacter*	182	185	323	194	178	210	0.84	1.00	1.00
Proteobacteria	*Nitrosococcus*	514	430	418	421	760	515	1.25	0.47	1.00
Proteobacteria	*Nitrosomonas*	510	384	570	324	661	314	0.89	0.93	1.00
Proteobacteria	*Nitrosospira*	276	361	409	240	417	222	0.84	0.86	1.00
Nitrospirae	*Nitrospira*	102	125	109	104	75	99	0.83	0.53	1.00
Actinobacteria	*Nocardia*	81	72	105	76	63	76	0.84	0.74	1.00
Actinobacteria	*Nocardioides*	145	171	114	129	106	215	1.05	1.00	1.00
Actinobacteria	*Nocardiopsis*	43	72	62	48	49	56	0.86	0.82	1.00
Cyanobacteria	*Nostoc*	159	129	105	102	181	166	1.14	0.76	1.00
Proteobacteria	*Novosphingobium*	167	237	299	468	287	177	1.33	0.41	1.00
Deinococcus-Thermus	*Oceanithermus*	44	62	24	85	44	74	1.55	0.49	1.00
Firmicutes	*Oceanobacillus*	550	495	822	583	487	505	0.84	0.82	1.00
Proteobacteria	*Ochrobactrum*	842	434	1216	356	682	696	0.70	0.72	1.00
Firmicutes	*Oenococcus*	190	180	119	255	249	155	1.35	0.61	1.00
Proteobacteria	*Oligotropha*	49	68	67	61	92	50	1.11	0.62	1.00
Actinobacteria	*Olsenella*	1031	803	955	1144	788	1204	1.12	0.77	1.00
Verrucomicrobia	*Opitutus*	554	522	466	438	382	899	1.11	0.75	1.00

Proteobacteria	*Orientia*	74	75	81	85	51	72	0.91	0.73	1.00
Firmicutes	*Paenibacillus*	1658	2572	1777	1593	1343	2071	0.83	0.53	1.00
Bacteroidetes	*Paludibacter*	1715	1477	2052	4045	3811	4463	2.35	0.00	0.12
Proteobacteria	*Pantoea*	294	361	147	222	260	133	0.77	0.21	1.00
Bacteroidetes	*Parabacteroides*	3612	3155	4157	8425	10387	11958	2.82	0.00	0.07
Proteobacteria	*Paracoccus*	147	119	233	119	162	147	0.86	0.93	1.00
Proteobacteria	*Parvibaculum*	176	140	418	199	260	462	1.25	0.22	1.00
Proteobacteria	*Parvularcula*	83	339	152	118	84	135	0.59	0.34	1.00
Proteobacteria	*Pasteurella*	275	198	352	278	712	198	1.44	0.37	1.00
Proteobacteria	*Pectobacterium*	327	266	347	239	435	188	0.92	0.93	1.00
Firmicutes	*Pediococcus*	300	335	323	272	462	314	1.10	0.65	1.00
Bacteroidetes	*Pedobacter*	1082	693	1026	1680	2858	3310	2.80	0.00	0.07
Proteobacteria	*Pelobacter*	1722	2092	1459	2006	2127	2336	1.23	0.59	1.00
Chlorobi	*Pelodictyon*	347	305	375	432	480	484	1.36	0.27	1.00
Firmicutes	*Pelotomaculum*	736	677	732	1218	700	925	1.32	0.46	1.00
Aquificae	*Persephonella*	155	254	209	175	268	154	0.97	0.94	1.00
Thermotogae	*Petrotoga*	412	359	285	334	406	426	1.10	0.90	1.00
Proteobacteria	*Phenylobacterium*	96	53	52	123	39	62	1.11	0.73	1.00
Proteobacteria	*Photobacterium*	201	283	299	208	333	229	0.98	0.76	1.00
Proteobacteria	*Photorhabdus*	277	381	271	308	528	282	1.20	0.55	1.00
Planctomycetes	*Pirellula*	315	560	114	757	674	409	1.86	0.33	1.00
Planctomycetes	*Planctomyces*	379	354	537	536	328	627	1.17	0.43	1.00
Proteobacteria	*Polaromonas*	1913	1082	1739	635	1515	547	0.57	0.15	1.00
Proteobacteria	*Polynucleobacter*	295	198	323	147	291	182	0.76	0.58	1.00
Bacteroidetes	*Porphyromonas*	2738	1749	2323	4703	5320	6952	2.49	0.00	0.12
Bacteroidetes	*Prevotella*	9978	7713	11170	38186	27316	38712	3.61	0.00	0.02
Cyanobacteria	*Prochlorococcus*	362	605	404	455	378	350	0.86	0.54	1.00
Actinobacteria	*Propionibacterium*	787	810	2233	1046	1385	978	0.89	0.60	1.00
Chlorobi	*Prosthecochloris*	121	112	76	137	128	152	1.35	0.55	1.00
Proteobacteria	*Proteus*	139	134	128	115	259	162	1.34	0.30	1.00
Proteobacteria	*Pseudoalteromonas*	401	379	375	378	685	572	1.41	0.21	1.00

Proteobacteria	*Pseudomonas*	2394	2160	2960	1641	2965	1594	0.83	0.76	1.00
Proteobacteria	*Pseudoxanthomonas*	74	71	90	58	83	88	0.97	0.85	1.00
Proteobacteria	*Psychrobacter*	942	699	1421	727	1236	528	0.81	0.91	1.00
Proteobacteria	*Psychromonas*	194	282	176	302	301	225	1.27	0.58	1.00
Proteobacteria	*Ralstonia*	1243	803	1544	711	1123	680	0.70	0.43	1.00
Actinobacteria	*Renibacterium*	21	28	43	42	32	19	1.01	0.76	1.00
Proteobacteria	*Rhizobium*	293	565	456	411	400	471	0.98	0.90	1.00
Proteobacteria	*Rhodobacter*	239	282	304	311	257	229	0.97	0.96	1.00
Actinobacteria	*Rhodococcus*	379	304	489	323	314	267	0.77	0.54	1.00
Proteobacteria	*Rhodomicrobium*	50	102	52	145	117	39	1.47	0.59	1.00
Planctomycetes	*Rhodopirellula*	512	724	280	1202	721	709	1.74	0.31	1.00
Proteobacteria	*Rhodopseudomonas*	1191	1385	1383	1197	1081	1003	0.83	0.55	1.00
Proteobacteria	*Rhodospirillum*	1508	1952	2841	2481	996	1159	0.74	0.46	1.00
Bacteroidetes	*Rhodothermus*	1053	1797	1482	867	975	1737	0.83	0.74	1.00
Proteobacteria	*Rickettsia*	1012	875	855	304	637	1030	0.72	0.37	1.00
Bacteroidetes	*Riemerella*	421	233	390	477	817	879	2.08	0.01	0.24
Bacteroidetes	*Robiginitalea*	449	330	361	627	932	966	2.22	0.01	0.24
Chloroflexi	*Roseiflexus*	1097	1497	641	1023	680	1026	0.84	0.29	1.00
Proteobacteria	*Roseobacter*	57	184	38	81	53	76	0.75	0.47	1.00
Actinobacteria	*Rothia*	58	38	233	124	120	110	1.08	0.32	1.00
Actinobacteria	*Rubrobacter*	197	206	133	204	139	175	0.97	0.61	1.00
Proteobacteria	*Ruegeria*	157	153	190	145	139	153	0.88	0.80	1.00
Firmicutes	*Ruminococcus*	4614	4107	4875	5623	5967	8854	1.50	0.13	1.00
Actinobacteria	*Saccharomonospora*	40	99	38	45	51	60	0.88	0.67	1.00
Proteobacteria	*Saccharophagus*	370	394	432	199	466	306	0.81	0.74	1.00
Actinobacteria	*Saccharopolyspora*	203	213	105	169	131	107	0.78	0.17	1.00
Bacteroidetes	*Salinibacter*	177	153	176	221	176	313	1.41	0.27	1.00
Actinobacteria	*Salinispora*	142	175	124	87	117	127	0.75	0.36	1.00
Proteobacteria	*Salmonella*	484	347	912	343	588	405	0.77	1.00	1.00
Actinobacteria	*Sanguibacter*	37	46	38	43	51	62	1.28	0.47	1.00
Fusobacteria	*Sebaldella*	729	1422	1383	608	640	551	0.51	0.06	0.79

Actinobacteria	*Segniliparus*	21	24	10	12	22	22	1.04	0.91	1.00
Proteobacteria	*Serratia*	159	142	133	132	195	173	1.15	0.67	1.00
Proteobacteria	*Shewanella*	1572	1611	1558	1561	2348	1731	1.19	0.51	1.00
Proteobacteria	*Shigella*	341	421	432	336	559	354	1.04	0.69	1.00
Proteobacteria	*Sideroxydans*	428	249	447	167	384	277	0.74	0.53	1.00
Proteobacteria	*Sinorhizobium*	441	634	736	900	969	615	1.37	0.18	1.00
Actinobacteria	*Slackia*	2161	2026	2480	2357	1938	2486	1.02	0.88	1.00
Proteobacteria	*Sodalis*	83	58	105	70	105	46	0.90	0.98	1.00
Proteobacteria	*Sorangium*	246	232	157	164	237	379	1.23	0.59	1.00
Chloroflexi	*Sphaerobacter*	181	155	176	238	177	208	1.22	0.60	1.00
Proteobacteria	*Sphingobium*	211	109	133	124	201	121	0.99	0.82	1.00
Proteobacteria	*Sphingomonas*	157	106	171	162	141	115	0.96	0.95	1.00
Proteobacteria	*Sphingopyxis*	148	279	95	448	398	161	1.93	0.31	1.00
Spirochaetes	*Spirochaeta*	7220	4516	5383	7001	4556	5671	1.01	0.76	1.00
Bacteroidetes	*Spirosoma*	638	687	584	908	1558	1789	2.23	0.01	0.21
Actinobacteria	*Stackebrandtia*	45	68	48	33	40	56	0.81	0.61	1.00
Firmicutes	*Staphylococcus*	1799	2306	3397	2136	1582	2009	0.76	0.66	1.00
Proteobacteria	*Starkeya*	284	137	105	101	55	79	0.45	0.03	0.57
Proteobacteria	*Stenotrophomonas*	348	478	532	237	291	192	0.53	0.08	0.88
Fusobacteria	*Streptobacillus*	328	396	489	363	258	321	0.78	0.54	1.00
Firmicutes	*Streptococcus*	3723	4052	4770	4047	4025	3950	0.96	0.96	1.00
Actinobacteria	*Streptomyces*	355	489	323	297	327	409	0.88	0.65	1.00
Actinobacteria	*Streptosporangium*	72	137	105	74	80	76	0.73	0.43	1.00
Proteobacteria	*Sulfuricurvum*	144	192	242	194	122	120	0.75	0.47	1.00
Aquificae	*Sulfurihydrogenibium*	506	498	276	314	518	560	1.09	0.97	1.00
Proteobacteria	*Sulfurimonas*	428	498	893	636	490	438	0.86	0.96	1.00
Proteobacteria	*Sulfurospirillum*	1397	1283	2071	1327	811	829	0.62	0.16	1.00
Proteobacteria	*Sulfurovum*	328	359	523	381	274	321	0.81	0.69	1.00
Firmicutes	*Symbiobacterium*	905	677	827	1221	725	780	1.13	0.88	1.00
Cyanobacteria	*Synechococcus*	913	1464	960	1366	1063	1183	1.08	0.92	1.00
Cyanobacteria	*Synechocystis*	216	206	171	222	239	255	1.21	0.65	1.00

Proteobacteria	*Syntrophobacter*	1354	1322	779	1317	767	1352	0.99	0.60	1.00
Firmicutes	*Syntrophomonas*	1315	1185	1235	1136	1109	1240	0.93	0.79	1.00
Firmicutes	*Syntrophothermus*	343	280	290	343	244	298	0.97	0.75	1.00
Proteobacteria	*Syntrophus*	616	621	755	589	597	733	0.96	0.93	1.00
Proteobacteria	*Taylorella*	246	204	314	120	404	125	0.85	1.00	1.00
Proteobacteria	*Teredinibacter*	164	168	152	120	224	119	0.96	0.94	1.00
Acidobacteria	*Terriglobus*	70	65	62	71	57	87	1.09	0.88	1.00
Proteobacteria	*Thauera*	1782	1291	4452	1082	1310	1216	0.48	0.51	1.00
Firmicutes	*Thermaerobacter*	181	140	423	269	196	251	0.96	0.57	1.00
Synergistetes	*Thermanaerovibrio*	1510	1361	808	2107	1244	1417	1.30	0.84	1.00
Firmicutes	*Thermincola*	653	696	466	765	526	970	1.25	0.67	1.00
Firmicutes	*Thermoanaerobacter*	1638	1647	1919	1849	1481	1766	0.98	1.00	1.00
Firmicutes	*Thermoanaerobacterium*	955	971	1554	1117	920	1151	0.92	0.89	1.00
Chloroflexi	*Thermobaculum*	157	178	100	159	133	141	1.00	0.68	1.00
Actinobacteria	*Thermobifida*	120	93	90	92	86	163	1.12	0.79	1.00
Actinobacteria	*Thermobispora*	81	84	29	56	53	72	0.94	0.48	1.00
Aquificae	*Thermocrinis*	85	80	166	74	111	94	0.84	0.84	1.00
Nitrospirae	*Thermodesulfovibrio*	246	230	608	250	223	269	0.68	0.85	1.00
Chloroflexi	*Thermomicrobium*	386	867	285	361	411	578	0.88	0.64	1.00
Actinobacteria	*Thermomonospora*	58	49	76	118	123	78	1.75	0.07	0.85
Firmicutes	*Thermosediminibacter*	602	614	603	863	641	682	1.20	0.64	1.00
Thermotogae	*Thermosipho*	2175	2573	1283	2595	1070	2211	0.97	0.52	1.00
Cyanobacteria	*Thermosynechococcus*	203	330	181	199	222	229	0.91	0.66	1.00
Thermotogae	*Thermotoga*	2128	3440	2029	2593	1355	1845	0.76	0.20	1.00
Aquificae	*Thermovibrio*	765	523	675	388	208	718	0.67	0.23	1.00
Deinococcus-Thermus	*Thermus*	302	390	285	335	319	332	1.01	0.93	1.00
Proteobacteria	*Thioalkalivibrio*	338	311	299	278	599	245	1.19	0.61	1.00
Proteobacteria	*Thiobacillus*	413	306	485	232	544	253	0.85	0.91	1.00
Proteobacteria	*Thiomicrospira*	174	140	128	135	214	112	1.04	0.99	1.00
Proteobacteria	*Thiomonas*	336	191	409	120	401	193	0.76	0.83	1.00

Proteobacteria	*Tolumonas*	149	190	257	352	694	294	2.25	0.03	0.57
Spirochaetes	*Treponema*	16858	9955	30972	32676	16972	19725	1.20	0.42	1.00
Cyanobacteria	*Trichodesmium*	168	137	90	117	200	161	1.21	0.75	1.00
Actinobacteria	*Tropheryma*	26	38	24	26	14	18	0.65	0.19	1.00
Deinococcus-Thermus	*Truepera*	105	95	62	91	66	77	0.89	0.44	1.00
Actinobacteria	*Tsukamurella*	13	44	43	37	39	34	1.09	0.49	1.00
Tenericutes	*Ureaplasma*	103	87	67	82	73	99	0.99	0.77	1.00
Proteobacteria	*Variovorax*	983	605	727	336	732	187	0.54	0.08	0.90
Firmicutes	*Veillonella*	639	543	793	860	673	879	1.22	0.41	1.00
Proteobacteria	*Verminephrobacter*	881	444	627	254	470	218	0.48	0.03	0.50
Proteobacteria	*Vibrio*	813	946	1506	768	1813	724	1.01	0.58	1.00
Chlamydiae	*Waddlia*	153	160	67	113	129	322	1.49	0.58	1.00
Proteobacteria	*Wigglesworthia*	61	78	14	36	45	32	0.74	0.26	1.00
Proteobacteria	*Wolbachia*	101	158	57	49	129	109	0.91	0.73	1.00
Proteobacteria	*Wolinella*	707	834	1644	1018	623	535	0.68	0.59	1.00
Proteobacteria	*Xanthobacter*	391	287	271	320	209	720	1.32	0.63	1.00
Proteobacteria	*Xanthomonas*	816	851	1140	526	1247	609	0.85	0.96	1.00
Proteobacteria	*Xenorhabdus*	110	78	124	87	150	70	0.99	0.85	1.00
Actinobacteria	*Xylanimonas*	410	115	119	115	365	98	0.90	0.69	1.00
Proteobacteria	*Xylella*	286	199	252	157	326	155	0.87	0.72	1.00
Proteobacteria	*Yersinia*	991	1094	2100	876	1211	633	0.65	0.58	1.00
Bacteroidetes	*Zunongwangia*	328	313	385	690	899	1145	2.67	0.00	0.07
Proteobacteria	*Zymomonas*	107	112	57	140	99	103	1.24	0.93	1.00
Firmicutes	unclassified (derived from *Alicyclobacillaceae*)	270	327	371	389	218	289	0.93	0.83	1.00
Cyanobacteria	unclassified (derived from *Chroococcales*)	22	25	10	23	40	37	1.79	0.26	1.00
Bacteroidetes	unclassified (derived from *Flavobacteriaceae*)	390	254	261	422	528	862	2.00	0.05	0.77
