# Supplementary material for: Metatranscriptome Sequencing Reveals Insights into the Gene Expression and Functional Potential of Rumen Wall Bacteria
Source: Front Microbiol. 2018 Jan 23;9:43. doi: 10.3389/fmicb.2018.00043 (PMC5787071; doi:10.3389/fmicb.2018.00043)
Supplement: Supplementary file 3 [file Table_3.PDF]

Table S3. Reads annotated as *Archaea* at phylum and genus level (only the 15 most abundant genera are shown). Baseline = mean of samples taken at the Baseline period; SARA = mean of samples taken at the SARA period. Duplicate reads were removed during quality control.

**Phylum**	**Genus**	**Baseline**	**SARA**	**Fold change**	***P* value**	**corrected *P* value**
Euryarchaeota	*Methanocaldococcus*	34494	10275	0.30	0.120	0.780
Euryarchaeota	*Methanobrevibacter*	3031	2802	0.92	0.860	1.000
Euryarchaeota	*Methanosarcina*	1279	750	0.59	0.550	0.940
Euryarchaeota	*Methanothermobacter*	674	692	1.03	0.910	1.000
Euryarchaeota	*Methanococcoides*	579	227	0.39	0.210	0.910
Euryarchaeota	*Methanococcus*	463	85	0.18	0.070	0.780
Euryarchaeota	*Thermococcus*	138	104	0.76	0.500	0.940
Euryarchaeota	*Methanohalobium*	135	129	0.95	0.580	0.940
Euryarchaeota	*Methanohalophilus*	118	141	1.19	0.710	0.970
Euryarchaeota	*Methanosphaera*	116	173	1.49	0.590	0.940
Crenarchaeota	*Sulfolobus*	113	44	0.39	0.700	0.970
Euryarchaeota	*Pyrococcus*	110	54	0.50	0.490	0.940
Euryarchaeota	*Archaeoglobus*	46	100	2.20	0.320	0.940
Euryarchaeota	*Aciduliprofundum*	40	150	3.78	0.150	0.780
Euryarchaeota	*Methanocorpusculum*	39	208	5.35	0.000	0.220
