# Supplementary material for: Metatranscriptome Sequencing Reveals Insights into the Gene Expression and Functional Potential of Rumen Wall Bacteria
Source: Front Microbiol. 2018 Jan 23;9:43. doi: 10.3389/fmicb.2018.00043 (PMC5787071; doi:10.3389/fmicb.2018.00043)
Supplement: Supplementary file 4 [file Table_4.PDF]

Table S4. Reads annotated to functional genes at 4 levels of KEGG orthology system. Features in red are defined to be highly expressed (top 10%), features in blue are defined to be low expressed (last 50%). All other features are defined to be moderately expressed. Duplicate reads were removed during quality control.

Level 1	Level 2	Level 3	Level function	Baseline 1	Baseline 2	Baseline 3	SARA 1	SARA 2	SARA 3
Metabolism	Carbohydrate metabolism	00620 Pyruvate metabolism [PATH:ko00620]	ppdK pyruvateorthophosphate dikinase [EC:2.7.9.1]	27932	50782	32627	28091	19065	29824
Environmental Information Processing	Glycolysis / Gluconeogenesis	00010 Glycolysis / Gluconeogenesis [PATH:ko00010]	ENO enolase [EC:4.2.1.11]	9903	15011	8132	8282	5820	8365
Cellular Processes	Cell motility	02040 Flagellar assembly [PATH:ko02040]	fliC flagellin	10604	10820	10841	10329	6545	7332
Organismal Systems	Translation	03010 Ribosome [PATH:ko03010]	tuf TUFM elongation factor Tu	8395	6390	7305	8236	6418	7649
Metabolism	Carbohydrate metabolism	00051 Fructose and mannose metabolism [PATH:ko00051]	E2.7.1.4 scrK fructokinase [EC:2.7.1.4]	4107	11447	4175	4202	2649	3684
Metabolism	Amino acid metabolism	00260 Glycine serine and threonine metabolism [PATH:ko00260]	gpmA PGAM 23-bisphosphoglycerate-dependent phosphoglycerate mutase [EC:5.4.2.1]	3859	5891	3520	3317	3619	3761
Environmental Information Processing	Glycolysis / Gluconeogenesis	00010 Glycolysis / Gluconeogenesis [PATH:ko00010]	GAPDH gapA glyceraldehyde 3-phosphate dehydrogenase [EC:1.2.1.12]	2317	2486	1639	2619	2246	3414
Genetic Information Processing	Folding sorting and degradation	03018 RNA degradation [PATH:ko03018]	dnaK molecular chaperone DnaK	2221	3438	2006	2369	2422	2358
Genetic Information Processing	Transcription	03020 RNA polymerase [PATH:ko03020]	rpoC DNA-directed RNA polymerase subunit beta' [EC:2.7.7.6]	2515	1804	1869	3117	2100	2473
Environmental Information Processing	Signal transduction	02020 Two-component system [PATH:ko02020]	pilA type IV pilus assembly protein PilA	3572	1862	2696	1671	2435	1527
Genetic Information Processing	Transcription	03020 RNA polymerase [PATH:ko03020]	rpoA DNA-directed RNA polymerase subunit alpha [EC:2.7.7.6]	2901	1918	3656	2704	1470	2023
Metabolism	Carbohydrate metabolism	00520 Amino sugar and nucleotide sugar metabolism [PATH:ko00520]	E2.5.1.56 neuB N-acetylneuraminate synthase [EC:2.5.1.56]	3666	2960	3645	2022	530	1134

Genetic Information Processing	Translation	03010 Ribosome [PATH:ko03010]	RP-S21 rpsU small subunit ribosomal protein S21	2040	1522	1502	2326	2262	1919
Genetic Information Processing	Transcription	03020 RNA polymerase [PATH:ko03020]	rpoB DNA-directed RNA polymerase subunit beta [EC:2.7.7.6]	2078	1580	1666	2314	1754	1755
Metabolism	Carbohydrate metabolism	00500 Starch and sucrose metabolism [PATH:ko00500]	E2.4.1.1 glgP PYG starch phosphorylase [EC:2.4.1.1]	1478	3117	1097	1669	1434	1752
Genetic Information Processing	Translation	03010 Ribosome [PATH:ko03010]	RP-L31 rpmE large subunit ribosomal protein L31	1945	1239	1588	1729	1658	1963
Metabolism	Amino acid metabolism	00250 Alanine aspartate and glutamate metabolism [PATH:ko00250]	E1.4.1.4 gdhA glutamate dehydrogenase (NADP+) [EC:1.4.1.4]	1861	1511	1342	1548	1292	2157
Genetic Information Processing	Folding sorting and degradation	03018 RNA degradation [PATH:ko03018]	groEL HSPD1 chaperonin GroEL	1686	1971	1639	1896	1418	1522
Environmental Information Processing	Membrane transport	03070 Bacterial secretion system [PATH:ko03070]	gspG general secretion pathway protein G	2026	2011	3009	1799	945	1365
Genetic Information Processing	Translation	03010 Ribosome [PATH:ko03010]	RP-L19 rplS large subunit ribosomal protein L19	1485	1162	1315	2077	1607	1784
Metabolism	Glycolysis / Gluconeogenesis	00010 Glycolysis / Gluconeogenesis [PATH:ko00010]	glk glucokinase [EC:2.7.1.2]	1868	3762	1151	889	499	1522
Environmental Information Processing	Pyruvate metabolism	Carbon metabolism [PATH:ko01200]	E1.1.1.38 sfcA maeA malate dehydrogenase (oxaloacetate-decarboxylating) [EC:1.1.1.38]	1536	2852	702	1070	1472	1360
Genetic Information Processing	Translation	03010 Ribosome [PATH:ko03010]	RP-L1 rplA large subunit ribosomal protein L1	1621	1194	1104	2042	1597	1518
Genetic Information Processing	Translation	03010 Ribosome [PATH:ko03010]	RP-L13 rplM large subunit ribosomal protein L13	1565	1281	1276	1553	1814	1524
Human Diseases	nitrogen metabolism	00330 Arginine and proline metabolism [PATH:ko00330]	ureC urease subunit alpha [EC:3.5.1.5]	2524	1852	4238	1640	495	949
Metabolism	Amino acid metabolism	00300 Lysine biosynthesis [PATH:ko00300]	lysA diaminopimelate decarboxylase [EC:4.1.1.20]	1747	3700	2661	1201	817	719
Genetic Information Processing	Translation	03010 Ribosome [PATH:ko03010]	RP-L7 rplL large subunit ribosomal protein L7/L12	1412	1059	1249	1883	1336	1455

Metabolism	Glycolysis / Gluconeogenesis	00010 Glycolysis / Gluconeogenesis [PATH:ko00010]	pgm phosphoglucomutase [EC:5.4.2.2]	913	3096	1249	1113	1270	1162
Genetic Information Processing	Translation	03010 Ribosome [PATH:ko03010]	RP-S2 rpsB small subunit ribosomal protein S2	1438	1216	1385	1511	1529	1293
Genetic Information Processing	Translation	03010 Ribosome [PATH:ko03010]	RP-S15 rpsO small subunit ribosomal protein S15	1313	1031	1167	1383	1505	1505
Metabolism	Nucleotide metabolism	00230 Purine metabolism [PATH:ko00230]	nrdD ribonucleoside-triphosphate reductase [EC:1.17.4.2]	1498	1481	1452	1437	945	1332
Genetic Information Processing	Translation	03010 Ribosome [PATH:ko03010]	RP-L20 rplT large subunit ribosomal protein L20	1159	986	1190	1565	1313	1477
Genetic Information Processing	Translation	03010 Ribosome [PATH:ko03010]	RP-L21 rplU large subunit ribosomal protein L21	1079	1171	1210	1444	1460	1280
Genetic Information Processing	Translation	03010 Ribosome [PATH:ko03010]	RP-S20 rpsT small subunit ribosomal protein S20	1114	886	925	1362	1512	1433
Human Diseases	Glycolysis / Gluconeogenesis	00010 Glycolysis / Gluconeogenesis [PATH:ko00010]	PK pyk pyruvate kinase [EC:2.7.1.40]	1114	2703	460	814	934	1108
Genetic Information Processing	Translation	03010 Ribosome [PATH:ko03010]	RP-S4 rpsD small subunit ribosomal protein S4	1357	1106	1210	1537	1148	1086
Genetic Information Processing	Translation	03010 Ribosome [PATH:ko03010]	RP-L27 rpmA large subunit ribosomal protein L27	1272	935	1272	1559	1201	1149
Metabolism	oxidative stress response	00450 Selenocompound metabolism [PATH:ko00450]	E1.8.1.9 trxB thioredoxin reductase (NADPH) [EC:1.8.1.9]	1478	1704	2377	1080	896	788
Environmental Information Processing	Membrane transport	03070 Bacterial secretion system [PATH:ko03070]	secY preprotein translocase subunit SecY	1283	1009	1182	1657	979	1144
Genetic Information Processing	Translation	03010 Ribosome [PATH:ko03010]	RP-L3 rplC large subunit ribosomal protein L3	1265	955	1323	1538	948	1016
Genetic Information Processing	Translation	03010 Ribosome [PATH:ko03010]	RP-L11 rplK large subunit ribosomal protein L11	1152	992	952	1266	1249	965
Genetic Information Processing	Translation	03010 Ribosome [PATH:ko03010]	RP-S9 rpsI small subunit ribosomal protein S9	1138	1096	1046	1349	959	1016
Metabolism	Carbohydrate metabolism	00620 Pyruvate metabolism [PATH:ko00620]	E2.3.1.54 pflD formate C-acetyltransferase [EC:2.3.1.54]	815	1796	1393	720	1077	959
Metabolism	Glycolysis / Gluconeogenesis	00010 Glycolysis / Gluconeogenesis [PATH:ko00010]	E2.7.1.90 pfk pyrophosphate--fructose-6-phosphate 1-phosphotransferase [EC:2.7.1.90]	939	1286	987	1122	926	1151

Metabolism	Carbohydrate metabolism	00010 Glycolysis / Gluconeogenesis [PATH:ko00010]	E4.1.1.49 pckA phosphoenolpyruvate carboxykinase (ATP) [EC:4.1.1.49]	735	709	890	1308	1070	1463
Genetic Information Processing	Translation	03010 Ribosome [PATH:ko03010]	RP-L2 rplB large subunit ribosomal protein L2	1245	979	1046	1490	797	872
Genetic Information Processing	Translation	03010 Ribosome [PATH:ko03010]	RP-S12 rpsL small subunit ribosomal protein S12	996	831	819	1050	1071	1092
Metabolism	Carbohydrate metabolism	00010 Glycolysis / Gluconeogenesis [PATH:ko00010]	FBA fbaA fructose-bisphosphate aldolase class II [EC:4.1.2.13]	993	940	995	1209	769	1209
Metabolism	oxidative stress response	00480 Glutathione metabolism [PATH:ko00480]	E1.11.1.9 glutathione peroxidase [EC:1.11.1.9]	923	1736	1007	1028	801	814
Genetic Information Processing	Folding sorting and degradation	03018 RNA degradation [PATH:ko03018]	pnp PNPT1 polyribonucleotide nucleotidyltransferase [EC:2.7.7.8]	997	791	987	1126	881	1095
Genetic Information Processing	Translation	00970 Aminoacyl-tRNA biosynthesis [PATH:ko00970]	TARS thrS threonyl-tRNA synthetase [EC:6.1.1.3]	1009	846	761	836	913	1149
Genetic Information Processing	Transcription	03020 RNA polymerase [PATH:ko03020]	rpoBC DNA-directed RNA polymerase subunit beta-beta' [EC:2.7.7.6]	1041	1074	1030	919	966	803
Genetic Information Processing	Translation	03010 Ribosome [PATH:ko03010]	RP-S7 rpsG small subunit ribosomal protein S7	973	759	663	1189	940	949
Metabolism	Carbohydrate metabolism	00052 Galactose metabolism [PATH:ko00052]	galK galactokinase [EC:2.7.1.6]	937	2051	371	712	471	943
Genetic Information Processing	Translation	03010 Ribosome [PATH:ko03010]	RP-S3 rpsC small subunit ribosomal protein S3	1027	825	972	1419	723	824
Cellular Processes	oxidative stress response	04146 Peroxisome [PATH:ko04146]	SOD2 superoxide dismutase Fe-Mn family [EC:1.15.1.1]	957	1256	1616	763	924	671
Genetic Information Processing	Translation	03010 Ribosome [PATH:ko03010]	RP-L5 rplE large subunit ribosomal protein L5	1136	793	913	1433	765	674
Metabolism	Nucleotide metabolism	00230 Purine metabolism [PATH:ko00230]	E2.7.4.6 ndk nucleoside-diphosphate kinase [EC:2.7.4.6]	786	1468	851	686	906	720
Genetic Information Processing	Translation	03010 Ribosome [PATH:ko03010]	RP-L10 rplJ large subunit ribosomal protein L10	840	692	1019	1197	972	762
Metabolism	starch and sucrose metabolism	00500 Starch and sucrose metabolism [PATH:ko00500]	bglX beta-glucosidase [EC:3.2.1.21]	869	1999	203	804	379	784
Genetic Information Processing	Translation	03010 Ribosome [PATH:ko03010]	RP-S10 rpsJ small subunit ribosomal protein S10	924	735	745	1077	824	822

Genetic Information Processing	Translation	00970 Aminoacyl-tRNA biosynthesis [PATH:ko00970]	IARS ileS isoleucyl-tRNA synthetase [EC:6.1.1.5]	815	802	620	926	844	966
Cellular Processes	citrate cycle	00020 Citrate cycle (TCA cycle) [PATH:ko00020]	IDH1 IDH2 icd isocitrate dehydrogenase [EC:1.1.1.42]	1044	840	925	720	794	805
Genetic Information Processing	Translation	03010 Ribosome [PATH:ko03010]	RP-S16 rpsP small subunit ribosomal protein S16	678	619	671	1019	896	1062
Environmental Information Processing	Membrane transport	02010 ABC transporters [PATH:ko02010]	ABC.GGU.S chvE putative multiple sugar transport system substrate-binding protein	1168	1138	788	1045	395	669
Metabolism	Carbohydrate metabolism	00620 Pyruvate metabolism [PATH:ko00620]	E1.1.1.40 maeB malate dehydrogenase (oxaloacetate-decarboxylating)(NADP+) [EC:1.1.1.40]	789	1254	667	654	704	862
Genetic Information Processing	Translation	03010 Ribosome [PATH:ko03010]	RP-L28 rpmB large subunit ribosomal protein L28	933	690	788	819	886	727
Metabolism	Carbohydrate metabolism	00020 Citrate cycle (TCA cycle) [PATH:ko00020]	mdh malate dehydrogenase [EC:1.1.1.37]	822	946	558	710	868	737
Metabolism	Energy metabolism	00190 Oxidative phosphorylation [PATH:ko00190]	ATPF1A atpA F-type H+-transporting ATPase subunit alpha [EC:3.6.3.14]	955	666	886	850	752	683
Metabolism	Energy metabolism	00190 Oxidative phosphorylation [PATH:ko00190]	nuoG NADH-quinone oxidoreductase subunit G [EC:1.6.5.3]	869	856	659	754	658	836
Genetic Information Processing	Translation	03010 Ribosome [PATH:ko03010]	RP-L17 rplQ large subunit ribosomal protein L17	772	590	819	917	769	849
Environmental Information Processing	Amino acid metabolism	00280 Valine, leucine and isoleucine degradation [PATH:ko00280]	E2.3.1.9 atoB acetyl-CoA C-acetyltransferase [EC:2.3.1.9]	966	834	792	662	601	791
Cellular Processes	Folding sorting and degradation	04112 Cell cycle - Caulobacter [PATH:ko04112]	lon ATP-dependent Lon protease [EC:3.4.21.53]	750	697	714	736	944	691
Metabolism	Amino acid metabolism	00250 Alanine aspartate and glutamate metabolism [PATH:ko00250]	aspA aspartate ammonia-lyase [EC:4.3.1.1]	495	1028	570	747	704	1007
Metabolism	Energy metabolism	00190 Oxidative phosphorylation [PATH:ko00190]	ATPF1B atpD F-type H+-transporting ATPase subunit beta [EC:3.6.3.14]	863	627	995	827	640	777

Environmental Information Processing	Membrane transport	03070 Bacterial secretion system [PATH:ko03070]	secA preprotein translocase subunit SecA	712	668	636	741	733	916
Genetic Information Processing	Translation	03010 Ribosome [PATH:ko03010]	RP-S6 rpsF small subunit ribosomal protein S6	644	702	570	764	959	688
Metabolism	Amino acid metabolism	00260 Glycine serine and threonine metabolism [PATH:ko00260]	E4.3.1.19 ilvA tdcB threonine dehydratase [EC:4.3.1.19]	541	1678	421	567	686	584
Genetic Information Processing	Translation	03010 Ribosome [PATH:ko03010]	RP-L6 rplF large subunit ribosomal protein L6	905	645	855	1036	585	626
Genetic Information Processing	Translation	03010 Ribosome [PATH:ko03010]	RP-L4 rplD large subunit ribosomal protein L4	846	637	823	1071	608	626
Metabolism	Amino acid metabolism	00290 Valine leucine and isoleucine biosynthesis [PATH:ko00290]	ilvC ketol-acid reductoisomerase [EC:1.1.1.86]	849	591	983	589	932	552
Genetic Information Processing	Translation	03010 Ribosome [PATH:ko03010]	RP-S13 rpsM small subunit ribosomal protein S13	780	576	800	961	676	710
Metabolism	Lipid metabolism	00061 Fatty acid biosynthesis [PATH:ko00061]	fabF 3-oxoacyl-[acyl-carrier-protein] synthase II [EC:2.3.1.179]	561	491	617	676	853	984
Genetic Information Processing	Translation	03010 Ribosome [PATH:ko03010]	RP-L33 rpmG large subunit ribosomal protein L33	633	419	574	853	919	741
Metabolism	Amino acid metabolism	00350 Tyrosine metabolism [PATH:ko00350]	E1.1.1.1 adh alcohol dehydrogenase [EC:1.1.1.1]	805	715	1069	519	691	647
Genetic Information Processing	Translation	03010 Ribosome [PATH:ko03010]	RP-S5 rpsE small subunit ribosomal protein S5	877	603	706	977	495	583
Metabolism	Amino acid metabolism	00290 Valine leucine and isoleucine biosynthesis [PATH:ko00290]	leuA 2-isopropylmalate synthase [EC:2.3.3.13]	1053	785	972	407	712	321
Genetic Information Processing	Translation	03010 Ribosome [PATH:ko03010]	RP-S8 rpsH small subunit ribosomal protein S8	840	558	800	966	581	536
Metabolism	Energy metabolism	00190 Oxidative phosphorylation [PATH:ko00190]	ppa inorganic pyrophosphatase [EC:3.6.1.1]	667	602	738	808	608	746
Metabolism	Carbohydrate metabolism	00010 Glycolysis / Gluconeogenesis [PATH:ko00010]	PGK pgk phosphoglycerate kinase [EC:2.7.2.3]	680	591	527	734	598	808
Metabolism	Amino acid metabolism	00260 Glycine serine and threonine metabolism [PATH:ko00260]	DLD lpd pdhD dihydrolipoamide dehydrogenase [EC:1.8.1.4]	974	699	1225	133	926	265

Metabolism	Carbohydrate metabolism	00052 Galactose metabolism [PATH:ko00052]	galE GALE UDP-glucose 4-epimerase [EC:5.1.3.2]	652	1372	371	520	464	572
Genetic Information Processing	Translation	00970 Aminoacyl-tRNA biosynthesis [PATH:ko00970]	VARS valS valyl-tRNA synthetase [EC:6.1.1.9]	665	711	687	782	546	688
Metabolism	Carbohydrate metabolism	00620 Pyruvate metabolism [PATH:ko00620]	ackA acetate kinase [EC:2.7.2.1]	700	643	601	616	641	669
Genetic Information Processing	Translation	03010 Ribosome [PATH:ko03010]	RP-L15 rplO large subunit ribosomal protein L15	817	539	800	860	507	538
Genetic Information Processing	Translation	00970 Aminoacyl-tRNA biosynthesis [PATH:ko00970]	AARS alaS alanyl-tRNA synthetase [EC:6.1.1.7]	744	668	695	680	586	567
Metabolism	Carbohydrate metabolism	00630 Glyoxylate and dicarboxylate metabolism [PATH:ko00630]	crt 3-hydroxybutyryl-CoA dehydratase [EC:4.2.1.55]	547	880	542	676	619	626
Environmental Information Processing	Membrane transport	02010 ABC transporters [PATH:ko02010]	msmX msmK maltose/maltodextrin transport system ATP-binding protein	516	465	363	530	628	1030
Genetic Information Processing	Translation	03010 Ribosome [PATH:ko03010]	RP-S11 rpsK small subunit ribosomal protein S11	683	583	636	894	491	643
Metabolism	Carbohydrate metabolism	00030 Pentose phosphate pathway [PATH:ko00030]	PRPS prsA ribose-phosphate pyrophosphokinase [EC:2.7.6.1]	572	795	488	579	672	633
Environmental Information Processing	Membrane transport	03070 Bacterial secretion system [PATH:ko03070]	SRP54 ffh signal recognition particle subunit SRP54	675	607	531	676	660	589
Metabolism	Carbohydrate metabolism	00500 Starch and sucrose metabolism [PATH:ko00500]	glgC glucose-1-phosphate adenylyltransferase [EC:2.7.7.27]	432	680	418	650	772	699
Metabolism	Carbohydrate metabolism	00030 Pentose phosphate pathway [PATH:ko00030]	E2.2.1.1 tktA tktB transketolase [EC:2.2.1.1]	585	597	460	554	578	798
Environmental Information Processing	Amino acid metabolism	01230 Biosynthesis of amino acids [PATH:ko01230]	glnA glutamine synthetase [EC:6.3.1.2]	578	627	578	606	678	608
Cellular Processes	Cell growth and death	04112 Cell cycle - Caulobacter [PATH:ko04112]	ftsZ cell division protein FtsZ	711	538	667	511	606	624
Genetic Information Processing	Translation	03010 Ribosome [PATH:ko03010]	RP-L16 rplP large subunit ribosomal protein L16	707	597	687	894	437	518
Genetic Information Processing	Translation	03010 Ribosome [PATH:ko03010]	RP-S18 rpsR small subunit ribosomal protein S18	550	451	433	666	747	619
Genetic Information Processing	Transcription	03020 RNA polymerase [PATH:ko03020]	fliA RNA polymerase sigma factor for flagellar operon FliA	330	323	304	505	1155	607

Genetic Information Processing	Translation	03010 Ribosome [PATH:ko03010]	RP-L14 rplN large subunit ribosomal protein L14	710	502	617	814	563	481
Metabolism	Amino acid metabolism	00260 Glycine serine and threonine metabolism [PATH:ko00260]	glyA SHMT glycine hydroxymethyltransferase [EC:2.1.2.1]	528	488	460	517	636	762
Metabolism	Lipid metabolism	00061 Fatty acid biosynthesis [PATH:ko00061]	fabG 3-oxoacyl-[acyl-carrier protein] reductase [EC:1.1.1.100]	530	515	749	388	714	685
Genetic Information Processing	Translation	00970 Aminoacyl-tRNA biosynthesis [PATH:ko00970]	LARS leuS leucyl-tRNA synthetase [EC:6.1.1.4]	614	542	460	677	583	591
Metabolism	Glycolysis / Gluconeogenesis	00010 Glycolysis / Gluconeogenesis [PATH:ko00010]	GPI pgi glucose-6-phosphate isomerase [EC:5.3.1.9]	465	823	390	524	481	739
Genetic Information Processing	Translation	00970 Aminoacyl-tRNA biosynthesis [PATH:ko00970]	MARS metG methionyl-tRNA synthetase [EC:6.1.1.10]	593	563	468	508	553	661
Metabolism	Amino acid metabolism	00330 Arginine and proline metabolism [PATH:ko00330]	OTC argF argI ornithine carbamoyltransferase [EC:2.1.3.3]	438	517	457	542	588	763
Metabolism	Amino acid metabolism	00250 Alanine aspartate and glutamate metabolism [PATH:ko00250]	asnA aspartate--ammonia ligase [EC:6.3.1.1]	516	1029	242	456	583	436
Environmental Information Processing	Folding sorting and degradation	04141 Protein processing in endoplasmic reticulum [PATH:ko04141]	htpG HSP90A molecular chaperone HtpG	467	646	515	552	615	580
Metabolism	Amino acid metabolism	00270 Cysteine and methionine metabolism [PATH:ko00270]	E2.5.1.49 metY O-acetylhomoserine (thiol)-lyase [EC:2.5.1.49]	606	834	1978	504	448	267
Genetic Information Processing	Translation	03010 Ribosome [PATH:ko03010]	RP-L18 rplR large subunit ribosomal protein L18	676	426	648	751	483	481
Environmental Information Processing	Membrane transport	02020 Two-component system [PATH:ko02020]	tctC putative tricarboxylic transport membrane protein	755	401	749	519	542	420
Genetic Information Processing	Translation	03010 Ribosome [PATH:ko03010]	RP-L35 rpmI large subunit ribosomal protein L35	515	398	488	545	789	439
Metabolism	Amino acid metabolism	00250 Alanine aspartate and glutamate metabolism [PATH:ko00250]	carB CPA2 carbamoyl-phosphate synthase large subunit [EC:6.3.5.5]	520	452	585	579	519	622

Metabolism	Energy metabolism	00190 Oxidative phosphorylation [PATH:ko00190]	nuoF NADH-quinone oxidoreductase subunit F [EC:1.6.5.3]	640	545	472	535	362	638
Genetic Information Processing	Folding sorting and degradation	04141 Protein processing in endoplasmic reticulum [PATH:ko04141]	HSP20 HSP20 family protein	278	318	254	530	589	946
Metabolism	Amino acid metabolism	00280 Valine leucine and isoleucine degradation [PATH:ko00280]	ACADS bcd butyryl-CoA dehydrogenase [EC:1.3.8.1]	566	631	613	594	358	580
Environmental Information Processing	Membrane transport	02010 ABC transporters [PATH:ko02010]	livK branched-chain amino acid transport system substrate-binding protein	713	678	855	618	303	364
Genetic Information Processing	Translation	00970 Aminoacyl-tRNA biosynthesis [PATH:ko00970]	DARS aspS aspartyl-tRNA synthetase [EC:6.1.1.12]	546	503	484	544	513	533
Environmental Information Processing	Membrane transport	02010 ABC transporters [PATH:ko02010]	ABC.MET.S metQ D-methionine transport system substrate-binding protein	483	1072	1378	438	405	192
Metabolism	nitrogen metabolism	00330 Arginine and proline metabolism [PATH:ko00330]	ureA urease subunit gamma [EC:3.5.1.5]	865	423	1374	550	155	386
Genetic Information Processing	Translation	03010 Ribosome [PATH:ko03010]	RP-L34 rpmH large subunit ribosomal protein L34	476	302	293	710	454	640
Metabolism	Amino acid metabolism	00270 Cysteine and methionine metabolism [PATH:ko00270]	E2.5.1.6 metK S-adenosylmethionine synthetase [EC:2.5.1.6]	470	445	402	437	623	495
Genetic Information Processing	Replication and repair	03440 Homologous recombination [PATH:ko03440]	recA recombination protein RecA	457	430	476	471	560	537
Genetic Information Processing	Translation	00970 Aminoacyl-tRNA biosynthesis [PATH:ko00970]	KARS lysS lysyl-tRNA synthetase class II [EC:6.1.1.6]	485	441	406	509	521	520
Genetic Information Processing	Translation	00970 Aminoacyl-tRNA biosynthesis [PATH:ko00970]	PARS proS prolyl-tRNA synthetase [EC:6.1.1.15]	472	475	359	492	526	524
Metabolism	citrate cycle	00020 Citrate cycle (TCA cycle) [PATH:ko00020]	CS gltA citrate synthase [EC:2.3.3.1]	624	353	671	510	492	408
Genetic Information Processing	Translation	03010 Ribosome [PATH:ko03010]	RP-S19 rpsS small subunit ribosomal protein S19	534	461	609	699	408	422

Metabolism	Amino acid metabolism	00260 Glycine serine and threonine metabolism [PATH:ko00260]	serC PSAT1 phosphoserine aminotransferase [EC:2.6.1.52]	446	459	363	483	491	574
Metabolism	Carbohydrate metabolism	00650 Butanoate metabolism [PATH:ko00650]	ptb phosphate butyryltransferase [EC:2.3.1.19]	356	890	226	417	421	521
Genetic Information Processing	Translation	03010 Ribosome [PATH:ko03010]	RP-L9 rplI large subunit ribosomal protein L9	451	317	320	540	592	511
Cellular Processes	Folding sorting and degradation	04112 Cell cycle - Caulobacter [PATH:ko04112]	clpX CLPX ATP-dependent Clp protease ATP-binding subunit ClpX	501	459	464	469	456	512
Metabolism	Nucleotide metabolism	00230 Purine metabolism [PATH:ko00230]	E2.7.4.3 adk adenylate kinase [EC:2.7.4.3]	512	435	347	519	520	427
Cellular Processes	Membrane transport	02030 Bacterial chemotaxis [PATH:ko02030]	mglB methyl-galactoside transport system substrate-binding protein	478	401	636	622	349	556
Metabolism	citrate cycle	00020 Citrate cycle (TCA cycle) [PATH:ko00020]	korA 2-oxoglutarate ferredoxin oxidoreductase subunit alpha [EC:1.2.7.3]	414	366	558	580	458	560
Genetic Information Processing	Translation	03010 Ribosome [PATH:ko03010]	RP-S17 rpsQ small subunit ribosomal protein S17	546	363	527	621	430	393
Cellular Processes	Folding sorting and degradation	04112 Cell cycle - Caulobacter [PATH:ko04112]	clpP CLPP ATP-dependent Clp protease protease subunit [EC:3.4.21.92]	463	515	379	459	478	447
Genetic Information Processing	Translation	03010 Ribosome [PATH:ko03010]	RP-L25 rplY large subunit ribosomal protein L25	485	420	562	600	531	312
Metabolism	Nucleotide metabolism	00230 Purine metabolism [PATH:ko00230]	E1.1.1.205 guaB IMP dehydrogenase [EC:1.1.1.205]	429	338	398	476	440	596
Genetic Information Processing	Translation	03010 Ribosome [PATH:ko03010]	RP-L24 rplX large subunit ribosomal protein L24	460	370	363	572	494	425
Metabolism	Nucleotide metabolism	00230 Purine metabolism [PATH:ko00230]	E6.3.5.3 purL phosphoribosylformylglycinamidine synthase [EC:6.3.5.3]	377	353	476	551	465	546
Metabolism	Amino acid metabolism	00290 Valine leucine and isoleucine biosynthesis [PATH:ko00290]	E2.2.1.6L ilvB ilvG ilvI acetolactate synthase I/II/III large subunit [EC:2.2.1.6]	553	443	589	465	420	351
Environmental Information Processing	Membrane Transport	02010 ABC transporters [PATH:ko02010]	dppF dipeptide transport system ATP-binding protein	479	564	593	568	299	391
Metabolism	Energy metabolism	00910 Nitrogen metabolism [PATH:ko00910]	napA periplasmic nitrate reductase NapA [EC:1.7.99.4]	350	372	418	763	223	649

Genetic Information Processing	Translation	03010 Ribosome [PATH:ko03010]	RP-L22 rplV large subunit ribosomal protein L22	476	412	566	576	391	375
Metabolism	Amino acid metabolism	00250 Alanine aspartate and glutamate metabolism [PATH:ko00250]	gltD glutamate synthase (NADPH/NADH) small chain [EC:1.4.1.13 1.4.1.14]	380	337	324	563	362	598
Metabolism	Carbohydrate metabolism	00051 Fructose and mannose metabolism [PATH:ko00051]	E5.4.2.8 manB phosphomannomutase [EC:5.4.2.8]	310	381	336	424	473	598
Genetic Information Processing	Translation	00970 Aminoacyl-tRNA biosynthesis [PATH:ko00970]	RARS argS arginyl-tRNA synthetase [EC:6.1.1.19]	353	398	304	534	444	498
Environmental Information Processing	Pyruvate metabolism	00620 Pyruvate metabolism [PATH:ko00620]	E4.1.1.32 pckA PEPCK phosphoenolpyruvate carboxykinase (GTP) [EC:4.1.1.32]	483	441	332	340	183	715
Metabolism	citrate cycle	00020 Citrate cycle (TCA cycle) [PATH:ko00020]	acnB aconitate hydratase 2 / 2-methylisocitrate dehydratase [EC:4.2.1.3 4.2.1.99]	653	296	589	426	443	259
Genetic Information Processing	Folding sorting and degradation	04122 Sulfur relay system [PATH:ko04122]	iscS NFS1 cysteine desulfurase [EC:2.8.1.7]	550	650	609	406	370	222
Metabolism	Amino acid metabolism	00280 Valine leucine and isoleucine degradation [PATH:ko00280]	MUT methylmalonyl-CoA mutase [EC:5.4.99.2]	243	294	203	490	525	625
Genetic Information Processing	Translation	03010 Ribosome [PATH:ko03010]	RP-L23 rplW large subunit ribosomal protein L23	448	392	464	636	427	318
Metabolism	Amino acid metabolism	00260 Glycine serine and threonine metabolism [PATH:ko00260]	serA PHGDH D-3-phosphoglycerate dehydrogenase [EC:1.1.1.95]	393	382	398	432	408	528
Genetic Information Processing	sulfur metabolism	04122 Sulfur relay system [PATH:ko04122]	tusE dsrC tRNA 2-thiouridine synthesizing protein E [EC:2.8.1.-]	477	412	172	444	432	417
Human Diseases	citrate cycle	00020 Citrate cycle (TCA cycle) [PATH:ko00020]	sdhA succinate dehydrogenase flavoprotein subunit [EC 1.3.5.1]	444	359	433	330	457	446
Genetic Information Processing	Replication and repair	03030 DNA replication [PATH:ko03030]	ssb single-strand DNA-binding protein	463	446	515	474	393	335
Metabolism	Glycolysis / Gluconeogenesis	00010 Glycolysis / Gluconeogenesis [PATH:ko00010]	pfkA PFK 6-phosphofructokinase 1 [EC:2.7.1.11]	391	399	265	392	359	551
Genetic Information Processing	Translation	00970 Aminoacyl-tRNA biosynthesis [PATH:ko00970]	FARSB pheT phenylalanyl-tRNA synthetase beta chain [EC:6.1.1.20]	403	342	402	448	459	406

Metabolism	Pyruvate metabolism	00620 Pyruvate metabolism [PATH:ko00620]	LDH ldh L-lactate dehydrogenase [EC:1.1.1.27]	385	606	70	460	176	601
Genetic Information Processing	Translation	00970 Aminoacyl-tRNA biosynthesis [PATH:ko00970]	NARS asnS asparaginyl-tRNA synthetase [EC:6.1.1.22]	380	608	308	403	330	415
Cellular Processes	Cell motility	02030 Bacterial chemotaxis [PATH:ko02030]	cheY two-component system chemotaxis family response regulator CheY	497	448	519	478	282	340
Metabolism	Amino acid metabolism	00280 Valine leucine and isoleucine degradation [PATH:ko00280]	E2.6.1.42 ilvE branched-chain amino acid aminotransferase [EC:2.6.1.42]	360	359	351	364	368	472
Genetic Information Processing	Translation	03010 Ribosome [PATH:ko03010]	RP-L32 rpmF large subunit ribosomal protein L32	326	240	503	379	511	380
Metabolism	Amino acid metabolism	00250 Alanine aspartate and glutamate metabolism [PATH:ko00250]	E2.6.1.16 glmS glucosamine--fructose-6-phosphate aminotransferase (isomerizing) [EC:2.6.1.16]	304	345	293	332	446	459
Metabolism	Carbohydrate metabolism	00040 Pentose and glucuronate interconversions [PATH:ko00040]	UGDH ugd UDPglucose 6-dehydrogenase [EC:1.1.1.22]	352	573	156	325	410	321
Metabolism	Amino acid metabolism	00260 Glycine serine and threonine metabolism [PATH:ko00260]	asd aspartate-semialdehyde dehydrogenase [EC:1.2.1.11]	388	377	410	400	371	357
Genetic Information Processing	Replication and repair	03420 Nucleotide excision repair [PATH:ko03420]	uvrA excinuclease ABC subunit A	382	351	332	353	376	408
Cellular Processes	Cell motility	02030 Bacterial chemotaxis [PATH:ko02030]	cheA two-component system chemotaxis family sensor kinase CheA [EC:2.7.13.3]	452	380	457	444	274	329
Metabolism	Amino acid metabolism	00250 Alanine aspartate and glutamate metabolism [PATH:ko00250]	E6.3.4.5 argG argininosuccinate synthase [EC:6.3.4.5]	357	381	472	390	328	378
Metabolism	citrate cycle	00020 Citrate cycle (TCA cycle) [PATH:ko00020]	E6.4.1.1B pycB pyruvate carboxylase subunit B [EC:6.4.1.1]	433	320	425	436	284	359
Metabolism	Nucleotide metabolism	00230 Purine metabolism [PATH:ko00230]	E6.3.5.2 guaA GMP synthase (glutamine-hydrolysing) [EC:6.3.5.2]	318	286	336	354	370	450
Genetic Information Processing	Translation	00970 Aminoacyl-tRNA biosynthesis [PATH:ko00970]	SARS serS seryl-tRNA synthetase [EC:6.1.1.11]	345	316	351	375	380	373

Metabolism	Energy metabolism	00190 Oxidative phosphorylation [PATH:ko00190]	ATPF1G atpG F-type H+-transporting ATPase subunit gamma [EC:3.6.3.14]	405	284	340	368	400	307
Genetic Information Processing	Translation	00970 Aminoacyl-tRNA biosynthesis [PATH:ko00970]	GARS glyS1 glycyl-tRNA synthetase [EC:6.1.1.14]	346	331	285	383	297	448
Metabolism	Amino acid metabolism	00300 Lysine biosynthesis [PATH:ko00300]	dapA 4-hydroxy-tetrahydrodipicolinate synthase [EC:4.3.3.7]	345	253	382	281	493	331
Environmental Information Processing	Membrane transport	03070 Bacterial secretion system [PATH:ko03070]	secDF SecD/SecF fusion protein	229	199	164	396	377	579
Cellular Processes	fatty acid metabolism	01212 Fatty acid metabolism [PATH:ko01212]	ACSL fadD long-chain acyl-CoA synthetase [EC:6.2.1.3]	219	249	203	275	366	631
Genetic Information Processing	Replication and repair	03030 DNA replication [PATH:ko03030]	DPO3A1 dnaE DNA polymerase III subunit alpha [EC:2.7.7.7]	333	274	340	368	374	389
Genetic Information Processing	Translation	00970 Aminoacyl-tRNA biosynthesis [PATH:ko00970]	QARS glnS glutaminyl-tRNA synthetase [EC:6.1.1.18]	336	343	242	361	392	341
Cellular Processes	Cell motility	02030 Bacterial chemotaxis [PATH:ko02030]	mcp methyl-accepting chemotaxis protein	333	445	632	484	264	276
Metabolism	Glycolysis / Gluconeogenesis	00010 Glycolysis / Gluconeogenesis [PATH:ko00010]	TPI tpiA triosephosphate isomerase (TIM) [EC:5.3.1.1]	361	302	324	375	301	405
Metabolism	Amino acid metabolism	00270 Cysteine and methionine metabolism [PATH:ko00270]	cysK cysteine synthase A [EC:2.5.1.47]	312	425	808	426	340	220
Metabolism	citrate cycle	00020 Citrate cycle (TCA cycle) [PATH:ko00020]	korB 2-oxoglutarate ferredoxin oxidoreductase subunit beta [EC:1.2.7.3]	310	262	453	372	330	413
Metabolism	Carbohydrate metabolism	00500 Starch and sucrose metabolism [PATH:ko00500]	E2.4.1.21 glgA starch synthase [EC:2.4.1.21]	231	218	207	442	425	420
Metabolism	Nucleotide metabolism	00230 Purine metabolism [PATH:ko00230]	relA GTP pyrophosphokinase [EC:2.7.6.5]	333	328	277	340	367	347
Metabolism	Energy metabolism	00190 Oxidative phosphorylation [PATH:ko00190]	ATPF0C atpE F-type H+-transporting ATPase subunit c [EC:3.6.3.14]	441	288	429	329	271	335
Genetic Information Processing	Translation	00970 Aminoacyl-tRNA biosynthesis [PATH:ko00970]	YARS tyrS tyrosyl-tRNA synthetase [EC:6.1.1.1]	365	293	324	364	339	330

Metabolism	Metabolism of cofactors and vitamins	00670 One carbon pool by folate [PATH:ko00670]	purH phosphoribosylaminoimidazolecarboxamide formyltransferase / IMP cyclohydrolase [EC:2.1.2.3 3.5.4.10]	318	261	269	302	436	341
Genetic Information Processing	Translation	03010 Ribosome [PATH:ko03010]	RP-L29 rpmC large subunit ribosomal protein L29	371	288	324	498	316	232
Metabolism	Energy metabolism	00190 Oxidative phosphorylation [PATH:ko00190]	ATPVB ntpB V-type H+-transporting ATPase subunit B [EC:3.6.3.14]	359	321	472	463	220	314
Genetic Information Processing	Translation	00970 Aminoacyl-tRNA biosynthesis [PATH:ko00970]	EARS gltX glutamyl-tRNA synthetase [EC:6.1.1.17]	311	288	300	336	292	391
Metabolism	Energy metabolism	00910 Nitrogen metabolism [PATH:ko00910]	hao hydroxylamine oxidase [EC:1.7.3.4]	299	238	437	489	164	457
Genetic Information Processing	Replication and repair	03030 DNA replication [PATH:ko03030]	DPO3B dnaN DNA polymerase III subunit beta [EC:2.7.7.7]	205	185	152	247	466	454
Metabolism	Metabolism of terpenoids and polyketides	00900 Terpenoid backbone biosynthesis [PATH:ko00900]	E1.17.1.2 lytB ispH 4-hydroxy-3-methylbut-2-enyl diphosphate reductase [EC:1.17.1.2]	280	313	304	349	263	419
Metabolism	Amino acid metabolism	00250 Alanine aspartate and glutamate metabolism [PATH:ko00250]	E6.3.4.4 purA adenylosuccinate synthase [EC:6.3.4.4]	278	283	254	270	386	354
Metabolism	Carbohydrate metabolism	00520 Amino sugar and nucleotide sugar metabolism [PATH:ko00520]	murA UDP-N-acetylglucosamine 1-carboxyvinyltransferase [EC:2.5.1.7]	308	315	320	274	325	349
Metabolism	Glycolysis / Gluconeogenesis	00010 Glycolysis / Gluconeogenesis [PATH:ko00010]	E5.1.3.3 galM aldose 1-epimerase [EC:5.1.3.3]	230	698	160	169	287	301
Metabolism	Amino acid metabolism	00300 Lysine biosynthesis [PATH:ko00300]	ACO acnA aconitate hydratase 1 / homoaconitase [EC:4.2.1.3 4.2.1.-]	355	342	207	260	253	358
Genetic Information Processing	Translation	00970 Aminoacyl-tRNA biosynthesis [PATH:ko00970]	CARS cysS cysteinyl-tRNA synthetase [EC:6.1.1.16]	285	246	273	321	352	337
Metabolism	Metabolism of cofactors and vitamins	00760 Nicotinate and nicotinamide metabolism [PATH:ko00760]	deoD purine-nucleoside phosphorylase [EC:2.4.2.1]	280	449	273	266	321	272
Metabolism	Nucleotide metabolism	00240 Pyrimidine metabolism [PATH:ko00240]	E6.3.4.2 pyrG CTP synthase [EC:6.3.4.2]	261	258	351	308	377	321

Metabolism	Amino acid metabolism	00260 Glycine serine and threonine metabolism [PATH:ko00260]	E2.7.2.4 lysC aspartate kinase [EC:2.7.2.4]	355	309	340	298	273	279
Metabolism	Energy metabolism	00190 Oxidative phosphorylation [PATH:ko00190]	ATPVA ntpA V-type H+-transporting ATPase subunit A [EC:3.6.3.14]	344	292	312	396	175	336
Metabolism	Lipid metabolism	00061 Fatty acid biosynthesis [PATH:ko00061]	fabH 3-oxoacyl-[acyl-carrier-protein] synthase III [EC:2.3.1.180]	213	204	234	337	444	282
Metabolism	Carbohydrate metabolism	00051 Fructose and mannose metabolism [PATH:ko00051]	E1.1.1.- [EC:1.1.1.-]	206	428	187	235	321	337
Metabolism	Glycan biosynthesis and metabolism	00540 Lipopolysaccharide biosynthesis [PATH:ko00540]	lpxC UDP-3-O-[3-hydroxymyristoyl] N-acetylglucosamine deacetylase [EC:3.5.1.108]	319	339	414	222	348	213
Metabolism	Amino acid metabolism	00360 Phenylalanine metabolism [PATH:ko00360]	paaH hbd fadB mmgB 3-hydroxybutyryl-CoA dehydrogenase [EC:1.1.1.157]	336	349	293	302	208	309
Environmental Information Processing	Membrane Transport	02010 ABC transporters [PATH:ko02010]	ABC.PE.P1 peptide/nickel transport system permease protein	276	261	460	380	236	325
Metabolism	Biosynthesis of other secondary metabolites	00521 Streptomycin biosynthesis [PATH:ko00521]	E4.2.1.46 rfbB rffG dTDP-glucose 46-dehydratase [EC:4.2.1.46]	301	293	265	244	318	294
Environmental Information Processing	Membrane transport	03070 Bacterial secretion system [PATH:ko03070]	secG preprotein translocase subunit SecG	283	233	254	300	388	246
Genetic Information Processing	Translation	00970 Aminoacyl-tRNA biosynthesis [PATH:ko00970]	FARSA pheS phenylalanyl-tRNA synthetase alpha chain [EC:6.1.1.20]	267	244	289	366	300	299
Metabolism	Amino acid metabolism	00250 Alanine aspartate and glutamate metabolism [PATH:ko00250]	E2.4.2.14 purF amidophosphoribosyltransferase [EC:2.4.2.14]	212	179	176	264	274	480
Metabolism	Nucleotide metabolism	00230 Purine metabolism [PATH:ko00230]	purC phosphoribosylaminoimidazole-succinocarboxamide synthase [EC:6.3.2.6]	235	212	285	246	374	290
Metabolism	Carbohydrate metabolism	00500 Starch and sucrose metabolism [PATH:ko00500]	glgB 14-alpha-glucan branching enzyme [EC:2.4.1.18]	283	261	160	289	255	326

Metabolism	Amino acid metabolism	00280 Valine leucine and isoleucine degradation [PATH:ko00280]	PCCB pccB propionyl-CoA carboxylase beta chain [EC:6.4.1.3]	235	251	211	257	306	334
Genetic Information Processing	Translation	03010 Ribosome [PATH:ko03010]	RP-S14 rpsN small subunit ribosomal protein S14	257	225	269	365	297	263
Metabolism	Lipid metabolism	00561 Glycerolipid metabolism [PATH:ko00561]	E2.7.1.30 glpK glycerol kinase [EC:2.7.1.30]	250	419	246	235	170	352
Environmental Information Processing	Energy metabolism	00190 Oxidative phosphorylation [PATH:ko00190]	ccoN cytochrome c oxidase cbb3-type subunit I [EC:1.9.3.1]	398	244	332	223	231	229
Environmental Information Processing	Energy metabolism	00190 Oxidative phosphorylation [PATH:ko00190]	frdA fumarate reductase flavoprotein subunit [EC:1.3.99.1]	375	346	433	326	168	170
Cellular Processes	Replication and repair	04112 Cell cycle - Caulobacter [PATH:ko04112]	dnaA chromosomal replication initiator protein	259	285	269	251	320	238
Environmental Information Processing	Membrane transport	02010 ABC transporters [PATH:ko02010]	msbA ATP-binding cassette subfamily B bacterial MsbA [EC:3.6.3.-]	256	517	187	183	238	229
Metabolism	Nucleotide metabolism	00230 Purine metabolism [PATH:ko00230]	E1.17.4.1A nrdA nrdE ribonucleoside-diphosphate reductase alpha chain [EC:1.17.4.1]	329	226	133	232	303	241
Metabolism	Amino acid metabolism	00300 Lysine biosynthesis [PATH:ko00300]	dapB dihydrodipicolinate reductase [EC:1.3.1.26]	262	412	269	233	228	243
Metabolism	Amino acid metabolism	00330 Arginine and proline metabolism [PATH:ko00330]	arcC carbamate kinase [EC:2.7.2.2]	249	257	164	274	217	340
Metabolism	Amino acid metabolism	00250 Alanine aspartate and glutamate metabolism [PATH:ko00250]	E4.3.2.2 purB adenylosuccinate lyase [EC:4.3.2.2]	203	183	316	278	304	316
Metabolism	Amino acid metabolism	00330 Arginine and proline metabolism [PATH:ko00330]	E4.1.1.3B oadB oxaloacetate decarboxylase beta subunit [EC:4.1.1.3]	203	242	180	305	234	351
Metabolism	Amino acid metabolism	00290 Valine leucine and isoleucine biosynthesis [PATH:ko00290]	ilvD dihydroxy-acid dehydratase [EC:4.2.1.9]	272	303	324	255	234	239
Metabolism	Carbohydrate metabolism	00500 Starch and sucrose metabolism [PATH:ko00500]	malQ 4-alpha-glucanotransferase [EC:2.4.1.25]	218	185	199	271	315	295
Metabolism	Amino acid metabolism	00350 Tyrosine metabolism [PATH:ko00350]	adhE acetaldehyde dehydrogenase / alcohol dehydrogenase [EC:1.2.1.10 1.1.1.1]	242	267	137	225	269	300

Metabolism	Amino acid metabolism	00380 Tryptophan metabolism [PATH:ko00380]	tnaA tryptophanase [EC:4.1.99.1]	253	626	90	123	143	248
Metabolism	Energy metabolism	00190 Oxidative phosphorylation [PATH:ko00190]	ATPF0A atpB F-type H+-transporting ATPase subunit a [EC:3.6.3.14]	320	236	293	181	275	221
Metabolism	Energy metabolism	00720 Carbon fixation pathways in prokaryotes [PATH:ko00720]	folD methylenetetrahydrofolate dehydrogenase (NADP+) / methenyltetrahydrofolate cyclohydrolase [EC:1.5.1.5 3.5.4.9]	224	242	152	203	292	309
Metabolism	Energy metabolism	00920 Sulfur metabolism [PATH:ko00920]	E2.7.7.4C met3 sulfate adenylyltransferase [EC:2.7.7.4]	262	285	101	211	179	359
Genetic Information Processing	Transcription	03020 RNA polymerase [PATH:ko03020]	SIG3.2 rpoE RNA polymerase sigma-70 factor ECF subfamily	477	252	375	76	281	72
Metabolism	Nucleotide metabolism	00240 Pyrimidine metabolism [PATH:ko00240]	pyrH uridylate kinase [EC:2.7.4.22]	275	263	367	236	218	248
Genetic Information Processing	Transcription	03018 RNA degradation [PATH:ko03018]	rho transcription termination factor Rho	211	240	222	263	302	248
Environmental Information Processing	Membrane Transport	02010 ABC transporters [PATH:ko02010]	ABC.PE.A peptide/nickel transport system ATP-binding protein	279	245	332	258	215	234
Metabolism	Amino acid metabolism	00270 Cysteine and methionine metabolism [PATH:ko00270]	E3.3.1.1 ahcY adenosylhomocysteinase [EC:3.3.1.1]	263	263	148	187	226	297
Metabolism	Carbohydrate metabolism	00620 Pyruvate metabolism [PATH:ko00620]	E2.3.1.8 pta phosphate acetyltransferase [EC:2.3.1.8]	239	255	375	228	182	309
Genetic Information Processing	Translation	00970 Aminoacyl-tRNA biosynthesis [PATH:ko00970]	WARS trpS tryptophanyl-tRNA synthetase [EC:6.1.1.2]	203	229	152	251	297	265
Metabolism	Carbohydrate metabolism	00520 Amino sugar and nucleotide sugar metabolism [PATH:ko00520]	nagB GNPDA glucosamine-6-phosphate deaminase [EC:3.5.99.6]	197	256	238	278	301	220
Metabolism	Carbohydrate metabolism	00630 Glyoxylate and dicarboxylate metabolism [PATH:ko00630]	glcD glycolate oxidase [EC:1.1.3.15]	280	272	363	304	160	231
Genetic Information Processing	Replication and repair	03420 Nucleotide excision repair [PATH:ko03420]	uvrD pcrA DNA helicase II / ATP-dependent DNA helicase PcrA [EC:3.6.4.12]	202	254	226	216	265	286

Environmental Information Processing	Membrane transport	03070 Bacterial secretion system [PATH:ko03070]	secE preprotein translocase subunit SecE	199	154	242	334	310	229
Metabolism	Nucleotide metabolism	00230 Purine metabolism [PATH:ko00230]	purD phosphoribosylamine--glycine ligase [EC:6.3.4.13]	218	192	242	229	250	295
Environmental Information Processing	Membrane transport	02060 Phosphotransferase system (PTS) [PATH:ko02060]	PTS-Ula-EIIC ulaA sgaT PTS system ascorbate-specific IIC component	317	395	199	123	181	199
Metabolism	Amino acid metabolism	00270 Cysteine and methionine metabolism [PATH:ko00270]	E2.1.1.14 metE 5-methyltetrahydropteroyltriglutamate--homocysteine methyltransferase [EC:2.1.1.14]	249	350	371	191	267	140
Metabolism	Lipid metabolism	00561 Glycerolipid metabolism [PATH:ko00561]	plsX glycerol-3-phosphate acyltransferase PlsX [EC:2.3.1.15]	274	288	195	256	221	194
Metabolism	Glycolysis / Gluconeogenesis	00010 Glycolysis / Gluconeogenesis [PATH:ko00010]	E3.2.1.86B bglA 6-phospho-beta-glucosidase [EC:3.2.1.86]	222	675	78	136	97	211
Environmental Information Processing	Membrane transport	03070 Bacterial secretion system [PATH:ko03070]	gspD general secretion pathway protein D	280	219	261	255	169	259
Cellular Processes	Replication and repair	04112 Cell cycle - Caulobacter [PATH:ko04112]	dnaB replicative DNA helicase [EC:3.6.4.12]	161	194	156	167	330	291
Environmental Information Processing	Membrane transport	02020 Two-component system [PATH:ko02020]	atoE short-chain fatty acids transporter	334	288	410	287	127	144
Metabolism	Amino acid metabolism	00250 Alanine aspartate and glutamate metabolism [PATH:ko00250]	ASP5 aspartate aminotransferase chloroplastic [EC:2.6.1.1]	114	120	74	156	328	404
Metabolism	Carbohydrate metabolism	00620 Pyruvate metabolism [PATH:ko00620]	pps ppsA pyruvate water dikinase [EC:2.7.9.2]	311	207	226	105	296	171
Metabolism	Amino acid metabolism	00260 Glycine serine and threonine metabolism [PATH:ko00260]	GLDC gcvP glycine dehydrogenase [EC:1.4.4.2]	211	288	55	162	220	300
Metabolism	nitrogen metabolism	00330 Arginine and proline metabolism [PATH:ko00330]	ureB urease subunit beta [EC:3.5.1.5]	445	264	546	190	62	137
Metabolism	Glycan biosynthesis and metabolism	00550 Peptidoglycan biosynthesis [PATH:ko00550]	mrcA penicillin-binding protein 1A [EC:2.4.1.- 3.4.-.-]	196	216	230	214	225	298

Genetic Information Processing	Replication and repair	03030 DNA replication [PATH:ko03030]	DPO1 polA DNA polymerase I [EC:2.7.7.7]	207	225	156	202	242	279
Metabolism	Amino acid metabolism	00260 Glycine serine and threonine metabolism [PATH:ko00260]	kbl GCAT glycine C-acetyltransferase [EC:2.3.1.29]	155	228	183	218	209	351
Environmental Information Processing	Membrane transport	03070 Bacterial secretion system [PATH:ko03070]	yidC spoIIIJ OXA1 preprotein translocase subunit YidC	225	181	144	222	297	216
Metabolism	Amino acid metabolism	00270 Cysteine and methionine metabolism [PATH:ko00270]	E2.1.1.37 DNMT dcm DNA (cytosine-5-)-methyltransferase [EC:2.1.1.37]	228	163	219	242	240	250
Metabolism	Metabolism of cofactors and vitamins	00760 Nicotinate and nicotinamide metabolism [PATH:ko00760]	pncB NAPRT1 nicotinate phosphoribosyltransferase [EC:2.4.2.11]	227	197	258	182	274	221
Genetic Information Processing	Replication and repair	03030 DNA replication [PATH:ko03030]	E6.5.1.2 ligA ligB DNA ligase (NAD+) [EC:6.5.1.2]	222	219	265	214	219	242
Metabolism	Carbohydrate metabolism	00650 Butanoate metabolism [PATH:ko00650]	E1.1.1.30 bdh 3-hydroxybutyrate dehydrogenase [EC:1.1.1.30]	322	407	394	258	90	100
Metabolism	Nucleotide metabolism	00230 Purine metabolism [PATH:ko00230]	E2.4.2.7 apt adenine phosphoribosyltransferase [EC:2.4.2.7]	184	440	183	137	207	205
Metabolism	Amino acid metabolism	00360 Phenylalanine metabolism [PATH:ko00360]	paaK phenylacetate-CoA ligase [EC:6.2.1.30]	196	236	222	307	166	264
Metabolism	Biosynthesis of other secondary metabolites	00521 Streptomycin biosynthesis [PATH:ko00521]	E2.7.7.24 rfbA rffH glucose-1-phosphate thymidylyltransferase [EC:2.7.7.24]	203	204	199	223	239	250
Metabolism	Nucleotide metabolism	00240 Pyrimidine metabolism [PATH:ko00240]	upp UPRT uracil phosphoribosyltransferase [EC:2.4.2.9]	202	197	199	252	202	274
Metabolism	Glycan biosynthesis and metabolism	00550 Peptidoglycan biosynthesis [PATH:ko00550]	ddl D-alanine-D-alanine ligase [EC:6.3.2.4]	219	225	207	168	256	208
Metabolism	citrate cycle	00020 Citrate cycle (TCA cycle) [PATH:ko00020]	korG 2-oxoglutarate ferredoxin oxidoreductase subunit gamma [EC:1.2.7.3]	224	142	265	181	219	265
Metabolism	Glycan biosynthesis and metabolism	00550 Peptidoglycan biosynthesis [PATH:ko00550]	murC UDP-N-acetylmuramate--alanine ligase [EC:6.3.2.8]	199	218	199	214	222	229

Metabolism	Amino acid metabolism	00260 Glycine serine and threonine metabolism [PATH:ko00260]	thrC threonine synthase [EC:4.2.3.1]	182	217	230	222	210	238
Environmental Information Processing	Membrane transport	03070 Bacterial secretion system [PATH:ko03070]	secD preprotein translocase subunit SecD	243	254	234	278	173	147
Genetic Information Processing	Translation	00970 Aminoacyl-tRNA biosynthesis [PATH:ko00970]	HARS hisS histidyl-tRNA synthetase [EC:6.1.1.21]	199	209	199	211	220	213
Environmental Information Processing	Energy metabolism	00190 Oxidative phosphorylation [PATH:ko00190]	ccoP cytochrome c oxidase cbb3-type subunit III	267	171	343	140	225	176
Cellular Processes	Cell motility	02040 Flagellar assembly [PATH:ko02040]	flgE flagellar hook protein FlgE	235	245	340	281	138	171
Genetic Information Processing	Replication and repair	03018 RNA degradation [PATH:ko03018]	recQ ATP-dependent DNA helicase RecQ [EC:3.6.4.12]	103	85	168	200	295	325
Metabolism	Amino acid metabolism	00330 Arginine and proline metabolism [PATH:ko00330]	E4.1.1.3A oadA oxaloacetate decarboxylase alpha subunit [EC:4.1.1.3]	231	223	191	263	147	213
Metabolism	Amino acid metabolism	00300 Lysine biosynthesis [PATH:ko00300]	E1.4.1.16 diaminopimelate dehydrogenase [EC:1.4.1.16]	330	190	172	212	166	137
Environmental Information Processing	Membrane transport	02010 ABC transporters [PATH:ko02010]	ABC-2.AB.A antibiotic transport system ATP-binding protein	176	314	148	168	190	218
Metabolism	Amino acid metabolism	00480 Glutathione metabolism [PATH:ko00480]	K01270 pepD dipeptidase D [EC:3.4.13.-]	158	154	160	229	198	291
Environmental Information Processing	Signal transduction	02020 Two-component system [PATH:ko02020]	atoD acetate CoA-transferase alpha subunit [EC:2.8.3.8]	221	275	324	230	145	166
Metabolism	Nucleotide metabolism	00230 Purine metabolism [PATH:ko00230]	purM phosphoribosylformylglycinamidine cyclo-ligase [EC:6.3.3.1]	183	185	156	201	208	241
Metabolism	Carbohydrate metabolism	00630 Glyoxylate and dicarboxylate metabolism [PATH:ko00630]	E1.2.1.2A formate dehydrogenase alpha subunit [EC:1.2.1.2]	227	277	195	296	99	187
Metabolism	Amino acid metabolism	00250 Alanine aspartate and glutamate metabolism [PATH:ko00250]	E2.6.1.1A aspB aspartate aminotransferase [EC:2.6.1.1]	157	216	211	227	167	255
Metabolism	Amino acid metabolism	00260 Glycine serine and threonine metabolism [PATH:ko00260]	E1.1.1.3 homoserine dehydrogenase [EC:1.1.1.3]	254	163	172	137	283	120

Genetic Information Processing	Replication and repair	03420 Nucleotide excision repair [PATH:ko03420]	uvrB excinuclease ABC subunit B	197	161	144	192	212	223
Metabolism	Nucleotide metabolism	00240 Pyrimidine metabolism [PATH:ko00240]	E2.7.1.48 udk uridine kinase [EC:2.7.1.48]	188	231	168	208	191	193
Metabolism	Carbohydrate metabolism	00052 Galactose metabolism [PATH:ko00052]	lacZ beta-galactosidase [EC:3.2.1.23]	240	188	94	161	196	192
Genetic Information Processing	Translation	00970 Aminoacyl-tRNA biosynthesis [PATH:ko00970]	gatB aspartyl-tRNA(Asn)/glutamyl-tRNA (Gln) amidotransferase subunit B [EC:6.3.5.6 6.3.5.7]	225	212	254	180	176	174
Metabolism	Energy metabolism	00190 Oxidative phosphorylation [PATH:ko00190]	ATPF0B atpF F-type H+-transporting ATPase subunit b [EC:3.6.3.14]	252	156	297	115	239	140
Metabolism	Metabolism of terpenoids and polyketides	00900 Terpenoid backbone biosynthesis [PATH:ko00900]	uppS undecaprenyl diphosphate synthase [EC:2.5.1.31]	149	146	133	212	222	243
Metabolism	Lipid metabolism	00061 Fatty acid biosynthesis [PATH:ko00061]	fabD [acyl-carrier-protein] S-malonyltransferase [EC:2.3.1.39]	150	144	195	200	203	257
Metabolism	Amino acid metabolism	00290 Valine leucine and isoleucine biosynthesis [PATH:ko00290]	leuB 3-isopropylmalate dehydrogenase [EC:1.1.1.85]	238	189	258	193	162	170
Metabolism	Glycan biosynthesis and metabolism	00540 Lipopolysaccharide biosynthesis [PATH:ko00540]	lpxD UDP-3-O-[3-hydroxymyristoyl] glucosamine N-acyltransferase [EC:2.3.1.191]	136	163	215	193	202	257
Metabolism	Energy metabolism	00680 Methane metabolism [PATH:ko00680]	hdrA heterodisulfide reductase subunit A [EC:1.8.98.1]	208	149	113	182	159	253
Metabolism	Amino acid metabolism	00330 Arginine and proline metabolism [PATH:ko00330]	E4.1.1.19S speA arginine decarboxylase [EC:4.1.1.19]	119	134	140	187	247	258
Organismal Systems	Endocrine system	04910 Insulin signaling pathway [PATH:ko04910]	FLOT flotillin	227	336	246	111	150	134
Metabolism	Carbohydrate metabolism	00520 Amino sugar and nucleotide sugar metabolism [PATH:ko00520]	wecB UDP-N-acetylglucosamine 2-epimerase [EC:5.1.3.14]	205	111	129	202	234	173
Genetic Information Processing	Replication and repair	03030 DNA replication [PATH:ko03030]	DPO3G dnaX DNA polymerase III subunit gamma/tau [EC:2.7.7.7]	145	164	180	178	262	178
Metabolism	Amino acid metabolism	00330 Arginine and proline metabolism [PATH:ko00330]	E4.1.1.17 ODC1 speC speF ornithine decarboxylase [EC:4.1.1.17]	165	147	101	125	353	109
Metabolism	Amino acid metabolism	00290 Valine leucine and isoleucine biosynthesis [PATH:ko00290]	leuC 3-isopropylmalate/(R)-2-methylmalate dehydratase large subunit [EC:4.2.1.33 4.2.1.35]	182	198	285	176	144	220

Environmental Information Processing	Membrane transport	03070 Bacterial secretion system [PATH:ko03070]	ftsY fused signal recognition particle receptor	170	150	140	155	217	220
Metabolism	Amino acid metabolism	00480 Glutathione metabolism [PATH:ko00480]	pepN aminopeptidase N [EC:3.4.11.2]	124	295	98	140	229	173
Metabolism	Amino acid metabolism	00260 Glycine serine and threonine metabolism [PATH:ko00260]	gcvT AMT aminomethyltransferase [EC:2.1.2.10]	167	195	129	138	128	295
Metabolism	Carbohydrate metabolism	00520 Amino sugar and nucleotide sugar metabolism [PATH:ko00520]	glmM phosphoglucosamine mutase [EC:5.4.2.10]	192	216	273	179	186	141
Metabolism	Metabolism of cofactors and vitamins	00770 Pantothenate and CoA biosynthesis [PATH:ko00770]	coaBC dfp phosphopantothenoylcysteine decarboxylase / phosphopantothenate--cysteine ligase [EC:4.1.1.36 6.3.2.5]	144	160	156	180	176	255
Metabolism	Energy metabolism	00190 Oxidative phosphorylation [PATH:ko00190]	nuoE NADH-quinone oxidoreductase subunit E [EC:1.6.5.3]	206	203	164	146	149	203
Metabolism	Carbohydrate metabolism	00053 Ascorbate and aldarate metabolism [PATH:ko00053]	ulaG L-ascorbate 6-phosphate lactonase [EC:3.1.1.-]	183	248	168	122	286	63
Environmental Information Processing	Membrane transport	03070 Bacterial secretion system [PATH:ko03070]	yajC preprotein translocase subunit YajC	169	143	265	215	192	176
Metabolism	Amino acid metabolism	00250 Alanine aspartate and glutamate metabolism [PATH:ko00250]	argH ASL argininosuccinate lyase [EC:4.3.2.1]	176	186	207	167	190	179
Environmental Information Processing	Membrane transport	02010 ABC transporters [PATH:ko02010]	afuA fbpA iron(III) transport system substrate-binding protein	202	230	137	183	134	182
Cellular Processes	Cell motility	02030 Bacterial chemotaxis [PATH:ko02030]	cheV two-component system chemotaxis family response regulator CheV	165	305	324	189	135	135
Environmental Information Processing	Membrane transport	02010 ABC transporters [PATH:ko02010]	rbsA ribose transport system ATP-binding protein [EC:3.6.3.17]	219	261	180	174	152	117
Metabolism	Nucleotide metabolism	00230 Purine metabolism [PATH:ko00230]	E2.7.4.8 gmk guanylate kinase [EC:2.7.4.8]	187	212	219	112	214	146

Metabolism	Amino acid metabolism	00250 Alanine aspartate and glutamate metabolism [PATH:ko00250]	pyrB PYR2 aspartate carbamoyltransferase catalytic subunit [EC:2.1.3.2]	138	150	129	134	233	218
Cellular Processes	Cell motility	02030 Bacterial chemotaxis [PATH:ko02030]	cheX chemotaxis protein CheX	250	112	172	119	234	121
Genetic Information Processing	Replication and repair	03440 Homologous recombination [PATH:ko03440]	ruvB holliday junction DNA helicase RuvB [EC:3.6.4.12]	154	185	168	163	179	201
Metabolism	Energy metabolism	00020 Citrate cycle (TCA cycle) [PATH:ko00020]	sdhB succinate dehydrogenase iron-sulfur subunit [EC:1.3.99.1]	218	149	211	129	188	151
Metabolism	Amino acid metabolism	00350 Tyrosine metabolism [PATH:ko00350]	yiaY alcohol dehydrogenase [EC:1.1.1.1]	85	175	47	108	103	401
Metabolism	Energy metabolism	00920 Sulfur metabolism [PATH:ko00920]	aprA adenylylsulfate reductase subunit A [EC:1.8.99.2]	183	188	98	172	91	252
Metabolism	Amino acid metabolism	00330 Arginine and proline metabolism [PATH:ko00330]	argJ glutamate N-acetyltransferase / amino-acid N-acetyltransferase [EC:2.3.1.35 2.3.1.1]	181	181	199	108	205	155
Metabolism	Amino acid metabolism	00300 Lysine biosynthesis [PATH:ko00300]	E6.3.2.13 murE UDP-N-acetylmuramoyl-L-alanyl-D-glutamate--26-diaminopimelate ligase [EC:6.3.2.13]	166	169	187	178	149	198
Environmental Information Processing	Energy metabolism	00190 Oxidative phosphorylation [PATH:ko00190]	ccoO cytochrome c oxidase cbb3-type subunit II	222	127	324	135	152	165
Metabolism	Amino acid metabolism	00340 Histidine metabolism [PATH:ko00340]	hisD histidinol dehydrogenase [EC:1.1.1.23]	165	179	195	193	173	154
Environmental Information Processing	Energy metabolism	00190 Oxidative phosphorylation [PATH:ko00190]	CYC1 CYT1 petC ubiquinol-cytochrome c reductase cytochrome c1 subunit	235	149	273	151	165	110
Metabolism	Energy metabolism	00190 Oxidative phosphorylation [PATH:ko00190]	nuoD NADH-quinone oxidoreductase subunit D [EC:1.6.5.3]	245	164	273	113	166	109
Genetic Information Processing	Folding sorting and degradation	03018 RNA degradation [PATH:ko03018]	rnj ribonuclease J [EC:3.1.-.-]	162	210	203	176	127	168
Metabolism	Carbohydrate metabolism	00620 Pyruvate metabolism [PATH:ko00620]	mqo malate dehydrogenase (quinone) [EC:1.1.5.4]	219	155	199	219	103	123

Cellular Processes	Cell motility	02040 Flagellar assembly [PATH:ko02040]	flgG flagellar basal-body rod protein FlgG	181	156	211	237	102	149
Metabolism	Carbohydrate metabolism	00030 Pentose phosphate pathway [PATH:ko00030]	deoC DERA deoxyribose-phosphate aldolase [EC:4.1.2.4]	127	170	113	157	151	204
Human Diseases	Amino acid metabolism	05120 Epithelial cell signaling in Helicobacter pylori infection [PATH:ko05120]	K08303 putative protease [EC:3.4.-.-]	128	128	148	195	164	188
Genetic Information Processing	Replication and repair	03440 Homologous recombination [PATH:ko03440]	recG ATP-dependent DNA helicase RecG [EC:3.6.4.12]	146	139	90	146	174	187
Metabolism	Carbohydrate metabolism	00620 Pyruvate metabolism [PATH:ko00620]	accC acetyl-CoA carboxylase biotin carboxylase subunit [EC:6.4.1.2 6.3.4.14]	161	159	293	125	155	156
Genetic Information Processing	Folding sorting and degradation	03060 Protein export [PATH:ko03060]	lepB signal peptidase I [EC:3.4.21.89]	147	156	164	156	174	155
Environmental Information Processing	Energy metabolism	00190 Oxidative phosphorylation [PATH:ko00190]	CYTB petB ubiquinol-cytochrome c reductase cytochrome b subunit	212	145	281	167	129	112
Genetic Information Processing	Folding sorting and degradation	03018 RNA degradation [PATH:ko03018]	deaD ATP-dependent RNA helicase DeaD [EC:3.6.4.13]	101	342	74	113	125	170
Metabolism	Carbohydrate metabolism	00030 Pentose phosphate pathway [PATH:ko00030]	rpe RPE ribulose-phosphate 3-epimerase [EC:5.1.3.1]	136	121	113	127	199	184
Metabolism	starch and sucrose metabolism	00500 Starch and sucrose metabolism [PATH:ko00500]	E3.2.1.21 beta-glucosidase [EC:3.2.1.21]	110	521	47	86	68	119
Cellular Processes	Cell growth and death	04112 Cell cycle - Caulobacter [PATH:ko04112]	ftsA cell division protein FtsA	157	128	164	113	176	175
Genetic Information Processing	Replication and repair	03410 Base excision repair [PATH:ko03410]	recJ single-stranded-DNA-specific exonuclease [EC:3.1.-.-]	111	121	117	128	186	218
Metabolism	Amino acid metabolism	00280 Valine leucine and isoleucine degradation [PATH:ko00280]	E2.8.3.5B scoB 3-oxoacid CoA-transferase subunit B [EC:2.8.3.5]	191	221	265	180	113	89
Genetic Information Processing	Translation	03010 Ribosome [PATH:ko03010]	RP-L36 rpmJ large subunit ribosomal protein L36	244	139	273	147	127	88
Metabolism	Metabolism of Cofactors and Vitamins	00730 Thiamine metabolism [PATH:ko00730]	thiL thiamine-monophosphate kinase [EC:2.7.4.16]	115	170	101	163	177	170
Metabolism	Nucleotide metabolism	00240 Pyrimidine metabolism [PATH:ko00240]	pyrF orotidine-5'-phosphate decarboxylase [EC:4.1.1.23]	88	91	121	125	228	215

Metabolism	Energy metabolism	00720 Carbon fixation pathways in prokaryotes [PATH:ko00720]	fhs formate--tetrahydrofolate ligase [EC:6.3.4.3]	138	150	125	149	124	212
Environmental Information Processing	Folding sorting and degradation	02020 Two-component system [PATH:ko02020]	degP htrA serine protease Do [EC:3.4.21.107]	172	339	343	139	109	39
Metabolism	Carbohydrate metabolism	00620 Pyruvate metabolism [PATH:ko00620]	E3.1.2.1 ACH1 acetyl-CoA hydrolase [EC:3.1.2.1]	204	106	144	144	167	121
Genetic Information Processing	Translation	03010 Ribosome [PATH:ko03010]	RP-L30 rpmD large subunit ribosomal protein L30	150	93	176	224	155	147
Environmental Information Processing	Signal transduction	02020 Two-component system [PATH:ko02020]	tctA putative tricarboxylic transport membrane protein	228	115	183	159	131	101
Genetic Information Processing	Folding sorting and degradation	03018 RNA degradation [PATH:ko03018]	hfq host factor-I protein	174	94	242	78	253	73
Cellular Processes	Membrane transport	02010 ABC transporters [PATH:ko02010]	rbsB ribose transport system substrate-binding protein	154	256	121	109	150	99
Metabolism	Nucleotide metabolism	00240 Pyrimidine metabolism [PATH:ko00240]	pyrD dihydroorotate dehydrogenase (fumarate) [EC:1.3.98.1]	121	122	113	121	167	194
Metabolism	Amino acid metabolism	00310 Lysine degradation [PATH:ko00310]	OGDH sucA 2-oxoglutarate dehydrogenase E1 component [EC:1.2.4.2]	209	122	226	41	211	78
Metabolism	Amino acid metabolism	00260 Glycine serine and threonine metabolism [PATH:ko00260]	trpB tryptophan synthase beta chain [EC:4.2.1.20]	171	123	293	156	141	112
Environmental Information Processing	Signal transduction	02020 Two-component system [PATH:ko02020]	dcuB anaerobic C4-dicarboxylate transporter DcuB	109	92	152	207	123	208
Metabolism	Metabolism of cofactors and vitamins	00860 Porphyrin and chlorophyll metabolism [PATH:ko00860]	hemN hemZ oxygen-independent coproporphyrinogen III oxidase [EC:1.3.99.22]	144	126	144	125	187	129
Metabolism	Nucleotide metabolism	00240 Pyrimidine metabolism [PATH:ko00240]	pyrE orotate phosphoribosyltransferase [EC:2.4.2.10]	136	110	180	117	165	160
Environmental Information Processing	Membrane transport	02010 ABC transporters [PATH:ko02010]	potA spermidine/putrescine transport system ATP-binding protein [EC:3.6.3.31]	148	138	133	173	130	140

Metabolism	Nucleotide metabolism	00240 Pyrimidine metabolism [PATH:ko00240]	cmk cytidylate kinase [EC:2.7.4.14]	127	126	137	106	214	117
Metabolism	Metabolism of terpenoids and polyketides	00281 Geraniol degradation [PATH:ko00281]	E1.3.99.- [EC:1.3.99.-]	111	119	109	90	113	257
Metabolism	Metabolism of cofactors and vitamins	00770 Pantothenate and CoA biosynthesis [PATH:ko00770]	panC pantoate--beta-alanine ligase [EC:6.3.2.1]	87	78	117	110	188	217
Metabolism	Amino acid metabolism	00340 Histidine metabolism [PATH:ko00340]	hutU UROC1 urocanate hydratase [EC:4.2.1.49]	82	95	86	121	202	193
Metabolism	Metabolism of cofactors and vitamins	00860 Porphyrin and chlorophyll metabolism [PATH:ko00860]	hemL glutamate-1-semialdehyde 21-aminomutase [EC:5.4.3.8]	227	138	207	122	112	78
Metabolism	Metabolism of cofactors and vitamins	00760 Nicotinate and nicotinamide metabolism [PATH:ko00760]	pntA NAD(P) transhydrogenase subunit alpha [EC:1.6.1.2]	158	101	62	68	196	142
Metabolism	Amino acid metabolism	00250 Alanine aspartate and glutamate metabolism [PATH:ko00250]	carA CPA1 carbamoyl-phosphate synthase small subunit [EC:6.3.5.5]	151	101	117	117	180	128
Cellular Processes	Cell motility	02030 Bacterial chemotaxis [PATH:ko02030]	fliM flagellar motor switch protein FliM	212	128	105	173	88	112
Metabolism	Energy metabolism	00190 Oxidative phosphorylation [PATH:ko00190]	ATPVK ntpK V-type H+-transporting ATPase subunit K [EC:3.6.3.14]	190	151	121	150	68	152
Environmental Information Processing	Membrane transport	03070 Bacterial secretion system [PATH:ko03070]	gspE general secretion pathway protein E	171	150	148	110	114	142
Metabolism	Nucleotide metabolism	00240 Pyrimidine metabolism [PATH:ko00240]	URA4 pyrC dihydroorotase [EC:3.5.2.3]	123	122	176	114	143	171
Metabolism	Energy metabolism	00190 Oxidative phosphorylation [PATH:ko00190]	ATPF1E atpC F-type H+-transporting ATPase subunit epsilon [EC:3.6.3.14]	192	94	207	90	153	112
Genetic Information Processing	Translation	00970 Aminoacyl-tRNA biosynthesis [PATH:ko00970]	gatA aspartyl-tRNA(Asn)/glutamyl-tRNA (Gln) amidotransferase subunit A [EC:6.3.5.6 6.3.5.7]	146	142	140	121	131	144
Metabolism	Amino acid metabolism	00250 Alanine aspartate and glutamate metabolism [PATH:ko00250]	gudB rocG glutamate dehydrogenase [EC:1.4.1.2]	98	144	12	69	69	309

Metabolism	Amino acid metabolism	00250 Alanine aspartate and glutamate metabolism [PATH:ko00250]	E1.5.1.12 1-pyrroline-5-carboxylate dehydrogenase [EC:1.5.1.12]	77	86	101	77	162	241
Metabolism	Amino acid metabolism	00400 Phenylalanine tyrosine and tryptophan biosynthesis [PATH:ko00400]	aroQ qutE 3-dehydroquinate dehydratase II [EC:4.2.1.10]	163	145	137	103	164	88
Genetic Information Processing	Folding sorting and degradation	03018 RNA degradation [PATH:ko03018]	rnr vacB ribonuclease R [EC:3.1.-.-]	100	131	121	158	161	135
Metabolism	Energy metabolism	00190 Oxidative phosphorylation [PATH:ko00190]	nuoL NADH-quinone oxidoreductase subunit L [EC:1.6.5.3]	170	86	219	82	176	101
Metabolism	Amino acid metabolism	00260 Glycine serine and threonine metabolism [PATH:ko00260]	K06001 trpB tryptophan synthase beta chain [EC:4.2.1.20]	144	134	172	82	157	124
Metabolism	Lipid metabolism	00061 Fatty acid biosynthesis [PATH:ko00061]	fabZ 3-hydroxyacyl-[acyl-carrier-protein] dehydratase [EC:4.2.1.59]	109	114	105	126	153	164
Metabolism	Carbohydrate metabolism	00620 Pyruvate metabolism [PATH:ko00620]	E2.3.3.9 aceB glcB malate synthase [EC:2.3.3.9]	127	85	191	31	274	72
Genetic Information Processing	Folding sorting and degradation	03018 RNA degradation [PATH:ko03018]	nudH putative (di)nucleoside polyphosphate hydrolase [EC:3.6.1.-]	197	154	242	71	149	53
Metabolism	Amino acid metabolism	00270 Cysteine and methionine metabolism [PATH:ko00270]	E2.1.1.13 metH 5-methyltetrahydrofolate--homocysteine methyltransferase [EC:2.1.1.13]	100	101	101	142	105	218
Metabolism	Metabolism of cofactors and vitamins	00760 Nicotinate and nicotinamide metabolism [PATH:ko00760]	pntB NAD(P) transhydrogenase subunit beta [EC:1.6.1.2]	218	103	156	46	119	118
Metabolism	Amino acid metabolism	00330 Arginine and proline metabolism [PATH:ko00330]	argC N-acetyl-gamma-glutamyl-phosphate reductase [EC:1.2.1.38]	138	125	117	109	152	124
Metabolism	Carbohydrate metabolism	00030 Pentose phosphate pathway [PATH:ko00030]	rbsK RBKS ribokinase [EC:2.7.1.15]	127	346	90	77	90	79
Environmental Information Processing	Membrane transport	02010 ABC transporters [PATH:ko02010]	ABC.GGU.A gguA putative multiple sugar transport system ATP-binding protein	146	191	125	168	92	94
Metabolism	Metabolism of terpenoids and polyketides	00900 Terpenoid backbone biosynthesis [PATH:ko00900]	E1.17.7.1 gcpE ispG (E)-4-hydroxy-3-methylbut-2-enyl-diphosphate synthase [EC:1.17.7.1]	155	139	117	154	109	109

Metabolism	Metabolism of cofactors and vitamins	00730 Thiamine metabolism [PATH:ko00730]	thiH thiamine biosynthesis ThiH	113	101	125	162	130	149
Metabolism	Amino acid metabolism	00250 Alanine aspartate and glutamate metabolism [PATH:ko00250]	ald alanine dehydrogenase [EC:1.4.1.1]	111	125	191	151	126	134
Environmental Information Processing	Membrane transport	03070 Bacterial secretion system [PATH:ko03070]	tatA sec-independent protein translocase protein TatA	110	109	74	122	149	152
Metabolism	Energy metabolism	00020 Citrate cycle (TCA cycle) [PATH:ko00020]	sdhC succinate dehydrogenase cytochrome b556 subunit	157	94	125	88	196	71
Genetic Information Processing	Replication and repair	03030 DNA replication [PATH:ko03030]	dnaG DNA primase [EC:2.7.7.-]	131	115	55	113	152	119
Metabolism	Amino acid metabolism	00400 Phenylalanine tyrosine and tryptophan biosynthesis [PATH:ko00400]	aroB 3-dehydroquinate synthase [EC:4.2.3.4]	135	130	180	105	121	121
Cellular Processes	Cell motility	02030 Bacterial chemotaxis [PATH:ko02030]	cheR chemotaxis protein methyltransferase CheR [EC:2.1.1.80]	124	180	195	144	83	116
Metabolism	citrate cycle	00020 Citrate cycle (TCA cycle) [PATH:ko00020]	E4.2.1.2A fumA fumB fumarate hydratase class I [EC:4.2.1.2]	103	80	90	116	172	137
Metabolism	Metabolism of cofactors and vitamins	00770 Pantothenate and CoA biosynthesis [PATH:ko00770]	panB 3-methyl-2-oxobutanoate hydroxymethyltransferase [EC:2.1.2.11]	139	98	195	114	116	128
Metabolism	Metabolism of cofactors and vitamins	00860 Porphyrin and chlorophyll metabolism [PATH:ko00860]	hemB ALAD porphobilinogen synthase [EC:4.2.1.24]	154	138	191	116	122	78
Metabolism	Nucleotide metabolism	00240 Pyrimidine metabolism [PATH:ko00240]	E2.4.2.2 pdp pyrimidine-nucleoside phosphorylase [EC:2.4.2.2]	114	204	90	93	105	123
Metabolism	Amino acid metabolism	00330 Arginine and proline metabolism [PATH:ko00330]	proA glutamate-5-semialdehyde dehydrogenase [EC:1.2.1.41]	114	132	78	153	127	114
Environmental Information Processing	Membrane transport	02010 ABC transporters [PATH:ko02010]	ftsE cell division transport system ATP-binding protein	110	114	82	102	137	144
Metabolism	Carbohydrate metabolism	00030 Pentose phosphate pathway [PATH:ko00030]	kdgK 2-dehydro-3-deoxygluconokinase [EC:2.7.1.45]	119	91	90	167	86	164
Metabolism	Lipid metabolism	00564 Glycerophospholipid metabolism [PATH:ko00564]	E2.7.8.5 pgsA PGS1 CDP-diacylglycerol--glycerol-3-phosphate 3-phosphatidyltransferase [EC:2.7.8.5]	148	122	140	69	165	75

Metabolism	Amino acid metabolism	00330 Arginine and proline metabolism [PATH:ko00330]	argB acetylglutamate kinase [EC:2.7.2.8]	139	119	140	114	128	100
Environmental Information Processing	Membrane transport	02010 ABC transporters [PATH:ko02010]	livF branched-chain amino acid transport system ATP-binding protein	155	142	160	117	103	88
Human Diseases	Amino acid metabolism	00270 Cysteine and methionine metabolism [PATH:ko00270]	luxS S-ribosylhomocysteine lyase [EC:4.4.1.21]	111	152	74	112	121	126
Metabolism	Metabolism of terpenoids and polyketides	00900 Terpenoid backbone biosynthesis [PATH:ko00900]	dxr 1-deoxy-D-xylulose-5-phosphate reductoisomerase [EC:1.1.1.267]	101	120	113	148	113	138
Metabolism	Carbohydrate metabolism	00040 Pentose and glucuronate interconversions [PATH:ko00040]	xylA xylose isomerase [EC:5.3.1.5]	87	180	191	92	168	74
Metabolism	Carbohydrate metabolism	00040 Pentose and glucuronate interconversions [PATH:ko00040]	UGP2 galU galF UTP--glucose-1-phosphate uridylyltransferase [EC:2.7.7.9]	173	106	109	105	133	72
Metabolism	Metabolism of cofactors and vitamins	00860 Porphyrin and chlorophyll metabolism [PATH:ko00860]	ftnA ftn ferritin [EC:1.16.3.1]	64	107	133	92	170	152
Environmental Information Processing	Membrane Transport	02010 ABC transporters [PATH:ko02010]	ABC.PE.P peptide/nickel transport system permease protein	127	142	199	123	81	126
Metabolism	Glycan biosynthesis and metabolism	00540 Lipopolysaccharide biosynthesis [PATH:ko00540]	kdsA 2-dehydro-3-deoxyphosphooctonate aldolase (KDO 8-P synthase) [EC:2.5.1.55]	97	104	144	132	160	104
Metabolism	Amino acid metabolism	00260 Glycine serine and threonine metabolism [PATH:ko00260]	GATM glycine amidinotransferase [EC:2.1.4.1]	162	287	8	143	34	65
Metabolism	Carbohydrate metabolism	00051 Fructose and mannose metabolism [PATH:ko00051]	E4.2.1.47 gmd GDPmannose 46-dehydratase [EC:4.2.1.47]	101	121	86	120	119	147
Metabolism	Glycan biosynthesis and metabolism	00550 Peptidoglycan biosynthesis [PATH:ko00550]	murD UDP-N-acetylmuramoylalanine--D-glutamate ligase [EC:6.3.2.9]	105	102	129	111	121	148
Genetic Information Processing	Translation	00970 Aminoacyl-tRNA biosynthesis [PATH:ko00970]	MTFMT fmt methionyl-tRNA formyltransferase [EC:2.1.2.9]	101	98	101	93	136	153

Environmental Information Processing	Membrane transport	02010 ABC transporters [PATH:ko02010]	pstB phosphate transport system ATP-binding protein [EC:3.6.3.27]	184	114	172	112	38	135
Metabolism	Energy metabolism	00190 Oxidative phosphorylation [PATH:ko00190]	ATPVD ntpD V-type H+-transporting ATPase subunit D [EC:3.6.3.14]	148	101	82	146	90	122
Metabolism	Carbohydrate metabolism	00520 Amino sugar and nucleotide sugar metabolism [PATH:ko00520]	glmU bifunctional UDP-N-acetylglucosamine pyrophosphorylase / Glucosamine-1-phosphate N-acetyltransferase [EC:2.7.7.23 2.3.1.157]	109	122	180	104	139	105
Metabolism	Carbohydrate metabolism	00630 Glyoxylate and dicarboxylate metabolism [PATH:ko00630]	E1.1.1.36 phbB acetoacetyl-CoA reductase [EC:1.1.1.36]	125	135	281	71	112	108
Metabolism	Amino acid metabolism	00290 Valine leucine and isoleucine biosynthesis [PATH:ko00290]	leuD 3-isopropylmalate/(R)-2-methylmalate dehydratase small subunit [EC:4.2.1.33 4.2.1.35]	120	133	211	122	111	96
Genetic Information Processing	Replication and repair	03430 Mismatch repair [PATH:ko03430]	mutL DNA mismatch repair protein MutL	99	82	66	136	122	152
Environmental Information Processing	Membrane transport	02060 Phosphotransferase system (PTS) [PATH:ko02060]	PTS-Ntr-EIIA ptsN PTS system nitrogen regulatory IIA component [EC:2.7.1.69]	104	85	86	121	94	178
Metabolism	Metabolism of cofactors and vitamins	00760 Nicotinate and nicotinamide metabolism [PATH:ko00760]	pncA nicotinamidase/pyrazinamidase [EC:3.5.1.19 3.5.1.-]	84	168	78	88	142	116
Metabolism	Metabolism of cofactors and vitamins	00740 Riboflavin metabolism [PATH:ko00740]	ribB RIB3 34-dihydroxy 2-butanone 4-phosphate synthase [EC:4.1.99.12]	103	100	121	125	128	126
Metabolism	Carbohydrate metabolism	00030 Pentose phosphate pathway [PATH:ko00030]	E2.2.1.2 talA talB transaldolase [EC:2.2.1.2]	100	84	133	74	202	88
Genetic Information Processing	Folding sorting and degradation	04122 Sulfur relay system [PATH:ko04122]	mnmA trmU TRMU tRNA-specific 2-thiouridylase [EC:2.8.1.-]	107	114	105	104	103	145
Metabolism	Metabolism of cofactors and vitamins	00730 Thiamine metabolism [PATH:ko00730]	sufS cysteine desulfurase / selenocysteine lyase [EC:2.8.1.7 4.4.1.16]	95	101	55	108	125	149
Metabolism	Amino acid metabolism	00300 Lysine biosynthesis [PATH:ko00300]	LYS1 saccharopine dehydrogenase (NAD+ L-lysine forming) [EC:1.5.1.7]	117	127	156	147	91	104

Human Diseases	nitrogen metabolism	00330 Arginine and proline metabolism [PATH:ko00330]	ureAB urease subunit gamma/beta [EC:3.5.1.5]	160	168	250	67	60	95
Genetic Information Processing	Replication and repair	03410 Base excision repair [PATH:ko03410]	E3.1.11.2 xthA exodeoxyribonuclease III [EC:3.1.11.2]	106	89	140	102	137	114
Environmental Information Processing	Membrane transport	03070 Bacterial secretion system [PATH:ko03070]	K11903 hcp type VI secretion system secreted protein Hcp	152	179	55	117	115	35
Metabolism	Glycan biosynthesis and metabolism	00550 Peptidoglycan biosynthesis [PATH:ko00550]	mraY phospho-N-acetylmuramoyl-pentapeptide-transferase [EC:2.7.8.13]	117	86	121	119	125	110
Metabolism	Amino acid metabolism	00400 Phenylalanine tyrosine and tryptophan biosynthesis [PATH:ko00400]	aroC chorismate synthase [EC:4.2.3.5]	90	114	140	92	135	121
Environmental Information Processing	Membrane transport	02010 ABC transporters [PATH:ko02010]	livG branched-chain amino acid transport system ATP-binding protein	148	151	152	98	85	78
Environmental Information Processing	Membrane transport	03070 Bacterial secretion system [PATH:ko03070]	secF preprotein translocase subunit SecF	119	107	148	116	122	87
Metabolism	Lipid metabolism	00061 Fatty acid biosynthesis [PATH:ko00061]	fabI enoyl-[acyl-carrier protein] reductase I [EC:1.3.1.9 1.3.1.10]	110	101	109	54	163	100
Cellular Processes	Cell motility	02030 Bacterial chemotaxis [PATH:ko02030]	motB chemotaxis protein MotB	116	96	129	156	105	96
Environmental Information Processing	Membrane Transport	02010 ABC transporters [PATH:ko02010]	ABC.PE.S peptide/nickel transport system substrate-binding protein	123	120	160	112	78	121
Metabolism	Amino acid metabolism	00270 Cysteine and methionine metabolism [PATH:ko00270]	E2.3.1.30 cysE serine O-acetyltransferase [EC:2.3.1.30]	117	108	113	81	118	114
Metabolism	Lipid metabolism	00564 Glycerophospholipid metabolism [PATH:ko00564]	gpsA glycerol-3-phosphate dehydrogenase (NAD(P)+) [EC:1.1.1.94]	94	127	160	121	112	103
Metabolism	Biosynthesis of other secondary metabolites	00521 Streptomycin biosynthesis [PATH:ko00521]	rfbD dTDP-4-dehydrorhamnose reductase [EC:1.1.1.133]	94	90	66	105	134	123
Genetic Information Processing	Translation	00970 Aminoacyl-tRNA biosynthesis [PATH:ko00970]	glyS glycyl-tRNA synthetase beta chain [EC:6.1.1.14]	120	101	129	86	143	77
Genetic Information Processing	Translation	00970 Aminoacyl-tRNA biosynthesis [PATH:ko00970]	glyQ glycyl-tRNA synthetase alpha chain [EC:6.1.1.14]	122	85	133	105	124	90

Cellular Processes	Cell motility	02040 Flagellar assembly [PATH:ko02040]	flgK flagellar hook-associated protein 1 FlgK	113	114	172	150	81	94
Environmental Information Processing	Membrane transport	02010 ABC transporters [PATH:ko02010]	lptB lipopolysaccharide export system ATP-binding protein [EC:3.6.3.-]	97	95	82	93	133	114
Metabolism	Amino acid metabolism	00310 Lysine degradation [PATH:ko00310]	E5.4.3.3 beta-lysine 56-aminomutase [EC:5.4.3.3]	81	75	74	100	104	171
Metabolism	Amino acid metabolism	00270 Cysteine and methionine metabolism [PATH:ko00270]	patB malY cystathione beta-lyase [EC:4.4.1.8]	59	127	51	67	128	158
Metabolism	Metabolism of cofactors and vitamins	00670 One carbon pool by folate [PATH:ko00670]	E2.1.1.45 thyA thymidylate synthase [EC:2.1.1.45]	113	100	125	79	121	106
Genetic Information Processing	Replication and repair	03030 DNA replication [PATH:ko03030]	DPO3A2 polC DNA polymerase III subunit alpha Gram-positive type [EC:2.7.7.7]	107	129	113	104	82	122
Metabolism	Amino acid metabolism	00400 Phenylalanine tyrosine and tryptophan biosynthesis [PATH:ko00400]	pheA2 prephenate dehydratase [EC:4.2.1.51]	122	198	125	84	81	70
Metabolism	Carbohydrate metabolism	00520 Amino sugar and nucleotide sugar metabolism [PATH:ko00520]	npdA NAD-dependent deacetylase [EC:3.5.1.-]	87	80	105	104	124	128
Metabolism	citrate cycle	00020 Citrate cycle (TCA cycle) [PATH:ko00020]	sucC succinyl-CoA synthetase beta subunit [EC:6.2.1.5]	125	103	51	108	81	124
Metabolism	Amino acid metabolism	00340 Histidine metabolism [PATH:ko00340]	hisC histidinol-phosphate aminotransferase [EC:2.6.1.9]	97	99	156	122	109	98
Metabolism	Nucleotide metabolism	00230 Purine metabolism [PATH:ko00230]	E1.17.4.1B nrdB nrdF ribonucleoside-diphosphate reductase beta chain [EC:1.17.4.1]	116	95	144	114	115	78
Genetic Information Processing	Folding sorting and degradation	03018 RNA degradation [PATH:ko03018]	ppk polyphosphate kinase [EC:2.7.4.1]	103	99	254	106	93	100
Environmental Information Processing	Membrane transport	02010 ABC transporters [PATH:ko02010]	cbiO cobalt/nickel transport system ATP-binding protein	118	125	121	102	91	91
Metabolism	Carbohydrate metabolism	00620 Pyruvate metabolism [PATH:ko00620]	accD acetyl-CoA carboxylase carboxyl transferase subunit beta [EC:6.4.1.2]	109	115	137	76	128	79
Metabolism	Metabolism of cofactors and vitamins	00770 Pantothenate and CoA biosynthesis [PATH:ko00770]	E2.7.7.3A coaD kdtB pantetheine-phosphate adenylyltransferase [EC:2.7.7.3]	118	119	94	91	106	87

Metabolism	Amino acid metabolism	00290 Valine leucine and isoleucine biosynthesis [PATH:ko00290]	E2.2.1.6S ilvH ilvN acetolactate synthase I/III small subunit [EC:2.2.1.6]	125	111	125	111	105	69
Metabolism	Metabolism of terpenoids and polyketides	00900 Terpenoid backbone biosynthesis [PATH:ko00900]	ispE 4-diphosphocytidyl-2-C-methyl-D-erythritol kinase [EC:2.7.1.148]	92	90	140	78	120	117
Metabolism	Amino acid metabolism	00260 Glycine serine and threonine metabolism [PATH:ko00260]	ltaE threonine aldolase [EC:4.1.2.5]	75	116	43	77	145	109
Metabolism	Carbohydrate metabolism	00051 Fructose and mannose metabolism [PATH:ko00051]	manC cpsB mannose-1-phosphate guanylyltransferase [EC:2.7.7.13]	69	77	55	133	109	143
Genetic Information Processing	Replication and repair	03420 Nucleotide excision repair [PATH:ko03420]	uvrC excinuclease ABC subunit C	94	74	74	123	118	109
Metabolism	Amino acid metabolism	00250 Alanine aspartate and glutamate metabolism [PATH:ko00250]	E1.4.1.3 glutamate dehydrogenase (NAD(P)+) [EC:1.4.1.3]	133	159	43	121	40	97
Metabolism	Amino acid metabolism	00260 Glycine serine and threonine metabolism [PATH:ko00260]	gcvPB glycine dehydrogenase subunit 2 [EC:1.4.4.2]	113	121	82	79	74	120
Metabolism	Amino acid metabolism	00480 Glutathione metabolism [PATH:ko00480]	CARP pepA leucyl aminopeptidase [EC:3.4.11.1]	102	97	164	74	110	98
Metabolism	Amino acid metabolism	00310 Lysine degradation [PATH:ko00310]	E5.4.3.2 kamA lysine 23-aminomutase [EC:5.4.3.2]	83	86	121	92	95	137
Metabolism	Amino acid metabolism	00400 Phenylalanine tyrosine and tryptophan biosynthesis [PATH:ko00400]	aroA 3-phosphoshikimate 1-carboxyvinyltransferase [EC:2.5.1.19]	82	115	113	95	140	70
Metabolism	Carbohydrate metabolism	00030 Pentose phosphate pathway [PATH:ko00030]	rpiB ribose 5-phosphate isomerase B [EC:5.3.1.6]	78	91	86	112	101	127
Human Diseases	citrate cycle	00020 Citrate cycle (TCA cycle) [PATH:ko00020]	E4.2.1.2B fumC fumarate hydratase class II [EC:4.2.1.2]	124	115	195	83	86	70
Cellular Processes	Cell motility	02030 Bacterial chemotaxis [PATH:ko02030]	fliG flagellar motor switch protein FliG	117	89	180	142	65	85
Metabolism	Amino acid metabolism	00330 Arginine and proline metabolism [PATH:ko00330]	E2.6.1.13 rocD ornithine--oxo-acid transaminase [EC:2.6.1.13]	90	70	70	59	78	178
Cellular Processes	Cell growth and death	04112 Cell cycle - Caulobacter [PATH:ko04112]	murG UDP-N-acetylglucosamine--N-acetylmuramyl-(pentapeptide) pyrophosphoryl-undecaprenol N-acetylglucosamine transferase [EC:2.4.1.227]	67	79	70	92	106	147

Genetic Information Processing	Replication and repair	03440 Homologous recombination [PATH:ko03440]	recR recombination protein RecR	88	64	70	84	127	116
Metabolism	Metabolism of cofactors and vitamins	00760 Nicotinate and nicotinamide metabolism [PATH:ko00760]	punA purine-nucleoside phosphorylase [EC:2.4.2.1]	74	71	51	59	100	171
Metabolism	citrate cycle	00020 Citrate cycle (TCA cycle) [PATH:ko00020]	korD 2-oxoglutarate ferredoxin oxidoreductase subunit delta [EC:1.2.7.3]	91	78	172	101	100	104
Cellular Processes	Cell motility	02030 Bacterial chemotaxis [PATH:ko02030]	cheW purine-binding chemotaxis protein CheW	101	75	168	117	90	97
Environmental Information Processing	Energy metabolism	00190 Oxidative phosphorylation [PATH:ko00190]	cydA cytochrome d ubiquinol oxidase subunit I [EC:1.10.3.-]	125	78	66	123	83	90
Metabolism	Amino acid metabolism	00330 Arginine and proline metabolism [PATH:ko00330]	proB glutamate 5-kinase [EC:2.7.2.11]	106	95	129	112	100	77
Metabolism	Carbohydrate metabolism	00010 Glycolysis / Gluconeogenesis [PATH:ko00010]	ACSS acs acetyl-CoA synthetase [EC:6.2.1.1]	67	86	66	120	72	158
Metabolism	Carbohydrate metabolism	00040 Pentose and glucuronate interconversions [PATH:ko00040]	uxaC glucuronate isomerase [EC:5.3.1.12]	76	90	55	159	64	133
Metabolism	Amino acid metabolism	00250 Alanine aspartate and glutamate metabolism [PATH:ko00250]	yhdR aspartate aminotransferase [EC:2.6.1.1]	78	119	74	78	94	121
Metabolism	Metabolism of cofactors and vitamins	00770 Pantothenate and CoA biosynthesis [PATH:ko00770]	panD aspartate 1-decarboxylase [EC:4.1.1.11]	91	79	82	57	125	106
Metabolism	Nucleotide metabolism	00240 Pyrimidine metabolism [PATH:ko00240]	E3.5.4.13 dcd dCTP deaminase [EC:3.5.4.13]	123	90	121	67	100	79
Metabolism	Amino acid metabolism	00300 Lysine biosynthesis [PATH:ko00300]	murF UDP-N-acetylmuramoyl-tripeptide--D-alanyl-D-alanine ligase [EC:6.3.2.10]	81	83	55	79	110	121
Metabolism	Carbohydrate metabolism	00520 Amino sugar and nucleotide sugar metabolism [PATH:ko00520]	wbpO UDP-N-acetyl-D-galactosamine dehydrogenase [EC:1.1.1.-]	121	101	109	87	93	73

Metabolism	Amino acid metabolism	00260 Glycine serine and threonine metabolism [PATH:ko00260]	trpA tryptophan synthase alpha chain [EC:4.2.1.20]	94	60	105	92	145	66
Metabolism	Metabolism of terpenoids and polyketides	00908 Zeatin biosynthesis [PATH:ko00908]	miaA TRIT1 tRNA dimethylallyltransferase [EC:2.5.1.75]	74	90	43	108	109	104
Environmental Information Processing	Energy metabolism	00190 Oxidative phosphorylation [PATH:ko00190]	UQCRFS1 RIP1 petA ubiquinol-cytochrome c reductase iron-sulfur subunit [EC:1.10.2.2]	120	74	117	74	115	65
Metabolism	Energy metabolism	00920 Sulfur metabolism [PATH:ko00920]	aprB adenylylsulfate reductase subunit B [EC:1.8.99.2]	108	106	70	99	64	105
Metabolism	Carbohydrate metabolism	00030 Pentose phosphate pathway [PATH:ko00030]	deoB phosphopentomutase [EC:5.4.2.7]	108	129	74	78	104	52
Metabolism	Amino acid metabolism	00340 Histidine metabolism [PATH:ko00340]	hutH HAL histidine ammonia-lyase [EC:4.3.1.3]	78	71	55	76	98	132
Metabolism	Metabolism of cofactors and vitamins	00740 Riboflavin metabolism [PATH:ko00740]	ribH RIB4 67-dimethyl-8-ribityllumazine synthase [EC:2.5.1.78]	58	39	59	95	106	153
Metabolism	Nucleotide metabolism	00230 Purine metabolism [PATH:ko00230]	hprT hpt HPRT1 hypoxanthine phosphoribosyltransferase [EC:2.4.2.8]	87	109	117	64	108	79
Metabolism	citrate cycle	00020 Citrate cycle (TCA cycle) [PATH:ko00020]	PC pyc pyruvate carboxylase [EC:6.4.1.1]	102	138	105	91	55	84
Genetic Information Processing	Replication and repair	03440 Homologous recombination [PATH:ko03440]	priA primosomal protein N' (replication factor Y) (superfamily II helicase) [EC:3.6.4.-]	75	84	78	92	80	126
Metabolism	Carbohydrate metabolism	00500 Starch and sucrose metabolism [PATH:ko00500]	E3.2.1.4 endoglucanase [EC:3.2.1.4]	70	123	86	94	91	86
Environmental Information Processing	Signal transduction	02020 Two-component system [PATH:ko02020]	csrA carbon storage regulator	104	125	226	101	59	57
Metabolism	Metabolism of other amino acids	00440 Phosphonate and phosphinate metabolism [PATH:ko00440]	E4.1.1.82 phosphonopyruvate decarboxylase [EC:4.1.1.82]	85	181	43	60	56	95
Metabolism	Glycan biosynthesis and metabolism	00540 Lipopolysaccharide biosynthesis [PATH:ko00540]	kdsB 3-deoxy-manno-octulosonate cytidylyltransferase (CMP-KDO synthetase) [EC:2.7.7.38]	44	51	55	78	112	150

Cellular Processes	Cell motility	02030 Bacterial chemotaxis [PATH:ko02030]	cheB two-component system chemotaxis family response regulator CheB [EC:3.1.1.61]	86	83	129	101	67	107
Metabolism	Nucleotide metabolism	00240 Pyrimidine metabolism [PATH:ko00240]	E3.6.1.23 dut dUTP pyrophosphatase [EC:3.6.1.23]	65	72	101	88	102	107
Cellular Processes	Cell motility	02040 Flagellar assembly [PATH:ko02040]	fliD flagellar hook-associated protein 2	91	120	101	105	67	74
Metabolism	Amino acid metabolism	00300 Lysine biosynthesis [PATH:ko00300]	argD acetylornithine/N-succinyldiaminopimelate aminotransferase [EC:2.6.1.11 2.6.1.17]	79	91	105	56	95	104
Metabolism	Carbohydrate metabolism	00040 Pentose and glucuronate interconversions [PATH:ko00040]	E2.7.1.17 xylulokinase [EC:2.7.1.17]	80	84	62	90	78	112
Cellular Processes	Cell motility	02040 Flagellar assembly [PATH:ko02040]	flhA flagellar biosynthesis protein FlhA	87	102	86	137	63	78
Metabolism	Energy metabolism	00190 Oxidative phosphorylation [PATH:ko00190]	nuoN NADH-quinone oxidoreductase subunit N [EC:1.6.5.3]	93	66	125	81	92	88
Metabolism	Amino acid metabolism	00280 Valine leucine and isoleucine degradation [PATH:ko00280]	E1.4.1.9 leucine dehydrogenase [EC:1.4.1.9]	27	17	168	217	111	84
Metabolism	Amino acid metabolism	00270 Cysteine and methionine metabolism [PATH:ko00270]	metA homoserine O-succinyltransferase [EC:2.3.1.46]	62	90	70	92	95	103
Genetic Information Processing	Replication and repair	03430 Mismatch repair [PATH:ko03430]	mutS2 DNA mismatch repair protein MutS2	58	95	82	128	78	99
Metabolism	Biosynthesis of other secondary metabolites	00521 Streptomycin biosynthesis [PATH:ko00521]	rfbC dTDP-4-dehydrorhamnose 35-epimerase [EC:5.1.3.13]	69	67	47	87	109	100
Metabolism	Energy metabolism	00910 Nitrogen metabolism [PATH:ko00910]	E1.13.12.16 nitronate monooxygenase [EC:1.13.12.16]	103	72	121	60	116	57
Metabolism	Energy metabolism	00190 Oxidative phosphorylation [PATH:ko00190]	nuoH NADH-quinone oxidoreductase subunit H [EC:1.6.5.3]	113	72	117	67	96	64
Cellular Processes	Cell growth and death	04112 Cell cycle - Caulobacter [PATH:ko04112]	rseP regulator of sigma E protease [EC:3.4.24.-]	65	69	90	84	97	109

Environmental Information Processing	Energy metabolism	00020 Citrate cycle (TCA cycle) [PATH:ko00020]	frdB fumarate reductase iron-sulfur subunit [EC:1.3.99.1]	104	83	183	114	60	65
Metabolism	Energy metabolism	00190 Oxidative phosphorylation [PATH:ko00190]	ATPF1D atpH F-type H+-transporting ATPase subunit delta [EC:3.6.3.14]	114	98	82	59	96	54
Metabolism	Amino acid metabolism	00340 Histidine metabolism [PATH:ko00340]	E2.1.1.- [EC:2.1.1.-]	68	68	70	86	116	87
Metabolism	Carbohydrate metabolism	00620 Pyruvate metabolism [PATH:ko00620]	accA acetyl-CoA carboxylase carboxyl transferase subunit alpha [EC:6.4.1.2]	84	89	101	76	107	67
Cellular Processes	Cell motility	02030 Bacterial chemotaxis [PATH:ko02030]	motA chemotaxis protein MotA	110	88	113	122	57	64
Metabolism	Amino acid metabolism	00340 Histidine metabolism [PATH:ko00340]	hisF cyclase [EC:4.1.3.-]	87	69	113	78	95	81
Metabolism	Amino acid metabolism	00250 Alanine aspartate and glutamate metabolism [PATH:ko00250]	E3.5.1.1 ansA ansB L-asparaginase [EC:3.5.1.1]	59	90	23	87	96	102
Metabolism	Nucleotide metabolism	00230 Purine metabolism [PATH:ko00230]	purE 5-(carboxyamino)imidazole ribonucleotide mutase [EC:5.4.99.18]	90	47	90	79	96	90
Cellular Processes	Cell motility	02030 Bacterial chemotaxis [PATH:ko02030]	fliNY fliN flagellar motor switch protein FliN/FliY	97	123	144	99	49	61
Metabolism	Amino acid metabolism	00270 Cysteine and methionine metabolism [PATH:ko00270]	tyrB aromatic-amino-acid transaminase [EC:2.6.1.57]	97	57	62	40	151	42
Metabolism	Metabolism of cofactors and vitamins	00860 Porphyrin and chlorophyll metabolism [PATH:ko00860]	hemE UROD uroporphyrinogen decarboxylase [EC:4.1.1.37]	127	58	82	61	83	65
Metabolism	Energy metabolism	00190 Oxidative phosphorylation [PATH:ko00190]	nuoA NADH-quinone oxidoreductase subunit A [EC:1.6.5.3]	106	82	78	77	88	59
Metabolism	Amino acid metabolism	00310 Lysine degradation [PATH:ko00310]	E3.4.-.- [EC:3.4.-.-]	76	141	101	91	67	61
Genetic Information Processing	Replication and repair	03410 Base excision repair [PATH:ko03410]	UNG UDG uracil-DNA glycosylase [EC:3.2.2.27]	77	94	62	66	100	77

Metabolism	Nucleotide metabolism	00240 Pyrimidine metabolism [PATH:ko00240]	E2.4.2.3 udp uridine phosphorylase [EC:2.4.2.3]	69	64	78	58	103	102
Metabolism	Lipid metabolism	00071 Fatty acid metabolism [PATH:ko00071]	aas acyl-[acyl-carrier-protein]-phospholipid O-acyltransferase / long-chain-fatty-acid--[acyl-carrier-protein] ligase [EC:2.3.1.40 6.2.1.20]	82	80	117	90	102	53
Environmental Information Processing	Membrane transport	02010 ABC transporters [PATH:ko02010]	mglA methyl-galactoside transport system ATP-binding protein [EC:3.6.3.17]	106	64	117	88	74	66
Metabolism	Nucleotide metabolism	00230 Purine metabolism [PATH:ko00230]	ade adenine deaminase [EC:3.5.4.2]	111	80	59	75	63	78
Environmental Information Processing	Membrane transport	02060 Phosphotransferase system (PTS) [PATH:ko02060]	PTS-Ula-EIIB ulaB sgaB PTS system ascorbate-specific IIB component [EC:2.7.1.69]	66	112	51	37	158	25
Environmental Information Processing	Membrane transport	02010 ABC transporters [PATH:ko02010]	ABC.LPT.A lolD lipoprotein-releasing system ATP-binding protein [EC:3.6.3.-]	67	66	82	92	81	98
Metabolism	Metabolism of terpenoids and polyketides	00900 Terpenoid backbone biosynthesis [PATH:ko00900]	ispF 2-C-methyl-D-erythritol 24-cyclodiphosphate synthase [EC:4.6.1.12]	88	88	121	83	85	54
Metabolism	Carbohydrate metabolism	00020 Citrate cycle (TCA cycle) [PATH:ko00020]	sucD succinyl-CoA synthetase alpha subunit [EC:6.2.1.5]	93	59	62	84	70	91
Metabolism	Glycan biosynthesis and metabolism	00540 Lipopolysaccharide biosynthesis [PATH:ko00540]	gmhD rfaD ADP-L-glycero-D-manno-heptose 6-epimerase [EC:5.1.3.20]	58	45	59	35	125	107
Metabolism	Amino acid metabolism	00400 Phenylalanine tyrosine and tryptophan biosynthesis [PATH:ko00400]	pheB chorismate mutase [EC:5.4.99.5]	122	152	66	49	51	38
Metabolism	Amino acid metabolism	00250 Alanine aspartate and glutamate metabolism [PATH:ko00250]	asnB ASNS asparagine synthase (glutamine-hydrolysing) [EC:6.3.5.4]	69	91	90	69	76	91
Genetic Information Processing	Folding sorting and degradation	03018 RNA degradation [PATH:ko03018]	rne ribonuclease E [EC:3.1.26.12]	94	63	98	52	123	41
Metabolism	Glycan biosynthesis and metabolism	00550 Peptidoglycan biosynthesis [PATH:ko00550]	mrdA penicillin-binding protein 2	84	52	78	78	82	90

Metabolism	Glycan biosynthesis and metabolism	00550 Peptidoglycan biosynthesis [PATH:ko00550]	E3.6.1.27 bacA undecaprenyl-diphosphatase [EC:3.6.1.27]	69	61	55	74	88	97
Metabolism	Metabolism of cofactors and vitamins	00790 Folate biosynthesis [PATH:ko00790]	folC dihydrofolate synthase / folylpolyglutamate synthase [EC:6.3.2.12 6.3.2.17]	64	52	43	68	93	107
Environmental Information Processing	Membrane transport	02010 ABC transporters [PATH:ko02010]	livM branched-chain amino acid transport system permease protein	95	75	144	110	67	45
Genetic Information Processing	Replication and repair	03430 Mismatch repair [PATH:ko03430]	xseA exodeoxyribonuclease VII large subunit [EC:3.1.11.6]	85	71	82	104	67	72
Metabolism	Energy metabolism	00190 Oxidative phosphorylation [PATH:ko00190]	nuoM NADH-quinone oxidoreductase subunit M [EC:1.6.5.3]	89	48	117	55	81	87
Metabolism	Amino acid metabolism	00330 Arginine and proline metabolism [PATH:ko00330]	pip proline iminopeptidase [EC:3.4.11.5]	63	135	39	80	63	72
Metabolism	Carbohydrate metabolism	00010 Glycolysis / Gluconeogenesis [PATH:ko00010]	ALDO fructose-bisphosphate aldolase class I [EC:4.1.2.13]	66	96	31	56	60	112
Environmental Information Processing	Membrane transport	02010 ABC transporters [PATH:ko02010]	afuB fbpB iron(III) transport system permease protein	91	92	62	83	54	73
Metabolism	citrate cycle	00020 Citrate cycle (TCA cycle) [PATH:ko00020]	E6.4.1.1A pycA pyruvate carboxylase subunit A [EC:6.4.1.1]	98	71	180	113	48	47
Metabolism	Energy metabolism	00680 Methane metabolism [PATH:ko00680]	nhaC Na+:H+ antiporter NhaC family	62	72	31	84	56	117
Environmental Information Processing	Membrane transport	02010 ABC transporters [PATH:ko02010]	ABC-2.LPSE.A lipopolysaccharide transport system ATP-binding protein	97	80	86	66	66	65
Metabolism	Amino acid metabolism	00330 Arginine and proline metabolism [PATH:ko00330]	E2.6.1.11 argD acetylornithine aminotransferase [EC:2.6.1.11]	71	72	66	89	67	84
Metabolism	Lipid metabolism	00061 Fatty acid biosynthesis [PATH:ko00061]	fabK enoyl-[acyl-carrier protein] reductase II [EC:1.3.1.-]	68	110	51	58	81	69
Environmental Information Processing	Membrane transport	02010 ABC transporters [PATH:ko02010]	livH branched-chain amino acid transport system permease protein	93	103	94	97	45	55
Genetic Information Processing	Replication and repair	03410 Base excision repair [PATH:ko03410]	NTH endonuclease III [EC:4.2.99.18]	65	83	47	74	87	73

Cellular Processes	Cell motility	02040 Flagellar assembly [PATH:ko02040]	fliS flagellar protein FliS	82	123	183	81	51	39
Genetic Information Processing	Replication and repair	03030 DNA replication [PATH:ko03030]	DPO3E dnaQ DNA polymerase III subunit epsilon [EC:2.7.7.7]	37	58	43	74	102	102
Cellular Processes	Cell motility	02040 Flagellar assembly [PATH:ko02040]	flgC flagellar basal-body rod protein FlgC	84	80	140	116	47	55
Environmental Information Processing	Membrane transport	02010 ABC transporters [PATH:ko02010]	ABC.MET.A metN D-methionine transport system ATP-binding protein	90	77	74	56	88	51
Metabolism	Lipid metabolism	00564 Glycerophospholipid metabolism [PATH:ko00564]	glpA glpD glycerol-3-phosphate dehydrogenase [EC:1.1.5.3]	63	91	47	86	50	98
Metabolism	Carbohydrate metabolism	00520 Amino sugar and nucleotide sugar metabolism [PATH:ko00520]	murB UDP-N-acetylmuramate dehydrogenase [EC:1.1.1.158]	63	83	66	66	63	97
Metabolism	Amino acid metabolism	00400 Phenylalanine tyrosine and tryptophan biosynthesis [PATH:ko00400]	trpE anthranilate synthase component I [EC:4.1.3.27]	79	75	117	75	90	41
Metabolism	Nucleotide metabolism	00230 Purine metabolism [PATH:ko00230]	dgt dGTPase [EC:3.1.5.1]	63	70	98	67	70	86
Metabolism	Metabolism of cofactors and vitamins	00760 Nicotinate and nicotinamide metabolism [PATH:ko00760]	ppnK NADK NAD+ kinase [EC:2.7.1.23]	65	67	31	57	100	71
Metabolism	Metabolism of cofactors and vitamins	00750 Vitamin B6 metabolism [PATH:ko00750]	pdxA 4-hydroxythreonine-4-phosphate dehydrogenase [EC:1.1.1.262]	53	47	86	84	84	88
Metabolism	Energy metabolism	00910 Nitrogen metabolism [PATH:ko00910]	cynT can carbonic anhydrase [EC:4.2.1.1]	78	137	117	69	50	43
Cellular Processes	Cell motility	02040 Flagellar assembly [PATH:ko02040]	flgB flagellar basal-body rod protein FlgB	91	49	140	111	51	57
Environmental Information Processing	Signal transduction	02020 Two-component system [PATH:ko02020]	citF citrate lyase subunit alpha / citrate CoA-transferase [EC:4.1.3.6 2.8.3.10]	66	76	35	71	68	82
Metabolism	Nucleotide metabolism	00240 Pyrimidine metabolism [PATH:ko00240]	E2.7.4.9 tmk dTMP kinase [EC:2.7.4.9]	81	81	74	37	94	48
Metabolism	Carbohydrate metabolism	00010 Glycolysis / Gluconeogenesis [PATH:ko00010]	FBP fbp fructose-16-bisphosphatase I [EC:3.1.3.11]	74	68	105	66	79	57

Metabolism	Carbohydrate metabolism	00640 Propanoate metabolism [PATH:ko00640]	E6.2.1.17 prpE propionyl-CoA synthetase [EC:6.2.1.17]	44	199	27	71	58	35
Metabolism	Carbohydrate metabolism	00650 Butanoate metabolism [PATH:ko00650]	phbC phaC polyhydroxyalkanoate synthase [EC:2.3.1.-]	122	85	117	26	53	39
Metabolism	Metabolism of cofactors and vitamins	00760 Nicotinate and nicotinamide metabolism [PATH:ko00760]	E6.3.5.1 NADSYN1 QNS1 nadE NAD+ synthase (glutamine-hydrolysing) [EC:6.3.5.1]	46	60	62	84	76	87
Cellular Processes	Membrane transport	02010 ABC transporters [PATH:ko02010]	malE maltose/maltodextrin transport system substrate-binding protein	20	23	20	94	74	142
Metabolism	Metabolism of cofactors and vitamins	00860 Porphyrin and chlorophyll metabolism [PATH:ko00860]	hemD UROS uroporphyrinogen-III synthase [EC:4.2.1.75]	23	24	27	66	84	140
Human Diseases	nitrogen metabolism	00910 Nitrogen metabolism [PATH:ko00910]	nrfA nitrite reductase cytochrome c-552 [EC:1.7.2.2]	76	87	27	57	56	77
Organismal Systems	Amino acid metabolism	04974 Protein digestion and absorption [PATH:ko04974]	DPP4 dipeptidyl-peptidase 4 [EC:3.4.14.5]	34	29	27	56	70	143
Environmental Information Processing	Membrane transport	02010 ABC transporters [PATH:ko02010]	ABCC-BAC ATP-binding cassette subfamily C bacterial	74	89	62	66	51	70
Metabolism	Metabolism of terpenoids and polyketides	00281 Geraniol degradation [PATH:ko00281]	E2.8.3.- [EC:2.8.3.-]	62	81	90	48	80	64
Metabolism	Carbohydrate metabolism	00630 Glyoxylate and dicarboxylate metabolism [PATH:ko00630]	E3.1.3.18 gph phosphoglycolate phosphatase [EC:3.1.3.18]	58	85	101	67	81	51
Metabolism	Carbohydrate metabolism	00010 Glycolysis / Gluconeogenesis [PATH:ko00010]	porB pyruvate ferredoxin oxidoreductase beta subunit [EC:1.2.7.1]	41	34	27	129	74	86
Metabolism	Amino acid metabolism	00330 Arginine and proline metabolism [PATH:ko00330]	prdA D-proline reductase (dithiol) PrdA [EC:1.21.4.1]	63	82	43	46	63	87
Metabolism	Amino acid metabolism	00330 Arginine and proline metabolism [PATH:ko00330]	E3.5.3.6 arcA arginine deiminase [EC:3.5.3.6]	59	78	62	84	51	81
Metabolism	Amino acid metabolism	00270 Cysteine and methionine metabolism [PATH:ko00270]	E4.4.1.11 methionine-gamma-lyase [EC:4.4.1.11]	55	85	59	49	66	85
Metabolism	Amino acid metabolism	00340 Histidine metabolism [PATH:ko00340]	E3.5.2.7 hutI imidazolonepropionase [EC:3.5.2.7]	36	23	43	55	84	125

Metabolism	Carbohydrate metabolism	00052 Galactose metabolism [PATH:ko00052]	E3.2.1.22B galA rafA alpha-galactosidase [EC:3.2.1.22]	57	53	43	70	86	71
Metabolism	Nucleotide metabolism	00230 Purine metabolism [PATH:ko00230]	purK 5-(carboxyamino)imidazole ribonucleotide synthase [EC:6.3.4.18]	80	51	82	7	94	70
Metabolism	Carbohydrate metabolism	00520 Amino sugar and nucleotide sugar metabolism [PATH:ko00520]	E3.2.1.52 nagZ beta-N-acetylhexosaminidase [EC:3.2.1.52]	63	92	59	33	91	52
Metabolism	Energy metabolism	00190 Oxidative phosphorylation [PATH:ko00190]	ATPVI ntpI V-type H+-transporting ATPase subunit I [EC:3.6.3.14]	74	84	51	90	37	70
Metabolism	Amino acid metabolism	00270 Cysteine and methionine metabolism [PATH:ko00270]	E2.3.1.31 metX homoserine O-acetyltransferase [EC:2.3.1.31]	105	65	47	63	64	37
Environmental Information Processing	Carbohydrate metabolism	00620 Pyruvate metabolism [PATH:ko00620]	E4.4.1.5 GLO1 gloA lactoylglutathione lyase [EC:4.4.1.5]	46	66	78	42	118	49
Metabolism	Carbohydrate metabolism	00650 Butanoate metabolism [PATH:ko00650]	E2.7.2.7 buk butyrate kinase [EC:2.7.2.7]	62	65	20	79	48	93
Metabolism	Glycan biosynthesis and metabolism	00510 N-Glycan biosynthesis [PATH:ko00510]	DPM1 dolichol-phosphate mannosyltransferase [EC:2.4.1.83]	52	59	27	69	67	91
Metabolism	Carbohydrate metabolism	00051 Fructose and mannose metabolism [PATH:ko00051]	E1.1.1.271 fcl GDP-L-fucose synthase [EC:1.1.1.271]	56	54	39	68	74	80
Metabolism	Amino acid metabolism	00360 Phenylalanine metabolism [PATH:ko00360]	E4.1.3.39 mhpE 4-hydroxy 2-oxovalerate aldolase [EC:4.1.3.39]	84	68	152	64	17	85
Cellular Processes	Cell growth and death	04112 Cell cycle - Caulobacter [PATH:ko04112]	flp pilA pilus assembly protein Flp/PilA	111	72	363	1	8	65
Metabolism	Carbohydrate metabolism	00051 Fructose and mannose metabolism [PATH:ko00051]	mtlD mannitol-1-phosphate 5-dehydrogenase [EC:1.1.1.17]	27	98	39	36	118	53
Environmental Information Processing	Membrane transport	02010 ABC transporters [PATH:ko02010]	potD spermidine/putrescine transport system substrate-binding protein	60	69	59	94	52	69
Genetic Information Processing	Replication and repair	03440 Homologous recombination [PATH:ko03440]	recD exodeoxyribonuclease V alpha subunit [EC:3.1.11.5]	72	68	82	57	69	57
Human Diseases	Nucleotide metabolism	00230 Purine metabolism [PATH:ko00230]	E3.5.4.4 ADA add adenosine deaminase [EC:3.5.4.4]	91	95	59	57	47	46

Metabolism	Amino acid metabolism	00260 Glycine serine and threonine metabolism [PATH:ko00260]	pssA CHO1 phosphatidylserine synthase [EC:2.7.8.8]	81	49	105	71	59	58
Environmental Information Processing	Membrane transport	02010 ABC transporters [PATH:ko02010]	afuC fbpC iron(III) transport system ATP-binding protein [EC:3.6.3.30]	69	83	70	66	59	56
Metabolism	Glycan biosynthesis and metabolism	00511 Other glycan degradation [PATH:ko00511]	FUCA alpha-L-fucosidase [EC:3.2.1.51]	46	44	31	26	72	121
Metabolism	Carbohydrate metabolism	00620 Pyruvate metabolism [PATH:ko00620]	pta phosphate acetyltransferase [EC:2.3.1.8]	65	58	74	61	74	60
Genetic Information Processing	Transcription	03020 RNA polymerase [PATH:ko03020]	rpoZ DNA-directed RNA polymerase subunit omega [EC:2.7.7.6]	63	42	59	52	87	67
Metabolism	Carbohydrate metabolism	00020 Citrate cycle (TCA cycle) [PATH:ko00020]	E4.2.1.2AB fumB fumarate hydratase subunit beta [EC:4.2.1.2]	64	56	47	100	44	73
Metabolism	Amino acid metabolism	00340 Histidine metabolism [PATH:ko00340]	hisE phosphoribosyl-ATP pyrophosphohydrolase [EC:3.6.1.31]	125	56	47	15	77	20
Metabolism	Carbohydrate metabolism	00630 Glyoxylate and dicarboxylate metabolism [PATH:ko00630]	purU formyltetrahydrofolate deformylase [EC:3.5.1.10]	59	55	62	70	85	48
Metabolism	Metabolism of terpenoids and polyketides	00900 Terpenoid backbone biosynthesis [PATH:ko00900]	ispB octaprenyl-diphosphate synthase [EC:2.5.1.90]	58	41	59	53	94	60
Environmental Information Processing	Membrane transport	02010 ABC transporters [PATH:ko02010]	rbsC ribose transport system permease protein	71	106	74	45	64	36
Genetic Information Processing	Replication and repair	03030 DNA replication [PATH:ko03030]	rnhB ribonuclease HII [EC:3.1.26.4]	60	61	23	63	70	68
Metabolism	Amino acid metabolism	00260 Glycine serine and threonine metabolism [PATH:ko00260]	gcvPA glycine dehydrogenase subunit 1 [EC:1.4.4.2]	58	82	55	60	47	77
Metabolism	Metabolism of cofactors and vitamins	00760 Nicotinate and nicotinamide metabolism [PATH:ko00760]	nadA quinolinate synthase [EC:2.5.1.72]	29	36	66	102	81	75
Metabolism	Lipid metabolism	01040 Biosynthesis of unsaturated fatty acids [PATH:ko01040]	yciA acyl-CoA thioesterase YciA [EC:3.1.2.-]	74	129	199	70	33	15
Genetic Information Processing	Translation	00970 Aminoacyl-tRNA biosynthesis [PATH:ko00970]	gatC aspartyl-tRNA(Asn)/glutamyl-tRNA (Gln) amidotransferase subunit C [EC:6.3.5.6 6.3.5.7]	61	71	86	71	73	40

Metabolism	Metabolism of cofactors and vitamins	00750 Vitamin B6 metabolism [PATH:ko00750]	pdxK pdxY pyridoxine kinase [EC:2.7.1.35]	63	67	59	48	64	67
Metabolism	Lipid metabolism	00564 Glycerophospholipid metabolism [PATH:ko00564]	psd PISD phosphatidylserine decarboxylase [EC:4.1.1.65]	46	48	23	50	103	62
Metabolism	Energy metabolism	00680 Methane metabolism [PATH:ko00680]	nhaA Na+:H+ antiporter NhaA family	67	71	74	83	54	47
Metabolism	Amino acid metabolism	00340 Histidine metabolism [PATH:ko00340]	E4.2.1.19 hisB imidazoleglycerol-phosphate dehydratase [EC:4.2.1.19]	71	82	74	49	67	40
Metabolism	Carbohydrate metabolism	00020 Citrate cycle (TCA cycle) [PATH:ko00020]	E4.2.1.2AA fumA fumarate hydratase subunit alpha [EC:4.2.1.2]	56	49	86	95	42	76
Metabolism	Nucleotide metabolism	00240 Pyrimidine metabolism [PATH:ko00240]	comEB dCMP deaminase [EC:3.5.4.12]	54	60	27	57	54	89
Metabolism	Carbohydrate metabolism	00051 Fructose and mannose metabolism [PATH:ko00051]	E5.3.1.8 manA mannose-6-phosphate isomerase [EC:5.3.1.8]	57	47	43	40	77	77
Environmental Information Processing	Signal transduction	04070 Phosphatidylinositol signaling system [PATH:ko04070]	E2.7.7.41 CDS1 CDS2 cdsA phosphatidate cytidylyltransferase [EC:2.7.7.41]	56	60	43	72	56	72
Metabolism	Carbohydrate metabolism	00520 Amino sugar and nucleotide sugar metabolism [PATH:ko00520]	UAP1 UDP-N-acetylglucosamine pyrophosphorylase [EC:2.7.7.23]	47	152	101	60	32	44
Metabolism	Lipid metabolism	00561 Glycerolipid metabolism [PATH:ko00561]	plsY glycerol-3-phosphate acyltransferase PlsY [EC:2.3.1.15]	89	59	101	54	54	41
Metabolism	Glycan biosynthesis and metabolism	00540 Lipopolysaccharide biosynthesis [PATH:ko00540]	gmhA lpcA D-sedoheptulose 7-phosphate isomerase [EC:5.3.1.28]	69	35	27	53	90	50
Metabolism	Carbohydrate metabolism	00040 Pentose and glucuronate interconversions [PATH:ko00040]	uxaB tagaturonate reductase [EC:1.1.1.58]	58	27	27	101	39	93
Metabolism	Glycan biosynthesis and metabolism	00540 Lipopolysaccharide biosynthesis [PATH:ko00540]	kdsC 3-deoxy-D-manno-octulosonate 8-phosphate phosphatase (KDO 8-P phosphatase) [EC:3.1.3.45]	76	66	43	38	71	46
Metabolism	Metabolism of terpenoids and polyketides	00900 Terpenoid backbone biosynthesis [PATH:ko00900]	ispD 2-C-methyl-D-erythritol 4-phosphate cytidylyltransferase [EC:2.7.7.60]	56	54	62	59	62	70

Genetic Information Processing	Replication and repair	03440 Homologous recombination [PATH:ko03440]	recF DNA replication and repair protein RecF	43	51	51	60	59	91
Metabolism	Energy metabolism	00190 Oxidative phosphorylation [PATH:ko00190]	nuoB NADH-quinone oxidoreductase subunit B [EC:1.6.5.3]	72	53	117	54	69	39
Metabolism	Carbohydrate metabolism	00052 Galactose metabolism [PATH:ko00052]	E3.2.1.26 sacA beta-fructofuranosidase [EC:3.2.1.26]	78	106	35	35	35	57
Metabolism	Energy metabolism	00910 Nitrogen metabolism [PATH:ko00910]	napG ferredoxin-type protein NapG	50	72	86	103	23	76
Genetic Information Processing	Replication and repair	03030 DNA replication [PATH:ko03030]	E3.1.26.4A RNASEH1 rnhA ribonuclease HI [EC:3.1.26.4]	59	48	55	67	60	68
Environmental Information Processing	Signal transduction	02020 Two-component system [PATH:ko02020]	SIG54 rpoN RNA polymerase sigma-54 factor	36	59	51	72	64	77
Metabolism	Amino acid metabolism	00310 Lysine degradation [PATH:ko00310]	DLST sucB 2-oxoglutarate dehydrogenase E2 component (dihydrolipoamide succinyltransferase) [EC:2.3.1.61]	91	53	74	13	79	37
Environmental Information Processing	Membrane Transport	02010 ABC transporters [PATH:ko02010]	cmpD bicarbonate transport system ATP-binding protein [EC:3.6.3.-]	66	40	101	97	50	50
Metabolism	Metabolism of cofactors and vitamins	00760 Nicotinate and nicotinamide metabolism [PATH:ko00760]	nadE NAD+ synthase [EC:6.3.1.5]	60	67	31	46	76	50
Metabolism	Xenobiotics biodegradation and metabolism	00791 Atrazine degradation [PATH:ko00791]	atzB hydroxyatrazine ethylaminohydrolase [EC:3.5.99.3]	45	96	35	67	62	50
Genetic Information Processing	Translation	00970 Aminoacyl-tRNA biosynthesis [PATH:ko00970]	lysK lysyl-tRNA synthetase class I [EC:6.1.1.6]	63	38	94	77	43	72
Metabolism	Metabolism of cofactors and vitamins	00670 One carbon pool by folate [PATH:ko00670]	E2.1.1.148 thyX thy1 thymidylate synthase (FAD) [EC:2.1.1.148]	62	59	47	59	59	58
Metabolism	Amino acid metabolism	00250 Alanine aspartate and glutamate metabolism [PATH:ko00250]	E5.1.1.13 aspartate racemase [EC:5.1.1.13]	58	95	66	55	51	46
Metabolism	Metabolism of cofactors and vitamins	00860 Porphyrin and chlorophyll metabolism [PATH:ko00860]	hemC HMBS hydroxymethylbilane synthase [EC:2.5.1.61]	62	82	125	63	62	25

Metabolism	Amino acid metabolism	00250 Alanine aspartate and glutamate metabolism [PATH:ko00250]	nadB L-aspartate oxidase [EC:1.4.3.16]	39	32	55	77	66	80
Cellular Processes	Cell growth and death	04112 Cell cycle - Caulobacter [PATH:ko04112]	ftsW spoVE cell division protein FtsW	47	59	55	70	48	74
Metabolism	Nucleotide metabolism	00230 Purine metabolism [PATH:ko00230]	rdgB dITP/XTP pyrophosphatase [EC:3.6.1.19]	50	79	35	52	53	67
Metabolism	Lipid metabolism	00061 Fatty acid biosynthesis [PATH:ko00061]	fabB 3-oxoacyl-[acyl-carrier-protein] synthase I [EC:2.3.1.41]	43	42	27	50	82	68
Metabolism	Metabolism of cofactors and vitamins	00670 One carbon pool by folate [PATH:ko00670]	purN phosphoribosylglycinamide formyltransferase 1 [EC:2.1.2.2]	47	50	47	43	74	68
Environmental Information Processing	Membrane transport	02010 ABC transporters [PATH:ko02010]	ABC.GGU.P gguB putative multiple sugar transport system permease protein	75	94	51	74	26	41
Metabolism	Glycan biosynthesis and metabolism	00550 Peptidoglycan biosynthesis [PATH:ko00550]	ftsI cell division protein FtsI (penicillin-binding protein 3) [EC:2.4.1.129]	50	43	51	45	81	58
Metabolism	Amino acid metabolism	00340 Histidine metabolism [PATH:ko00340]	E5.3.1.16 hisA phosphoribosylformimino-5-aminoimidazole carboxamide ribotide isomerase [EC:5.3.1.16]	53	62	105	55	62	45
Metabolism	Metabolism of cofactors and vitamins	00860 Porphyrin and chlorophyll metabolism [PATH:ko00860]	hemA glutamyl-tRNA reductase [EC:1.2.1.70]	72	73	74	37	56	39
Metabolism	Amino acid metabolism	00270 Cysteine and methionine metabolism [PATH:ko00270]	mtnN mtn pfs S-adenosylhomocysteine/5'-methylthioadenosine nucleosidase [EC:3.2.2.9]	51	52	62	77	59	51
Metabolism	Metabolism of cofactors and vitamins	00740 Riboflavin metabolism [PATH:ko00740]	ribD diaminohydroxyphosphoribosylaminopyrimidine deaminase / 5-amino-6-(5-phosphoribosylamino)uracil reductase [EC:3.5.4.26 1.1.1.193]	31	20	74	42	88	79
Environmental Information Processing	Membrane transport	02010 ABC transporters [PATH:ko02010]	macB macrolide transport system ATP-binding/permease protein [EC:3.6.3.-]	58	70	55	38	79	31

Metabolism	Metabolism of cofactors and vitamins	00130 Ubiquinone and other terpenoid-quinone biosynthesis [PATH:ko00130]	ubiE ubiquinone/menaquinone biosynthesis methyltransferase [EC:2.1.1.163 2.1.1.201]	59	48	47	36	73	53
Metabolism	Energy metabolism	00190 Oxidative phosphorylation [PATH:ko00190]	nuoC NADH-quinone oxidoreductase subunit C [EC:1.6.5.3]	66	56	66	38	67	39
Metabolism	Amino acid metabolism	00260 Glycine serine and threonine metabolism [PATH:ko00260]	thrA bifunctional aspartokinase / homoserine dehydrogenase 1 [EC:2.7.2.4 1.1.1.3]	33	25	16	108	57	74
Metabolism	Metabolism of cofactors and vitamins	00750 Vitamin B6 metabolism [PATH:ko00750]	pdxJ pyridoxine 5-phosphate synthase [EC:2.6.99.2]	43	46	82	56	81	43
Metabolism	Carbohydrate metabolism	00620 Pyruvate metabolism [PATH:ko00620]	E4.2.3.3 mgsA methylglyoxal synthase [EC:4.2.3.3]	39	108	47	37	62	41
Metabolism	Carbohydrate metabolism	00040 Pentose and glucuronate interconversions [PATH:ko00040]	uxuB fructuronate reductase [EC:1.1.1.57]	42	41	16	63	75	58
Metabolism	Amino acid metabolism	00340 Histidine metabolism [PATH:ko00340]	hisH glutamine amidotransferase [EC:2.4.2.-]	53	62	86	54	60	42
Metabolism	Carbohydrate metabolism	00620 Pyruvate metabolism [PATH:ko00620]	ldhA D-lactate dehydrogenase [EC:1.1.1.28]	49	66	27	41	50	70
Metabolism	Metabolism of terpenoids and polyketides	00900 Terpenoid backbone biosynthesis [PATH:ko00900]	GGPS geranylgeranyl diphosphate synthase type II [EC:2.5.1.1 2.5.1.10 2.5.1.29]	38	45	51	63	45	84
Metabolism	Metabolism of cofactors and vitamins	00790 Folate biosynthesis [PATH:ko00790]	folP dihydropteroate synthase [EC:2.5.1.15]	43	40	66	58	65	60
Environmental Information Processing	Membrane transport	02010 ABC transporters [PATH:ko02010]	znuA zinc transport system substrate-binding protein	66	51	98	66	39	46
Metabolism	Metabolism of cofactors and vitamins	00780 Biotin metabolism [PATH:ko00780]	bioB biotin synthase [EC:2.8.1.6]	58	62	27	48	56	49
Metabolism	Carbohydrate metabolism	00520 Amino sugar and nucleotide sugar metabolism [PATH:ko00520]	E3.2.1.55 abfA alpha-N-arabinofuranosidase [EC:3.2.1.55]	44	57	31	53	56	63
Environmental Information Processing	Signal transduction	02020 Two-component system [PATH:ko02020]	atoA acetate CoA-transferase beta subunit [EC:2.8.3.8]	69	69	62	44	35	46

Metabolism	Nucleotide metabolism	00240 Pyrimidine metabolism [PATH:ko00240]	E2.7.1.21 tdk thymidine kinase [EC:2.7.1.21]	43	36	70	42	57	72
Metabolism	Amino acid metabolism	00400 Phenylalanine tyrosine and tryptophan biosynthesis [PATH:ko00400]	pheA chorismate mutase / prephenate dehydratase [EC:5.4.99.5 4.2.1.51]	59	43	70	43	63	43
Metabolism	Amino acid metabolism	00300 Lysine biosynthesis [PATH:ko00300]	dapF diaminopimelate epimerase [EC:5.1.1.7]	29	47	47	50	74	58
Metabolism	Glycan biosynthesis and metabolism	00540 Lipopolysaccharide biosynthesis [PATH:ko00540]	gmhC hldE waaE rfaE D-beta-D-heptose 7-phosphate kinase / D-beta-D-heptose 1-phosphate adenosyltransferase [EC:2.7.1.167 2.7.7.70]	40	39	20	49	66	65
Metabolism	Amino acid metabolism	00340 Histidine metabolism [PATH:ko00340]	hisIE phosphoribosyl-ATP pyrophosphohydrolase / phosphoribosyl-AMP cyclohydrolase [EC:3.6.1.31 3.5.4.19]	56	38	51	59	39	63
Cellular Processes	Transport and catabolism	04142 Lysosome [PATH:ko04142]	HEXA B hexosaminidase [EC:3.2.1.52]	52	58	27	45	38	67
Environmental Information Processing	Membrane transport	02010 ABC transporters [PATH:ko02010]	ftsX cell division transport system permease protein	40	42	86	52	62	50
Genetic Information Processing	Replication and repair	03440 Homologous recombination [PATH:ko03440]	ruvA holliday junction DNA helicase RuvA [EC:3.6.4.12]	42	44	70	43	58	60
Metabolism	Carbohydrate metabolism	00660 C5-Branched dibasic acid metabolism [PATH:ko00660]	E5.4.99.1 methylaspartate mutase [EC:5.4.99.1]	18	19	109	76	74	57
Environmental Information Processing	Membrane transport	03070 Bacterial secretion system [PATH:ko03070]	gspF general secretion pathway protein F	71	55	86	37	30	49
Metabolism	Amino acid metabolism	00300 Lysine biosynthesis [PATH:ko00300]	E2.6.1.83 LL-diaminopimelate aminotransferase [EC:2.6.1.83]	52	49	43	46	34	70
Environmental Information Processing	Membrane transport	02010 ABC transporters [PATH:ko02010]	ABC.FEV.P iron complex transport system permease protein	41	66	78	42	51	48
Metabolism	Metabolism of cofactors and vitamins	00740 Riboflavin metabolism [PATH:ko00740]	ribE RIB5 riboflavin synthase [EC:2.5.1.9]	30	38	35	53	54	75

Cellular Processes	Cell motility	02040 Flagellar assembly [PATH:ko02040]	flgD flagellar basal-body rod modification protein FlgD	68	39	121	44	35	44
Metabolism	Amino acid metabolism	00260 Glycine serine and threonine metabolism [PATH:ko00260]	thrH phosphoserine / homoserine phosphotransferase [EC:3.1.3.3 2.7.1.39]	46	49	51	54	42	60
Cellular Processes	Cell growth and death	04113 Meiosis - yeast [PATH:ko04113]	E4.6.1.1 adenylate cyclase [EC:4.6.1.1]	62	38	47	56	53	37
Metabolism	Carbohydrate metabolism	00650 Butanoate metabolism [PATH:ko00650]	E4.1.1.70 glutaconyl-CoA decarboxylase [EC:4.1.1.70]	42	47	47	91	32	55
Metabolism	Metabolism of cofactors and vitamins	00785 Lipoic acid metabolism [PATH:ko00785]	lplA lipoate-protein ligase A [EC:2.7.7.63]	30	51	51	33	43	85
Environmental Information Processing	Signal transduction	02020 Two-component system [PATH:ko02020]	citE citrate lyase subunit beta / citryl-CoA lyase [EC:4.1.3.6 4.1.3.34]	39	59	39	65	56	39
Cellular Processes	Cell motility	02040 Flagellar assembly [PATH:ko02040]	fliF flagellar M-ring protein FliF	40	36	78	79	47	48
Metabolism	Glycan biosynthesis and metabolism	00540 Lipopolysaccharide biosynthesis [PATH:ko00540]	kdtA waaA 3-deoxy-D-manno-octulosonic-acid transferase [EC:2.4.99.12 2.4.99.13 2.4.99.14 2.4.99.15]	56	38	16	48	58	46
Metabolism	Carbohydrate metabolism	00010 Glycolysis / Gluconeogenesis [PATH:ko00010]	fbp3 fructose-16-bisphosphatase III [EC:3.1.3.11]	28	46	43	55	49	70
Metabolism	Metabolism of cofactors and vitamins	00760 Nicotinate and nicotinamide metabolism [PATH:ko00760]	surE 5'-nucleotidase [EC:3.1.3.5]	43	60	51	50	44	51
Environmental Information Processing	Membrane transport	02010 ABC transporters [PATH:ko02010]	ABC.GLN1.S putative glutamine transport system substrate-binding protein	46	55	35	57	41	52
Metabolism	Metabolism of other amino acids	00440 Phosphonate and phosphinate metabolism [PATH:ko00440]	phnW 2-aminoethylphosphonate-pyruvate transaminase [EC:2.6.1.37]	44	84	12	20	46	55
Metabolism	Carbohydrate metabolism	00500 Starch and sucrose metabolism [PATH:ko00500]	E2.4.1.20 cellobiose phosphorylase [EC:2.4.1.20]	36	34	43	8	75	67
Metabolism	Amino acid metabolism	00400 Phenylalanine tyrosine and tryptophan biosynthesis [PATH:ko00400]	aroE shikimate dehydrogenase [EC:1.1.1.25]	38	32	55	44	71	48

Metabolism	Metabolism of cofactors and vitamins	00760 Nicotinate and nicotinamide metabolism [PATH:ko00760]	nadC QPRT nicotinate-nucleotide pyrophosphorylase (carboxylating) [EC:2.4.2.19]	22	34	31	55	58	74
Metabolism	Amino acid metabolism	00260 Glycine serine and threonine metabolism [PATH:ko00260]	E4.3.1.17 sdaA L-serine dehydratase [EC:4.3.1.17]	33	35	62	42	42	80
Metabolism	Metabolism of cofactors and vitamins	00130 Ubiquinone and other terpenoid-quinone biosynthesis [PATH:ko00130]	ubiD 3-octaprenyl-4-hydroxybenzoate carboxy-lyase UbiD [EC:4.1.1.-]	44	51	74	43	59	39
Environmental Information Processing	Membrane transport	02010 ABC transporters [PATH:ko02010]	ABC.LPT.P lolC lolE lipoprotein-releasing system permease protein	38	25	20	41	76	53
Genetic Information Processing	Folding sorting and degradation	03018 RNA degradation [PATH:ko03018]	pcnB poly(A) polymerase [EC:2.7.7.19]	52	38	62	53	50	43
Metabolism	Carbohydrate metabolism	00040 Pentose and glucuronate interconversions [PATH:ko00040]	uxaA altronate hydrolase [EC:4.2.1.7]	51	35	31	80	29	58
Metabolism	Amino acid metabolism	00330 Arginine and proline metabolism [PATH:ko00330]	eda 2-dehydro-3-deoxyphosphogluconate aldolase / 4-hydroxy-2-oxoglutarate aldolase [EC:4.1.2.14 4.1.3.16]	44	55	51	61	33	55
Environmental Information Processing	Membrane transport	02060 Phosphotransferase system (PTS) [PATH:ko02060]	PTS-Gat-EIIC gatC PTS system galactitol-specific IIC component	49	59	23	42	42	52
Metabolism	Metabolism of cofactors and vitamins	00780 Biotin metabolism [PATH:ko00780]	bioF 8-amino-7-oxononanoate synthase [EC:2.3.1.47]	39	27	16	66	56	56
Environmental Information Processing	Membrane Transport	02010 ABC transporters [PATH:ko02010]	ABC.SN.P ssuC tauC sulfonate/nitrate/taurine transport system permease protein	42	60	62	37	47	50
Metabolism	Nucleotide metabolism	00240 Pyrimidine metabolism [PATH:ko00240]	E3.5.4.5 cdd cytidine deaminase [EC:3.5.4.5]	32	65	20	59	47	51
Environmental Information Processing	Membrane transport	02010 ABC transporters [PATH:ko02010]	ABC-2.AB.P antibiotic transport system permease protein	27	31	27	37	90	44
Metabolism	Amino acid metabolism	00330 Arginine and proline metabolism [PATH:ko00330]	E1.5.1.2 proC pyrroline-5-carboxylate reductase [EC:1.5.1.2]	57	53	51	53	32	46
Metabolism	Amino acid metabolism	00250 Alanine aspartate and glutamate metabolism [PATH:ko00250]	alaA alanine-synthesizing transaminase [EC:2.6.1.66 2.6.1.2]	72	41	70	16	54	32

Metabolism	Carbohydrate metabolism	00562 Inositol phosphate metabolism [PATH:ko00562]	iolD 3D-(35/4)-trihydroxycyclohexane-12-dione hydrolase [EC:3.7.1.-]	56	61	74	47	35	36
Cellular Processes	Cell motility	02040 Flagellar assembly [PATH:ko02040]	fliI flagellum-specific ATP synthase [EC:3.6.3.14]	60	42	109	63	33	30
Metabolism	Metabolism of cofactors and vitamins	00760 Nicotinate and nicotinamide metabolism [PATH:ko00760]	nadD nicotinate-nucleotide adenylyltransferase [EC:2.7.7.18]	30	36	51	50	59	57
Genetic Information Processing	Folding sorting and degradation	04122 Sulfur relay system [PATH:ko04122]	thiI thiamine biosynthesis protein ThiI	59	54	51	44	34	42
Genetic Information Processing	Translation	03013 RNA transport [PATH:ko03013]	cca tRNA nucleotidyltransferase (CCA-adding enzyme) [EC:2.7.7.72 3.1.3.- 3.1.4.-]	49	46	39	27	50	51
Metabolism	Carbohydrate metabolism	00052 Galactose metabolism [PATH:ko00052]	E3.2.1.20 malZ alpha-glucosidase [EC:3.2.1.20]	35	43	23	34	39	78
Environmental Information Processing	Membrane transport	02010 ABC transporters [PATH:ko02010]	mglC methyl-galactoside transport system permease protein	45	56	47	70	27	47
Metabolism	Carbohydrate metabolism	00040 Pentose and glucuronate interconversions [PATH:ko00040]	araB L-ribulokinase [EC:2.7.1.16]	21	113	39	38	38	41
Metabolism	Carbohydrate metabolism	00620 Pyruvate metabolism [PATH:ko00620]	fucO lactaldehyde reductase [EC:1.1.1.77]	40	34	59	53	39	59
Cellular Processes	Cell motility	02040 Flagellar assembly [PATH:ko02040]	flgL flagellar hook-associated protein 3 FlgL	43	43	98	66	34	40
Metabolism	Xenobiotics biodegradation and metabolism	00983 Drug metabolism - other enzymes [PATH:ko00983]	CES2 carboxylesterase 2 [EC:3.1.1.1 3.1.1.84 3.1.1.56]	58	50	62	47	24	46
Metabolism	Carbohydrate metabolism	00040 Pentose and glucuronate interconversions [PATH:ko00040]	uxuA mannonate dehydratase [EC:4.2.1.8]	36	41	35	46	42	61
Metabolism	Carbohydrate metabolism	00520 Amino sugar and nucleotide sugar metabolism [PATH:ko00520]	wecC UDP-N-acetyl-D-mannosaminuronic acid dehydrogenase [EC:1.1.1.336]	120	16	66	11	38	6
Environmental Information Processing	Membrane transport	02010 ABC transporters [PATH:ko02010]	ABC-2.LPSE.P lipopolysaccharide transport system permease protein	56	56	43	34	41	35
Metabolism	Glycan biosynthesis and metabolism	00540 Lipopolysaccharide biosynthesis [PATH:ko00540]	lpxB lipid-A-disaccharide synthase [EC:2.4.1.182]	34	27	12	45	65	50

Environmental Information Processing	Membrane transport	02010 ABC transporters [PATH:ko02010]	ABC.FEV.A iron complex transport system ATP-binding protein [EC:3.6.3.34]	44	53	47	33	45	46
Metabolism	Amino acid metabolism	00340 Histidine metabolism [PATH:ko00340]	FTCD glutamate formiminotransferase / formiminotetrahydrofolate cyclodeaminase [EC:2.1.2.5 4.3.1.4]	20	20	16	23	35	114
Environmental Information Processing	Signal transduction	02020 Two-component system [PATH:ko02020]	cydB cytochrome d ubiquinol oxidase subunit II [EC:1.10.3.-]	46	32	20	59	42	48
Metabolism	Energy metabolism	00680 Methane metabolism [PATH:ko00680]	E1.5.1.20 metF methylenetetrahydrofolate reductase (NADPH) [EC:1.5.1.20]	39	41	70	29	54	47
Genetic Information Processing	Replication and repair	03030 DNA replication [PATH:ko03030]	DPO3D2 holB DNA polymerase III subunit delta' [EC:2.7.7.7]	30	42	20	40	57	53
Metabolism	Carbohydrate metabolism	00051 Fructose and mannose metabolism [PATH:ko00051]	E2.4.1.- [EC:2.4.1.-]	41	37	31	29	51	55
Metabolism	Carbohydrate metabolism	00010 Glycolysis / Gluconeogenesis [PATH:ko00010]	aceE pyruvate dehydrogenase E1 component [EC:1.2.4.1]	56	50	62	20	65	16
Metabolism	Amino acid metabolism	00280 Valine leucine and isoleucine degradation [PATH:ko00280]	E1.2.1.3 aldehyde dehydrogenase (NAD+) [EC:1.2.1.3]	53	67	27	31	32	41
Metabolism	Carbohydrate metabolism	00010 Glycolysis / Gluconeogenesis [PATH:ko00010]	porA pyruvate ferredoxin oxidoreductase alpha subunit [EC:1.2.7.1]	29	48	23	74	31	55
Metabolism	Glycan biosynthesis and metabolism	00550 Peptidoglycan biosynthesis [PATH:ko00550]	dacC dacA dacD D-alanyl-D-alanine carboxypeptidase (penicillin-binding protein 5/6) [EC:3.4.16.4]	43	43	31	33	44	51
Metabolism	Carbohydrate metabolism	00620 Pyruvate metabolism [PATH:ko00620]	accB bccP acetyl-CoA carboxylase biotin carboxyl carrier protein	40	32	62	34	60	38
Environmental Information Processing	Signal transduction	04070 Phosphatidylinositol signaling system [PATH:ko04070]	E3.1.3.25 IMPA suhB myo-inositol-1(or 4)-monophosphatase [EC:3.1.3.25]	28	48	47	22	78	29
Metabolism	Metabolism of other amino acids	00480 Glutathione metabolism [PATH:ko00480]	E6.3.2.3 gshB glutathione synthase [EC:6.3.2.3]	57	27	59	12	76	18

Metabolism	Carbohydrate metabolism	00500 Starch and sucrose metabolism [PATH:ko00500]	E3.2.1.91 cellulose 14-beta-cellobiosidase [EC:3.2.1.91]	34	56	66	4	84	17
Metabolism	Metabolism of cofactors and vitamins	00730 Thiamine metabolism [PATH:ko00730]	thiG thiamine biosynthesis ThiG	44	42	98	47	42	29
Metabolism	Amino acid metabolism	00280 Valine leucine and isoleucine degradation [PATH:ko00280]	E1.1.1.31 mmsB 3-hydroxyisobutyrate dehydrogenase [EC:1.1.1.31]	51	89	27	18	48	14
Genetic Information Processing	Replication and repair	03430 Mismatch repair [PATH:ko03430]	dam DNA adenine methylase [EC:2.1.1.72]	42	37	31	29	60	37
Metabolism	Amino acid metabolism	00270 Cysteine and methionine metabolism [PATH:ko00270]	mtnA methylthioribose-1-phosphate isomerase [EC:5.3.1.23]	47	38	23	32	43	48
Metabolism	Amino acid metabolism	00400 Phenylalanine tyrosine and tryptophan biosynthesis [PATH:ko00400]	trpD anthranilate phosphoribosyltransferase [EC:2.4.2.18]	40	32	66	27	69	27
Environmental Information Processing	Membrane transport	02010 ABC transporters [PATH:ko02010]	lacE araN lactose/L-arabinose transport system substrate-binding protein	41	50	43	49	35	41
Environmental Information Processing	Signal transduction	02020 Two-component system [PATH:ko02020]	vicR two-component system OmpR family response regulator VicR	46	60	31	33	31	45
Metabolism	Glycan biosynthesis and metabolism	00540 Lipopolysaccharide biosynthesis [PATH:ko00540]	lpxK tetraacyldisaccharide 4'-kinase [EC:2.7.1.130]	22	23	47	37	60	58
Metabolism	Carbohydrate metabolism	00520 Amino sugar and nucleotide sugar metabolism [PATH:ko00520]	murQ N-acetylmuramic acid 6-phosphate etherase [EC:4.2.1.126]	44	78	16	36	21	46
Genetic Information Processing	Replication and repair	03440 Homologous recombination [PATH:ko03440]	ruvC crossover junction endodeoxyribonuclease RuvC [EC:3.1.22.4]	27	44	23	47	49	48
Metabolism	Carbohydrate metabolism	00040 Pentose and glucuronate interconversions [PATH:ko00040]	araD L-ribulose-5-phosphate 4-epimerase [EC:5.1.3.4]	43	43	31	43	44	38
Genetic Information Processing	Replication and repair	03410 Base excision repair [PATH:ko03410]	nfo deoxyribonuclease IV [EC:3.1.21.2]	43	45	31	44	47	32
Metabolism	Amino acid metabolism	00400 Phenylalanine tyrosine and tryptophan biosynthesis [PATH:ko00400]	trpC indole-3-glycerol phosphate synthase [EC:4.1.1.48]	48	31	35	24	62	30

Environmental Information Processing	Signal transduction	02020 Two-component system [PATH:ko02020]	chpA chemosensory pili system protein ChpA (sensor histidine kinase/response regulator)	78	41	23	23	45	12
Metabolism	Carbohydrate metabolism	00051 Fructose and mannose metabolism [PATH:ko00051]	fucI L-fucose isomerase [EC:5.3.1.25]	46	31	23	55	28	52
Environmental Information Processing	Membrane transport	03070 Bacterial secretion system [PATH:ko03070]	tatC sec-independent protein translocase protein TatC	36	47	66	34	51	31
Metabolism	Metabolism of cofactors and vitamins	00770 Pantothenate and CoA biosynthesis [PATH:ko00770]	coaX type III pantothenate kinase [EC:2.7.1.33]	42	43	31	55	40	31
Metabolism	sulfur metabolism	00920 Sulfur metabolism [PATH:ko00920]	dsrA sulfite reductase dissimilatory-type alpha subunit [EC 1.8.99.5]	40	51	8	53	32	40
Metabolism	Biosynthesis of other secondary metabolites	00521 Streptomycin biosynthesis [PATH:ko00521]	iolG myo-inositol 2-dehydrogenase [EC:1.1.1.18]	46	65	27	48	17	41
Environmental Information Processing	Membrane transport	02010 ABC transporters [PATH:ko02010]	ABC.FEV.S iron complex transport system substrate-binding protein	28	50	129	40	48	23
Genetic Information Processing	Folding sorting and degradation	04122 Sulfur relay system [PATH:ko04122]	MOCS1 moaA molybdenum cofactor biosynthesis protein	44	38	35	46	36	36
Metabolism	Energy metabolism	00190 Oxidative phosphorylation [PATH:ko00190]	nuoI NADH-quinone oxidoreductase subunit I [EC:1.6.5.3]	44	48	55	33	44	25
Metabolism	Lipid metabolism	00564 Glycerophospholipid metabolism [PATH:ko00564]	tagD glycerol-3-phosphate cytidylyltransferase [EC:2.7.7.39]	44	42	59	43	41	26
Metabolism	Metabolism of cofactors and vitamins	00860 Porphyrin and chlorophyll metabolism [PATH:ko00860]	hemH FECH ferrochelatase [EC:4.99.1.1]	43	43	51	25	57	20
Environmental Information Processing	Membrane transport	03070 Bacterial secretion system [PATH:ko03070]	secB preprotein translocase subunit SecB	53	21	59	21	58	23
Environmental Information Processing	nitrogen metabolism	00910 Nitrogen metabolism [PATH:ko00910]	narG nitrate reductase 1 alpha subunit [EC:1.7.99.4]	69	58	55	24	35	6
Environmental Information Processing	Membrane transport	02010 ABC transporters [PATH:ko02010]	cbiM cobalt/nickel transport system permease protein	40	73	31	16	44	25
Metabolism	Glycan biosynthesis and metabolism	00540 Lipopolysaccharide biosynthesis [PATH:ko00540]	htrB lipid A biosynthesis lauroyl acyltransferase [EC:2.3.1.-]	31	26	62	29	56	39

Metabolism	Amino acid metabolism	00260 Glycine serine and threonine metabolism [PATH:ko00260]	hprA glycerate dehydrogenase [EC:1.1.1.29]	33	36	43	42	28	55
Environmental Information Processing	Membrane transport	02010 ABC transporters [PATH:ko02010]	phnD phosphonate transport system substrate-binding protein	37	48	51	44	21	46
Metabolism	Metabolism of terpenoids and polyketides	00900 Terpenoid backbone biosynthesis [PATH:ko00900]	ispDF 2-C-methyl-D-erythritol 4-phosphate cytidylyltransferase / 2-C-methyl-D-erythritol 24-cyclodiphosphate synthase [EC:2.7.7.60 4.6.1.12]	47	38	47	69	18	30
Metabolism	Energy metabolism	00680 Methane metabolism [PATH:ko00680]	hdrB heterodisulfide reductase subunit B [EC:1.8.98.1]	44	53	12	18	39	37
Metabolism	Carbohydrate metabolism	00630 Glyoxylate and dicarboxylate metabolism [PATH:ko00630]	E1.1.1.60 garR 2-hydroxy-3-oxopropionate reductase [EC:1.1.1.60]	33	57	12	41	32	39
Metabolism	Carbohydrate metabolism	00500 Starch and sucrose metabolism [PATH:ko00500]	E3.2.1.1A alpha-amylase [EC:3.2.1.1]	28	21	8	53	25	68
Genetic Information Processing	Transcription	03020 RNA polymerase [PATH:ko03020]	SIG3.3.2 sigB RNA polymerase sigma-B factor	23	28	55	53	31	55
Metabolism	Energy metabolism	00910 Nitrogen metabolism [PATH:ko00910]	napB cytochrome c-type protein NapB	30	53	20	84	17	32
Metabolism	Biosynthesis of other secondary metabolites	00521 Streptomycin biosynthesis [PATH:ko00521]	E5.5.1.4 INO1 myo-inositol-1-phosphate synthase [EC:5.5.1.4]	20	28	16	32	43	62
Metabolism	Lipid metabolism	00564 Glycerophospholipid metabolism [PATH:ko00564]	E3.1.4.46 glpQ ugpQ glycerophosphoryl diester phosphodiesterase [EC:3.1.4.46]	49	49	31	36	30	25
Metabolism	Amino acid metabolism	00280 Valine leucine and isoleucine degradation [PATH:ko00280]	ACADM acd acyl-CoA dehydrogenase [EC:1.3.8.7]	42	33	31	18	36	46
Metabolism	Lipid metabolism	00590 Arachidonic acid metabolism [PATH:ko00590]	ggt gamma-glutamyltranspeptidase [EC:2.3.2.2]	35	43	27	46	26	42
Metabolism	Amino acid metabolism	00300 Lysine biosynthesis [PATH:ko00300]	dapD 2345-tetrahydropyridine-2-carboxylate N-succinyltransferase [EC:2.3.1.117]	11	12	12	23	56	70
Metabolism	Glycan biosynthesis and metabolism	00550 Peptidoglycan biosynthesis [PATH:ko00550]	vanY D-alanyl-D-alanine carboxypeptidase [EC:3.4.16.4]	35	44	12	30	28	50

Genetic Information Processing	Folding sorting and degradation	04122 Sulfur relay system [PATH:ko04122]	moaC molybdenum cofactor biosynthesis protein C	40	38	109	45	27	23
Metabolism	Amino acid metabolism	00270 Cysteine and methionine metabolism [PATH:ko00270]	metC cystathionine beta-lyase [EC:4.4.1.8]	37	52	31	52	29	25
Metabolism	Amino acid metabolism	00260 Glycine serine and threonine metabolism [PATH:ko00260]	thrB1 homoserine kinase [EC:2.7.1.39]	40	41	62	34	34	28
Metabolism	Amino acid metabolism	00350 Tyrosine metabolism [PATH:ko00350]	adhP alcohol dehydrogenase propanol-preferring [EC:1.1.1.1]	25	50	4	21	19	70
Metabolism	Amino acid metabolism	00300 Lysine biosynthesis [PATH:ko00300]	dapE succinyl-diaminopimelate desuccinylase [EC:3.5.1.18]	32	45	27	49	30	34
Genetic Information Processing	Folding sorting and degradation	03060 Protein export [PATH:ko03060]	lspA signal peptidase II [EC:3.4.23.36]	20	33	20	41	48	42
Environmental Information Processing	Membrane transport	02010 ABC transporters [PATH:ko02010]	malF maltose/maltodextrin transport system permease protein	29	20	8	49	34	51
Metabolism	Energy metabolism	00910 Nitrogen metabolism [PATH:ko00910]	napH ferredoxin-type protein NapH	37	32	47	56	14	46
Metabolism	Metabolism of cofactors and vitamins	00860 Porphyrin and chlorophyll metabolism [PATH:ko00860]	cbiK sirohydrochlorin cobaltochelatase [EC:4.99.1.3]	32	37	59	30	33	41
Cellular Processes	Cell motility	02040 Flagellar assembly [PATH:ko02040]	fliE flagellar hook-basal body complex protein FliE	29	21	74	25	43	44
Metabolism	Metabolism of cofactors and vitamins	00730 Thiamine metabolism [PATH:ko00730]	thiD hydroxymethylpyrimidine/phosphomethylpyrimidine kinase [EC:2.7.1.49 2.7.4.7]	36	48	62	23	32	34
Metabolism	Lipid metabolism	00561 Glycerolipid metabolism [PATH:ko00561]	plsC 1-acyl-sn-glycerol-3-phosphate acyltransferase [EC:2.3.1.51]	36	37	16	41	38	31
Environmental Information Processing	Membrane transport	02010 ABC transporters [PATH:ko02010]	znuC zinc transport system ATP-binding protein [EC:3.6.3.-]	37	36	35	40	36	29
Human Diseases	oxidative stress response	00480 Glutathione metabolism [PATH:ko00480]	GST gst glutathione S-transferase [EC:2.5.1.18]	37	44	70	13	56	11
Metabolism	Amino acid metabolism	00310 Lysine degradation [PATH:ko00310]	dat D-alanine transaminase [EC:2.6.1.21]	45	42	70	32	21	30

Human Diseases	Amino acid metabolism	00220 Arginine biosynthesis [PATH:ko00220]	E3.5.3.1 rocF arg arginase [EC:3.5.3.1]	33	50	12	32	33	34
Metabolism	Carbohydrate metabolism	00620 Pyruvate metabolism [PATH:ko00620]	E3.1.2.6 gloB hydroxyacylglutathione hydrolase [EC:3.1.2.6]	12	142	12	12	26	15
Environmental Information Processing	Membrane transport	02060 Phosphotransferase system (PTS) [PATH:ko02060]	PTS-Fru-EIIA fruB PTS system fructose-specific IIA component [EC:2.7.1.69]	21	42	8	36	43	39
Metabolism	Nucleotide metabolism	00240 Pyrimidine metabolism [PATH:ko00240]	pyrR pyrimidine operon attenuation protein / uracil phosphoribosyltransferase [EC:2.4.2.9]	36	36	16	37	30	39
Metabolism	Carbohydrate metabolism	00640 Propanoate metabolism [PATH:ko00640]	E4.1.3.30 prpB methylisocitrate lyase [EC:4.1.3.30]	34	18	43	8	70	22
Metabolism	Metabolism of cofactors and vitamins	00740 Riboflavin metabolism [PATH:ko00740]	ribF riboflavin kinase / FMN adenylyltransferase [EC:2.7.1.26 2.7.7.2]	24	37	23	46	36	37
Metabolism	Carbohydrate metabolism	00620 Pyruvate metabolism [PATH:ko00620]	mhpF acetaldehyde dehydrogenase [EC:1.2.1.10]	69	48	82	20	7	17
Metabolism	Carbohydrate metabolism	00020 Citrate cycle (TCA cycle) [PATH:ko00020]	sdhD succinate dehydrogenase membrane anchor subunit	52	26	62	3	51	13
Metabolism	Amino acid metabolism	00340 Histidine metabolism [PATH:ko00340]	E2.1.2.5 glutamate formiminotransferase [EC:2.1.2.5]	21	33	31	38	35	43
Metabolism	Xenobiotics biodegradation and metabolism	00362 Benzoate degradation [PATH:ko00362]	badH 2-hydroxycyclohexanecarboxyl-CoA dehydrogenase [EC:1.1.1.-]	42	26	31	11	55	18
Metabolism	Amino acid metabolism	00340 Histidine metabolism [PATH:ko00340]	hisZ ATP phosphoribosyltransferase regulatory subunit	37	35	35	12	54	18
Metabolism	Metabolism of cofactors and vitamins	00130 Ubiquinone and other terpenoid-quinone biosynthesis [PATH:ko00130]	ubiB aarF ubiquinone biosynthesis protein	40	24	12	16	53	24
Metabolism	Carbohydrate metabolism	00040 Pentose and glucuronate interconversions [PATH:ko00040]	araA L-arabinose isomerase [EC:5.3.1.4]	28	22	39	34	26	50
Metabolism	Amino acid metabolism	00330 Arginine and proline metabolism [PATH:ko00330]	nspC carboxynorspermidine decarboxylase [EC:4.1.1.-]	28	23	43	41	39	31

Metabolism	Nucleotide metabolism	00230 Purine metabolism [PATH:ko00230]	cpdB 2'3'-cyclic-nucleotide 2'-phosphodiesterase [EC:3.1.4.16]	41	28	23	42	29	27
Environmental Information Processing	Energy metabolism	00190 Oxidative phosphorylation [PATH:ko00190]	frdC fumarate reductase subunit C	39	40	74	37	23	22
Metabolism	Carbohydrate metabolism	00630 Glyoxylate and dicarboxylate metabolism [PATH:ko00630]	E1.2.1.2B1 formate dehydrogenase beta subunit	33	41	98	45	17	25
Metabolism	Amino acid metabolism	00340 Histidine metabolism [PATH:ko00340]	hisI phosphoribosyl-AMP cyclohydrolase [EC:3.5.4.19]	39	22	35	38	41	19
Environmental Information Processing	Signal transduction	02020 Two-component system [PATH:ko02020]	pilJ twitching motility protein PilJ	49	27	47	20	35	20
Metabolism	Amino acid metabolism	00250 Alanine aspartate and glutamate metabolism [PATH:ko00250]	pyrBI aspartate carbamoyltransferase [EC:2.1.3.2]	20	5	47	72	47	22
Genetic Information Processing	Translation	03013 RNA transport [PATH:ko03013]	rnz ribonuclease Z [EC:3.1.26.11]	31	16	55	43	34	34
Metabolism	Amino acid metabolism	00400 Phenylalanine tyrosine and tryptophan biosynthesis [PATH:ko00400]	AROA2 aroA 3-deoxy-7-phosphoheptulonate synthase [EC:2.5.1.54]	42	28	43	44	16	33
Metabolism	Carbohydrate metabolism	00500 Starch and sucrose metabolism [PATH:ko00500]	E3.2.1.1 amyA malS alpha-amylase [EC:3.2.1.1]	35	28	16	22	46	25
Metabolism	Glycan biosynthesis and metabolism	00550 Peptidoglycan biosynthesis [PATH:ko00550]	mviN virulence factor	36	29	35	29	39	24
Metabolism	Carbohydrate metabolism	00030 Pentose phosphate pathway [PATH:ko00030]	rpiA ribose 5-phosphate isomerase A [EC:5.3.1.6]	28	31	70	20	30	39
Metabolism	Amino acid metabolism	00260 Glycine serine and threonine metabolism [PATH:ko00260]	serB PSPH phosphoserine phosphatase [EC:3.1.3.3]	29	36	51	19	41	28
Environmental Information Processing	Membrane transport	02010 ABC transporters [PATH:ko02010]	xylG D-xylose transport system ATP-binding protein [EC:3.6.3.17]	37	40	12	33	35	20
Metabolism	Energy metabolism	00190 Oxidative phosphorylation [PATH:ko00190]	nuoJ NADH-quinone oxidoreductase subunit J [EC:1.6.5.3]	35	23	74	18	48	17

Metabolism	Energy metabolism	00190 Oxidative phosphorylation [PATH:ko00190]	E1.6.99.3 NADH dehydrogenase [EC:1.6.99.3]	27	86	4	33	15	22
Metabolism	Lipid metabolism	00561 Glycerolipid metabolism [PATH:ko00561]	pduC propanediol dehydratase large subunit [EC:4.2.1.28]	23	47	4	57	18	35
Environmental Information Processing	Membrane transport	02010 ABC transporters [PATH:ko02010]	potC spermidine/putrescine transport system permease protein	30	29	20	57	26	27
Environmental Information Processing	Membrane transport	02010 ABC transporters [PATH:ko02010]	ABC-2.CPSE.P1 capsular polysaccharide transport system permease protein	38	18	27	7	65	12
Metabolism	Carbohydrate metabolism	00640 Propanoate metabolism [PATH:ko00640]	E2.3.3.5 prpC 2-methylcitrate synthase [EC:2.3.3.5]	28	32	47	13	42	30
Metabolism	Amino acid metabolism	00400 Phenylalanine tyrosine and tryptophan biosynthesis [PATH:ko00400]	E2.7.1.71 aroK aroL shikimate kinase [EC:2.7.1.71]	31	24	47	31	34	30
Metabolism	Metabolism of cofactors and vitamins	00860 Porphyrin and chlorophyll metabolism [PATH:ko00860]	E6.3.5.10 cobQ cbiP adenosylcobyric acid synthase [EC:6.3.5.10]	33	30	20	33	33	29
Metabolism	sulfur metabolism	00920 Sulfur metabolism [PATH:ko00920]	dsrB sulfite reductase dissimilatory-type beta subunit [EC:1.8.99.3]	29	32	20	30	25	42
Metabolism	Glycan biosynthesis and metabolism	00540 Lipopolysaccharide biosynthesis [PATH:ko00540]	lpxH UDP-23-diacylglucosamine hydrolase [EC:3.6.1.54]	30	19	16	21	48	32
Environmental Information Processing	Membrane transport	02060 Phosphotransferase system (PTS) [PATH:ko02060]	PTS-Man-EIID manZ PTS system mannose-specific IID component	15	17	27	14	46	51
Cellular Processes	Cell motility	02040 Flagellar assembly [PATH:ko02040]	flhC flagellar transcriptional activator FlhC	35	47	94	29	9	30
Metabolism	Energy metabolism	00190 Oxidative phosphorylation [PATH:ko00190]	ATPVE ntpE V-type H+-transporting ATPase subunit E [EC:3.6.3.14]	33	19	23	55	25	29
Metabolism	Carbohydrate metabolism	00051 Fructose and mannose metabolism [PATH:ko00051]	rhaA L-rhamnose isomerase [EC:5.3.1.14]	32	35	16	23	20	44
Metabolism	Nucleotide metabolism	00230 Purine metabolism [PATH:ko00230]	xpt xanthine phosphoribosyltransferase [EC:2.4.2.22]	37	52	16	22	21	27

Environmental Information Processing	Membrane transport	02010 ABC transporters [PATH:ko02010]	potB spermidine/putrescine transport system permease protein	28	47	35	34	18	32
Metabolism	Carbohydrate metabolism	00040 Pentose and glucuronate interconversions [PATH:ko00040]	E5.3.1.17 kduI 4-deoxy-L-threo-5-hexosulose-uronate ketol-isomerase [EC:5.3.1.17]	22	40	12	37	27	34
Metabolism	Energy metabolism	00680 Methane metabolism [PATH:ko00680]	E1.12.7.2L ferredoxin hydrogenase large subunit [EC:1.12.7.2]	36	28	4	27	21	38
Metabolism	Carbohydrate metabolism	00010 Glycolysis / Gluconeogenesis [PATH:ko00010]	porG pyruvate ferredoxin oxidoreductase gamma subunit [EC:1.2.7.1]	13	21	51	37	48	25
Cellular Processes	Cell motility	02040 Flagellar assembly [PATH:ko02040]	flgI flagellar P-ring protein precursor FlgI	27	15	27	32	35	34
Metabolism	Amino acid metabolism	00250 Alanine aspartate and glutamate metabolism [PATH:ko00250]	gltB glutamate synthase (NADPH/NADH) large chain [EC:1.4.1.13 1.4.1.14]	21	15	27	32	20	57
Environmental Information Processing	Membrane transport	02060 Phosphotransferase system (PTS) [PATH:ko02060]	PTS-Fru-EIIC fruA PTS system fructose-specific IIC component	18	32	12	24	24	51
Genetic Information Processing	Replication and repair	03030 DNA replication [PATH:ko03030]	DPO3D1 holA DNA polymerase III subunit delta [EC:2.7.7.7]	16	16	16	32	33	49
Metabolism	Amino acid metabolism	00260 Glycine serine and threonine metabolism [PATH:ko00260]	E2.3.1.37 ALAS 5-aminolevulinate synthase [EC:2.3.1.37]	42	78	4	13	7	21
Metabolism	Carbohydrate metabolism	00660 C5-Branched dibasic acid metabolism [PATH:ko00660]	E4.3.1.2 methylaspartate ammonia-lyase [EC:4.3.1.2]	10	22	70	49	31	34
Metabolism	Carbohydrate metabolism	00010 Glycolysis / Gluconeogenesis [PATH:ko00010]	glpX fructose-16-bisphosphatase II [EC:3.1.3.11]	33	37	4	37	17	32
Metabolism	Carbohydrate metabolism	00030 Pentose phosphate pathway [PATH:ko00030]	E1.1.1.44 PGD gnd 6-phosphogluconate dehydrogenase [EC:1.1.1.44]	30	43	43	31	26	18
Metabolism	Xenobiotics biodegradation and metabolism	00361 Chlorocyclohexane and chlorobenzene degradation [PATH:ko00361]	dmpB catechol 23-dioxygenase [EC:1.13.11.2]	59	44	59	10	5	18
Metabolism	Amino acid metabolism	00330 Arginine and proline metabolism [PATH:ko00330]	E3.5.3.11 speB agmatinase [EC:3.5.3.11]	19	19	16	14	36	48

Environmental Information Processing	Membrane transport	02060 Phosphotransferase system (PTS) [PATH:ko02060]	PTS-Man-EIIB manX PTS system mannose-specific IIB component [EC:2.7.1.69]	28	23	4	16	39	32
Environmental Information Processing	Signal transduction	02020 Two-component system [PATH:ko02020]	pilH twitching motility two-component system response regulator PilH	28	57	35	32	26	10
Environmental Information Processing	Membrane transport	02010 ABC transporters [PATH:ko02010]	ABC.GLN1.A putative glutamine transport system ATP-binding protein [EC:3.6.3.-]	28	32	23	27	27	29
Metabolism	Metabolism of cofactors and vitamins	00790 Folate biosynthesis [PATH:ko00790]	E4.2.3.12 queD ptpS PTS 6-pyruvoyl tetrahydrobiopterin synthase [EC:4.2.3.12]	16	25	20	42	27	39
Metabolism	Carbohydrate metabolism	00030 Pentose phosphate pathway [PATH:ko00030]	gdh glucose 1-dehydrogenase [EC:1.1.1.47]	30	12	62	106	9	8
Environmental Information Processing	Signal transduction	02020 Two-component system [PATH:ko02020]	glnB nitrogen regulatory protein P-II 1	31	24	20	21	50	11
Metabolism	Amino acid metabolism	00250 Alanine aspartate and glutamate metabolism [PATH:ko00250]	pyrI aspartate carbamoyltransferase regulatory subunit	15	17	8	29	38	44
Metabolism	Nucleotide metabolism	00230 Purine metabolism [PATH:ko00230]	spoT HDDC3 guanosine-3'5'-bis(diphosphate) 3'-pyrophosphohydrolase [EC:3.1.7.2]	22	33	27	12	45	23
Metabolism	Energy metabolism	00910 Nitrogen metabolism [PATH:ko00910]	napC cytochrome c-type protein NapC	15	8	8	29	17	71
Metabolism	Carbohydrate metabolism	00051 Fructose and mannose metabolism [PATH:ko00051]	fruK 1-phosphofructokinase [EC:2.7.1.56]	13	14	31	11	50	39
Metabolism	Carbohydrate metabolism	00520 Amino sugar and nucleotide sugar metabolism [PATH:ko00520]	E2.7.7.43 neuA CMAS N-acylneuraminate cytidylyltransferase [EC:2.7.7.43]	24	43	8	37	27	20
Environmental Information Processing	Membrane transport	02010 ABC transporters [PATH:ko02010]	cbiQ cobalt/nickel transport system permease protein	31	35	39	26	24	23
Environmental Information Processing	Signal transduction	02020 Two-component system [PATH:ko02020]	E3.1.3.1 phoA phoB alkaline phosphatase [EC:3.1.3.1]	12	12	8	20	34	57

Environmental Information Processing	Membrane transport	02010 ABC transporters [PATH:ko02010]	pstS phosphate transport system substrate-binding protein	25	28	23	26	34	25
Metabolism	Carbohydrate metabolism	00500 Starch and sucrose metabolism [PATH:ko00500]	rfbF glucose-1-phosphate cytidylyltransferase [EC:2.7.7.33]	36	29	12	36	17	27
Metabolism	Metabolism of terpenoids and polyketides	00900 Terpenoid backbone biosynthesis [PATH:ko00900]	ispA farnesyl diphosphate synthase [EC:2.5.1.1 2.5.1.10]	30	29	47	30	26	21
Metabolism	Metabolism of cofactors and vitamins	00670 One carbon pool by folate [PATH:ko00670]	purT phosphoribosylglycinamide formyltransferase 2 [EC:2.1.2.2]	12	22	27	33	36	34
Metabolism	Carbohydrate metabolism	00010 Glycolysis / Gluconeogenesis [PATH:ko00010]	DLAT aceF pdhC pyruvate dehydrogenase E2 component (dihydrolipoamide acetyltransferase) [EC:2.3.1.12]	23	26	31	19	48	15
Metabolism	Amino acid metabolism	00280 Valine leucine and isoleucine degradation [PATH:ko00280]	PCCA pccA propionyl-CoA carboxylase alpha chain [EC:6.4.1.3]	44	48	74	16	11	11
Environmental Information Processing	Membrane transport	02060 Phosphotransferase system (PTS) [PATH:ko02060]	PTS-Man-EIIC manY PTS system mannose-specific IIC component	34	12	16	7	33	37
Metabolism	Amino acid metabolism	00360 Phenylalanine metabolism [PATH:ko00360]	enr 2-enoate reductase [EC:1.3.1.31]	29	17	20	32	15	43
Environmental Information Processing	Signal transduction	02020 Two-component system [PATH:ko02020]	cusR copR silR two-component system OmpR family copper resistance phosphate regulon response regulator CusR	33	38	78	21	21	15
Metabolism	Metabolism of cofactors and vitamins	00670 One carbon pool by folate [PATH:ko00670]	folA dihydrofolate reductase [EC:1.5.1.3]	9	21	20	14	36	49
Metabolism	Xenobiotics biodegradation and metabolism	00633 Nitrotoluene degradation [PATH:ko00633]	nemA N-ethylmaleimide reductase [EC:1.-.-.-]	34	42	12	14	26	20
Metabolism	Amino acid metabolism	00260 Glycine serine and threonine metabolism [PATH:ko00260]	tdh threonine 3-dehydrogenase [EC:1.1.1.103]	30	26	20	30	11	41
Metabolism	Metabolism of terpenoids and polyketides	00900 Terpenoid backbone biosynthesis [PATH:ko00900]	mvaA hydroxymethylglutaryl-CoA reductase [EC:1.1.1.88]	17	17	8	5	39	46
Metabolism	Amino acid metabolism	00400 Phenylalanine tyrosine and tryptophan biosynthesis	tyrA2 prephenate dehydrogenase [EC:1.3.1.12]	27	28	39	27	30	20

		[PATH:ko00400]							
Metabolism	Amino acid metabolism	00270 Cysteine and methionine metabolism [PATH:ko00270]	E2.5.1.48 metB cystathionine gamma-synthase [EC:2.5.1.48]	30	41	16	15	28	20
Metabolism	Carbohydrate metabolism	00051 Fructose and mannose metabolism [PATH:ko00051]	fucA L-fuculose-phosphate aldolase [EC:4.1.2.17]	25	39	27	37	24	15
Environmental Information Processing	Membrane transport	02010 ABC transporters [PATH:ko02010]	ugpB sn-glycerol 3-phosphate transport system substrate-binding protein	30	21	20	31	26	25
Metabolism	Metabolism of other amino acids	00450 Selenocompound metabolism [PATH:ko00450]	selD SEPHS selenide water dikinase [EC:2.7.9.3]	17	50	35	21	26	22
Cellular Processes	Cell motility	02030 Bacterial chemotaxis [PATH:ko02030]	cheD chemotaxis protein CheD [EC:3.5.1.44]	26	27	47	37	17	24
Metabolism	Metabolism of cofactors and vitamins	00130 Ubiquinone and other terpenoid-quinone biosynthesis [PATH:ko00130]	ubiA 4-hydroxybenzoate octaprenyltransferase [EC:2.5.1.-]	29	25	27	38	20	22
Metabolism	Xenobiotics biodegradation and metabolism	00633 Nitrotoluene degradation [PATH:ko00633]	E1.12.99.6L hydrogenase large subunit [EC:1.12.99.6]	24	38	16	45	13	23
Metabolism	Amino acid metabolism	00260 Glycine serine and threonine metabolism [PATH:ko00260]	E2.6.1.76 ectB diaminobutyrate-2-oxoglutarate transaminase [EC:2.6.1.76]	19	22	12	21	36	29
Metabolism	Lipid metabolism	00561 Glycerolipid metabolism [PATH:ko00561]	dhaK dihydroxyacetone kinase N-terminal domain [EC:2.7.1.-]	31	46	39	12	11	27
Metabolism	oxidative stress response	00360 Phenylalanine metabolism [PATH:ko00360]	katG catalase-peroxidase [EC:1.11.1.21]	59	34	8	15	11	8
Environmental Information Processing	Signal transduction	02020 Two-component system [PATH:ko02020]	pilG twitching motility two-component system response regulator PilG	38	38	16	9	32	8
Metabolism	Metabolism of cofactors and vitamins	00730 Thiamine metabolism [PATH:ko00730]	thiE thiamine-phosphate pyrophosphorylase [EC:2.5.1.3]	21	24	35	25	33	21
Metabolism	Amino acid metabolism	00330 Arginine and proline metabolism [PATH:ko00330]	argA amino-acid N-acetyltransferase [EC:2.3.1.1]	30	30	16	11	33	18
Metabolism	Carbohydrate metabolism	00562 Inositol phosphate metabolism [PATH:ko00562]	iolE inosose dehydratase [EC:4.2.1.44]	32	24	16	38	16	22

Environmental Information Processing	Signal transduction	02020 Two-component system [PATH:ko02020]	pilR two-component system NtrC family response regulator PilR	26	26	20	16	36	18
Metabolism	Carbohydrate metabolism	00010 Glycolysis / Gluconeogenesis [PATH:ko00010]	E1.2.7.5 aor aldehyde:ferredoxin oxidoreductase [EC:1.2.7.5]	29	40	12	33	13	20
Cellular Processes	Cell motility	02030 Bacterial chemotaxis [PATH:ko02030]	cheC chemotaxis protein CheC	20	42	27	44	14	18
Metabolism	Energy metabolism	00190 Oxidative phosphorylation [PATH:ko00190]	nuoK NADH-quinone oxidoreductase subunit K [EC:1.6.5.3]	25	22	31	15	39	15
Metabolism	Amino acid metabolism	00330 Arginine and proline metabolism [PATH:ko00330]	aguB N-carbamoylputrescine amidase [EC:3.5.1.53]	28	11	39	35	17	30
Metabolism	Nucleotide metabolism	00230 Purine metabolism [PATH:ko00230]	PPX1 exopolyphosphatase [EC:3.6.1.11]	25	23	27	23	30	18
Metabolism	oxidative stress response	00480 Glutathione metabolism [PATH:ko00480]	E1.8.1.7 GSR gor glutathione reductase (NADPH) [EC:1.8.1.7]	38	49	8	11	23	6
Metabolism	Carbohydrate metabolism	00650 Butanoate metabolism [PATH:ko00650]	gctA glutaconate CoA-transferase subunit A [EC:2.8.3.12]	33	28	20	27	13	24
Metabolism	Amino acid metabolism	00350 Tyrosine metabolism [PATH:ko00350]	hpaB 4-hydroxyphenylacetate 3-monooxygenase [EC:1.14.14.9]	24	31	23	25	15	30
Genetic Information Processing	Replication and repair	03410 Base excision repair [PATH:ko03410]	mutY A/G-specific adenine glycosylase [EC:3.2.2.-]	24	26	27	20	23	27
Metabolism	Energy metabolism	00680 Methane metabolism [PATH:ko00680]	E1.12.7.2 ferredoxin hydrogenase [EC:1.12.7.2]	24	32	4	36	23	18
Cellular Processes	Cell motility	02040 Flagellar assembly [PATH:ko02040]	fliP flagellar biosynthetic protein FliP	27	27	20	42	13	22
Environmental Information Processing	Membrane transport	02010 ABC transporters [PATH:ko02010]	ABC.MET.P metI D-methionine transport system permease protein	24	29	31	34	24	14
Cellular Processes	Cell motility	02040 Flagellar assembly [PATH:ko02040]	flgM negative regulator of flagellin synthesis FlgM	25	43	23	23	19	17
Metabolism	Energy metabolism	00190 Oxidative phosphorylation [PATH:ko00190]	ndh NADH dehydrogenase [EC:1.6.99.3]	33	7	55	34	19	19
Metabolism	Carbohydrate metabolism	00040 Pentose and glucuronate interconversions [PATH:ko00040]	rhaB rhamnulokinase [EC:2.7.1.5]	24	27	8	30	13	32

Metabolism	Carbohydrate metabolism	00520 Amino sugar and nucleotide sugar metabolism [PATH:ko00520]	rfbH CDP-6-deoxy-D-xylo-4-hexulose-3-dehydrase	27	28	23	33	15	21
Metabolism	Carbohydrate metabolism	00620 Pyruvate metabolism [PATH:ko00620]	pct propionate CoA-transferase [EC:2.8.3.1]	24	28	12	34	17	21
Metabolism	Metabolism of cofactors and vitamins	00130 Ubiquinone and other terpenoid-quinone biosynthesis [PATH:ko00130]	ubiG 2-polyprenyl-6-hydroxyphenyl methylase / 3-demethylubiquinone-9 3-methyltransferase [EC:2.1.1.222 2.1.1.64]	31	7	20	11	36	20
Metabolism	Metabolism of cofactors and vitamins	00860 Porphyrin and chlorophyll metabolism [PATH:ko00860]	E2.4.2.21 cobU cobT nicotinate-nucleotide--dimethylbenzimidazole phosphoribosyltransferase [EC:2.4.2.21]	25	24	12	27	19	24
Cellular Processes	Transport and catabolism	04142 Lysosome [PATH:ko04142]	E3.2.1.31 GUSB uidA beta-glucuronidase [EC:3.2.1.31]	24	17	4	9	44	16
Metabolism	Metabolism of cofactors and vitamins	00860 Porphyrin and chlorophyll metabolism [PATH:ko00860]	cobB-cbiA cobyrinic acid ac-diamide synthase [EC:6.3.5.9 6.3.5.11]	18	28	4	24	25	26
Cellular Processes	Cell motility	02040 Flagellar assembly [PATH:ko02040]	flhB flagellar biosynthetic protein FlhB	19	29	35	27	26	16
Metabolism	Nucleotide metabolism	00230 Purine metabolism [PATH:ko00230]	nudF ADP-ribose pyrophosphatase [EC:3.6.1.13]	25	33	27	18	17	23
Metabolism	Xenobiotics biodegradation and metabolism	00362 Benzoate degradation [PATH:ko00362]	pcaC 4-carboxymuconolactone decarboxylase [EC:4.1.1.44]	9	26	12	37	24	29
Genetic Information Processing	Folding sorting and degradation	03018 RNA degradation [PATH:ko03018]	rhlE ATP-dependent RNA helicase RhlE [EC:3.6.4.13]	20	52	12	14	14	24
Metabolism	Xenobiotics biodegradation and metabolism	00362 Benzoate degradation [PATH:ko00362]	E5.3.2.- 4-oxalocrotonate tautomerase [EC:5.3.2.-]	13	31	31	11	25	31
Environmental Information Processing	Membrane transport	02010 ABC transporters [PATH:ko02010]	znuB zinc transport system permease protein	24	20	31	33	17	22
Metabolism	Nucleotide metabolism	00230 Purine metabolism [PATH:ko00230]	E3.5.4.3 guaD guanine deaminase [EC:3.5.4.3]	22	19	16	16	19	36
Environmental Information	Signal transduction	02020 Two-component system [PATH:ko02020]	phoB two-component system OmpR family phosphate regulon response regulator PhoB	18	33	20	18	19	29

Processing									
Metabolism	Carbohydrate metabolism	00620 Pyruvate metabolism [PATH:ko00620]	E1.1.1.39 malate dehydrogenase (decarboxylating) [EC:1.1.1.39]	3	5	23	37	18	51
Metabolism	Metabolism of cofactors and vitamins	00670 One carbon pool by folate [PATH:ko00670]	E6.3.3.2 5-formyltetrahydrofolate cyclo-ligase [EC:6.3.3.2]	19	26	20	29	23	20
Genetic Information Processing	Folding sorting and degradation	04141 Protein processing in endoplasmic reticulum [PATH:ko04141]	STT3 dolichyl-diphosphooligosaccharide--protein glycosyltransferase [EC:2.4.99.18]	30	21	31	31	16	17
Cellular Processes	oxidative stress response	04146 Peroxisome [PATH:ko04146]	katE CAT catB srpA catalase [EC:1.11.1.6]	12	17	12	14	34	32
Metabolism	Carbohydrate metabolism	00520 Amino sugar and nucleotide sugar metabolism [PATH:ko00520]	rfbG CDP-glucose 46-dehydratase [EC:4.2.1.45]	24	20	8	47	13	21
Metabolism	Amino acid metabolism	00280 Valine leucine and isoleucine degradation [PATH:ko00280]	paaF echA enoyl-CoA hydratase [EC:4.2.1.17]	31	29	8	20	15	19
Metabolism	Amino acid metabolism	00350 Tyrosine metabolism [PATH:ko00350]	E2.3.1.- [EC:2.3.1.-]	23	18	20	18	19	29
Environmental Information Processing	Membrane transport	03070 Bacterial secretion system [PATH:ko03070]	tatB sec-independent protein translocase protein TatB	19	26	16	26	28	14
Metabolism	Carbohydrate metabolism	00520 Amino sugar and nucleotide sugar metabolism [PATH:ko00520]	E3.5.1.25 nagA AMDHD2 N-acetylglucosamine-6-phosphate deacetylase [EC:3.5.1.25]	18	21	4	30	13	33
Metabolism	Lipid metabolism	00564 Glycerophospholipid metabolism [PATH:ko00564]	cls cardiolipin synthase [EC:2.7.8.-]	19	18	8	16	27	27
Metabolism	Amino acid metabolism	00400 Phenylalanine tyrosine and tryptophan biosynthesis [PATH:ko00400]	trpG anthranilate synthase component II [EC:4.1.3.27]	29	10	23	12	35	13
Metabolism	Lipid metabolism	00071 Fatty acid metabolism [PATH:ko00071]	E1.18.1.1 rubredoxin-NAD+ reductase [EC:1.18.1.1]	12	14	23	29	29	24
Metabolism	Lipid metabolism	00564 Glycerophospholipid metabolism [PATH:ko00564]	eutB ethanolamine ammonia-lyase large subunit [EC:4.3.1.7]	31	12	27	24	10	28
Environmental Information	Membrane transport	02010 ABC transporters [PATH:ko02010]	tupA vupA tungstate transport system substrate-binding protein	19	27	8	34	17	20

Processing									
Metabolism	Carbohydrate metabolism	00650 Butanoate metabolism [PATH:ko00650]	E3.1.1.75 phaZ poly(3-hydroxybutyrate) depolymerase [EC:3.1.1.75]	35	33	23	7	17	13
Metabolism	Lipid metabolism	00120 Primary bile acid biosynthesis [PATH:ko00120]	E3.5.1.24 choloylglycine hydrolase [EC:3.5.1.24]	12	8	4	16	25	42
Metabolism	Metabolism of cofactors and vitamins	00780 Biotin metabolism [PATH:ko00780]	birA BirA family transcriptional regulator biotin operon repressor / biotin-[acetyl-CoA-carboxylase] ligase [EC:6.3.4.15]	19	23	27	29	21	18
Metabolism	Metabolism of cofactors and vitamins	00750 Vitamin B6 metabolism [PATH:ko00750]	pdxS pdx1 pyridoxine biosynthesis protein [EC:4.-.-.-]	22	21	4	19	25	22
Metabolism	Nucleotide metabolism	00230 Purine metabolism [PATH:ko00230]	amn AMP nucleosidase [EC:3.2.2.4]	10	11	12	9	37	32
Metabolism	Energy metabolism	00190 Oxidative phosphorylation [PATH:ko00190]	nuoCD NADH-quinone oxidoreductase subunit C/D [EC:1.6.5.3]	10	9	12	36	27	27
Environmental Information Processing	Energy metabolism	00190 Oxidative phosphorylation [PATH:ko00190]	ccoQ cytochrome c oxidase cbb3-type subunit IV	27	23	35	15	19	15
Cellular Processes	Cell motility	02030 Bacterial chemotaxis [PATH:ko02030]	aer aerotaxis receptor	23	24	39	5	37	5
Metabolism	Metabolism of cofactors and vitamins	00860 Porphyrin and chlorophyll metabolism [PATH:ko00860]	cobA-hemD uroporphyrinogen III methyltransferase / synthase [EC:2.1.1.107 4.2.1.75]	20	35	31	18	24	9
Metabolism	Metabolism of other amino acids	00480 Glutathione metabolism [PATH:ko00480]	gshA glutamate--cysteine ligase [EC:6.3.2.2]	24	20	27	12	32	10
Metabolism	Carbohydrate metabolism	00630 Glyoxylate and dicarboxylate metabolism [PATH:ko00630]	E1.2.1.2G formate dehydrogenase gamma subunit	21	27	62	36	5	17
Metabolism	Metabolism of cofactors and vitamins	00770 Pantothenate and CoA biosynthesis [PATH:ko00770]	E3.5.2.2 DPYS dihydropyrimidinase [EC:3.5.2.2]	21	25	20	16	20	20
Cellular Processes	Cell motility	02040 Flagellar assembly [PATH:ko02040]	fliH flagellar assembly protein FliH	19	19	16	33	13	25
Metabolism	Nucleotide metabolism	00230 Purine metabolism [PATH:ko00230]	ygeS xdhA xanthine dehydrogenase molybdenum-binding subunit [EC:1.17.1.4]	23	26	16	26	13	20

Metabolism	Amino acid metabolism	00380 Tryptophan metabolism [PATH:ko00380]	E3.5.5.1 nitrilase [EC:3.5.5.1]	24	19	23	14	24	17
Metabolism	Lipid metabolism	00564 Glycerophospholipid metabolism [PATH:ko00564]	PCYT1 choline-phosphate cytidylyltransferase [EC:2.7.7.15]	27	9	12	12	25	22
Environmental Information Processing	Membrane transport	02060 Phosphotransferase system (PTS) [PATH:ko02060]	PTS-Mtl-EIIA mtlA PTS system mannitol-specific IIA component [EC:2.7.1.69]	11	12	8	12	57	4
Metabolism	Amino acid metabolism	00400 Phenylalanine tyrosine and tryptophan biosynthesis [PATH:ko00400]	AROA1 aroA chorismate mutase [EC:5.4.99.5]	8	14	20	31	26	25
Environmental Information Processing	Membrane Transport	02010 ABC transporters [PATH:ko02010]	ABC.SN.S ssuA tauA sulfonate/nitrate/taurine transport system substrate-binding protein	24	31	20	21	11	18
Metabolism	Amino acid metabolism	00360 Phenylalanine metabolism [PATH:ko00360]	mhpC 2-hydroxy-6-ketonona-24-dienedioic acid hydrolase [EC:3.7.1.-]	46	39	27	7	1	7
Environmental Information Processing	Membrane transport	02010 ABC transporters [PATH:ko02010]	potF putrescine transport system substrate-binding protein	11	15	78	26	23	17
Environmental Information Processing	Membrane transport	02060 Phosphotransferase system (PTS) [PATH:ko02060]	PTS-Mtl-EIIB mtlA PTS system mannitol-specific IIB component [EC:2.7.1.69]	9	16	4	12	54	5
Metabolism	Carbohydrate metabolism	00620 Pyruvate metabolism [PATH:ko00620]	E1.2.1.10 acetaldehyde dehydrogenase (acetylating) [EC:1.2.1.10]	17	36	12	5	10	31
Metabolism	Carbohydrate metabolism	00040 Pentose and glucuronate interconversions [PATH:ko00040]	sgbU hexulose-6-phosphate isomerase [EC:5.-.-.-]	13	27	27	20	31	8
Metabolism	Amino acid metabolism	00260 Glycine serine and threonine metabolism [PATH:ko00260]	glxK glycerate kinase [EC:2.7.1.31]	19	19	39	19	20	17
Human Diseases	Infectious diseases	05100 Bacterial invasion of epithelial cells [PATH:ko05100]	sfb1 fibronectin-binding protein 1	27	26	27	18	17	10
Metabolism	Carbohydrate metabolism	00030 Pentose phosphate pathway [PATH:ko00030]	E4.1.2.9 phosphoketolase [EC:4.1.2.9]	17	31	16	11	10	30
Metabolism	Carbohydrate metabolism	00520 Amino sugar and nucleotide sugar metabolism	arnC pmrF undecaprenyl-phosphate 4-deoxy-4-formamido-L-arabinose transferase [EC:2.7.8.30]	18	26	12	19	20	15

		[PATH:ko00520]							
Environmental Information Processing	Membrane transport	02010 ABC transporters [PATH:ko02010]	modA molybdate transport system substrate-binding protein	20	26	35	18	16	15
Environmental Information Processing	Signal transduction	02020 Two-component system [PATH:ko02020]	qseB two-component system OmpR family response regulator QseB	31	16	12	1	30	8
Environmental Information Processing	Signal transduction	02020 Two-component system [PATH:ko02020]	barA two-component system NarL family sensor histidine kinase BarA [EC:2.7.13.3]	18	20	20	24	9	27
Metabolism	Glycan biosynthesis and metabolism	00540 Lipopolysaccharide biosynthesis [PATH:ko00540]	gmhB D-glycero-D-manno-heptose 17-bisphosphate phosphatase [EC:3.1.3.82 3.1.3.83]	25	20	23	8	22	14
Environmental Information Processing	Membrane transport	02010 ABC transporters [PATH:ko02010]	yejB microcin C transport system permease protein	21	9	23	24	20	19
Metabolism	Biosynthesis of other secondary metabolites	00940 Phenylpropanoid biosynthesis [PATH:ko00940]	bglB beta-glucosidase [EC:3.2.1.21]	9	46	35	8	19	13
Metabolism	Amino acid metabolism	00250 Alanine aspartate and glutamate metabolism [PATH:ko00250]	glsA GLS glutaminase [EC:3.5.1.2]	4	11	4	31	30	20
Environmental Information Processing	Membrane transport	02010 ABC transporters [PATH:ko02010]	phnC phosphonate transport system ATP-binding protein [EC:3.6.3.28]	17	22	47	31	8	13
Metabolism	Amino acid metabolism	00260 Glycine serine and threonine metabolism [PATH:ko00260]	ttuD hydroxypyruvate reductase [EC:1.1.1.81]	19	15	8	21	19	17
Metabolism	Nucleotide metabolism	00230 Purine metabolism [PATH:ko00230]	E2.1.2.3 phosphoribosylaminoimidazolecarboxamide formyltransferase [EC:2.1.2.3]	14	16	12	19	16	25
Metabolism	Amino acid metabolism	00400 Phenylalanine tyrosine and tryptophan biosynthesis [PATH:ko00400]	trpGD anthranilate synthase/phosphoribosyltransferase [EC:4.1.3.27 2.4.2.18]	17	16	39	29	14	15
Metabolism	Amino acid metabolism	00280 Valine leucine and isoleucine degradation [PATH:ko00280]	mmsA iolA ALDH6A1 malonate-semialdehyde dehydrogenase (acetylating) / methylmalonate-semialdehyde dehydrogenase [EC:1.2.1.18 1.2.1.27]	31	27	35	10	9	8

Environmental Information Processing	Membrane transport	02010 ABC transporters [PATH:ko02010]	ABC-2.CPSE.A capsular polysaccharide transport system ATP-binding protein	24	14	20	4	32	4
Cellular Processes	Transport and catabolism	04142 Lysosome [PATH:ko04142]	E3.2.1.25 MANBA manB beta-mannosidase [EC:3.2.1.25]	8	8	8	9	39	17
Metabolism	Amino acid metabolism	00280 Valine leucine and isoleucine degradation [PATH:ko00280]	E1.2.4.4C bkdA 2-oxoisovalerate dehydrogenase E1 component [EC:1.2.4.4]	19	11	27	22	10	23
Metabolism	Nucleotide metabolism	00230 Purine metabolism [PATH:ko00230]	E1.7.1.7 guaC GMP reductase [EC:1.7.1.7]	33	23	16	7	11	9
Environmental Information Processing	Signal transduction	02020 Two-component system [PATH:ko02020]	atoC two-component system NtrC family response regulator AtoC	4	5	16	50	20	18
Metabolism	Amino acid metabolism	00270 Cysteine and methionine metabolism [PATH:ko00270]	E2.5.1.16 SRM speE spermidine synthase [EC:2.5.1.16]	11	20	4	5	30	16
Metabolism	Carbohydrate metabolism	00052 Galactose metabolism [PATH:ko00052]	E3.2.1.10 oligo-16-glucosidase [EC:3.2.1.10]	19	11	20	18	19	17
Human Diseases	Nucleotide metabolism	00240 Pyrimidine metabolism [PATH:ko00240]	E2.4.2.4 deoA thymidine phosphorylase [EC:2.4.2.4]	21	31	8	7	5	23
Metabolism	Carbohydrate metabolism	00630 Glyoxylate and dicarboxylate metabolism [PATH:ko00630]	glcF glycolate oxidase iron-sulfur subunit	20	22	12	18	8	20
Environmental Information Processing	Membrane transport	02010 ABC transporters [PATH:ko02010]	hlyB cyaB ATP-binding cassette subfamily B bacterial HlyB/CyaB	17	18	55	10	22	8
Metabolism	Carbohydrate metabolism	00500 Starch and sucrose metabolism [PATH:ko00500]	treS maltose alpha-D-glucosyltransferase/ alpha-amylase [EC:5.4.99.16 3.2.1.1]	14	14	12	23	17	18
Environmental Information Processing	Membrane transport	02010 ABC transporters [PATH:ko02010]	yejE microcin C transport system permease protein	10	13	12	25	20	19
Metabolism	Amino acid metabolism	00330 Arginine and proline metabolism [PATH:ko00330]	E2.6.1.82 putrescine aminotransferase [EC:2.6.1.82]	15	16	8	8	10	32
Metabolism	Carbohydrate metabolism	00052 Galactose metabolism [PATH:ko00052]	bgaB lacA beta-galactosidase [EC:3.2.1.23]	17	10	4	26	18	16

Metabolism	Carbohydrate Metabolism	00640 Propanoate metabolism [PATH:ko00640]	mmdB methylmalonyl-CoA decarboxylase beta chain [EC:4.1.1.41]	6	12	8	26	20	23
Metabolism	Carbohydrate metabolism	00010 Glycolysis / Gluconeogenesis [PATH:ko00010]	K01622 fructose 16-bisphosphate aldolase/phosphatase [EC:4.1.2.13 3.1.3.11]	9	12	4	9	26	23
Environmental Information Processing	Signal transduction	02020 Two-component system [PATH:ko02020]	spo0A two-component system response regulator stage 0 sporulation protein A	7	15	8	25	15	26
Metabolism	Amino acid metabolism	00260 Glycine serine and threonine metabolism [PATH:ko00260]	thrB2 homoserine kinase type II [EC:2.7.1.39]	23	24	20	1	21	8
Genetic Information Processing	Replication and repair	03430 Mismatch repair [PATH:ko03430]	xseB exodeoxyribonuclease VII small subunit [EC:3.1.11.6]	18	16	23	15	17	14
Environmental Information Processing	Nitrogen metabolism	00910 Nitrogen metabolism [PATH:ko00910]	nifA Nif-specific regulatory protein	12	27	4	13	16	17
Metabolism	Amino acid metabolism	00260 Glycine serine and threonine metabolism [PATH:ko00260]	E4.2.1.22 CBS cystathionine beta-synthase [EC:4.2.1.22]	16	43	8	11	8	11
Metabolism	Amino acid metabolism	00400 Phenylalanine tyrosine and tryptophan biosynthesis [PATH:ko00400]	trpF phosphoribosylanthranilate isomerase [EC:5.3.1.24]	21	8	8	11	19	18
Metabolism	Xenobiotics biodegradation and metabolism	00633 Nitrotoluene degradation [PATH:ko00633]	E1.12.99.6S hydrogenase small subunit [EC:1.12.99.6]	14	25	12	11	16	15
Metabolism	Energy metabolism	00680 Methane metabolism [PATH:ko00680]	E1.2.99.2S coxS carbon-monoxide dehydrogenase small subunit [EC:1.2.99.2]	15	20	27	35	8	11
Metabolism	Energy metabolism	00190 Oxidative phosphorylation [PATH:ko00190]	E1.6.99.5 NADH dehydrogenase (quinone) [EC:1.6.99.5]	15	29	23	18	6	16
Environmental Information Processing	Signal transduction	02020 Two-component system [PATH:ko02020]	hydG zraR two-component system NtrC family response regulator HydG	11	11	12	20	11	29
Metabolism	Glycan biosynthesis and metabolism	00550 Peptidoglycan biosynthesis [PATH:ko00550]	pbpC penicillin-binding protein 1C [EC:2.4.1.-]	13	27	20	22	14	9
Metabolism	Glycan biosynthesis and metabolism	00540 Lipopolysaccharide biosynthesis [PATH:ko00540]	waaF rfaF heptosyltransferase II [EC:2.4.-.-]	11	12	31	12	24	15

Genetic Information Processing	Replication and repair	03420 Nucleotide excision repair [PATH:ko03420]	ERCC3 XPB DNA excision repair protein ERCC-3 [EC:3.6.4.12]	17	12	20	21	13	17
Metabolism	Metabolism of cofactors and vitamins	00770 Pantothenate and CoA biosynthesis [PATH:ko00770]	coaE dephospho-CoA kinase [EC:2.7.1.24]	8	9	12	21	21	21
Metabolism	Carbohydrate metabolism	00040 Pentose and glucuronate interconversions [PATH:ko00040]	rhaD rhamnulose-1-phosphate aldolase [EC:4.1.2.19]	12	9	23	25	14	18
Genetic Information Processing	Translation	00970 Aminoacyl-tRNA biosynthesis [PATH:ko00970]	selA L-seryl-tRNA(Ser) seleniumtransferase [EC:2.9.1.1]	11	24	23	8	14	20
Environmental Information Processing	Membrane transport	02060 Phosphotransferase system (PTS) [PATH:ko02060]	PTS-Ula-EIIA ulaC sgaA PTS system ascorbate-specific IIA component [EC:2.7.1.69]	9	23	12	22	24	4
Environmental Information Processing	Signal transduction	02020 Two-component system [PATH:ko02020]	crp CRP/FNR family transcriptional regulator cyclic AMP receptor protein	11	9	8	15	32	9
Metabolism	Nucleotide metabolism	00230 Purine metabolism [PATH:ko00230]	ppx-gppA exopolyphosphatase / guanosine-5'-triphosphate3'-diphosphate pyrophosphatase [EC:3.6.1.11 3.6.1.40]	17	15	27	20	16	10
Metabolism	Amino acid metabolism	00350 Tyrosine metabolism [PATH:ko00350]	E4.1.99.2 tyrosine phenol-lyase [EC:4.1.99.2]	15	18	23	10	15	17
Metabolism	Metabolism of cofactors and vitamins	00760 Nicotinate and nicotinamide metabolism [PATH:ko00760]	ushA 5'-nucleotidase / UDP-sugar diphosphatase [EC:3.1.3.5 3.6.1.45]	17	14	12	20	17	11
Metabolism	Carbohydrate metabolism	00650 Butanoate metabolism [PATH:ko00650]	gctB glutaconate CoA-transferase subunit B [EC:2.8.3.12]	20	17	43	21	8	10
Environmental Information Processing	Energy metabolism	00910 Nitrogen metabolism [PATH:ko00910]	narI nitrate reductase 1 gamma subunit [EC:1.7.99.4]	19	22	16	19	13	8
Metabolism	Nitrogen metabolism	00910 Nitrogen metabolism [PATH:ko00910]	nifH nitrogenase iron protein NifH [EC:1.18.6.1]	9	20	8	15	20	15
Metabolism	Metabolism of cofactors and vitamins	00760 Nicotinate and nicotinamide metabolism [PATH:ko00760]	yfbR 5'-nucleotidase [EC:3.1.3.5]	12	17	43	21	9	17
Cellular Processes	Cell growth and death	04112 Cell cycle - Caulobacter [PATH:ko04112]	ccrM modification methylase [EC:2.1.1.72]	17	13	12	4	23	13

Metabolism	Amino acid metabolism	00250 Alanine aspartate and glutamate metabolism [PATH:ko00250]	gabT 4-aminobutyrate aminotransferase / (S)-3-amino-2-methylpropionate transaminase [EC:2.6.1.19 2.6.1.22]	15	16	20	18	13	14
Cellular Processes	Cell growth and death	04112 Cell cycle - Caulobacter [PATH:ko04112]	pleD two-component system cell cycle response regulator	14	25	8	11	14	15
Metabolism	Metabolism of cofactors and vitamins	00790 Folate biosynthesis [PATH:ko00790]	folK 2-amino-4-hydroxy-6-hydroxymethyldihydropteridine diphosphokinase [EC:2.7.6.3]	15	15	12	16	19	10
Metabolism	Amino acid metabolism	00340 Histidine metabolism [PATH:ko00340]	hisB imidazoleglycerol-phosphate dehydratase / histidinol-phosphatase [EC:4.2.1.19 3.1.3.15]	8	8	12	15	14	27
Metabolism	Metabolism of cofactors and vitamins	00860 Porphyrin and chlorophyll metabolism [PATH:ko00860]	cbiD cobalt-precorrin-5B (C1)-methyltransferase [EC:2.1.1.195]	7	13	16	19	18	18
Environmental Information Processing	Membrane transport	02010 ABC transporters [PATH:ko02010]	yejF microcin C transport system ATP-binding protein	17	15	4	9	18	12
Metabolism	Metabolism of cofactors and vitamins	00860 Porphyrin and chlorophyll metabolism [PATH:ko00860]	E2.5.1.17 cobO btuR cob(I)alamin adenosyltransferase [EC:2.5.1.17]	18	6	27	18	11	15
Metabolism	Metabolism of cofactors and vitamins	00860 Porphyrin and chlorophyll metabolism [PATH:ko00860]	cobN cobaltochelatase CobN [EC:6.6.1.2]	18	21	8	10	15	8
Metabolism	Amino acid metabolism	00270 Cysteine and methionine metabolism [PATH:ko00270]	metZ O-succinylhomoserine sulfhydrylase [EC:2.5.1.-]	12	22	35	2	18	11
Metabolism	Glycan biosynthesis and metabolism	00540 Lipopolysaccharide biosynthesis [PATH:ko00540]	waaC rfaC heptosyltransferase I [EC:2.4.-.-]	19	15	23	10	16	8
Metabolism	Energy metabolism	00680 Methane metabolism [PATH:ko00680]	hdrC heterodisulfide reductase subunit C [EC:1.8.98.1]	12	20	4	15	13	13
Environmental Information Processing	Membrane transport	02060 Phosphotransferase system (PTS) [PATH:ko02060]	PTS-Cel-EIIB celA PTS system cellobiose-specific IIB component [EC:2.7.1.69]	14	2	8	16	16	18
Metabolism	Metabolism of cofactors and vitamins	00130 Ubiquinone and other terpenoid-quinone biosynthesis [PATH:ko00130]	menA 14-dihydroxy-2-naphthoate octaprenyltransferase [EC:2.5.1.74 2.5.1.-]	15	16	8	8	19	10

Genetic Information Processing	Folding sorting and degradation	04122 Sulfur relay system [PATH:ko04122]	thiF sulfur carrier protein ThiS adenylyltransferase [EC:2.7.7.73]	8	13	27	22	16	11
Environmental Information Processing	Membrane transport	03070 Bacterial secretion system [PATH:ko03070]	virB4 lvhB4 type IV secretion system protein VirB4	42	7	12	1	7	3
Environmental Information Processing	Membrane transport	02010 ABC transporters [PATH:ko02010]	lptG lipopolysaccharide export system permease protein	17	25	31	9	12	4
Cellular Processes	Cell motility	02040 Flagellar assembly [PATH:ko02040]	flgF flagellar basal-body rod protein FlgF	2	1	8	19	25	20
Environmental Information Processing	Signal transduction	02020 Two-component system [PATH:ko02020]	phoB1 phoP two-component system OmpR family alkaline phosphatase synthesis response regulator PhoP	15	22	8	14	9	11
Environmental Information Processing	Membrane transport	03070 Bacterial secretion system [PATH:ko03070]	vasG clpV type VI secretion system protein VasG	9	14	4	25	14	11
Metabolism	Amino acid metabolism	00250 Alanine aspartate and glutamate metabolism [PATH:ko00250]	E2.6.1.1B aspC aspartate aminotransferase [EC:2.6.1.1]	3	32	12	7	21	8
Metabolism	Nucleotide metabolism	00240 Pyrimidine metabolism [PATH:ko00240]	E4.2.1.70 pseudouridylate synthase [EC:4.2.1.70]	10	6	23	10	23	12
Environmental Information Processing	Energy metabolism	00910 Nitrogen metabolism [PATH:ko00910]	narH nitrate reductase 1 beta subunit [EC:1.7.99.4]	22	15	27	9	13	4
Environmental Information Processing	Membrane transport	02010 ABC transporters [PATH:ko02010]	ABC-2.CPSE.P capsular polysaccharide transport system permease protein	16	13	20	1	23	6
Metabolism	Carbohydrate metabolism	00040 Pentose and glucuronate interconversions [PATH:ko00040]	E1.1.1.125 kduD 2-deoxy-D-gluconate 3-dehydrogenase [EC:1.1.1.125]	14	17	4	9	13	15
Metabolism	Amino Acid Metabolism	00330 Arginine and proline metabolism [PATH:ko00330]	lysJ acetylornithine/acetyl-lysine aminotransferase [EC:2.6.1.11 2.6.1.-]	10	7	16	10	16	19
Metabolism	Carbohydrate metabolism	00030 Pentose phosphate pathway [PATH:ko00030]	pgl 6-phosphogluconolactonase [EC:3.1.1.31]	15	12	20	11	13	12

Environmental Information Processing	Signal transduction	02020 Two-component system [PATH:ko02020]	pilI twitching motility protein PilI	20	23	4	8	14	3
Metabolism	Nucleotide metabolism	00230 Purine metabolism [PATH:ko00230]	gpt xanthine phosphoribosyltransferase [EC:2.4.2.22]	18	12	12	10	13	11
Environmental Information Processing	Membrane transport	02010 ABC transporters [PATH:ko02010]	thiB tbpA thiamine transport system substrate-binding protein	5	5	39	20	18	14
Metabolism	Metabolism of cofactors and vitamins	00780 Biotin metabolism [PATH:ko00780]	bioA adenosylmethionine-8-amino-7-oxononanoate aminotransferase [EC:2.6.1.62]	13	12	20	10	14	13
Environmental Information Processing	Signal transduction	02020 Two-component system [PATH:ko02020]	ntrX two-component system NtrC family nitrogen regulation response regulator NtrX	16	11	4	13	17	8
Environmental Information Processing	Signal transduction	04070 Phosphatidylinositol signaling system [PATH:ko04070]	E2.7.1.107 DGK dgkA diacylglycerol kinase (ATP dependent) [EC:2.7.1.107]	7	13	20	14	22	7
Metabolism	Amino acid metabolism	00340 Histidine metabolism [PATH:ko00340]	E3.1.3.15B histidinol-phosphatase (PHP family) [EC:3.1.3.15]	9	15	20	21	10	13
Human Diseases	Infectious diseases	02026 Biofilm formation - Escherichia coli [PATH:ko02026]	SIG2 rpoS RNA polymerase nonessential primary-like sigma factor	13	14	23	15	10	11
Metabolism	Energy metabolism	00910 Nitrogen metabolism [PATH:ko00910]	cah carbonic anhydrase [EC:4.2.1.1]	17	22	4	10	7	11
Environmental Information Processing	Signal transduction	02020 Two-component system [PATH:ko02020]	glnD [protein-PII] uridylyltransferase [EC:2.7.7.59]	21	6	12	5	19	6
Metabolism	Metabolism of cofactors and vitamins	00130 Ubiquinone and other terpenoid-quinone biosynthesis [PATH:ko00130]	ubiX 3-octaprenyl-4-hydroxybenzoate carboxy-lyase UbiX [EC:4.1.1.-]	11	16	51	8	15	7
Metabolism	Carbohydrate metabolism	00620 Pyruvate metabolism [PATH:ko00620]	aldA lactaldehyde dehydrogenase / glycolaldehyde dehydrogenase [EC:1.2.1.22 1.2.1.21]	18	7	12	16	7	14
Human Diseases	Infectious diseases	05111 Vibrio cholerae pathogenic cycle [PATH:ko05111]	mshB MSHA pilin protein MshB	4	15	4	19	20	10
Environmental Information	Membrane transport	03070 Bacterial secretion system [PATH:ko03070]	virD4 lvhD4 type IV secretion system protein VirD4	21	13	4	15	9	6

Processing									
Metabolism	Xenobiotics biodegradation and metabolism	00625 Chloroalkane and chloroalkene degradation [PATH:ko00625]	E3.8.1.- [EC:3.8.1.-]	10	15	27	7	16	10
Genetic Information Processing	Translation	00970 Aminoacyl-tRNA biosynthesis [PATH:ko00970]	gltX1 nondiscriminating glutamyl-tRNA synthetase [EC:6.1.1.24]	20	16	8	5	4	15
Genetic Information Processing	Folding sorting and degradation	04122 Sulfur relay system [PATH:ko04122]	thiS sulfur carrier protein	17	18	8	7	8	12
Metabolism	Metabolism of cofactors and vitamins	00860 Porphyrin and chlorophyll metabolism [PATH:ko00860]	cbiB cobD adenosylcobinamide-phosphate synthase [EC:6.3.1.10]	12	13	20	7	13	13
Environmental Information Processing	Signal transduction	02020 Two-component system [PATH:ko02020]	pilL type IV pili sensor histidine kinase and response regulator	20	16	20	2	16	2
Cellular Processes	Cell motility	02040 Flagellar assembly [PATH:ko02040]	flgH flagellar L-ring protein precursor FlgH	8	8	12	18	18	9
Metabolism	Carbohydrate metabolism	00052 Galactose metabolism [PATH:ko00052]	melA alpha-galactosidase [EC:3.2.1.22]	20	6	12	12	5	14
Cellular Processes	Transport and catabolism	04146 Peroxisome [PATH:ko04146]	E3.6.1.22 NUDT12 nudC NAD+ diphosphatase [EC:3.6.1.22]	11	12	8	10	10	17
Metabolism	Nucleotide metabolism	00230 Purine metabolism [PATH:ko00230]	ygeT xdhB xanthine dehydrogenase FAD-binding subunit [EC:1.17.1.4]	13	20	4	12	10	9
Metabolism	Amino acid metabolism	00290 Valine leucine and isoleucine biosynthesis [PATH:ko00290]	cimA D-citramalate synthase [EC:2.3.1.182]	8	5	4	24	7	19
Environmental Information Processing	Membrane transport	02010 ABC transporters [PATH:ko02010]	potG putrescine transport system ATP-binding protein	10	8	12	3	23	8
Metabolism	Carbohydrate metabolism	00051 Fructose and mannose metabolism [PATH:ko00051]	GMPP mannose-1-phosphate guanylyltransferase [EC:2.7.7.13]	9	7	4	7	10	23
Environmental Information Processing	Signal transduction	02020 Two-component system [PATH:ko02020]	citD citrate lyase subunit gamma [EC:4.1.3.6]	9	9	16	13	14	10
Metabolism	Carbohydrate metabolism	00520 Amino sugar and nucleotide sugar metabolism [PATH:ko00520]	E4.1.3.3 nanA NPL N-acetylneuraminate lyase [EC:4.1.3.3]	12	17	12	14	7	10
Metabolism	Metabolism of cofactors and vitamins	00760 Nicotinate and nicotinamide metabolism [PATH:ko00760]	iunH purine nucleosidase [EC:3.2.2.1]	8	14	4	2	18	12

Metabolism	Lipid metabolism	00561 Glycerolipid metabolism [PATH:ko00561]	pduP propionaldehyde dehydrogenase	11	11	8	16	7	13
Genetic Information Processing	Replication and repair	03440 Homologous recombination [PATH:ko03440]	recB exodeoxyribonuclease V beta subunit [EC:3.1.11.5]	12	9	16	7	14	10
Environmental Information Processing	Signal transduction	02020 Two-component system [PATH:ko02020]	ompR two-component system OmpR family phosphate regulon response regulator OmpR	16	11	43	14	7	3
Metabolism	Lipid metabolism	00564 Glycerophospholipid metabolism [PATH:ko00564]	pgpA phosphatidylglycerophosphatase A [EC:3.1.3.27]	8	8	8	12	14	12
Metabolism	Metabolism of cofactors and vitamins	00860 Porphyrin and chlorophyll metabolism [PATH:ko00860]	E2.1.1.133 cobM cbiF precorrin-4 C11-methyltransferase [EC:2.1.1.133]	11	15	12	2	9	15
Environmental Information Processing	Signal transduction	02020 Two-component system [PATH:ko02020]	dctM C4-dicarboxylate transporter DctM subunit	12	12	8	14	8	11
Metabolism	Amino acid metabolism	00260 Glycine serine and threonine metabolism [PATH:ko00260]	dsdA D-serine dehydratase [EC:4.3.1.18]	17	15	12	5	4	11
Metabolism	Metabolism of terpenoids and polyketides	00900 Terpenoid backbone biosynthesis [PATH:ko00900]	hepST heptaprenyl diphosphate synthase [EC:2.5.1.30]	13	14	23	16	7	4
Metabolism	Energy metabolism	00910 Nitrogen metabolism [PATH:ko00910]	nosZ nitrous-oxide reductase [EC:1.7.2.4]	5	8	12	19	8	16
Metabolism	Glycan biosynthesis and metabolism	00511 Other glycan degradation [PATH:ko00511]	E3.2.1.24 alpha-mannosidase [EC:3.2.1.24]	7	10	4	18	8	15
Metabolism	Amino acid metabolism	00330 Arginine and proline metabolism [PATH:ko00330]	E4.3.1.12 ocd ornithine cyclodeaminase [EC:4.3.1.12]	9	3	8	8	21	8
Metabolism	Amino acid metabolism	00260 Glycine serine and threonine metabolism [PATH:ko00260]	E4.4.1.1 cystathionine gamma-lyase [EC:4.4.1.1]	9	10	8	25	6	10
Environmental Information Processing	Signal transduction	04066 HIF-1 signaling pathway [PATH:ko04066]	PDHB pdhB pyruvate dehydrogenase E1 component subunit beta [EC:1.2.4.1]	12	10	23	13	4	12
Environmental Information Processing	Membrane transport	02010 ABC transporters [PATH:ko02010]	yejA microcin C transport system substrate-binding protein	12	10	4	5	18	5
Metabolism	Energy metabolism	00710 Carbon fixation in photosynthetic organisms	E4.1.2.22 fructose-6-phosphate phosphoketolase [EC:4.1.2.22]	9	11	4	5	8	17

		[PATH:ko00710]							
Metabolism	Metabolism of cofactors and vitamins	00770 Pantothenate and CoA biosynthesis [PATH:ko00770]	acpS holo-[acyl-carrier protein] synthase [EC:2.7.8.7]	11	5	12	12	16	6
Metabolism	Metabolism of cofactors and vitamins	00860 Porphyrin and chlorophyll metabolism [PATH:ko00860]	cobL precorrin-6Y C515-methyltransferase (decarboxylating) [EC:2.1.1.132]	5	4	4	25	9	13
Environmental Information Processing	Signal transduction	02020 Two-component system [PATH:ko02020]	resD two-component system OmpR family response regulator ResD	9	15	4	9	13	8
Metabolism	Glycan biosynthesis and metabolism	00550 Peptidoglycan biosynthesis [PATH:ko00550]	dacB D-alanyl-D-alanine carboxypeptidase / D-alanyl-D-alanine-endopeptidase (penicillin-binding protein 4) [EC:3.4.16.4 3.4.21.-]	10	13	4	11	8	10
Cellular Processes	Cell motility	02040 Flagellar assembly [PATH:ko02040]	flhD flagellar transcriptional activator FlhD	20	11	23	3	5	6
Genetic Information Processing	Folding sorting and degradation	04122 Sulfur relay system [PATH:ko04122]	moaD molybdopterin synthase sulfur carrier subunit	10	18	16	12	5	8
Metabolism	Energy metabolism	00680 Methane metabolism [PATH:ko00680]	E2.7.1.29 DAK1 DAK2 dihydroxyacetone kinase [EC:2.7.1.29]	9	29	4	9	2	6
Metabolism	Carbohydrate metabolism	00562 Inositol phosphate metabolism [PATH:ko00562]	iolB 5-deoxy-glucuronate isomerase [EC:5.3.1.-]	9	14	23	9	5	10
Metabolism	Carbohydrate metabolism	00040 Pentose and glucuronate interconversions [PATH:ko00040]	E3.1.1.11 pectinesterase [EC:3.1.1.11]	4	11	4	24	11	5
Metabolism	Lipid metabolism	00564 Glycerophospholipid metabolism [PATH:ko00564]	glpC glycerol-3-phosphate dehydrogenase subunit C [EC:1.1.5.3]	9	13	20	12	5	10
Metabolism	Carbohydrate metabolism	00053 Ascorbate and aldarate metabolism [PATH:ko00053]	gudD glucarate dehydratase [EC:4.2.1.40]	10	8	4	19	7	8
Metabolism	Amino acid metabolism	00280 Valine leucine and isoleucine degradation [PATH:ko00280]	E2.8.3.5A scoA 3-oxoacid CoA-transferase subunit A [EC:2.8.3.5]	14	13	12	11	5	6

Genetic Information Processing	Folding sorting and degradation	04122 Sulfur relay system [PATH:ko04122]	MOCS2 moaE molybdopterin synthase catalytic subunit [EC:2.-.-.-]	8	22	27	9	4	6
Environmental Information Processing	Membrane transport	02010 ABC transporters [PATH:ko02010]	modF molybdate transport system ATP-binding protein	3	9	4	10	8	18
Environmental Information Processing	Amino acid metabolism	00480 Glutathione metabolism [PATH:ko00480]	pepD putative serine protease PepD [EC:3.4.21.-]	10	15	27	7	7	7
Environmental Information Processing	Signal transduction	04066 HIF-1 signaling pathway [PATH:ko04066]	PDHA pdhA pyruvate dehydrogenase E1 component subunit alpha [EC:1.2.4.1]	13	10	16	4	5	11
Environmental Information Processing	Membrane transport	02060 Phosphotransferase system (PTS) [PATH:ko02060]	PTS-Cel-EIIC celB PTS system cellobiose-specific IIC component	5	4	20	4	7	20
Metabolism	Energy metabolism	00920 Sulfur metabolism [PATH:ko00920]	cysD sulfate adenylyltransferase subunit 2 [EC:2.7.7.4]	15	12	4	13	6	3
Metabolism	Carbohydrate metabolism	00010 Glycolysis / Gluconeogenesis [PATH:ko00010]	E1.1.1.2 adh alcohol dehydrogenase (NADP+) [EC:1.1.1.2]	8	17	4	9	11	4
Metabolism	Energy metabolism	00680 Methane metabolism [PATH:ko00680]	E1.12.1.2 hydrogen dehydrogenase [EC:1.12.1.2]	4	24	8	10	7	6
Metabolism	Carbohydrate metabolism	00630 Glyoxylate and dicarboxylate metabolism [PATH:ko00630]	E1.4.7.1 glutamate synthase (ferredoxin) [EC:1.4.7.1]	6	10	16	16	7	9
Metabolism	Metabolism of cofactors and vitamins	00860 Porphyrin and chlorophyll metabolism [PATH:ko00860]	E5.4.1.2 cobH cbiC precorrin-8X methylmutase [EC:5.4.1.2]	3	18	16	10	7	11
Metabolism	Glycan biosynthesis and metabolism	00550 Peptidoglycan biosynthesis [PATH:ko00550]	mtgA monofunctional biosynthetic peptidoglycan transglycosylase [EC:2.4.1.-]	5	4	4	10	11	15
Metabolism	Metabolism of cofactors and vitamins	00770 Pantothenate and CoA biosynthesis [PATH:ko00770]	panE apbA 2-dehydropantoate 2-reductase [EC:1.1.1.169]	2	12	4	13	8	13
Genetic Information Processing	Replication and repair	03410 Base excision repair [PATH:ko03410]	tag DNA-3-methyladenine glycosylase I [EC:3.2.2.20]	5	3	16	11	10	13
Metabolism	Metabolism of cofactors and vitamins	00860 Porphyrin and chlorophyll metabolism [PATH:ko00860]	E2.1.1.131 cobJ cbiH precorrin-3B C17-methyltransferase [EC:2.1.1.131]	7	12	8	10	5	13
Metabolism	Metabolism of cofactors and vitamins	00130 Ubiquinone and other terpenoid-quinone biosynthesis [PATH:ko00130]	entC isochorismate synthase [EC:5.4.4.2]	3	10	23	5	14	8

Environmental Information Processing	Signal transduction	02020 Two-component system [PATH:ko02020]	regX3 two-component system OmpR family response regulator RegX3	12	11	8	9	6	8
Metabolism	Metabolism of cofactors and vitamins	00760 Nicotinate and nicotinamide metabolism [PATH:ko00760]	E3.1.3.5 5'-nucleotidase [EC:3.1.3.5]	8	13	12	9	7	8
Metabolism	Lipid metabolism	00561 Glycerolipid metabolism [PATH:ko00561]	dhaL dihydroxyacetone kinase C-terminal domain [EC:2.7.1.-]	7	17	8	8	5	11
Cellular Processes	Cell growth and death	04112 Cell cycle - Caulobacter [PATH:ko04112]	ftsQ cell division protein FtsQ	5	9	8	8	11	11
Metabolism	Metabolism of cofactors and vitamins	00785 Lipoic acid metabolism [PATH:ko00785]	lipB lipoyl(octanoyl) transferase [EC:2.3.1.181]	8	9	12	5	11	8
Metabolism	Carbohydrate metabolism	00010 Glycolysis / Gluconeogenesis [PATH:ko00010]	agp glucose-1-phosphatase [EC:3.1.3.10]	6	12	4	12	8	8
Metabolism	Carbohydrate metabolism	00650 Butanoate metabolism [PATH:ko00650]	E4.2.1.- [EC:4.2.1.-]	9	9	12	12	5	7
Environmental Information Processing	Membrane transport	02010 ABC transporters [PATH:ko02010]	lacF araP lactose/L-arabinose transport system permease protein	11	11	4	5	7	7
Environmental Information Processing	Membrane transport	02010 ABC transporters [PATH:ko02010]	phnE phosphonate transport system permease protein	8	22	4	7	5	4
Environmental Information Processing	Signal transduction	02020 Two-component system [PATH:ko02020]	pilS two-component system NtrC family sensor histidine kinase PilS [EC:2.7.13.3]	5	7	8	3	19	4
Genetic Information Processing	Folding sorting and degradation	03018 RNA degradation [PATH:ko03018]	rhlB ATP-dependent RNA helicase RhlB [EC:3.6.4.13]	6	12	4	7	13	5
Environmental Information Processing	Membrane transport	02010 ABC transporters [PATH:ko02010]	cysA sulfate transport system ATP-binding protein [EC:3.6.3.25]	6	19	12	7	4	8
Cellular Processes	Cell motility	02040 Flagellar assembly [PATH:ko02040]	fliQ flagellar biosynthetic protein FliQ	6	12	16	14	6	5
Environmental Information Processing	Signal transduction	02020 Two-component system [PATH:ko02020]	flrC fleR two component system response regulator FlrC	7	2	20	4	14	7
Metabolism	Amino acid metabolism	00400 Phenylalanine tyrosine and tryptophan biosynthesis [PATH:ko00400]	pheC cyclohexadienyl dehydratase [EC:4.2.1.51 4.2.1.91]	11	16	4	7	5	4

Metabolism	Lipid metabolism	00564 Glycerophospholipid metabolism [PATH:ko00564]	pldA phospholipase A1 [EC:3.1.1.32 3.1.1.4]	7	4	23	5	7	12
Metabolism	Lipid metabolism	00564 Glycerophospholipid metabolism [PATH:ko00564]	eutC ethanolamine ammonia-lyase small subunit [EC:4.3.1.7]	7	8	4	10	2	14
Genetic Information Processing	Replication and repair	03440 Homologous recombination [PATH:ko03440]	recC exodeoxyribonuclease V gamma subunit [EC:3.1.11.5]	8	11	20	2	7	8
Metabolism	Lipid metabolism	00561 Glycerolipid metabolism [PATH:ko00561]	E3.1.1.3 triacylglycerol lipase [EC:3.1.1.3]	6	10	20	7	8	5
Environmental Information Processing	Membrane transport	02010 ABC transporters [PATH:ko02010]	modB molybdate transport system permease protein	7	8	8	3	7	10
Metabolism	Xenobiotics biodegradation and metabolism	00930 Caprolactam degradation [PATH:ko00930]	DCAA acyl-CoA dehydrogenase [EC:1.3.99.-]	9	12	8	9	1	8
Metabolism	Amino acid metabolism	00270 Cysteine and methionine metabolism [PATH:ko00270]	dcyD D-cysteine desulfhydrase [EC:4.4.1.15]	7	5	4	10	4	12
Environmental Information Processing	Membrane transport	02010 ABC transporters [PATH:ko02010]	ABC.CEB.A msiK cellobiose transport system ATP-binding protein	9	10	4	4	5	7
Metabolism	Amino acid metabolism	00330 Arginine and proline metabolism [PATH:ko00330]	E3.5.3.12 agmatine deiminase [EC:3.5.3.12]	4	7	8	4	10	10
Environmental Information Processing	Membrane transport	03070 Bacterial secretion system [PATH:ko03070]	yscV type III secretion protein SctV	6	8	4	3	7	11
Cellular Processes	Cell motility	02040 Flagellar assembly [PATH:ko02040]	fliK flagellar hook-length control protein FliK	5	6	12	1	7	12
Metabolism	Carbohydrate metabolism	00040 Pentose and glucuronate interconversions [PATH:ko00040]	lyxK L-xylulokinase [EC:2.7.1.53]	2	3	12	4	21	1
Metabolism	Nucleotide metabolism	00230 Purine metabolism [PATH:ko00230]	ygeU xdhC xanthine dehydrogenase iron-sulfur-binding subunit	7	7	4	14	4	7
Metabolism	Metabolism of cofactors and vitamins	00860 Porphyrin and chlorophyll metabolism [PATH:ko00860]	E2.7.8.26 cobS cobV adenosylcobinamide-GDP ribazoletransferase [EC:2.7.8.26]	6	2	8	11	7	9

Environmental Information Processing	Membrane transport	02060 Phosphotransferase system (PTS) [PATH:ko02060]	PTS-Scr-EIIC scrA PTS system sucrose-specific IIC component	2	10	12	2	11	8
Metabolism	Energy metabolism	00920 Sulfur metabolism [PATH:ko00920]	cysN sulfate adenylyltransferase subunit 1 [EC:2.7.7.4]	9	8	8	9	7	3
Metabolism	Carbohydrate metabolism	00640 Propanoate metabolism [PATH:ko00640]	E1.3.1.- [EC:1.3.1.-]	9	6	12	9	8	1
Environmental Information Processing	Signal transduction	02020 Two-component system [PATH:ko02020]	cssR two-component system OmpR family response regulator CssR	9	5	8	7	3	9
Metabolism	Lipid metabolism	00564 Glycerophospholipid metabolism [PATH:ko00564]	eutA ethanolamine utilization protein EutA	5	11	4	7	2	11
Cellular Processes	Cell motility	02040 Flagellar assembly [PATH:ko02040]	fliR flagellar biosynthetic protein FliR	9	3	12	12	3	7
Metabolism	Metabolism of cofactors and vitamins	00860 Porphyrin and chlorophyll metabolism [PATH:ko00860]	chlD bchD magnesium chelatase subunit D [EC:6.6.1.1]	5	11	8	7	4	9
Metabolism	Carbohydrate metabolism	00630 Glyoxylate and dicarboxylate metabolism [PATH:ko00630]	ttuC dmlA tartrate dehydrogenase/decarboxylase / D-malate dehydrogenase [EC:1.1.1.93 4.1.1.73 1.1.1.83]	4	8	8	12	6	6
Metabolism	Carbohydrate metabolism	00053 Ascorbate and aldarate metabolism [PATH:ko00053]	ulaF sgaE L-ribulose-5-phosphate 4-epimerase [EC:5.1.3.4]	6	7	4	9	7	6
Cellular Processes	Cell growth and death	04113 Meiosis - yeast [PATH:ko04113]	HXT MFS transporter SP family sugar:H+ symporter	4	12	4	20	2	3
Genetic Information Processing	Folding sorting and degradation	04122 Sulfur relay system [PATH:ko04122]	MOCS3 UBA4 moeB adenylyltransferase and sulfurtransferase	5	15	8	3	5	5
Metabolism	Metabolism of cofactors and vitamins	00780 Biotin metabolism [PATH:ko00780]	bioD dethiobiotin synthetase [EC:6.3.3.3]	2	8	20	2	12	4
Genetic Information Processing	Translation	00970 Aminoacyl-tRNA biosynthesis [PATH:ko00970]	poxA lysyl-tRNA synthetase class II [EC:6.1.1.6]	7	8	4	7	7	5
Metabolism	Carbohydrate metabolism	00500 Starch and sucrose metabolism [PATH:ko00500]	treC trehalose-6-phosphate hydrolase [EC:3.2.1.93]	8	9	8	4	5	5
Environmental Information Processing	Membrane transport	02010 ABC transporters [PATH:ko02010]	modC molybdate transport system ATP-binding protein [EC:3.6.3.29]	6	10	12	1	7	5

Genetic Information Processing	Replication and repair	03440 Homologous recombination [PATH:ko03440]	recO DNA repair protein RecO (recombination protein O)	8	5	12	8	3	7
Environmental Information Processing	Membrane transport	03070 Bacterial secretion system [PATH:ko03070]	virB1 type IV secretion system protein VirB1	7	6	8	4	8	4
Metabolism	Carbohydrate metabolism	00520 Amino sugar and nucleotide sugar metabolism [PATH:ko00520]	RENBP N-acylglucosamine 2-epimerase [EC:5.1.3.8]	7	6	12	8	5	5
Environmental Information Processing	Membrane transport	03070 Bacterial secretion system [PATH:ko03070]	gspJ general secretion pathway protein J	3	26	4	1	1	4
Metabolism	Metabolism of terpenoids and polyketides	00900 Terpenoid backbone biosynthesis [PATH:ko00900]	idi IDI isopentenyl-diphosphate delta-isomerase [EC:5.3.3.2]	4	2	4	4	9	9
Environmental Information Processing	Membrane transport	02010 ABC transporters [PATH:ko02010]	opuA osmoprotectant transport system ATP-binding protein	4	11	16	2	4	9
Metabolism	Nucleotide metabolism	00230 Purine metabolism [PATH:ko00230]	purO IMP cyclohydrolase [EC:3.5.4.10]	8	3	8	14	5	2
Metabolism	Lipid metabolism	00140 Steroid hormone biosynthesis [PATH:ko00140]	SRD5A1 3-oxo-5-alpha-steroid 4-dehydrogenase 1 [EC:1.3.99.5]	8	5	8	10	5	2
Metabolism	Metabolism of cofactors and vitamins	00860 Porphyrin and chlorophyll metabolism [PATH:ko00860]	chlI bchI magnesium chelatase subunit I [EC:6.6.1.1]	3	8	8	1	7	9
Metabolism	Glycan biosynthesis and metabolism	00540 Lipopolysaccharide biosynthesis [PATH:ko00540]	waaQ rfaQ heptosyltransferase III [EC:2.4.-.-]	6	5	8	4	4	10
Environmental Information Processing	Signal transduction	02020 Two-component system [PATH:ko02020]	tctB putative tricarboxylic transport membrane protein	11	1	12	3	8	1
Environmental Information Processing	Membrane transport	02010 ABC transporters [PATH:ko02010]	natA sodium transport system ATP-binding protein	5	12	4	10	2	3
Environmental Information Processing	Membrane transport	03070 Bacterial secretion system [PATH:ko03070]	virB11 lvhB11 type IV secretion system protein VirB11	9	6	4	3	7	2
Metabolism	Amino acid metabolism	00360 Phenylalanine metabolism [PATH:ko00360]	mhpD 2-keto-4-pentenoate hydratase [EC:4.2.1.80]	9	6	4	3	2	6
Environmental Information Processing	Membrane transport	02010 ABC transporters [PATH:ko02010]	ugpC sn-glycerol 3-phosphate transport system ATP-binding protein [EC:3.6.3.20]	2	7	8	7	3	10

Metabolism	Carbohydrate metabolism	00620 Pyruvate metabolism [PATH:ko00620]	E1.1.2.4 dld D-lactate dehydrogenase (cytochrome) [EC:1.1.2.4]	4	8	4	4	4	8
Genetic Information Processing	Transcription	03020 RNA polymerase [PATH:ko03020]	SIG3.3.1 rpoH RNA polymerase sigma-32 factor	5	7	16	7	3	4
Environmental Information Processing	Membrane transport	02010 ABC transporters [PATH:ko02010]	ABC.CYST.A cystine transport system ATP-binding protein [EC:3.6.3.-]	8	1	8	4	4	6
Environmental Information Processing	Membrane transport	02010 ABC transporters [PATH:ko02010]	araG L-arabinose transport system ATP-binding protein [EC:3.6.3.17]	3	4	12	11	1	8
Environmental Information Processing	Signal transduction	02020 Two-component system [PATH:ko02020]	mdtB RND superfamily multidrug transport protein MdtB	1	9	8	9	4	5
Environmental Information Processing	Membrane transport	02010 ABC transporters [PATH:ko02010]	rbsD D-ribose pyranase [EC:5.-.-.-]	4	5	4	4	11	1
Metabolism	Glycan biosynthesis and metabolism	00550 Peptidoglycan biosynthesis [PATH:ko00550]	E2.4.1.129 peptidoglycan glycosyltransferase [EC:2.4.1.129]	7	4	4	5	4	4
Metabolism	Xenobiotics biodegradation and metabolism	00791 Atrazine degradation [PATH:ko00791]	E3.5.4.- [EC:3.5.4.-]	4	5	4	2	5	8
Environmental Information Processing	Membrane transport	02010 ABC transporters [PATH:ko02010]	ccmC heme exporter protein C	3	4	12	7	7	3
Metabolism	Metabolism of cofactors and vitamins	00730 Thiamine metabolism [PATH:ko00730]	thiO glycine oxidase [EC:1.4.3.19]	4	10	4	2	9	1
Human Diseases	Infectious diseases	05150 Staphylococcus aureus infection [PATH:ko05150]	eta exfoliative toxin A/B	6	3	16	5	6	1
Metabolism	Metabolism of cofactors and vitamins	00860 Porphyrin and chlorophyll metabolism [PATH:ko00860]	hemX uroporphyrin-III C-methyltransferase [EC:2.1.1.107]	2	1	4	1	16	1
Metabolism	Carbohydrate metabolism	00620 Pyruvate metabolism [PATH:ko00620]	ppc phosphoenolpyruvate carboxylase [EC:4.1.1.31]	4	7	20	5	4	2
Metabolism	Carbohydrate metabolism	00630 Glyoxylate and dicarboxylate metabolism [PATH:ko00630]	ttdB L(+)-tartrate dehydratase beta subunit [EC:4.2.1.32]	3	9	4	5	5	3
Metabolism	Energy metabolism	00910 Nitrogen metabolism [PATH:ko00910]	E1.7.99.4C nitrate reductase catalytic subunit [EC:1.7.99.4]	2	10	4	3	2	7

Metabolism	Carbohydrate metabolism	00630 Glyoxylate and dicarboxylate metabolism [PATH:ko00630]	FDH formate dehydrogenase [EC:1.2.1.2]	4	7	4	2	2	8
Metabolism	Metabolism of terpenoids and polyketides	01053 Biosynthesis of siderophore group nonribosomal peptides [PATH:ko01053]	pchF pyochelin synthetase	4	3	4	2	7	6
Environmental Information Processing	Signal transduction	02020 Two-component system [PATH:ko02020]	yesN two-component system response regulator YesN	2	3	8	9	4	6
Environmental Information Processing	Membrane transport	02010 ABC transporters [PATH:ko02010]	hisP histidine transport system ATP-binding protein [EC:3.6.3.21]	4	1	8	3	10	1
Metabolism	Energy metabolism	00910 Nitrogen metabolism [PATH:ko00910]	nirB nitrite reductase (NAD(P)H) large subunit [EC:1.7.1.4]	4	6	8	7	5	2
Genetic Information Processing	Replication and repair	03030 DNA replication [PATH:ko03030]	rnhC ribonuclease HIII [EC:3.1.26.4]	7	3	12	1	3	4
Environmental Information Processing	Membrane transport	02010 ABC transporters [PATH:ko02010]	pstC phosphate transport system permease protein	5	5	12	3	3	3
Metabolism	Carbohydrate metabolism	00650 Butanoate metabolism [PATH:ko00650]	E1.1.1.61 4-hydroxybutyrate dehydrogenase [EC:1.1.1.61]	5	2	8	2	4	5
Metabolism	Nucleotide metabolism	00230 Purine metabolism [PATH:ko00230]	E3.5.2.5 allB allantoinase [EC:3.5.2.5]	4	4	12	3	3	5
Metabolism	Amino acid metabolism	00280 Valine leucine and isoleucine degradation [PATH:ko00280]	E6.4.1.4A 3-methylcrotonyl-CoA carboxylase alpha subunit [EC:6.4.1.4]	4	5	4	2	7	3
Environmental Information Processing	Membrane transport	02010 ABC transporters [PATH:ko02010]	gluB glutamate transport system substrate-binding protein	7	10	4	3	1	1
Environmental Information Processing	Membrane transport	02010 ABC transporters [PATH:ko02010]	tagH teichoic acid transport system ATP-binding protein [EC:3.6.3.40]	5	5	23	4	1	3
Genetic Information Processing	Replication and repair	03430 Mismatch repair [PATH:ko03430]	E3.1.11.1 sbcB exodeoxyribonuclease I [EC:3.1.11.1]	2	8	8	2	4	3
Metabolism	Carbohydrate metabolism	00520 Amino sugar and nucleotide sugar metabolism [PATH:ko00520]	ascD ddhD rfbI CDP-4-dehydro-6-deoxyglucose reductase [EC:1.17.1.1]	4	4	16	2	4	3

Metabolism	Energy metabolism	00920 Sulfur metabolism [PATH:ko00920]	cysJ sulfite reductase (NADPH) flavoprotein alpha-component [EC:1.8.1.2]	4	7	4	3	5	2
Environmental Information Processing	Membrane transport	02010 ABC transporters [PATH:ko02010]	pstA phosphate transport system permease protein	4	2	8	7	2	5
Metabolism	Amino acid metabolism	00260 Glycine serine and threonine metabolism [PATH:ko00260]	soxA sarcosine oxidase subunit alpha [EC:1.5.3.1]	4	3	8	1	3	6
Environmental Information Processing	Membrane transport	02010 ABC transporters [PATH:ko02010]	tagG teichoic acid transport system permease protein	4	3	8	8	1	4
Metabolism	Amino acid metabolism	00330 Arginine and proline metabolism [PATH:ko00330]	E2.3.1.57 speG diamine N-acetyltransferase [EC:2.3.1.57]	2	4	4	2	4	7
Cellular Processes	Transport and catabolism	04142 Lysosome [PATH:ko04142]	NEU1 sialidase-1 [EC:3.2.1.18]	5	6	4	2	1	5
Environmental Information Processing	Membrane transport	02010 ABC transporters [PATH:ko02010]	potH putrescine transport system permease protein	4	6	8	2	3	3
Metabolism	Carbohydrate metabolism	00040 Pentose and glucuronate interconversions [PATH:ko00040]	sgbE L-ribulose-5-phosphate 4-epimerase [EC:5.1.3.4]	4	4	8	1	2	6
Metabolism	Carbohydrate metabolism	00640 Propanoate metabolism [PATH:ko00640]	E3.5.99.7 1-aminocyclopropane-1-carboxylate deaminase [EC:3.5.99.7]	3	6	12	2	5	1
Cellular Processes	Transport and catabolism	04146 Peroxisome [PATH:ko04146]	E4.1.3.4 HMGCL hmgL hydroxymethylglutaryl-CoA lyase [EC:4.1.3.4]	6	6	12	1	2	1
Metabolism	Metabolism of cofactors and vitamins	00860 Porphyrin and chlorophyll metabolism [PATH:ko00860]	cobI-cbiL precorrin-2/cobalt-factor-2 C20-methyltransferase [EC:2.1.1.130 2.1.1.151]	1	5	4	2	3	7
Environmental Information Processing	Membrane transport	02010 ABC transporters [PATH:ko02010]	ABC.GLN1.P putative glutamine transport system permease protein	3	3	4	8	4	2
Environmental Information Processing	Signal transduction	02020 Two-component system [PATH:ko02020]	envZ two-component system OmpR family osmolarity sensor histidine kinase EnvZ [EC:2.7.13.3]	4	7	8	4	1	2
Environmental Information Processing	Signal transduction	02020 Two-component system [PATH:ko02020]	algZ two-component system LytT family sensor histidine kinase AlgZ [EC:2.7.13.3]	4	3	4	1	5	3

Environmental Information Processing	Membrane transport	03070 Bacterial secretion system [PATH:ko03070]	hlyD cyaD hemolysin D	1	8	4	3	4	3
Metabolism	Carbohydrate metabolism	00500 Starch and sucrose metabolism [PATH:ko00500]	E2.4.1.4 amylosucrase [EC:2.4.1.4]	6	2	4	5	1	3
Metabolism	Amino acid metabolism	00280 Valine leucine and isoleucine degradation [PATH:ko00280]	fadJ 3-hydroxyacyl-CoA dehydrogenase / enoyl-CoA hydratase / 3-hydroxybutyryl-CoA epimerase [EC:1.1.1.35 4.2.1.17 5.1.2.3]	1	2	4	3	4	6
Environmental Information Processing	Signal transduction	02020 Two-component system [PATH:ko02020]	vicK two-component system OmpR family sensor histidine kinase VicK [EC:2.7.13.3]	6	3	4	2	2	3
Metabolism	Amino acid metabolism	00280 Valine leucine and isoleucine degradation [PATH:ko00280]	IVD ivd isovaleryl-CoA dehydrogenase [EC:1.3.8.4]	2	6	8	2	2	3
Metabolism	Energy metabolism	00680 Methane metabolism [PATH:ko00680]	echA ech hydrogenase subunit A	1	3	4	2	4	4
Metabolism	Carbohydrate metabolism	00620 Pyruvate metabolism [PATH:ko00620]	eutE aldehyde dehydrogenase	1	1	4	3	2	7
Environmental Information Processing	Membrane transport	03070 Bacterial secretion system [PATH:ko03070]	impK ompA vasF dotU type VI secretion system protein ImpK	1	5	4	3	3	4
Metabolism	Nucleotide metabolism	00230 Purine metabolism [PATH:ko00230]	E2.7.6.5X putative GTP pyrophosphokinase [EC:2.7.6.5]	4	5	4	1	1	4
Environmental Information Processing	Membrane transport	02010 ABC transporters [PATH:ko02010]	opuBD osmoprotectant transport system permease protein	2	7	4	1	3	2
Environmental Information Processing	Membrane transport	02060 Phosphotransferase system (PTS) [PATH:ko02060]	PTS-HPR.FRUB fruB fpr phosphocarrier protein FPr	3	3	12	2	1	3
Environmental Information Processing	Signal transduction	02020 Two-component system [PATH:ko02020]	mdtA putative multidrug efflux transporter MdtA	1	6	8	3	2	3
Metabolism	Energy metabolism	00910 Nitrogen metabolism [PATH:ko00910]	nifD nitrogenase molybdenum-iron protein alpha chain [EC:1.18.6.1]	1	3	8	2	4	3
Environmental Information Processing	Membrane transport	02010 ABC transporters [PATH:ko02010]	potI putrescine transport system permease protein	2	6	8	1	2	2

Environmental Information Processing	Signal transduction	02020 Two-component system [PATH:ko02020]	rcsC two-component system NarL family capsular synthesis sensor histidine kinase RcsC [EC:2.7.13.3]	4	3	4	1	1	4
Environmental Information Processing	Signal transduction	02020 Two-component system [PATH:ko02020]	ntrY two-component system NtrC family nitrogen regulation sensor histidine kinase NtrY [EC:2.7.13.3]	2	3	4	4	4	1
Environmental Information Processing	Signal transduction	02020 Two-component system [PATH:ko02020]	yesM two-component system sensor histidine kinase YesM [EC:2.7.13.3]	2	3	4	4	1	3
Metabolism	Lipid metabolism	00140 Steroid hormone biosynthesis [PATH:ko00140]	E1.1.1.53 3alpha(or 20beta)-hydroxysteroid dehydrogenase [EC:1.1.1.53]	2	3	4	1	5	1
Metabolism	Xenobiotics biodegradation and metabolism	00361 Chlorocyclohexane and chlorobenzene degradation [PATH:ko00361]	E3.8.1.2 2-haloacid dehalogenase [EC:3.8.1.2]	2	1	12	2	3	3
Metabolism	Energy metabolism	00190 Oxidative phosphorylation [PATH:ko00190]	ndhD NAD(P)H-quinone oxidoreductase subunit 4 [EC:1.6.5.3]	1	1	4	1	1	8
Metabolism	Carbohydrate metabolism	00010 Glycolysis / Gluconeogenesis [PATH:ko00010]	tal-pgi transaldolase / glucose-6-phosphate isomerase [EC:2.2.1.2 5.3.1.9]	1	6	4	5	1	2
Environmental Information Processing	Membrane transport	02010 ABC transporters [PATH:ko02010]	togN oligogalacturonide transport system permease protein	1	1	4	7	1	4
Metabolism	Biosynthesis of Other Secondary Metabolites	00941 Flavonoid biosynthesis [PATH:ko00941]	6DCS 6'-deoxychalcone synthase [EC:2.3.1.170]	2	5	8	1	1	2
Environmental Information Processing	Membrane transport	02060 Phosphotransferase system (PTS) [PATH:ko02060]	PTS-Tre-EIIC treB PTS system trehalose-specific IIC component	4	4	4	1	1	2
Metabolism	Amino acid metabolism	00270 Cysteine and methionine metabolism [PATH:ko00270]	mtnK 5-methylthioribose kinase [EC:2.7.1.100]	2	4	8	2	1	2
Metabolism	Lipid metabolism	00561 Glycerolipid metabolism [PATH:ko00561]	ugtP 12-diacylglycerol 3-glucosyltransferase [EC:2.4.1.157]	2	2	4	3	2	2
Metabolism	Energy metabolism	00680 Methane metabolism [PATH:ko00680]	E4.4.1.19 comA phosphosulfolactate synthase [EC:4.4.1.19]	1	1	4	5	1	3

Cellular Processes	Transport and catabolism	04144 Endocytosis [PATH:ko04144]	PLD phospholipase D [EC:3.1.4.4]	1	2	4	4	2	3
Environmental Information Processing	Signal transduction	02020 Two-component system [PATH:ko02020]	basR two-component system OmpR family response regulator BasR	1	6	8	2	1	1
Environmental Information Processing	Membrane transport	03070 Bacterial secretion system [PATH:ko03070]	gspL general secretion pathway protein L	2	1	8	1	2	3
Metabolism	Nucleotide metabolism	00230 Purine metabolism [PATH:ko00230]	rihB ribosylpyrimidine nucleosidase [EC:3.2.2.8]	1	4	4	1	1	3
Metabolism	Carbohydrate metabolism	00010 Glycolysis / Gluconeogenesis [PATH:ko00010]	E1.2.1.9 gapN glyceraldehyde-3-phosphate dehydrogenase (NADP) [EC:1.2.1.9]	3	2	8	1	2	1
Metabolism	Metabolism of terpenoids and polyketides	01053 Biosynthesis of siderophore group nonribosomal peptides [PATH:ko01053]	entF enterobactin synthetase component F [EC:2.7.7.-]	4	1	4	3	1	1
Environmental Information Processing	Signal transduction	02020 Two-component system [PATH:ko02020]	cpxR two-component system OmpR family response regulator CpxR	1	3	4	1	2	2
Metabolism	Carbohydrate metabolism	00630 Glyoxylate and dicarboxylate metabolism [PATH:ko00630]	gyaR glyoxylate reductase [EC:1.1.1.26]	1	2	4	4	2	1
Metabolism	Metabolism of cofactors and vitamins	00860 Porphyrin and chlorophyll metabolism [PATH:ko00860]	cbiGH-cobJ cobalt-precorrin 5A hydrolase / precorrin-3B C17-methyltransferase [EC:3.7.1.12 2.1.1.131]	1	1	8	1	3	1
Environmental Information Processing	Membrane transport	02010 ABC transporters [PATH:ko02010]	occP nocP octopine/nopaline transport system ATP-binding protein [EC:3.6.3.-]	1	1	4	1	2	1
Environmental Information Processing	Membrane transport	02010 ABC transporters [PATH:ko02010]	togM oligogalacturonide transport system permease protein	2	2	4	1	1	1
Metabolism	Energy metabolism	00680 Methane metabolism [PATH:ko00680]	mtaB methanol---5-hydroxybenzimidazolylcobamide Co-methyltransferase [EC:2.1.1.90]	20	22	0	13	11	121

Metabolism	Carbohydrate metabolism	00052 Galactose metabolism [PATH:ko00052]	galT GALT UDPglucose--hexose-1-phosphate uridylyltransferase [EC:2.7.7.12]	42	21	0	19	56	39
Metabolism	Amino acid metabolism	00280 Valine leucine and isoleucine degradation [PATH:ko00280]	E6.4.1.4B 3-methylcrotonyl-CoA carboxylase beta subunit [EC:6.4.1.4]	7	9	0	9	63	56
Environmental Information Processing	Membrane transport	02010 ABC transporters [PATH:ko02010]	malG maltose/maltodextrin transport system permease protein	17	14	0	19	33	48
Metabolism	Carbohydrate metabolism	00040 Pentose and glucuronate interconversions [PATH:ko00040]	E4.2.2.2 pel pectate lyase [EC:4.2.2.2]	18	33	0	14	19	24
Metabolism	Energy metabolism	00680 Methane metabolism [PATH:ko00680]	mvhA vhuA vhcA F420-non-reducing hydrogenase subunit A [EC:1.12.99.-]	14	16	0	19	8	48
Metabolism	Carbohydrate metabolism	00020 Citrate cycle (TCA cycle) [PATH:ko00020]	ACLY ATP citrate (pro-S)-lyase [EC:2.3.3.8]	16	23	0	15	9	41
Human Diseases	Infectious diseases	05111 Vibrio cholerae pathogenic cycle [PATH:ko05111]	mshA MSHA pilin protein MshA	8	3	0	23	40	23
Metabolism	Lipid metabolism	00561 Glycerolipid metabolism [PATH:ko00561]	E1.1.1.6 gldA glycerol dehydrogenase [EC:1.1.1.6]	15	23	0	23	11	20
Metabolism	Amino acid metabolism	00270 Cysteine and methionine metabolism [PATH:ko00270]	E4.1.1.50 speD S-adenosylmethionine decarboxylase [EC:4.1.1.50]	13	32	0	5	23	13
Metabolism	Amino acid metabolism	00280 Valine leucine and isoleucine degradation [PATH:ko00280]	E5.4.99.2A mcmA1 methylmalonyl-CoA mutase N-terminal domain [EC:5.4.99.2]	10	16	0	13	12	32
Environmental Information Processing	Membrane transport	02060 Phosphotransferase system (PTS) [PATH:ko02060]	PTS-Man-EIIA manX PTS system mannose-specific IIA component [EC:2.7.1.69]	12	13	0	11	24	18
Metabolism	Energy metabolism	00680 Methane metabolism [PATH:ko00680]	E3.1.3.71 comB 2-phosphosulfolactate phosphatase [EC:3.1.3.71]	14	51	0	7	4	13
Metabolism	Amino acid metabolism	00330 Arginine and proline metabolism [PATH:ko00330]	E3.5.4.1 codA cytosine deaminase [EC:3.5.4.1]	7	8	0	7	4	46

Human Diseases	Amino acid metabolism	05142 Chagas disease (American trypanosomiasis) [PATH:ko05142]	ptrB oligopeptidase B [EC:3.4.21.83]	1	3	0	3	17	42
Metabolism	Lipid metabolism	00561 Glycerolipid metabolism [PATH:ko00561]	pduD propanediol dehydratase medium subunit [EC:4.2.1.28]	10	17	0	18	12	17
Metabolism	Carbohydrate metabolism	00051 Fructose and mannose metabolism [PATH:ko00051]	E1.1.1.14 gutB L-iditol 2-dehydrogenase [EC:1.1.1.14]	11	20	0	3	21	7
Metabolism	Amino acid metabolism	00330 Arginine and proline metabolism [PATH:ko00330]	prdB D-proline reductase (dithiol) PrdB [EC:1.21.4.1]	3	9	0	9	17	22
Metabolism	Nucleotide metabolism	00230 Purine metabolism [PATH:ko00230]	xdhB xanthine dehydrogenase large subunit [EC:1.17.1.4]	10	28	0	5	4	15
Metabolism	Energy metabolism	00680 Methane metabolism [PATH:ko00680]	mvhG vhuG vhcG F420-non-reducing hydrogenase subunit G [EC:1.12.99.-]	6	8	0	9	5	29
Human Diseases	Infectious diseases	05146 Amoebiasis [PATH:ko05146]	SERPINB serpin B	3	20	0	4	18	11
Metabolism	Carbohydrate metabolism	00053 Ascorbate and aldarate metabolism [PATH:ko00053]	ulaD sgaH 3-dehydro-L-gulonate-6-phosphate decarboxylase [EC:4.1.1.85]	7	18	0	11	20	1
Genetic Information Processing	Replication and repair	03410 Base excision repair [PATH:ko03410]	E6.5.1.1 lig DNA ligase (ATP) [EC:6.5.1.1]	12	10	0	10	13	6
Metabolism	Amino acid metabolism	00280 Valine leucine and isoleucine degradation [PATH:ko00280]	OXCT 3-oxoacid CoA-transferase [EC:2.8.3.5]	4	6	0	33	1	16
Metabolism	Carbohydrate metabolism	00010 Glycolysis / Gluconeogenesis [PATH:ko00010]	gap2 glyceraldehyde-3-phosphate dehydrogenase (NAD(P)) [EC:1.2.1.59]	9	16	0	8	8	10
Metabolism	Energy metabolism	00680 Methane metabolism [PATH:ko00680]	mttC trimethylamine corrinoid protein	3	2	0	13	11	20
Environmental Information Processing	Membrane transport	02060 Phosphotransferase system (PTS) [PATH:ko02060]	PTS-Gut-EIIC srlA PTS system glucitol/sorbitol-specific IIC component	7	6	0	3	26	2
Metabolism	Energy metabolism	00920 Sulfur metabolism [PATH:ko00920]	cysC adenylylsulfate kinase [EC:2.7.1.25]	7	6	0	19	13	6
Metabolism	Metabolism of cofactors and vitamins	00130 Ubiquinone and other terpenoid-quinone biosynthesis [PATH:ko00130]	menD 2-succinyl-5-enolpyruvyl-6-hydroxy-3-cyclohexene-1-carboxylate synthase [EC:2.2.1.9]	12	6	0	5	13	8

Metabolism	Carbohydrate metabolism	00020 Citrate cycle (TCA cycle) [PATH:ko00020]	IDH3 isocitrate dehydrogenase (NAD+) [EC:1.1.1.41]	6	8	0	10	8	16
Metabolism	Carbohydrate Metabolism	00640 Propanoate metabolism [PATH:ko00640]	mmdA methylmalonyl-CoA decarboxylase alpha chain [EC:4.1.1.41]	9	9	0	12	10	8
Metabolism	Lipid metabolism	00061 Fatty acid biosynthesis [PATH:ko00061]	fabA 3-hydroxyacyl-[acyl-carrier-protein] dehydratase [EC:4.2.1.59]	14	10	0	8	12	2
Metabolism	Amino acid metabolism	00330 Arginine and proline metabolism [PATH:ko00330]	argE acetylornithine deacetylase [EC:3.5.1.16]	5	1	0	12	8	19
Environmental Information Processing	Signal transduction	02020 Two-component system [PATH:ko02020]	citC [citrate (pro-3S)-lyase] ligase [EC:6.2.1.22]	6	10	0	5	16	8
Metabolism	Lipid metabolism	00561 Glycerolipid metabolism [PATH:ko00561]	dhaT 13-propanediol dehydrogenase [EC:1.1.1.202]	4	2	0	12	6	21
Environmental Information Processing	Membrane transport	02010 ABC transporters [PATH:ko02010]	lptF lipopolysaccharide export system permease protein	12	7	0	8	13	3
Metabolism	Amino acid metabolism	00330 Arginine and proline metabolism [PATH:ko00330]	argF2 N-succinyl-L-ornithine transcarbamylase [EC:2.1.3.11]	4	6	0	10	5	18
Metabolism	Carbohydrate metabolism	00040 Pentose and glucuronate interconversions [PATH:ko00040]	dlgD 3-dehydro-L-gulonate 2-dehydrogenase [EC:1.1.1.130]	2	7	0	4	23	3
Metabolism	Biosynthesis of other secondary metabolites	00311 Penicillin and cephalosporin biosynthesis [PATH:ko00311]	E3.5.1.11 penicillin amidase [EC:3.5.1.11]	4	24	0	1	7	7
Metabolism	Amino acid metabolism	00270 Cysteine and methionine metabolism [PATH:ko00270]	E2.4.2.28 mtaP 5'-methylthioadenosine phosphorylase [EC:2.4.2.28]	4	2	0	7	5	21
Metabolism	Metabolism of cofactors and vitamins	00860 Porphyrin and chlorophyll metabolism [PATH:ko00860]	PPOX hemY oxygen-dependent protoporphyrinogen oxidase [EC:1.3.3.4]	5	5	0	12	13	6
Metabolism	Energy metabolism	00680 Methane metabolism [PATH:ko00680]	echE ech hydrogenase subunit E	7	10	0	3	10	9
Metabolism	Energy metabolism	00920 Sulfur metabolism [PATH:ko00920]	cysH phosphoadenosine phosphosulfate reductase [EC:1.8.4.8]	7	4	0	5	9	12

Environmental Information Processing	Membrane transport	02060 Phosphotransferase system (PTS) [PATH:ko02060]	PTS-Cel-EIIA celC PTS system cellobiose-specific IIA component [EC:2.7.1.69]	3	1	0	5	6	20
Metabolism	Metabolism of cofactors and vitamins	00750 Vitamin B6 metabolism [PATH:ko00750]	pdxT pdx2 glutamine amidotransferase [EC:2.6.-.-]	4	16	0	3	10	8
Genetic Information Processing	Folding sorting and degradation	04122 Sulfur relay system [PATH:ko04122]	tusA sirA tRNA 2-thiouridine synthesizing protein A [EC:2.8.1.-]	17	8	0	7	5	2
Metabolism	Energy metabolism	00680 Methane metabolism [PATH:ko00680]	E1.12.7.2G ferredoxin hydrogenase gamma subunit [EC:1.12.7.2]	13	6	0	8	5	6
Metabolism	Metabolism of cofactors and vitamins	00790 Folate biosynthesis [PATH:ko00790]	folB dihydroneopterin aldolase [EC:4.1.2.25]	4	6	0	7	7	13
Metabolism	Glycan biosynthesis and metabolism	00550 Peptidoglycan biosynthesis [PATH:ko00550]	mrcB penicillin-binding protein 1B [EC:2.4.1.129 3.4.-.-]	3	18	0	4	11	4
Metabolism	Lipid metabolism	00564 Glycerophospholipid metabolism [PATH:ko00564]	pldB lysophospholipase [EC:3.1.1.5]	12	9	0	9	2	7
Metabolism	Metabolism of terpenoids and polyketides	00900 Terpenoid backbone biosynthesis [PATH:ko00900]	MVD mvaD diphosphomevalonate decarboxylase [EC:4.1.1.33]	5	1	0	1	13	11
Metabolism	Amino acid metabolism	00360 Phenylalanine metabolism [PATH:ko00360]	hipO hippurate hydrolase [EC:3.5.1.32]	7	13	0	8	3	8
Metabolism	Energy metabolism	00680 Methane metabolism [PATH:ko00680]	CODH-ACSA carbon monoxide dehydrogenase / acetyl-CoA synthase subunit alpha [EC:1.2.7.4 1.2.99.2 2.3.1.169]	6	10	0	1	8	8
Metabolism	Metabolism of cofactors and vitamins	00860 Porphyrin and chlorophyll metabolism [PATH:ko00860]	cobP cobU adenosylcobinamide kinase / adenosylcobinamide-phosphate guanylyltransferase [EC:2.7.1.156 2.7.7.62]	4	3	0	5	7	13
Environmental Information Processing	Membrane transport	03070 Bacterial secretion system [PATH:ko03070]	yscN ATP synthase in type III secretion protein SctN [EC:3.6.3.14]	3	11	0	2	8	9
Environmental Information Processing	Signal transduction	04066 HIF-1 signaling pathway [PATH:ko04066]	HK hexokinase [EC:2.7.1.1]	11	3	0	9	2	8
Environmental Information Processing	Membrane transport	02010 ABC transporters [PATH:ko02010]	aglE alpha-glucoside transport system substrate-binding protein	8	10	0	7	6	3

Environmental Information Processing	Membrane transport	02010 ABC transporters [PATH:ko02010]	malK maltose/maltodextrin transport system ATP-binding protein [EC:3.6.3.19]	4	6	0	5	9	8
Metabolism	Metabolism of other amino acids	00440 Phosphonate and phosphinate metabolism [PATH:ko00440]	pat phosphinothricin acetyltransferase [EC:2.3.1.183]	3	19	0	2	8	3
Metabolism	Amino acid metabolism	00250 Alanine aspartate and glutamate metabolism [PATH:ko00250]	E1.2.1.16 gabD succinate-semialdehyde dehydrogenase (NADP+) [EC:1.2.1.16]	8	3	0	8	5	7
Metabolism	Carbohydrate metabolism	00010 Glycolysis / Gluconeogenesis [PATH:ko00010]	E6.2.1.13 acetyl-CoA synthetase (ADP-forming) [EC:6.2.1.13]	5	9	0	8	4	8
Metabolism	Amino acid metabolism	00250 Alanine aspartate and glutamate metabolism [PATH:ko00250]	puuE 4-aminobutyrate aminotransferase [EC:2.6.1.19]	7	5	0	5	5	8
Metabolism	Amino acid metabolism	00310 Lysine degradation [PATH:ko00310]	E4.1.1.18 ldcC cadA lysine decarboxylase [EC:4.1.1.18]	8	6	0	7	3	8
Metabolism	Metabolism of cofactors and vitamins	00770 Pantothenate and CoA biosynthesis [PATH:ko00770]	coaW type II pantothenate kinase [EC:2.7.1.33]	4	1	0	11	5	10
Metabolism	Amino acid metabolism	00280 Valine leucine and isoleucine degradation [PATH:ko00280]	liuC methylglutaconyl-CoA hydratase [EC:4.2.1.18]	5	4	0	1	8	10
Metabolism	Energy metabolism	00190 Oxidative phosphorylation [PATH:ko00190]	ATPVF ntpF V-type H+-transporting ATPase subunit F [EC:3.6.3.14]	5	7	0	15	1	6
Metabolism	Amino acid metabolism	00270 Cysteine and methionine metabolism [PATH:ko00270]	cysM cysteine synthase B [EC:2.5.1.47]	10	9	0	5	2	4
Metabolism	Metabolism of cofactors and vitamins	00130 Ubiquinone and other terpenoid-quinone biosynthesis [PATH:ko00130]	menB naphthoate synthase [EC:4.1.3.36]	1	6	0	9	8	7
Metabolism	Energy metabolism	00680 Methane metabolism [PATH:ko00680]	mtaC methanol corrinoid protein	2	1	0	2	1	20
Cellular Processes	Cell growth and death	04112 Cell cycle - Caulobacter [PATH:ko04112]	pleC two-component system cell cycle sensor histidine kinase PleC [EC:2.7.13.3]	6	11	0	2	6	4
Metabolism	Metabolism of other amino acids	00430 Taurine and hypotaurine metabolism [PATH:ko00430]	tpa taurine-pyruvate aminotransferase [EC:2.6.1.77]	14	6	0	8	1	2

Metabolism	Carbohydrate metabolism	00052 Galactose metabolism [PATH:ko00052]	lacC tagatose 6-phosphate kinase [EC:2.7.1.144]	6	10	0	2	4	6
Metabolism	Amino acid metabolism	00300 Lysine biosynthesis [PATH:ko00300]	lat L-lysine 6-transaminase [EC:2.6.1.36]	8	8	0	3	2	8
Metabolism	Energy metabolism	00680 Methane metabolism [PATH:ko00680]	E1.2.99.2C cooS carbon-monoxide dehydrogenase catalytic subunit [EC:1.2.99.2]	11	2	0	8	5	2
Metabolism	Energy metabolism	00680 Methane metabolism [PATH:ko00680]	E1.2.99.5A fwdA fmdA formylmethanofuran dehydrogenase subunit A [EC:1.2.99.5]	7	7	0	1	7	4
Metabolism	Amino acid metabolism	00330 Arginine and proline metabolism [PATH:ko00330]	E1.5.99.8 proline dehydrogenase [EC:1.5.99.8]	1	2	0	2	14	6
Metabolism	Lipid metabolism	00561 Glycerolipid metabolism [PATH:ko00561]	dhaB glycerol dehydratase large subunit [EC:4.2.1.30]	6	6	0	9	3	6
Genetic Information Processing	Folding sorting and degradation	04122 Sulfur relay system [PATH:ko04122]	mogA molybdopterin adenylyltransferase [EC:2.7.7.75]	8	6	0	5	6	2
Metabolism	Amino acid metabolism	00280 Valine leucine and isoleucine degradation [PATH:ko00280]	E2.3.1.16 fadA acetyl-CoA acyltransferase [EC:2.3.1.16]	2	7	0	8	8	3
Metabolism	Amino acid metabolism	00280 Valine leucine and isoleucine degradation [PATH:ko00280]	E5.4.99.2B mcmA2 methylmalonyl-CoA mutase C-terminal domain [EC:5.4.99.2]	4	3	0	4	4	11
Environmental Information Processing	Membrane transport	02060 Phosphotransferase system (PTS) [PATH:ko02060]	PTS-Gut-EIIB srlE PTS system glucitol/sorbitol-specific IIB component [EC:2.7.1.69]	5	5	0	2	11	2
Metabolism	Energy metabolism	00910 Nitrogen metabolism [PATH:ko00910]	nifK nitrogenase molybdenum-iron protein beta chain [EC:1.18.6.1]	4	6	0	5	8	3
Metabolism	Amino acid metabolism	00250 Alanine aspartate and glutamate metabolism [PATH:ko00250]	putA proline dehydrogenase / delta 1-pyrroline-5-carboxylate dehydrogenase [EC:1.5.99.8 1.5.1.12]	2	0	4	4	7	11
Metabolism	Metabolism of cofactors and vitamins	00860 Porphyrin and chlorophyll metabolism [PATH:ko00860]	cobD threonine-phosphate decarboxylase [EC:4.1.1.81]	5	3	0	2	5	8
Metabolism	Metabolism of cofactors and vitamins	00750 Vitamin B6 metabolism [PATH:ko00750]	pdxB erythronate-4-phosphate dehydrogenase [EC:1.1.1.290]	4	4	0	3	5	9

Metabolism	Amino acid metabolism	00340 Histidine metabolism [PATH:ko00340]	HIS7 glutamine amidotransferase / cyclase [EC:2.4.2.- 4.1.3.-]	4	7	0	9	4	4
Metabolism	Carbohydrate metabolism	00620 Pyruvate metabolism [PATH:ko00620]	acyP acylphosphatase [EC:3.6.1.7]	6	4	0	1	5	7
Metabolism	Metabolism of cofactors and vitamins	00860 Porphyrin and chlorophyll metabolism [PATH:ko00860]	bchE anaerobic magnesium-protoporphyrin IX monomethyl ester cyclase [EC:4.-.-.-]	9	2	0	1	3	7
Environmental Information Processing	Membrane transport	02010 ABC transporters [PATH:ko02010]	glnQ glutamine transport system ATP-binding protein [EC:3.6.3.-]	6	10	0	9	1	3
Metabolism	Energy Metabolism	00910 Nitrogen metabolism [PATH:ko00910]	nifV homocitrate synthase NifV	2	12	0	2	7	4
Environmental Information Processing	Membrane transport	02010 ABC transporters [PATH:ko02010]	aapP bztD general L-amino acid transport system ATP-binding protein [EC:3.6.3.-]	6	7	0	4	4	4
Metabolism	Amino acid metabolism	00330 Arginine and proline metabolism [PATH:ko00330]	E4.1.1.19A adi arginine decarboxylase [EC:4.1.1.19]	6	6	0	3	3	6
Environmental Information Processing	Membrane transport	02060 Phosphotransferase system (PTS) [PATH:ko02060]	PTS-Bgl-EIIB bglF PTS system beta-glucosides-specific IIB component [EC:2.7.1.69]	2	3	0	3	7	8
Metabolism	Energy metabolism	00680 Methane metabolism [PATH:ko00680]	echC ech hydrogenase subunit C	4	3	0	7	4	8
Metabolism	Carbohydrate metabolism	00500 Starch and sucrose metabolism [PATH:ko00500]	xynB xylan 14-beta-xylosidase [EC:3.2.1.37]	4	5	0	2	8	4
Metabolism	Carbohydrate metabolism	00500 Starch and sucrose metabolism [PATH:ko00500]	E2.4.1.7 sucrose phosphorylase [EC:2.4.1.7]	4	9	0	2	4	5
Metabolism	Lipid metabolism	00564 Glycerophospholipid metabolism [PATH:ko00564]	glpB glycerol-3-phosphate dehydrogenase subunit B [EC:1.1.5.3]	5	0	8	5	5	5
Environmental Information Processing	Signal transduction	02020 Two-component system [PATH:ko02020]	mdtC RND superfamily multidrug transport protein MdtC	6	8	0	7	2	3
Cellular Processes	Transport and catabolism	04142 Lysosome [PATH:ko04142]	NAGLU alpha-N-acetylglucosaminidase [EC:3.2.1.50]	6	2	0	2	8	2
Metabolism	Carbohydrate metabolism	00053 Ascorbate and aldarate metabolism [PATH:ko00053]	garD galactarate dehydratase [EC:4.2.1.42]	5	3	0	7	2	6

Metabolism	Carbohydrate metabolism	00052 Galactose metabolism [PATH:ko00052]	gatY-kbaY tagatose 16-diphosphate aldolase GatY/KbaY [EC:4.1.2.40]	4	5	0	3	5	4
Environmental Information Processing	Signal transduction	02020 Two-component system [PATH:ko02020]	glnG ntrC two-component system NtrC family nitrogen regulation response regulator GlnG	3	5	0	4	4	6
Metabolism	Lipid metabolism	00561 Glycerolipid metabolism [PATH:ko00561]	pduE propanediol dehydratase small subunit [EC:4.2.1.28]	4	4	0	9	4	3
Metabolism	Amino acid metabolism	00330 Arginine and proline metabolism [PATH:ko00330]	astC succinylornithine aminotransferase [EC:2.6.1.81]	3	1	0	7	7	3
Metabolism	Carbohydrate metabolism	00010 Glycolysis / Gluconeogenesis [PATH:ko00010]	porD pyruvate ferredoxin oxidoreductase delta subunit [EC:1.2.7.1]	1	6	0	9	4	4
Metabolism	Carbohydrate metabolism	00630 Glyoxylate and dicarboxylate metabolism [PATH:ko00630]	ttdA L(+)-tartrate dehydratase alpha subunit [EC:4.2.1.32]	4	5	0	4	3	4
Environmental Information Processing	Membrane transport	02060 Phosphotransferase system (PTS) [PATH:ko02060]	PTS-Dgl-EIIA gamP PTS system D-glucosamine-specific IIA component [EC:2.7.1.69]	5	3	0	4	2	6
Metabolism	Carbohydrate metabolism	00562 Inositol phosphate metabolism [PATH:ko00562]	iolC 5-dehydro-2-deoxygluconokinase [EC:2.7.1.92]	5	1	0	5	4	4
Environmental Information Processing	Membrane transport	02010 ABC transporters [PATH:ko02010]	xylF D-xylose transport system substrate-binding protein	2	9	0	3	2	6
Cellular Processes	Cell growth and death	04112 Cell cycle - Caulobacter [PATH:ko04112]	dgcB diguanylate cyclase	3	6	0	4	5	2
Metabolism	Energy metabolism	00680 Methane metabolism [PATH:ko00680]	echB ech hydrogenase subunit B	4	9	0	2	2	4
Metabolism	Energy metabolism	00910 Nitrogen metabolism [PATH:ko00910]	nrfC protein NrfC	4	2	0	3	5	4
Metabolism	Amino acid metabolism	00270 Cysteine and methionine metabolism [PATH:ko00270]	E2.1.1.10 mmuM homocysteine S-methyltransferase [EC:2.1.1.10]	2	4	0	2	4	6
Metabolism	Carbohydrate metabolism	00630 Glyoxylate and dicarboxylate metabolism [PATH:ko00630]	E5.3.1.22 gip hydroxypyruvate isomerase [EC:5.3.1.22]	1	2	0	3	5	6
Environmental Information Processing	Membrane transport	02060 Phosphotransferase system (PTS) [PATH:ko02060]	PTS-EI.PTSP ptsP phosphotransferase system enzyme I PtsP [EC:2.7.3.9]	2	2	0	8	5	3

Metabolism	Metabolism of cofactors and vitamins	00860 Porphyrin and chlorophyll metabolism [PATH:ko00860]	cbiG cobalt-precorrin 5A hydrolase [EC:3.7.1.12]	3	4	0	3	2	6
Cellular Processes	Cell motility	02030 Bacterial chemotaxis [PATH:ko02030]	dppA dipeptide transport system substrate-binding protein	2	3	0	13	2	2
Metabolism	Xenobiotics biodegradation and metabolism	00627 Aminobenzoate degradation [PATH:ko00627]	E3.1.3.41 4-nitrophenyl phosphatase [EC:3.1.3.41]	2	1	0	9	1	5
Metabolism	Carbohydrate metabolism	00010 Glycolysis / Gluconeogenesis [PATH:ko00010]	E3.2.1.86A celF 6-phospho-beta-glucosidase [EC:3.2.1.86]	4	5	0	2	4	3
Environmental Information Processing	Membrane transport	02060 Phosphotransferase system (PTS) [PATH:ko02060]	PTS-Scr-EIIB scrA PTS system sucrose-specific IIB component [EC:2.7.1.69]	2	5	0	1	6	2
Environmental Information Processing	Signal transduction	02020 Two-component system [PATH:ko02020]	flrA sigma-54 specific transcriptional regulator flagellar regulatory protein A	3	4	0	4	3	3
Environmental Information Processing	Membrane transport	02010 ABC transporters [PATH:ko02010]	gluA glutamate transport system ATP-binding protein [EC:3.6.3.-]	2	2	0	7	2	5
Environmental Information Processing	Membrane transport	02010 ABC transporters [PATH:ko02010]	nodI lipooligosaccharide transport system ATP-binding protein	2	4	0	2	5	3
Environmental Information Processing	Membrane transport	02060 Phosphotransferase system (PTS) [PATH:ko02060]	PTS-Dgl-EIIB gamP PTS system D-glucosamine-specific IIB component [EC:2.7.1.69]	1	0	4	3	7	3
Environmental Information Processing	Signal transduction	02020 Two-component system [PATH:ko02020]	glrR qseF two-component system NtrC family response regulator GlrR	4	3	0	5	2	3
Environmental Information Processing	Signal transduction	02020 Two-component system [PATH:ko02020]	ttrB tetrathionate reductase subunit B	2	4	0	4	4	3
Metabolism	Carbohydrate metabolism	00650 Butanoate metabolism [PATH:ko00650]	E1.3.1.44 trans-2-enoyl-CoA reductase (NAD+) [EC:1.3.1.44]	2	1	0	1	2	8
Metabolism	Amino acid metabolism	00330 Arginine and proline metabolism [PATH:ko00330]	E3.5.2.10 creatinine amidohydrolase [EC:3.5.2.10]	5	7	0	2	1	2
Environmental Information Processing	Membrane transport	02060 Phosphotransferase system (PTS) [PATH:ko02060]	PTS-Bgl-EIIA bglF PTS system beta-glucosides-specific IIA component [EC:2.7.1.69]	4	4	0	1	2	4

Metabolism	Amino acid metabolism	00400 Phenylalanine tyrosine and tryptophan biosynthesis [PATH:ko00400]	aroKB shikimate kinase / 3-dehydroquinate synthase [EC:2.7.1.71 4.2.3.4]	1	3	0	4	2	6
Metabolism	Carbohydrate metabolism	00030 Pentose phosphate pathway [PATH:ko00030]	hxlB 6-phospho-3-hexuloisomerase [EC:5.3.1.27]	2	4	0	2	5	3
Environmental Information Processing	Signal transduction	02020 Two-component system [PATH:ko02020]	kdpD two-component system OmpR family sensor histidine kinase KdpD [EC:2.7.13.3]	3	1	0	1	4	4
Environmental Information Processing	Membrane transport	02010 ABC transporters [PATH:ko02010]	ugpE sn-glycerol 3-phosphate transport system permease protein	1	4	0	4	3	4
Metabolism	Lipid metabolism	00600 Sphingolipid metabolism [PATH:ko00600]	E4.1.2.27 sphinganine-1-phosphate aldolase [EC:4.1.2.27]	2	3	0	7	1	4
Metabolism	Biosynthesis of other secondary metabolites	00960 Tropane piperidine and pyridine alkaloid biosynthesis [PATH:ko00960]	E6.2.1.- [EC:6.2.1.-]	3	3	0	2	4	2
Environmental Information Processing	Membrane transport	02060 Phosphotransferase system (PTS) [PATH:ko02060]	PTS-Nag-EIIB nagE PTS system N-acetylglucosamine-specific IIB component [EC:2.7.1.69]	2	3	0	3	2	4
Environmental Information Processing	Signal transduction	02020 Two-component system [PATH:ko02020]	kdpE two-component system OmpR family KDP operon response regulator KdpE	2	2	0	2	2	6
Metabolism	Carbohydrate metabolism	00053 Ascorbate and aldarate metabolism [PATH:ko00053]	ulaE sgaU L-ribulose-5-phosphate 3-epimerase [EC:5.1.3.22]	0	9	4	2	4	1
Cellular Processes	Transport and catabolism	04142 Lysosome [PATH:ko04142]	E3.2.1.45 GBA srfJ glucosylceramidase [EC:3.2.1.45]	0	1	4	5	2	6
Metabolism	Metabolism of cofactors and vitamins	00860 Porphyrin and chlorophyll metabolism [PATH:ko00860]	bchY chlorophyllide reductase subunit Y	5	2	0	5	1	1
Metabolism	Energy metabolism	00680 Methane metabolism [PATH:ko00680]	E1.2.99.5E fmdE formylmethanofuran dehydrogenase subunit E [EC:1.2.99.5]	2	3	0	3	3	3
Metabolism	Metabolism of cofactors and vitamins	00730 Thiamine metabolism [PATH:ko00730]	E2.7.6.2 THI80 thiamine pyrophosphokinase [EC:2.7.6.2]	0	3	4	3	2	4
Environmental Information Processing	Signal transduction	04151 PI3K-Akt signaling pathway [PATH:ko04151]	GYS glycogen(starch) synthase [EC:2.4.1.11]	4	6	0	2	1	1

Environmental Information Processing	Signal transduction	02020 Two-component system [PATH:ko02020]	dctP C4-dicarboxylate-binding protein DctP	4	5	0	2	1	1
Metabolism	Amino acid metabolism	00280 Valine leucine and isoleucine degradation [PATH:ko00280]	fadB 3-hydroxyacyl-CoA dehydrogenase / enoyl-CoA hydratase / 3-hydroxybutyryl-CoA epimerase / enoyl-CoA isomerase [EC:1.1.1.35 4.2.1.17 5.1.2.3 5.3.3.8]	2	7	0	2	2	1
Environmental Information Processing	Signal transduction	02020 Two-component system [PATH:ko02020]	narJ nitrate reductase 1 delta subunit	5	0	4	1	3	1
Metabolism	Metabolism of other amino acids	00430 Taurine and hypotaurine metabolism [PATH:ko00430]	xsc sulfoacetaldehyde acetyltransferase [EC:2.3.3.15]	2	2	0	5	1	3
Environmental Information Processing	Membrane transport	02060 Phosphotransferase system (PTS) [PATH:ko02060]	PTS-Glc-EIIB ptsG PTS system glucose-specific IIB component [EC:2.7.1.69]	1	0	4	1	1	8
Metabolism	Amino acid metabolism	00300 Lysine biosynthesis [PATH:ko00300]	dapC N-succinyldiaminopimelate aminotransferase [EC:2.6.1.17]	4	0	8	3	2	1
Metabolism	Metabolism of cofactors and vitamins	00130 Ubiquinone and other terpenoid-quinone biosynthesis [PATH:ko00130]	menE O-succinylbenzoic acid--CoA ligase [EC:6.2.1.26]	2	2	0	1	2	5
Metabolism	Energy metabolism	00190 Oxidative phosphorylation [PATH:ko00190]	ndhF NAD(P)H-quinone oxidoreductase subunit 5 [EC:1.6.5.3]	2	3	0	3	2	3
Metabolism	Metabolism of cofactors and vitamins	00790 Folate biosynthesis [PATH:ko00790]	E1.1.1.220 6-pyruvoyltetrahydropterin 2'-reductase [EC:1.1.1.220]	3	4	0	1	2	2
Metabolism	Carbohydrate metabolism	00500 Starch and sucrose metabolism [PATH:ko00500]	E3.2.1.122 glvA maltose-6'-phosphate glucosidase [EC:3.2.1.122]	1	5	0	3	1	4
Human Diseases	Endocrine and metabolic diseases	04940 Type I diabetes mellitus [PATH:ko04940]	E4.1.1.15 gadB gadA GAD glutamate decarboxylase [EC:4.1.1.15]	2	3	0	2	2	3
Metabolism	Metabolism of other amino acids	00480 Glutathione metabolism [PATH:ko00480]	E6.3.1.8 glutathionylspermidine synthase [EC:6.3.1.8]	0	2	4	1	4	3

Metabolism	Amino acid metabolism	00330 Arginine and proline metabolism [PATH:ko00330]	E6.3.4.6 urea carboxylase [EC:6.3.4.6]	2	1	0	1	1	5
Environmental Information Processing	Membrane transport	02060 Phosphotransferase system (PTS) [PATH:ko02060]	PTS-Fru1-EIID levG PTS system fructose-specific IID component	4	0	4	3	2	2
Environmental Information Processing	Membrane transport	02060 Phosphotransferase system (PTS) [PATH:ko02060]	PTS-Gat-EIIA gatA PTS system galactitol-specific IIA component [EC:2.7.1.69]	2	5	0	1	4	1
Environmental Information Processing	Membrane transport	02060 Phosphotransferase system (PTS) [PATH:ko02060]	PTS-Tre-EIIB treB PTS system trehalose-specific IIB component [EC:2.7.1.69]	2	1	0	1	4	3
Environmental Information Processing	Signal transduction	02020 Two-component system [PATH:ko02020]	evgS bvgS two-component system NarL family sensor histidine kinase EvgS [EC:2.7.13.3]	2	2	0	4	2	1
Environmental Information Processing	Membrane transport	02010 ABC transporters [PATH:ko02010]	gltL glutamate/aspartate transport system ATP-binding protein [EC:3.6.3.-]	3	3	0	3	1	2
Environmental Information Processing	Membrane transport	02010 ABC transporters [PATH:ko02010]	msmE multiple sugar transport system substrate-binding protein	1	3	0	1	1	5
Human Diseases	Infectious diseases	05150 Staphylococcus aureus infection [PATH:ko05150]	sdrC D E serine-aspartate repeat-containing protein C/D/E	1	0	4	2	2	5
Metabolism	Carbohydrate metabolism	00051 Fructose and mannose metabolism [PATH:ko00051]	srlD sorbitol-6-phosphate 2-dehydrogenase [EC:1.1.1.140]	1	4	0	1	2	4
Metabolism	Metabolism of cofactors and vitamins	00670 One carbon pool by folate [PATH:ko00670]	E4.3.1.4 formiminotetrahydrofolate cyclodeaminase [EC:4.3.1.4]	2	1	0	1	1	5
Human Diseases	Infectious diseases	05111 Vibrio cholerae pathogenic cycle [PATH:ko05111]	acfD accessory colonization factor AcfD	1	3	0	1	2	4
Metabolism	Amino acid metabolism	00260 Glycine serine and threonine metabolism [PATH:ko00260]	ddc L-24-diaminobutyrate decarboxylase [EC:4.1.1.86]	1	5	0	3	1	2
Metabolism	Lipid metabolism	00561 Glycerolipid metabolism [PATH:ko00561]	dhbC glycerol dehydratase medium subunit [EC:4.2.1.30]	2	5	0	2	1	2
Metabolism	Carbohydrate metabolism	00052 Galactose metabolism [PATH:ko00052]	ebgA evolved beta-galactosidase subunit alpha [EC:3.2.1.23]	4	2	0	1	1	2
Metabolism	Amino acid metabolism	00330 Arginine and proline metabolism [PATH:ko00330]	hyuA N-methylhydantoinase A [EC:3.5.2.14]	1	2	0	2	2	4

Environmental Information Processing	Signal transduction	02020 Two-component system [PATH:ko02020]	kdpB K+-transporting ATPase ATPase B chain [EC:3.6.3.12]	1	7	0	3	1	2
Metabolism	Energy metabolism	00910 Nitrogen metabolism [PATH:ko00910]	norB nitric oxide reductase subunit B [EC:1.7.2.5]	2	4	0	4	2	1
Metabolism	Energy metabolism	00680 Methane metabolism [PATH:ko00680]	E1.12.7.2S ferredoxin hydrogenase small subunit [EC:1.12.7.2]	2	3	0	2	1	3
Metabolism	Carbohydrate metabolism	00650 Butanoate metabolism [PATH:ko00650]	butA (RR)-butanediol dehydrogenase / diacetyl reductase [EC:1.1.1.4 1.1.1.303]	1	0	16	1	2	3
Metabolism	Carbohydrate metabolism	00620 Pyruvate metabolism [PATH:ko00620]	poxB pyruvate dehydrogenase (quinone) [EC:1.2.5.1]	2	1	0	1	4	1
Cellular Processes	Cell motility	02030 Bacterial chemotaxis [PATH:ko02030]	tsr methyl-accepting chemotaxis protein I serine sensor receptor	0	2	8	2	3	1
Environmental Information Processing	Membrane transport	02010 ABC transporters [PATH:ko02010]	msmF multiple sugar transport system permease protein	4	0	4	1	1	1
Metabolism	Energy metabolism	00190 Oxidative phosphorylation [PATH:ko00190]	ATPVC ntpC V-type H+-transporting ATPase subunit C [EC:3.6.3.14]	1	2	0	4	1	1
Metabolism	Biosynthesis of other secondary metabolites	00960 Tropane piperidine and pyridine alkaloid biosynthesis [PATH:ko00960]	E2.8.3.17 fldA cinnamoyl-CoA:phenyllactate CoA-transferase [EC:2.8.3.17]	2	2	0	2	1	2
Metabolism	Carbohydrate metabolism	00500 Starch and sucrose metabolism [PATH:ko00500]	E3.2.1.58 glucan 13-beta-glucosidase [EC:3.2.1.58]	1	2	0	1	1	4
Genetic Information Processing	Folding sorting and degradation	03060 Protein export [PATH:ko03060]	SEC11 sipW signal peptidase endoplasmic reticulum-type [EC:3.4.-.-]	1	2	0	1	1	3
Environmental Information Processing	Signal transduction	02020 Two-component system [PATH:ko02020]	cusS copS silS two-component system OmpR family heavy metal sensor histidine kinase CusS [EC:2.7.13.3]	1	4	0	1	1	3
Metabolism	Metabolism of cofactors and vitamins	00130 Ubiquinone and other terpenoid-quinone biosynthesis [PATH:ko00130]	menF menaquinone-specific isochorismate synthase [EC:5.4.4.2]	3	0	4	1	1	1
Metabolism	Amino acid metabolism	00300 Lysine biosynthesis [PATH:ko00300]	nifV homocitrate synthase NifV [EC:2.3.3.14]	1	1	0	2	2	1

Environmental Information Processing	Signal transduction	02020 Two-component system [PATH:ko02020]	resE two-component system OmpR family sensor histidine kinase ResE [EC:2.7.13.3]	1	6	0	1	1	1
Environmental Information Processing	Membrane transport	02010 ABC transporters [PATH:ko02010]	smoK mtlK sorbitol/mannitol transport system ATP-binding protein	1	1	0	2	1	3
Metabolism	Nucleotide metabolism	00230 Purine metabolism [PATH:ko00230]	yagR xanthine dehydrogenase YagR molybdenum-binding subunit [EC:1.17.1.4]	1	3	0	4	1	1
Environmental Information Processing	Signal transduction	02020 Two-component system [PATH:ko02020]	E3.5.2.6 ampC penP beta-lactamase [EC:3.5.2.6]	1	0	4	2	1	3
Metabolism	Metabolism of cofactors and vitamins	00770 Pantothenate and CoA biosynthesis [PATH:ko00770]	PPCDC coaC phosphopantothenoylcysteine decarboxylase [EC:4.1.1.36]	1	3	0	4	1	1
Genetic Information Processing	Folding sorting and degradation	03018 RNA degradation [PATH:ko03018]	SKI3 TTC37 superkiller protein 3	2	1	0	1	1	3
Environmental Information Processing	Membrane transport	02010 ABC transporters [PATH:ko02010]	fliY cystine transport system substrate-binding protein	1	1	0	1	3	1
Environmental Information Processing	Membrane transport	03070 Bacterial secretion system [PATH:ko03070]	gspH general secretion pathway protein H	1	3	0	2	2	1
Human Diseases	Infectious diseases	05100 Bacterial invasion of epithelial cells [PATH:ko05100]	yeeJ adhesin/invasin	1	1	0	3	1	2
Metabolism	Metabolism of cofactors and vitamins	00860 Porphyrin and chlorophyll metabolism [PATH:ko00860]	E1.14.13.81 acsF chlE magnesium-protoporphyrin IX monomethyl ester (oxidative) cyclase [EC:1.14.13.81]	1	2	0	1	1	1
Metabolism	Carbohydrate metabolism	00640 Propanoate metabolism [PATH:ko00640]	E2.7.2.15 tdcD pduW propionate kinase [EC:2.7.2.15]	1	1	0	1	2	1
Environmental Information Processing	Membrane transport	02060 Phosphotransferase system (PTS) [PATH:ko02060]	PTS-Nag-EIIC nagE PTS system N-acetylglucosamine-specific IIC component	2	0	4	2	1	1
Environmental Information Processing	Membrane transport	03070 Bacterial secretion system [PATH:ko03070]	gspI general secretion pathway protein I	1	1	0	3	1	1

Environmental Information Processing	Membrane transport	02010 ABC transporters [PATH:ko02010]	ugpA sn-glycerol 3-phosphate transport system permease protein	1	3	0	1	1	1
Environmental Information Processing	Signal transduction	02020 Two-component system [PATH:ko02020]	lytS two-component system LytT family sensor histidine kinase LytS [EC:2.7.13.3]	1	1	0	2	1	1
Metabolism	Amino acid metabolism	00300 Lysine biosynthesis [PATH:ko00300]	patA aminotransferase [EC:2.6.1.-]	1	2	0	1	1	1
Environmental Information Processing	Membrane transport	02010 ABC transporters [PATH:ko02010]	proV glycine betaine/proline transport system ATP-binding protein [EC:3.6.3.32]	1	1	0	1	1	1
Environmental Information Processing	Signal transduction	02020 Two-component system [PATH:ko02020]	desR two-component system NarL family response regulator DesR	1	1	0	1	1	1
Metabolism	Carbohydrate metabolism	00500 Starch and sucrose metabolism [PATH:ko00500]	E3.2.1.39 glucan endo-13-beta-D-glucosidase [EC:3.2.1.39]	27	91	4	0	4	1
Metabolism	Nucleotide metabolism	00230 Purine metabolism [PATH:ko00230]	apaH bis(5'-nucleosyl)-tetraphosphatase (symmetrical) [EC:3.6.1.41]	20	17	12	0	27	9
Metabolism	Xenobiotics biodegradation and metabolism	00362 Benzoate degradation [PATH:ko00362]	E3.7.1.9 2-hydroxymuconate-semialdehyde hydrolase [EC:3.7.1.9]	36	23	8	8	0	2
Metabolism	Xenobiotics biodegradation and metabolism	00362 Benzoate degradation [PATH:ko00362]	E4.1.1.77 4-oxalocrotonate decarboxylase [EC:4.1.1.77]	9	16	4	0	2	2
Metabolism	Metabolism of cofactors and vitamins	00770 Pantothenate and CoA biosynthesis [PATH:ko00770]	birA-coaX biotin-[acetyl-CoA-carboxylase] ligase / type III pantothenate kinase [EC:6.3.4.15 2.7.1.33]	2	4	8	0	12	3
Cellular Processes	Cell motility	02030 Bacterial chemotaxis [PATH:ko02030]	cheZ chemotaxis protein CheZ	7	8	16	0	4	3
Metabolism	Metabolism of cofactors and vitamins	00860 Porphyrin and chlorophyll metabolism [PATH:ko00860]	MET8 precorrin-2 dehydrogenase / sirohydrochlorin ferrochelatase [EC:1.3.1.76 4.99.1.4]	3	6	20	0	4	1
Environmental Information Processing	Membrane transport	02010 ABC transporters [PATH:ko02010]	artJ arginine transport system substrate-binding protein	4	10	8	0	2	1
Metabolism	Amino acid metabolism	00330 Arginine and proline metabolism [PATH:ko00330]	E2.7.3.3 arginine kinase [EC:2.7.3.3]	1	3	8	0	8	3

Metabolism	Energy metabolism	00680 Methane metabolism [PATH:ko00680]	ehbQ energy-converting hydrogenase B subunit Q	2	1	4	9	0	7
Environmental Information Processing	Signal transduction	02020 Two-component system [PATH:ko02020]	citG triphosphoribosyl-dephospho-CoA synthase [EC:2.7.8.25]	1	7	8	0	2	5
Genetic Information Processing	Replication and repair	03410 Base excision repair [PATH:ko03410]	OGG1 N-glycosylase/DNA lyase [EC:3.2.2.- 4.2.99.18]	3	5	8	0	2	3
Metabolism	Carbohydrate metabolism	00500 Starch and sucrose metabolism [PATH:ko00500]	bcsA cellulose synthase (UDP-forming) [EC:2.4.1.12]	3	3	4	2	6	0
Metabolism	Carbohydrate metabolism	00520 Amino sugar and nucleotide sugar metabolism [PATH:ko00520]	nanE N-acylglucosamine-6-phosphate 2-epimerase [EC:5.1.3.9]	2	7	8	0	2	4
Metabolism	Carbohydrate metabolism	00650 Butanoate metabolism [PATH:ko00650]	E4.1.1.5 alsD acetolactate decarboxylase [EC:4.1.1.5]	4	7	4	0	2	1
Metabolism	Metabolism of cofactors and vitamins	00860 Porphyrin and chlorophyll metabolism [PATH:ko00860]	eutT ethanolamine utilization cobalamin adenosyltransferase [EC:2.5.1.17]	1	5	8	2	0	6
Metabolism	Metabolism of cofactors and vitamins	00730 Thiamine metabolism [PATH:ko00730]	thiDE hydroxymethylpyrimidine kinase / phosphomethylpyrimidine kinase / thiamine-phosphate diphosphorylase [EC:2.7.1.49 2.7.4.7 2.5.1.3]	4	1	8	0	2	4
Environmental Information Processing	Membrane transport	02060 Phosphotransferase system (PTS) [PATH:ko02060]	PTS-Mal-EIIB malX PTS system maltose and glucose-specific IIB component [EC:2.7.1.69]	4	2	8	0	1	4
Environmental Information Processing	Membrane transport	02010 ABC transporters [PATH:ko02010]	yojI putative ATP-binding cassette transporter	2	8	4	0	1	2
Metabolism	Metabolism of cofactors and vitamins	00860 Porphyrin and chlorophyll metabolism [PATH:ko00860]	cysG uroporphyrin-III C-methyltransferase / precorrin-2 dehydrogenase / sirohydrochlorin ferrochelatase [EC:2.1.1.107 1.3.1.76 4.99.1.4]	4	5	4	0	1	1
Metabolism	Carbohydrate metabolism	00500 Starch and sucrose metabolism [PATH:ko00500]	pgmB beta-phosphoglucomutase [EC:5.4.2.6]	3	4	8	1	3	0

Metabolism	Biosynthesis of other secondary metabolites	00960 Tropane piperidine and pyridine alkaloid biosynthesis [PATH:ko00960]	E3.1.1.- esterase / lipase [EC:3.1.1.-]	1	3	4	0	3	3
Genetic Information Processing	Replication and repair	03410 Base excision repair [PATH:ko03410]	MPG DNA-3-methyladenine glycosylase [EC:3.2.2.21]	2	3	4	0	2	4
Environmental Information Processing	Membrane transport	02010 ABC transporters [PATH:ko02010]	alsA D-allose transport system ATP-binding protein [EC:3.6.3.17]	3	4	4	0	3	1
Environmental Information Processing	Signal transduction	02020 Two-component system [PATH:ko02020]	tctE two-component system OmpR family sensor histidine kinase TctE [EC:2.7.13.3]	3	4	12	0	2	1
Environmental Information Processing	Signal transduction	04066 HIF-1 signaling pathway [PATH:ko04066]	PFKFB 6-phosphofructo-2-kinase / fructose-26-bisphosphatase [EC:2.7.1.105 3.1.3.46]	4	2	4	0	2	1
Metabolism	Amino acid metabolism	00260 Glycine serine and threonine metabolism [PATH:ko00260]	betB gbsA betaine-aldehyde dehydrogenase [EC:1.2.1.8]	2	3	8	2	0	3
Environmental Information Processing	Membrane transport	02010 ABC transporters [PATH:ko02010]	lsrA ego AI-2 transport system ATP-binding protein	1	5	8	0	1	3
Metabolism	Carbohydrate metabolism	00500 Starch and sucrose metabolism [PATH:ko00500]	otsA trehalose 6-phosphate synthase [EC:2.4.1.15]	1	4	4	0	1	4
Genetic Information Processing	Transcription	03020 RNA polymerase [PATH:ko03020]	SIG3.4 RNA polymerase sporulation-specific sigma factor	2	2	4	1	4	0
Environmental Information Processing	Signal transduction	02020 Two-component system [PATH:ko02020]	baeR two-component system OmpR family response regulator BaeR	2	4	4	0	1	3
Metabolism	Metabolism of cofactors and vitamins	00860 Porphyrin and chlorophyll metabolism [PATH:ko00860]	cobA uroporphyrin-III C-methyltransferase [EC:2.1.1.107]	2	5	4	0	2	1
Metabolism	Carbohydrate metabolism	00030 Pentose phosphate pathway [PATH:ko00030]	E2.7.1.12 gntK idnK gluconokinase [EC:2.7.1.12]	2	1	4	0	2	3
Metabolism	Energy metabolism	00190 Oxidative phosphorylation [PATH:ko00190]	coxA cytochrome c oxidase subunit I [EC:1.9.3.1]	3	2	8	1	2	0
Metabolism	Carbohydrate metabolism	00051 Fructose and mannose metabolism [PATH:ko00051]	E2.7.1.105 PFK 6-phosphofructo-2-kinase [EC:2.7.1.105]	1	1	4	2	4	0
Cellular Processes	Cell motility	02040 Flagellar assembly [PATH:ko02040]	fliR-flhB flagellar biosynthetic protein FliR/FlhB	1	2	4	0	3	1

Metabolism	Carbohydrate metabolism	00640 Propanoate metabolism [PATH:ko00640]	pduL phosphotransacylase	1	6	4	1	1	0
Environmental Information Processing	Signal transduction	02020 Two-component system [PATH:ko02020]	wspE two-component system chemotaxis family sensor histidine kinase and response regulator WspE	1	1	4	3	0	3
Environmental Information Processing	Signal transduction	02020 Two-component system [PATH:ko02020]	hydH zraS two-component system NtrC family sensor histidine kinase HydH [EC:2.7.13.3]	1	1	4	1	4	0
Metabolism	Metabolism of cofactors and vitamins	00760 Nicotinate and nicotinamide metabolism [PATH:ko00760]	NAMPT nicotinamide phosphoribosyltransferase [EC:2.4.2.12]	2	1	4	1	2	0
Metabolism	Amino acid metabolism	00280 Valine leucine and isoleucine degradation [PATH:ko00280]	vorA 2-oxoisovalerate ferredoxin oxidoreductase alpha subunit [EC:1.2.7.7]	2	1	4	1	1	0
Human Diseases	Infectious diseases	05150 Staphylococcus aureus infection [PATH:ko05150]	dltA D-alanine--poly(phosphoribitol) ligase subunit 1 [EC:6.1.1.13]	1	1	4	0	1	1
Environmental Information Processing	Signal transduction	02020 Two-component system [PATH:ko02020]	lrgB holin-like protein LrgB	1	1	4	3	0	1
Environmental Information Processing	Membrane transport	02010 ABC transporters [PATH:ko02010]	rhaT rhamnose transport system ATP-binding protein [EC:3.6.3.17]	1	2	4	0	1	1
Metabolism	Amino acid metabolism	00250 Alanine aspartate and glutamate metabolism [PATH:ko00250]	E3.5.1.15 ASPA aspartoacylase [EC:3.5.1.15]	1	1	4	1	1	0
Cellular Processes	Transport and catabolism	04146 Peroxisome [PATH:ko04146]	E2.7.1.36 MVK mvaK1 mevalonate kinase [EC:2.7.1.36]	1	3	0	0	15	17
Metabolism	Amino acid metabolism	00330 Arginine and proline metabolism [PATH:ko00330]	E5.1.1.4 proline racemase [EC:5.1.1.4]	9	8	0	0	5	12
Environmental Information Processing	Membrane transport	02010 ABC transporters [PATH:ko02010]	lacG araQ lactose/L-arabinose transport system permease protein	9	5	0	0	6	8
Metabolism	Metabolism of cofactors and vitamins	00790 Folate biosynthesis [PATH:ko00790]	pabBC para-aminobenzoate synthetase / 4-amino-4-deoxychorismate lyase [EC:2.6.1.85 4.1.3.38]	3	1	0	0	14	5

Metabolism	Carbohydrate metabolism	00052 Galactose metabolism [PATH:ko00052]	dgoD galactonate dehydratase [EC:4.2.1.6]	4	7	0	0	5	5
Environmental Information Processing	Signal transduction	02020 Two-component system [PATH:ko02020]	rpaB two-component system OmpR family response regulator RpaB	7	5	0	0	5	3
Metabolism	Carbohydrate metabolism	00520 Amino sugar and nucleotide sugar metabolism [PATH:ko00520]	arnA pmrI UDP-4-amino-4-deoxy-L-arabinose formyltransferase / UDP-glucuronic acid dehydrogenase (UDP-4-keto-hexauronic acid decarboxylating) [EC:2.1.2.13 1.1.1.305]	4	4	0	0	4	6
Environmental Information Processing	Membrane transport	03070 Bacterial secretion system [PATH:ko03070]	yscU type III secretion protein SctU	2	6	0	0	7	3
Metabolism	Energy metabolism	00680 Methane metabolism [PATH:ko00680]	cdhE acetyl-CoA decarbonylase/synthase complex subunit gamma [EC:2.1.1.245]	5	3	0	0	2	6
Environmental Information Processing	Signal transduction	02020 Two-component system [PATH:ko02020]	arnB pmrH UDP-4-amino-4-deoxy-L-arabinose-oxoglutarate aminotransferase [EC:2.6.1.87]	1	8	0	0	5	3
Metabolism	Lipid metabolism	00140 Steroid hormone biosynthesis [PATH:ko00140]	E3.1.6.1 aslA arylsulfatase [EC:3.1.6.1]	2	3	0	4	0	7
Environmental Information Processing	Signal transduction	02020 Two-component system [PATH:ko02020]	algR two-component system LytT family response regulator AlgR	2	5	0	0	4	4
Environmental Information Processing	Membrane transport	02060 Phosphotransferase system (PTS) [PATH:ko02060]	PTS-Fru-EIIB fruA PTS system fructose-specific IIB component [EC:2.7.1.69]	1	4	0	0	6	4
Human Diseases	Immune diseases	05322 Systemic lupus erythematosus [PATH:ko05322]	TROVE2 SSA2 60 kDa SS-A/Ro ribonucleoprotein	4	5	0	2	5	0
Metabolism	Amino acid metabolism	00380 Tryptophan metabolism [PATH:ko00380]	cypD E CYP102A2 3 cytochrome P450 / NADPH-cytochrome P450 reductase [EC:1.14.14.1 1.6.2.4]	1	4	0	2	0	8
Environmental Information Processing	Membrane transport	02010 ABC transporters [PATH:ko02010]	xylH D-xylose transport system permease protein	4	10	0	2	0	2

Metabolism	Amino acid metabolism	00250 Alanine aspartate and glutamate metabolism [PATH:ko00250]	E4.1.1.12 aspartate 4-decarboxylase [EC:4.1.1.12]	7	2	0	0	2	1
Environmental Information Processing	Membrane transport	03070 Bacterial secretion system [PATH:ko03070]	virB10 lvhB10 type IV secretion system protein VirB10	7	1	0	0	4	1
Environmental Information Processing	Membrane transport	02010 ABC transporters [PATH:ko02010]	yddA putative ATP-binding cassette transporter	2	7	0	1	4	0
Environmental Information Processing	Membrane transport	02010 ABC transporters [PATH:ko02010]	opuC osmoprotectant transport system substrate-binding protein	2	2	0	0	4	4
Environmental Information Processing	Membrane transport	03070 Bacterial secretion system [PATH:ko03070]	yscJ type III secretion protein SctJ	2	3	0	0	1	6
Environmental Information Processing	Membrane transport	02060 Phosphotransferase system (PTS) [PATH:ko02060]	PTS-Glc-EIIA crr PTS system glucose-specific IIA component [EC:2.7.1.69]	2	2	0	0	3	4
Environmental Information Processing	Membrane transport	02010 ABC transporters [PATH:ko02010]	ABC.ARG.S argT lysine/arginine/ornithine transport system substrate-binding protein	0	11	4	0	1	1
Environmental Information Processing	Membrane transport	02010 ABC transporters [PATH:ko02010]	cbiN cobalt transport protein	1	6	0	0	1	3
Metabolism	Energy metabolism	00680 Methane metabolism [PATH:ko00680]	cdhD acetyl-CoA decarbonylase/synthase complex subunit delta [EC:2.1.1.245]	3	5	0	0	1	3
Metabolism	Amino acid metabolism	00400 Phenylalanine tyrosine and tryptophan biosynthesis [PATH:ko00400]	E1.3.1.12 prephenate dehydrogenase [EC:1.3.1.12]	2	1	0	0	5	1
Environmental Information Processing	Signal transduction	02020 Two-component system [PATH:ko02020]	mprA two-component system OmpR family response regulator MprA	1	6	0	0	2	2
Metabolism	Metabolism of cofactors and vitamins	00830 Retinol metabolism [PATH:ko00830]	RETSAT all-trans-retinol 1314-reductase [EC:1.3.99.23]	1	1	0	0	1	5
Environmental Information Processing	Membrane transport	02010 ABC transporters [PATH:ko02010]	ccmB heme exporter protein B	1	6	0	0	2	1
Metabolism	Amino acid metabolism	00260 Glycine serine and threonine metabolism	ghrA gyoxylate/hydroxypyruvate reductase A [EC:1.1.1.79 1.1.1.81]	4	1	0	0	2	1

		[PATH:ko00260]							
Metabolism	Amino acid metabolism	00330 Arginine and proline metabolism [PATH:ko00330]	E3.5.1.4 amiE amidase [EC:3.5.1.4]	3	1	0	2	0	3
Genetic Information Processing	Replication and repair	03410 Base excision repair [PATH:ko03410]	alkA DNA-3-methyladenine glycosylase II [EC:3.2.2.21]	3	3	0	2	1	0
Metabolism	Energy metabolism	00190 Oxidative phosphorylation [PATH:ko00190]	coxB cytochrome c oxidase subunit II [EC:1.9.3.1]	1	2	0	1	4	0
Metabolism	Energy metabolism	00720 Carbon fixation pathways in prokaryotes [PATH:ko00720]	fadN 3-hydroxyacyl-CoA dehydrogenase [EC:1.1.1.35]	1	2	0	7	1	0
Metabolism	Carbohydrate metabolism	00630 Glyoxylate and dicarboxylate metabolism [PATH:ko00630]	mcl1 L-malyl-CoA/beta-methylmalyl-CoA lyase	4	3	0	2	0	1
Human Diseases	Infectious diseases	05143 African trypanosomiasis [PATH:ko05143]	THOP1 thimet oligopeptidase [EC:3.4.24.15]	2	0	8	0	2	2
Metabolism	Amino acid metabolism	00360 Phenylalanine metabolism [PATH:ko00360]	dadA D-amino-acid dehydrogenase [EC:1.4.99.1]	2	0	4	0	1	3
Environmental Information Processing	Signal transduction	02020 Two-component system [PATH:ko02020]	mtrA two-component system OmpR family response regulator MtrA	2	3	0	0	1	2
Environmental Information Processing	Signal transduction	02020 Two-component system [PATH:ko02020]	narX two-component system NarL family nitrate/nitrite sensor histidine kinase NarX [EC:2.7.13.3]	2	2	0	1	2	0
Environmental Information Processing	Signal transduction	02020 Two-component system [PATH:ko02020]	tctD two-component system OmpR family response regulator TctD	5	1	0	0	1	1
Metabolism	Carbohydrate metabolism	00030 Pentose phosphate pathway [PATH:ko00030]	E1.1.1.215 gluconate 2-dehydrogenase [EC:1.1.1.215]	1	3	0	1	0	3
Metabolism	Lipid metabolism	00561 Glycerolipid metabolism [PATH:ko00561]	E2.7.8.20 mdoB phosphoglycerol transferase [EC:2.7.8.20]	2	1	0	1	3	0
Cellular Processes	Transport and catabolism	04146 Peroxisome [PATH:ko04146]	E5.1.99.4 AMACR mcr alpha-methylacyl-CoA racemase [EC:5.1.99.4]	2	1	0	0	1	3
Metabolism	Amino acid metabolism	00310 Lysine degradation [PATH:ko00310]	GCDH gcdH glutaryl-CoA dehydrogenase [EC:1.3.8.6]	2	5	0	0	1	1

Environmental Information Processing	Membrane transport	02060 Phosphotransferase system (PTS) [PATH:ko02060]	PTS-Gat-EIIB gatB PTS system galactitol-specific IIB component [EC:2.7.1.69]	2	1	0	0	2	1
Human Diseases	Infectious diseases	05133 Pertussis [PATH:ko05133]	cyaA anthrax edema toxin adenylate cyclase [EC:4.6.1.1]	1	2	0	3	0	3
Cellular Processes	Cell growth and death	04112 Cell cycle - Caulobacter [PATH:ko04112]	podJ localization factor PodJL	1	5	0	1	0	2
Metabolism	Amino acid metabolism	00330 Arginine and proline metabolism [PATH:ko00330]	prdE D-proline reductase (dithiol) PrdE [EC:1.21.4.1]	0	6	4	0	1	1
Cellular Processes	Cell communication	04510 Focal adhesion [PATH:ko04510]	COL4A collagen type IV alpha	2	1	0	1	0	2
Cellular Processes	Cell motility	02040 Flagellar assembly [PATH:ko02040]	fliJ flagellar FliJ protein	2	0	4	3	1	0
Human Diseases	Cancers	05204 Chemical carcinogenesis [PATH:ko05204]	frmA ADH5 adhC S-(hydroxymethyl)glutathione dehydrogenase / alcohol dehydrogenase [EC:1.1.1.284 1.1.1.1]	2	3	0	0	1	1
Environmental Information Processing	Signal transduction	02020 Two-component system [PATH:ko02020]	kdpA K+-transporting ATPase ATPase A chain [EC:3.6.3.12]	2	1	0	0	1	1
Environmental Information Processing	Membrane transport	02010 ABC transporters [PATH:ko02010]	msmG multiple sugar transport system permease protein	1	3	0	0	1	3
Metabolism	Glycan biosynthesis and metabolism	00550 Peptidoglycan biosynthesis [PATH:ko00550]	murM serine/alanine adding enzyme [EC:2.3.2.10]	1	0	4	0	1	3
Metabolism	Xenobiotics biodegradation and metabolism	00362 Benzoate degradation [PATH:ko00362]	pcaI 3-oxoadipate CoA-transferase alpha subunit [EC:2.8.3.6]	1	3	0	0	1	2
Metabolism	Nucleotide metabolism	00230 Purine metabolism [PATH:ko00230]	yagT xanthine dehydrogenase YagT iron-sulfur-binding subunit	1	3	0	1	2	0
Environmental Information Processing	Signal transduction	02020 Two-component system [PATH:ko02020]	ycbB glnL two-component system response regulator YcbB	2	1	0	0	1	3
Environmental Information Processing	Membrane transport	02060 Phosphotransferase system (PTS) [PATH:ko02060]	PTS-Glc-EIIC ptsG PTS system glucose-specific IIC component	1	2	0	0	2	1
Environmental Information	Signal transduction	02020 Two-component system [PATH:ko02020]	baeS two-component system OmpR family sensor histidine kinase BaeS [EC:2.7.13.3]	2	1	0	0	1	1

Processing									
Metabolism	Energy metabolism	00910 Nitrogen metabolism [PATH:ko00910]	narZ nitrate reductase 2 alpha subunit [EC:1.7.99.4]	1	2	0	0	2	1
Genetic Information Processing	Replication and repair	03410 Base excision repair [PATH:ko03410]	nei endonuclease VIII [EC:3.2.2.- 4.2.99.18]	1	3	0	0	1	1
Metabolism	Metabolism of terpenoids and polyketides	00909 Sesquiterpenoid and triterpenoid biosynthesis [PATH:ko00909]	shc sqhC squalene-hopene/tetraprenyl-beta-curcumene cyclase [EC:5.4.99.17 4.2.1.129]	1	4	0	0	1	1
Environmental Information Processing	Signal transduction	02020 Two-component system [PATH:ko02020]	cssS two-component system OmpR family sensor histidine kinase CssS [EC:2.7.13.3]	1	0	4	3	1	0
Environmental Information Processing	Membrane transport	02010 ABC transporters [PATH:ko02010]	dppB dipeptide transport system permease protein	1	2	0	0	2	1
Metabolism	Amino acid metabolism	00330 Arginine and proline metabolism [PATH:ko00330]	hyuB N-methylhydantoinase B [EC:3.5.2.14]	1	2	0	0	1	1
Environmental Information Processing	Signal transduction	02020 Two-component system [PATH:ko02020]	mprB two-component system OmpR family sensor histidine kinase MprB [EC:2.7.13.3]	2	2	0	0	1	1
Environmental Information Processing	Membrane transport	02010 ABC transporters [PATH:ko02010]	natB sodium transport system permease protein	1	2	0	2	1	0
Metabolism	Energy metabolism	00190 Oxidative phosphorylation [PATH:ko00190]	ndhB NAD(P)H-quinone oxidoreductase subunit 2 [EC:1.6.5.3]	1	2	0	1	0	1
Metabolism	Metabolism of cofactors and vitamins	00790 Folate biosynthesis [PATH:ko00790]	pabA para-aminobenzoate synthetase component II [EC:2.6.1.85]	1	3	0	1	1	0
Metabolism	Lipid metabolism	01040 Biosynthesis of unsaturated fatty acids [PATH:ko01040]	tesB acyl-CoA thioesterase II [EC:3.1.2.-]	1	3	0	1	0	1
Environmental Information Processing	Membrane transport	02010 ABC transporters [PATH:ko02010]	ABC.NGC.P N-acetylglucosamine transport system permease protein	1	0	4	0	2	1
Metabolism	Amino acid metabolism	00350 Tyrosine metabolism [PATH:ko00350]	E1.1.1.90 aryl-alcohol dehydrogenase [EC:1.1.1.90]	1	2	0	0	1	1

Metabolism	Carbohydrate metabolism	00500 Starch and sucrose metabolism [PATH:ko00500]	E2.4.1.8 mapA maltose phosphorylase [EC:2.4.1.8]	1	1	0	2	1	0
Metabolism	Amino acid metabolism	00400 Phenylalanine tyrosine and tryptophan biosynthesis [PATH:ko00400]	E5.4.99.5 chorismate mutase [EC:5.4.99.5]	1	1	0	1	0	1
Genetic Information Processing	Replication and repair	03410 Base excision repair [PATH:ko03410]	SMUG1 single-strand selective monofunctional uracil DNA glycosylase [EC:3.2.2.-]	1	2	0	2	0	1
Metabolism	Metabolism of cofactors and vitamins	00860 Porphyrin and chlorophyll metabolism [PATH:ko00860]	cbiX sirohydrochlorin cobaltochelatase [EC:4.99.1.3]	1	2	0	2	1	0
Environmental Information Processing	Membrane transport	02010 ABC transporters [PATH:ko02010]	ccmA heme exporter protein A [EC:3.6.3.41]	2	0	4	1	0	1
Metabolism	Carbohydrate metabolism	00620 Pyruvate metabolism [PATH:ko00620]	dld D-lactate dehydrogenase [EC:1.1.1.28]	2	1	0	1	1	0
Metabolism	Carbohydrate metabolism	00030 Pentose phosphate pathway [PATH:ko00030]	gcd quinoprotein glucose dehydrogenase [EC:1.1.5.2]	1	1	0	0	1	1
Metabolism	Carbohydrate metabolism	00630 Glyoxylate and dicarboxylate metabolism [PATH:ko00630]	oxc oxalyl-CoA decarboxylase [EC:4.1.1.8]	1	0	4	1	2	0
Metabolism	Glycan biosynthesis and metabolism	00550 Peptidoglycan biosynthesis [PATH:ko00550]	pbp penicillin-binding protein	2	1	0	1	1	0
Metabolism	Carbohydrate metabolism	00630 Glyoxylate and dicarboxylate metabolism [PATH:ko00630]	rbcL ribulose-bisphosphate carboxylase large chain [EC:4.1.1.39]	0	1	8	0	1	1
Cellular Processes	Cell motility	02030 Bacterial chemotaxis [PATH:ko02030]	tar methyl-accepting chemotaxis protein II aspartate sensor receptor	1	1	0	3	0	1
Metabolism	Lipid metabolism	01040 Biosynthesis of unsaturated fatty acids [PATH:ko01040]	E3.1.2.- [EC:3.1.2.-]	0	1	4	0	1	1
Cellular Processes	Cell growth and death	04210 Apoptosis [PATH:ko04210]	ENDOG endonuclease G mitochondrial	1	2	0	1	0	1
Metabolism	Metabolism of cofactors and vitamins	00760 Nicotinate and nicotinamide metabolism [PATH:ko00760]	K13522 nadM bifunctional NMN adenylyltransferase/nudix hydrolase [EC:2.7.7.1 3.6.1.-]	1	1	0	0	1	1
Environmental Information Processing	Signal transduction	02020 Two-component system [PATH:ko02020]	citT citrate:succinate antiporter	1	1	0	0	1	1

Metabolism	Energy metabolism	00190 Oxidative phosphorylation [PATH:ko00190]	cyoA cytochrome o ubiquinol oxidase subunit II [EC:1.10.3.-]	1	1	0	0	1	1
Environmental Information Processing	Membrane transport	02010 ABC transporters [PATH:ko02010]	gsiD glutathione transport system permease protein	1	1	0	1	1	0
Environmental Information Processing	Signal transduction	02020 Two-component system [PATH:ko02020]	vraR two-component system NarL family vancomycin resistance associated response regulator VraR	1	0	4	0	1	1
Metabolism	Xenobiotics biodegradation and metabolism	00621 Dioxin degradation [PATH:ko00621]	bphB cis-23-dihydrobiphenyl-23-diol dehydrogenase [EC:1.3.1.56]	1	1	0	0	1	1
Environmental Information Processing	Signal transduction	02020 Two-component system [PATH:ko02020]	dpiB citA two-component system CitB family cit operon sensor histidine kinase CitA [EC:2.7.13.3]	1	1	0	1	1	0
Metabolism	Glycan biosynthesis and metabolism	00550 Peptidoglycan biosynthesis [PATH:ko00550]	pbpA penicillin-binding protein 1 [EC:3.4.-.-]	0	1	4	0	1	1
Environmental Information Processing	Membrane transport	02010 ABC transporters [PATH:ko02010]	troB mntB znuC manganese/zinc/iron transport system ATP- binding protein	1	1	0	1	1	0
