# Supplementary material for: Metatranscriptome Sequencing Reveals Insights into the Gene Expression and Functional Potential of Rumen Wall Bacteria
Source: Front Microbiol. 2018 Jan 23;9:43. doi: 10.3389/fmicb.2018.00043 (PMC5787071; doi:10.3389/fmicb.2018.00043)
Supplement: Supplementary file 5 [file Table_5.PDF]

Table S5. Bacterial taxonomy hits for selected genes of interest. Most abundant taxonomy hits (max. 10 hits) were listed. B= Baseline, S= SARA. Hits were normalized by library size. Duplicate reads were removed during quality control.

Phylum	Genus	Total no. of hits	B1	B2	B3	S1	S2	S3	p value	FDR corr
***trxB* (thioredoxin reductase NADPH, [EC.1.8.1.9])**										
Proteobacteria	*Campylobacter*	2300	346	296	1068	396	130	64	0.183	0.326
Actinobacteria	*Atopobium*	1088	205	173	473	126	86	26	0.117	0.326
Actinobacteria	*Bifidobacterium*	756	168	143	236	104	74	32	0.067	0.326
**Glutathione peroxidase [EC.1.11.1.9]**										
Fibrobacteres	*Fibrobacter*	978	159	259	114	170	147	128	0.281	0.371
Bacteroidetes	*Prevotella*	921	84	94	200	251	133	159	0.229	0.337
Firmicutes	*Clostridium*	744	107	136	136	119	84	162	0.430	0.466
Fusobacteria	*Fusobacterium*	675	60	206	82	62	159	106	0.382	0.437
Firmicutes	*Butyrivibrio*	652	82	142	159	151	58	61	0.276	0.366
***scrK* (Fructokinase, [EC.2.7.1.4])**										
Firmicutes	*Butyrivibrio*	7001	615	1266	1859	1167	1072	1022	0.364	0.424
Firmicutes	*Eubacterium*	5869	607	575	1632	1054	1005	996	0.422	0.461
Firmicutes	*Geobacillus*	4404	1171	1309	427	735	127	635	0.181	0.326
Firmicutes	*Paenibacillus*	2268	165	892	277	237	177	520	0.348	0.413
Firmicutes	*Clostridium*	2181	702	339	327	283	234	296	0.130	0.326
Firmicutes	*Listeria*	1337	73	619	141	275	105	124	0.328	0.401
Firmicutes	*Lactococcus*	956	0	937	0	7	0	11	0.217	0.330
Firmicutes	*Bacillus*	936	160	295	91	152	72	166	0.308	0.386
Fusobacteria	*Leptotrichia*	906	0	540	0	367	0	0	0.423	0.462
Chloroflexi	*Chloroflexus*	618	153	240	41	125	23	37	0.171	0.326
**Enolase [EC.4.2.1.11]**										
Spirochaetes	*Borrelia*	4756	1264	824	691	693	486	798	0.156	0.326
Bacteroidetes	*Prevotella*	3720	358	539	1159	463	553	649	0.284	0.372
Thermotogae	*Thermotoga*	2482	502	575	241	683	124	357	0.411	0.453
Bacteroidetes	*Bacteroides*	2172	325	246	586	270	264	481	0.160	0.326
Deinococcus-Thermus	*Meiothermus*	2101	323	385	500	274	241	378	0.034	0.326
Tenericutes	*Candidatus* Phytoplasma	1810	356	710	95	98	109	442	0.300	0.383
Thermotogae	*Thermosipho*	1666	514	510	177	102	105	257	0.135	0.326
Thermotogae	*Fervidobacterium*	1503	390	507	186	102	63	253	0.140	0.326
Firmicutes	*Thermoanaerobacterium*	1458	197	137	582	260	119	163	0.246	0.350
Proteobacteria	*Campylobacter*	1450	301	101	382	195	216	256	0.333	0.405
***fliC* (flagellin)**										
Firmicutes	*Butyrivibrio*	20982	3750	3791	4723	3632	2780	2306	0.110	0.326
Spirochaetes	*Treponema*	9574	1388	720	1850	2757	1236	1623	0.177	0.326
Firmicutes	*Eubacterium*	8368	1682	1170	2077	1462	937	1039	0.104	0.326
Firmicutes	*Clostridium*	4118	806	563	1273	631	333	513	0.086	0.326
Spirochaetes	*Spirochaeta*	2964	695	343	373	720	292	541	0.269	0.362
Firmicutes	*Syntrophomonas*	1598	340	278	336	230	246	167	0.060	0.326
Firmicutes	*Bacillus*	985	164	163	150	225	193	90	0.404	0.448
Firmicutes	*Alkaliphilus*	897	193	202	168	143	101	91	0.018	0.326
Synergistetes	*Thermanaerovibrio*	767	141	150	64	226	76	110	0.364	0.424
Proteobacteria	*Azoarcus*	584	69	89	200	96	32	98	0.181	0.326
***gpmA* (2,3-bisphosphoglycerate-dependent phosphoglycerate mutase, [EC:5.4.2.11])**										
Spirochaetes	*Brachyspira*	3775	750	973	405	526	545	576	0.229	0.337
Spirochaetes	*Treponema*	3595	630	693	468	593	543	668	0.486	0.492
Actinobacteria	*Atopobium*	1455	111	137	255	223	247	482	0.030	0.326
Proteobacteria	*Cronobacter*	1359	211	288	509	174	94	82	0.096	0.326
Proteobacteria	*Bdellovibrio*	1226	302	372	45	153	91	264	0.342	0.409
Proteobacteria	*Alcanivorax*	988	27	49	255	175	274	209	0.154	0.326
Firmicutes	*Eubacterium*	822	78	139	291	154	94	66	0.269	0.362
Proteobacteria	*Ralstonia*	820	42	173	168	263	135	38	0.441	0.474
Firmicutes	*Ruminococcus*	819	191	319	9	144	81	74	0.247	0.351

Firmicutes	*Clostridium*	705	78	69	86	59	241	170	0.144	0.326
***ppdK* (pyruvate orthophosphate dikinase, [EC.2.7.9.1])**										
Firmicutes	*Caldicellulosiruptor*	14859	2764	2427	3409	2568	1305	2386	0.059	0.326
Proteobacteria	*Magnetospirillum*	13576	2204	3275	2018	2137	1498	2444	0.276	0.366
Firmicutes	*Clostridium*	12208	1234	2128	2868	2284	1212	2480	0.449	0.478
Proteobacteria	*Rhodospirillum*	7799	1027	1513	2214	1667	579	800	0.228	0.337
Acidobacteria	*Candidatus Solibacter*	7505	1042	1196	2041	1296	949	980	0.228	0.337
Proteobacteria	*Methylobacterium*	6731	279	1300	2086	752	1116	1197	0.331	0.403
Thermotogae	*Thermosipho*	6166	1027	1277	614	1720	337	1193	0.426	0.464
Dictyoglomi	*Dictyoglomus*	5591	556	1536	982	927	540	1049	0.349	0.413
Bacteroidetes	*Rhodothermus*	5302	681	1390	1159	484	514	1074	0.129	0.326
Thermotogae	*Thermotoga*	4101	508	1064	941	778	337	473	0.205	0.326
**SOD2 (superoxide dismutase Fe-Mn family, [EC.1.15.1.1])**										
Firmicutes	*Bacillus*	1063	101	231	127	236	175	192	0.240	0.345
Firmicutes	*Clostridium*	856	125	120	409	127	51	24	0.167	0.326
Proteobacteria	*Neisseria*	618	111	58	268	20	112	49	0.196	0.326
Firmicutes	*Butyrivibrio*	514	103	75	200	81	45	10	0.141	0.326
**Urease subunit gamma [EC.3.5.1.5]**										
Bacteroidetes	*Flavobacterium*	915	111	39	405	238	39	84	0.338	0.408
Actinobacteria	*Corynebacterium*	748	201	96	327	57	14	53	0.050	0.326
**Urease subunit gamma/beta [EC.3.5.1.5]**										
Proteobacteria	*Helicobacter*	640	146	82	209	73	62	68	0.078	0.326
**Urease subunit beta [EC.3.5.1.5]**										
Firmicutes	*Clostridium*	899	323	118	305	67	34	53	0.037	0.326
**Urease subunit alpha [EC.3.5.1.5]**										
Bacteroidetes	*Flavobacterium*	2427	480	181	1200	306	120	141	0.153	0.326
Firmicutes	*Bacillus*	1945	237	106	795	527	50	230	0.350	0.414
Firmicutes	*Clostridium*	1238	256	135	500	236	38	73	0.141	0.326
Bacteroidetes	*Chitinophaga*	700	211	49	241	78	59	62	0.109	0.326
Proteobacteria	*Rhodopseudomonas*	555	31	118	327	5	33	42	0.118	0.326
***napA* (periplasmatic nitrate reductase, [EC.1.7.99.4])**										
Proteobacteria	*Campylobacter*	2026	211	172	309	646	172	516	0.116	0.326
**Starch phosphorylase [EC.2.4.1.1]**										
Proteobacteria	*Aggregatibacter*	196	27	0	0	72	93	5	0.102	0.320
Proteobacteria	*Aliivibrio*	111	0	51	18	2	21	18	0.237	0.337
Chlamydiae	*Chlamydophila*	343	88	116	14	73	18	35	0.239	0.339
Proteobacteria	*Haemophilus*	330	31	47	64	57	81	50	0.202	0.320
Proteobacteria	*Methylococcus*	351	80	63	50	79	62	18	0.194	0.320
Bacteroidetes	*Prevotella*	195	2	0	9	56	58	70	0.001	0.320
Cyanobacteria	*Synechococcus*	176	38	69	68	1	0	0	0.016	0.320
Thermotogae	*Thermosipho*	550	35	193	36	90	71	124	0.463	0.482
Proteobacteria	*Thioalkalivibrio*	128	17	1	14	51	45	1	0.171	0.320
Proteobacteria	*Variovorax*	56	0	0	9	47	0	0	0.272	0.359
***GdhA* glutamate dehydrogenase NADP [EC.1.4.1.4]**										
Bacteroidetes	*Capnocytophaga*	1003	117	76	118	228	164	299	0.023	0.320
Bacteroidetes	*Bacteroides*	951	118	66	150	232	139	247	0.008	0.320
Proteobacteria	*Neisseria*	771	282	122	145	40	161	20	0.156	0.320
Proteobacteria	*Campylobacter*	476	106	45	109	156	1	59	0.347	0.409
Firmicutes	*Clostridium*	354	10	48	68	12	68	148	0.139	0.320
Bacteroidetes	*Porphyromonas*	198	45	30	0	0	32	91	0.364	0.421
Bacteroidetes	*Parabacteroides*	189	25	46	27	0	36	54	0.437	0.470
Bacteroidetes	*Prevotella*	180	0	0	32	2	86	59	0.131	0.320
Proteobacteria	*Acinetobacter*	166	106	21	23	5	10	1	0.130	0.320
Firmicutes	*Butyrivibrio*	144	37	31	45	0	15	16	0.025	0.320
***glnA* glutamine synthetase [EC.6.3.1.2]**										
Proteobacteria	*Campylobacter*	731	103	90	227	160	75	76	0.308	0.382
Proteobacteria	*Neisseria*	206	51	25	41	1	68	20	0.378	0.432
Firmicutes	*Clostridium*	180	41	17	9	12	57	43	0.284	0.367
Bacteroidetes	*Bacteroides*	84	2	1	18	27	22	14	0.131	0.320
Proteobacteria	*Laribacter*	82	5	1	23	1	42	10	0.333	0.401
Firmicutes	*Ruminococcus*	66	24	16	5	1	21	0	0.238	0.338

Bacteroidetes	*Parabacteroides*	42	0	1	0	0	23	18	0.094	0.320
Proteobacteria	*Methylotenera*	35	8	0	9	0	17	0	0.498	0.499
Bacteroidetes	*Prevotella*	28	0	1	14	4	10	0	0.483	0.490
Proteobacteria	*Chromobacterium*	21	7	1	9	0	1	2	0.069	0.320
***gltD* glutamate synthase NADPH small chain [EC.1.4.1.13 , 1.4.1.14]**										
Firmicutes	*Clostridium*	288	23	16	36	26	59	128	0.109	0.320
Spirochaetes	*Treponema*	199	0	3	27	91	0	78	0.116	0.320
Firmicutes	*Carboxydothermus*	111	11	0	23	42	13	22	0.126	0.320
Proteobacteria	*Desulfovibrio*	88	6	19	27	19	0	18	0.325	0.395
Bacteroidetes	*Bacteroides*	52	0	2	9	0	35	5	0.246	0.344
Bacteroidetes	*Prevotella*	51	0	0	18	0	33	0	0.387	0.438
Firmicutes	*Ruminococcus*	42	11	4	14	12	0	0	0.158	0.320
Firmicutes	*Eubacterium*	30	0	1	23	0	0	5	0.188	0.320
Firmicutes	*Thermoanaerobacterium*	29	12	0	5	2	3	7	0.388	0.439
Deferribacteres	*Denitrovibrio*	17	0	0	9	0	1	7	0.387	0.438
***tnaA* tryptophanase[EC.4.1.99.1]**										
Fusobacteria	*Fusobacterium*	849	175	341	59	94	57	123	0.212	0.320
Bacteroidetes	*Bacteroides*	186	15	3	5	23	48	92	0.089	0.320
**Cellobiose phosphorylase [EC.2.4.1.20]**										
Firmicutes	*Clostridium*	229	0	26	50	0	81	72	0.126	0.326
***dsrA* sulfite reductase [EC.1.8.99.3]**										
Proteobacteria	*Desulfobacterium*	8	2	3	0	0	0	3	0.422	0.459
Proteobacteria	*Desulfohalobium*	1	0	1	0	0	0	0	0.211	0.320
Proteobacteria	*Desulfotalea*	26	0	0	0	26	0	0	0.211	0.320
Firmicutes	*Desulfotomaculum*	1	0	1	0	0	0	0	0.211	0.320
Proteobacteria	*Desulfovibrio*	4	0	4	0	0	1	0	0.211	0.320
Proteobacteria	*Desulfurivibrio*	13	0	6	0	0	0	7	0.456	0.481
***dsrB* sulfite reductase [EC.1.8.99.3]**										
Proteobacteria	*Desulfotalea*	127	23	13	23	20	20	28	0.220	0.326
Firmicutes	*Desulfotomaculum*	7	0	0	0	6	0	1	0.182	0.320
Proteobacteria	*Desulfovibrio*	18	5	3	0	5	6	0	0.172	0.320
Proteobacteria	*Desulfurivibrio*	1	0	0	0	0	0	1	0.211	0.320
***dsrC* sulfite reductase [EC.1.8.99.3]**										
Proteobacteria	*Desulfotalea*	885	163	101	77	195	194	155	0.033	0.320
Proteobacteria	*Desulfovibrio*	160	0	16	9	0	0	135	0.249	0.320
Proteobacteria	*Desulfurivibrio*	771	146	85	55	169	166	150	0.048	0.320
